# Visible-Light-Driven Decarboxylative Coupling of 2*H*-Indazoles with α-Keto Acids without Photocatalysts and Oxidants

**DOI:** 10.1021/acs.joc.4c00176

**Published:** 2024-04-20

**Authors:** Mengyu Niu, Chen Yang, Mingzhu Leng, Qun Cao, Meichao Li, Zhenlu Shen

**Affiliations:** † College of Chemical Engineering, Zhejiang University of Technology, Hangzhou 310014, China; ‡ School of Chemistry, University of Leicester, Leicester LE1 7RH, United Kingdom of Great Britain and Northern Ireland

## Abstract

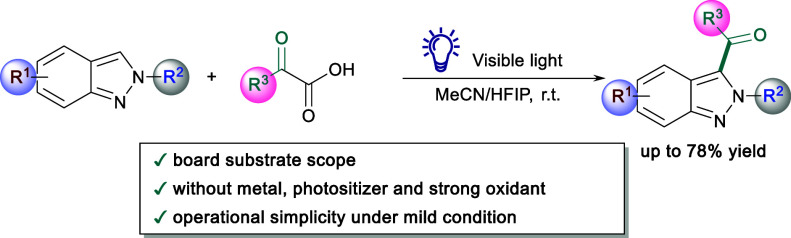

An efficient synthesis of functionalized 3-acyl-2*H*-indazoles via visible-light-induced self-catalyzed energy transfer was developed. This method utilized a self-catalyzed energy transfer process between 2*H*-indazoles and α-keto acids, offering advantages like absence of photosensitizers, metal catalysts, and strong oxidants, broad substrate compatibility, and operational simplicity under mild conditions.

## Introduction

Nitrogen-containing heterocycles play an essential role in the molecular structures of natural compounds,^1^ pharmaceuticals,^2^ and various biologically active substances.^3^ 2*H*-indazole derivatives are known for their diverse range of biological activities, including antitumor,^4^ antibacterial,^5^ anti-inflammatory,^6^ and anticancer^7^ properties. This makes them valuable components in the development of pharmaceuticals and other biologically active compounds. Thus, the pursuit of environmentally friendly and highly efficient methods for synthesizing functionalized 2*H*-indazoles has become a focal point in the field of synthetic methodology.^8^

**Figure 1:**
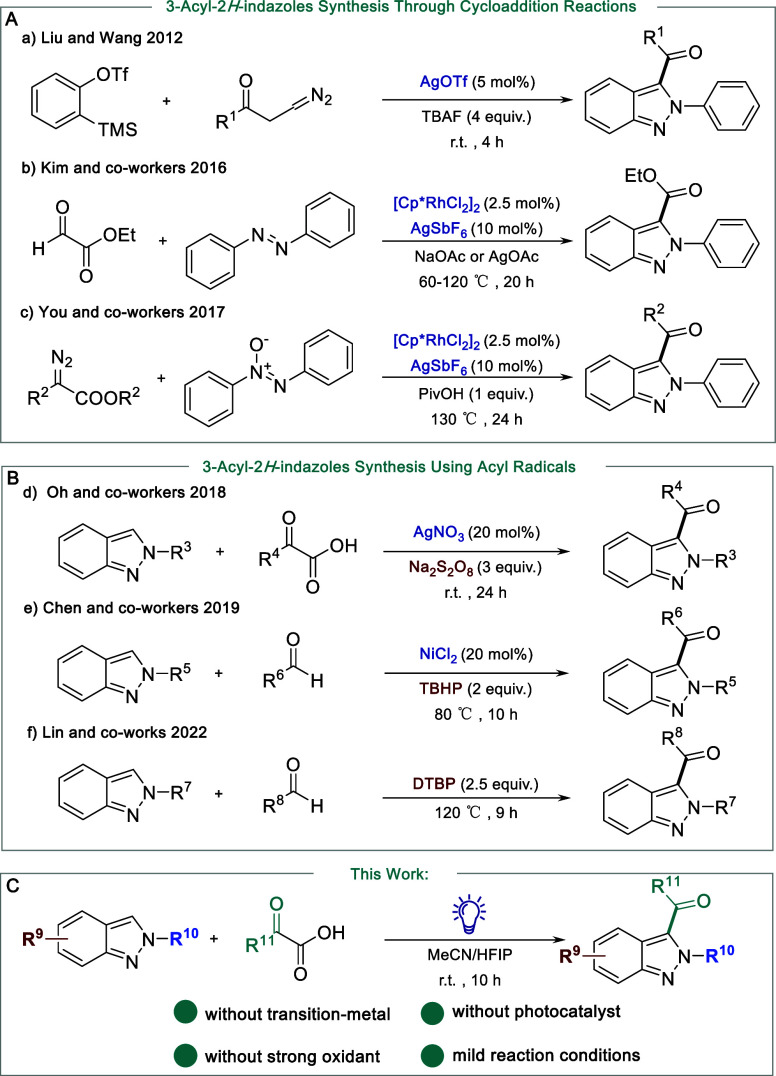
Representative examples of synthesis of 3-acyl-2*H*-indazoles and this work.

In particular, C3-acylated 2*H*-indazoles have been used as polymerase inhibitors^9^ and demonstrated notable value in pharmacology.^10^ While several methods for the synthesis of 3-acyl-2*H*-indazoles have been developed, these methods often involve the use of transition metals, environmentally unfriendly stoichiometric oxidants, or high temperature conditions.^11^ These conditions lead to the generation of chemical wastes and significantly raise the overall manufacturing cost. For example, Liu and co-workers introduced a Ag-catalyzed [3 + 2] cycloaddition for the synthesis of 3-acyl-2*H*-indazoles utilizing benzynes and diazocarbonyl compounds (Figure 1Aa).^12^ Kim and co-workers reported a Rh(III)-catalyzed C–H functionalization of azobenzene with keto aldehydes (Figure 1Ab).^13^ You and co-workers developed a tandem Rh(III)-catalyzed C–H alkylation/intramolecular decarboxylative cyclization method using azoxy compounds and diazoesters (Figure 1Ac).^14^ In addition to the above-mentioned cycloaddition methods, alternative approaches to the synthesis of 3-acyl-2*H*-indazoles involve the direct addition of acyl radicals to 2*H*-indazoles. In these cases, acyl radicals could be generated from various acyl precursors, such as α-keto acids^15^ and aldehydes.^16^ For example, in the presence of strong oxidants (e.g., Na_2_S_2_O_8_ and TBHP), silver nitrate^17^ and NiCl_2_^18^ have been employed for the C-3 substituted acyl 2*H*-indazoles using α-keto acids and aldehydes, respectively (Figure 1Bd,Be). Recently, a metal free method was developed by using aldehyde and di-*tert*-butylperoxide (DTBP) at a high temperature of 120 °C (Figure 1Bf).^19^ To the best of our knowledge, a mild C-3 acylation of 2*H*-indazoles at room temperature without the use of transition metals, oxidants, and photosensitizers has never been reported.

Building on our continued efforts to develop environmentally friendly and efficient methods for the synthesis of functionalized N-heterocycles,^20^ herein, we reported a visible-light-induced self-catalyzed decarboxylative coupling reaction of 2*H*-indazoles and α-keto acids (Figure 1C). Notably, this method did not require the use of photocatalysts or oxidants under mild conditions.

## Results and Discussion

We commenced our study by optimizing reaction conditions, employing a model reaction of 2-phenyl-2*H*-indazole (**1a**) with 2-oxo-2-phenylacetic acid (**2a**). Table 1 summarizes the results of optimization experiments. Initially, degassed acetonitrile (MeCN) was selected as the solvent, and **1a** and **2a** were irradiated with a 6 W LED (380–385 nm) for a duration of 10 h under a N_2_ atmosphere. Pleasingly, this initial experiment yielded our desired product **3aa** in 43% yield (Table 1, entry 1). Further optimization was carried out to determine the optimal conditions. Various solvents were systematically investigated, including 1,4-dioxane, dichloroethane (DCE), dimethyl sulfoxide (DMSO) (Table 1, entries 2–4). Among these solvents, MeCN emerged as the most effective solvent.

**Table 1:**
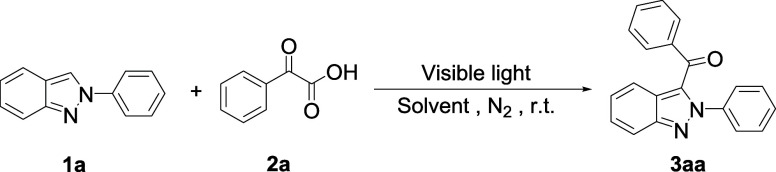
Optimization of Reaction Conditions^g^

entry	**2a** (equiv)	light source (nm)	additive	solvent	yield (%)^a^
1	2.0	380–385		MeCN	43
2	2.0	380–385		1,4-dioxane	14
3	2.0	380–385		DCE	26
4	2.0	380–385		DMSO	N.R.
5	3.0	380–385		MeCN	50
6	4.0	380–385		MeCN	57
7	5.0	380–385		MeCN	62
8	6.0	380–385		MeCN	62
9	5.0	380–385	4CzIPN (5 mol %)	MeCN	60
10	5.0	380–385	5 Å-MS (50 mg)	MeCN	54
11	5.0	380–385	DTBP (2 equiv)	MeCN	60
12	5.0	380–385	K_2_S_2_O_8_ (2 equiv)	MeCN	56
13^b^	5.0	380–385		MeCN	57
14^c^	5.0	380–385		MeCN	67
15^d^	5.0	380–385		MeCN	61
16^c^	5.0	380–385	^t^BuOK (2 equiv)	MeCN	41
17^c^	5.0	380–385	K_2_CO_3_ (2 equiv)	MeCN	38
18^c^	5.0	360–365		MeCN	57
19^c^	5.0	390–395		MeCN	50
20^c^	5.0	405–410		MeCN	56
21^c^	5.0	420–425		MeCN	68
22^c^	5.0	450–455		MeCN	50
23^c^	5.0	420–425		MeCN/HFIP (1:1)	52
24^c^	5.0	420–425		MeCN/HFIP (3:1)	71
25^c^	5.0	420–425		MeCN/HFIP (5:1)	68
26^c^	5.0	420–425		MeCN/TFA (3:1)	6
27^c^	5.0	420–425		MeCN/TfOH (3:1)	N.R.
28^c^^,^^e^	5.0	420–425		MeCN/HFIP (3:1)	45
29^c^^,^^f^	5.0	420–425		MeCN/HFIP (3:1)	71

a Yield of 3aa was determined by GC using docosane as internal standard. N.R.: no reaction.

b Solvent (3.0 mL).

c Solvent (4.0 mL).

d Solvent (5.0 mL).

e Reaction time was 6 h.

f Reaction time was 16 h.

g Reaction conditions: 1a (0.2 mmol), 2a (amount as specified), solvent as specified (2.0 mL), room temperature, nitrogen atmosphere, 10 h.

Furthermore, the influence of the ratio between **1a** and **2a** on the reaction was explored. As demonstrated in Table 1 (entries 5–7), increasing the ratio between **1a** and **2a** from 1:2 to 1:5 led to an enhanced yield of **3aa** from 43 to 62%. However, when the ratio was further increased to 1:6 (Table 1, entry 8), there was no further improvement in the yield of **3aa**. Therefore, the optimal ratio between **1a** and **2a** was identified as 1:5. It was known that α-keto acids (e.g., **2a**) could undergo decarboxylation under light irradiation, leading to the formation of acyl radicals.^21^ Indeed, during the reaction optimization, benzil was identified as one of the byproducts (details see Figure 4a), which could be generated from acyl radicals. It was found that the concentration of starting materials could also affect the efficiency of reaction. As indicated in Table 1 (entries 13–15), the amount of solvent utilized in the reaction (2–5 mL) also had an impact on the performance of the reaction, with the optimal solvent amount of 4 mL for 0.2 mmol scale.

It has been reported that the addition of oxidants (e.g., K_2_S_2_O_8_, organic peroxides) and photoinitiators could help the generation of free acyl radicals from **2a**, facilitating subsequent free radical coupling reactions.^22^ Thus, the addition of various additives such as photoinitiators (4CzIPN), oxidants (e.g., K_2_S_2_O_8_, DTBP), bases (^t^BuOK, K_2_CO_3_), and acids (trifluoroacetic acid, triflic acid) were examined under our optimized conditions (Table 1, entries 9–12, 16–17, 26–27). However, no improvement in the yield of product **3aa** was found. During the optimization process, different wavelengths of the light source, ranging from 360 to 455 nm, were tested. The results indicated that the wavelength (420–425 nm) was the most effective light source for the reaction (Table 1, entries 18–22). To further improve the yield of **3aa**, degassed hexafluoroisopropanol (HFIP) was added as cosolvent. Pleasingly, the solvent mixture of MeCN and HFIP (*V*_MeCN_:*V*_HFIP_ = 3:1) led to the highest yield of **3aa** at 71% (Table 1, entries 23–25). This improvement could be attributed to the previously reported role of HFIP as a radical stabilizer.^23^ Optimizing the reaction conditions revealed that the optimal reaction time is 10 h, as indicated in Table 1 (Table 1, entries 24, 28, and 29). Interestingly, extending the reaction time beyond 10 to 16 h did not result in an improvement in the yield of **3aa**.

**Figure 2:**
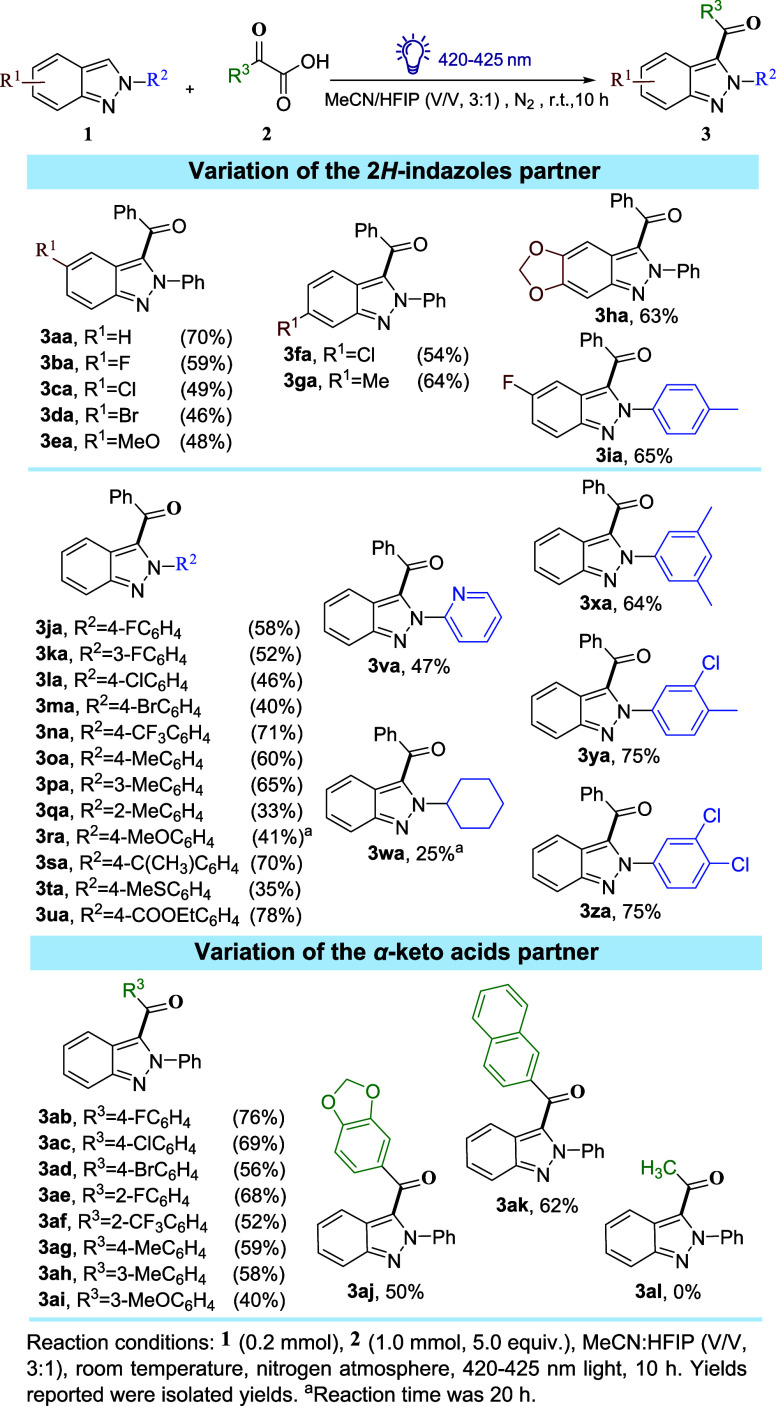
Substrate scope for the synthesis of 3-acyl-2*H*-indazoles.

With the optimized reaction condition in hand, the applicability of the synthetic approach to a range of functionalized 2*H*-indazoles was explored (Figure 2). The functional group tolerance of 2*H*-indazoles at C-5 and C-6 position was also examined. The introduction of both halogen atoms (−F, −Cl, −Br) (**3ba**–**3da**) or electron-donating groups (−OMe, −Me, −OMeO−) (**3ea**–**3ha**) at the C-5 and C-6 position of 2*H*-indazoles resulted in the target products with the yields ranging from 48 to 64%. The synthesis of product **3ia** showcased the feasibility of conducting the synthesis using 2*H*-indazoles containing multiple functional groups on various sites. Various indazoles bearing aromatic rings that were functionalized with diverse functional groups (e.g., −Me, −OMe, −SMe, −F, −Cl, −Br, −CF_3_, −COOEt, −phenyl) along with heteroaryl substituents (e.g., pyridyl) at the 2*N* position were effectively converted into their corresponding 3-acyl-2*H*-indazoles in fair to good yields (**3ja**–**3za**, 33–78%). It was noted that substrates with ortho-methyl substituted phenyl groups at 2*N* position led to lower yields of the products (e.g., **3qa**) compared to the products with meta- and para-substituted phenyl groups (e.g., **3oa**, **3pa**). This suggested that steric hindrance at the ortho position affected the reaction efficiency. Additionally, when aromatic functional group at 2*N* position was switched to aliphatic substituent, as in the case of 2-(cyclohexyl)-2*H*-indazole (**1w**), a poor isolated yield of **3wa** was obtained (25%), even after extending the reaction time to 20 h. It was proposed that the broader light absorption range of C3-acylated 2*H*-indazole products (e.g., **3aa** and **3wa**) could inhibit the continuous formation of desired product (for the product inhabitation effect and UV–vis spectrum, see Figures S2–S4 in Supporting Information).

After the exploration of the scope of 2*H*-indazole derivatives, a variety of aryl and hetaryl α-keto acids were synthesized (see Experimental Section). These α-keto acids were then investigated for the photoinitiated decarboxylative radical acylation reaction with **1a**. It was found that our reaction system exhibited versatility and accommodated a wide range of α-keto acids, allowing them to smoothly undergo the acylation process and yield the desired acylated 2*H*-indazole derivatives (**3ab**–**3ak**) with moderate to good yields. Aromatic α-keto acids bearing electron-withdrawing groups (e.g., −F, −Cl, −Br, −CF_3_) at the ortho- and para-positions of the aromatic rings provided the desired products (e.g., **3ab**–**3af**) with yields ranging from 52 to 76%. Moreover, α-keto acids with electron-donating groups (−OMe, −Me, −OMeO−) were also effective acylating reagents, although they resulted in slightly lower yields (40–59%) of acylated 2*H*-indazoles (**3ag**–**3aj**). Pleasingly, a good yield (62%) of **3ak** was obtained when naphthyl α-keto acid (**2k**) was used. Unfortunately, aliphatic α-keto acids, such as 2-oxopropanoic acid (**2l**), could not lead to the desired products **3al** using our method. To further establish the scalability of our method, we performed large-scale reactions using different quantities of **1a** as starting materials, ranging from 3 (0.58 g) to 6 mmol (1.17 g). The isolated yields of **3aa** were 64 and 56%, respectively (for details, please refer to the SI).

**Figure 3:**
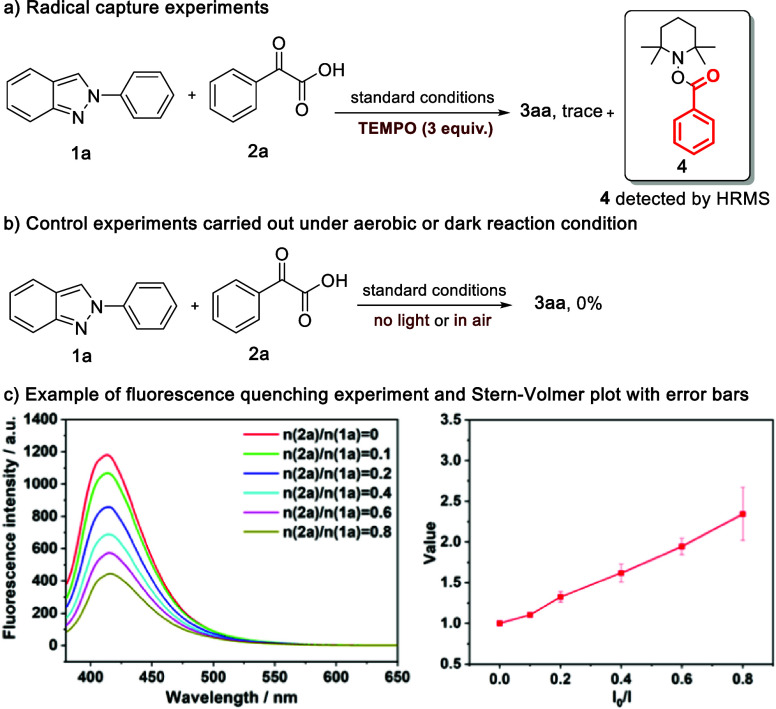
Control experiments.

To gain preliminary insight into the reaction mechanism, a series of control experiments were conducted. When the free radical inhibitor TEMPO (2,2,6,6-tetramethylpiperidinooxyl) was introduced (Figure 3a), the transformation was completely suppressed. The HRMS analysis of the reaction mixture confirmed the formation of acyl radical during the photoreaction, as evidenced by the detection of compound **4**, an adduct of TEMPO and acyl radical ([M + H]^+^ found 262.1800, calculated 262.1802, detailed HRMS see Figure S1). This finding strongly suggested that the reaction likely proceeded through a free radical pathway.

**Figure 4:**
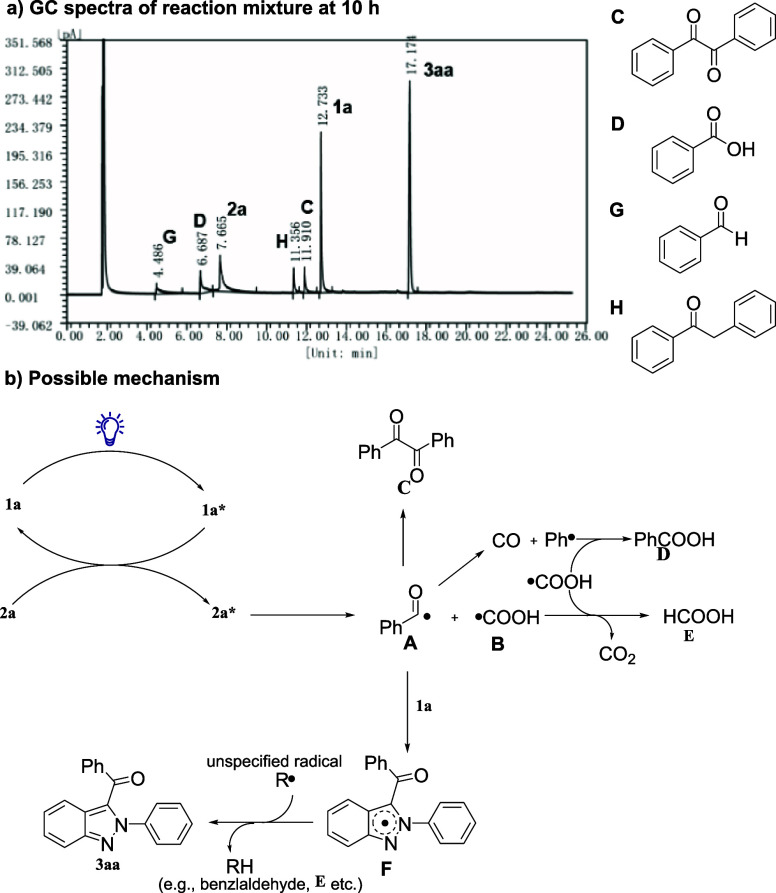
Organic intermediates determined by GC-MS and the proposed mechanism.

As depicted in Figure 3b, when the light source was removed from the reaction, product **3aa** was not detected, confirming that the reaction was indeed light driven. Interestingly, when the reaction was conducted in an air atmosphere, the reaction was quenched and did not proceed. This observation diverged from a method developed by He and co-workers,^24^ where oxygen played a crucial role in visible-light-induced aerobic acylation of quinoxalin-2(1*H*)-ones with α-keto acids. In this process, an acyl radical and byproduct H_2_O_2_ were generated through a hydrogen-atom-transfer (HAT) process via excited-state singlet oxygen (^1^O_2_). Jin and co-workers reported the generation of hydrogen gas during the synthesis of quinazolinone derivative under photoinduced conditions using α-keto acids.^25^ To investigate the possibility of formation of hydrogen gas from the H• radical, the gas mixture from the headspace of the reaction mixture was analyzed by GC with a TCD detector (Figure S7). However, only the formation of CO_2_ and minor CO were confirmed, and no hydrogen gas was generated in our system. In addition, we carried out fluorescence quenching experiments involving **1a** and **2a**. Upon the addition of **2a** to the visible light irradiated **1a**, the fluorescence intensity of **1a** decreased (Figure 3c, see SI for a detailed procedure). The straight Stern–Volmer plot of the quenching experiment in Figure 3c indicated that the excited state of **1a** could be quenched by **2a**. Further fluorescence quenching study demonstrated that no energy transfer between **3aa** and **2a** (Figure S6 in Supporting Information). Moreover, GC-MS analysis of reaction mixture at 10 h (reaction condition as listed in entry 24, Table 1) revealed the generation of byproducts such as benzaldehyde, benzoic acid and benzil during the reaction (Figure 4a). This was consistent with previous reports that under light irradiation, α-keto acids could undergo a complex decomposition mechanism and form a variety of radicals and organic intermediates.^24−26^

Based on the above obtained results and relevant literature studies,^26,27,28^ a plausible reaction mechanism was proposed (Figure 4b). Initially, **1a** absorbs visible light, transitioning to an exciting state (1a*). Subsequently, the ground state **2a** undergoes energy transfer from 1a*, leading to the excited state 2a*, which could homolyze to form an acyl radical (**A**) and carboxyl radical (**B**).^26,27^

The acyl radical (**A**) could then form benzil (**C**) or further decompose to CO and phenyl radical (Ph•), with the later react with **B** to form benzoic acid (**D**). In the meantime, **B** could react with another **B** to form CO_2_ and formic acid (**E**).^26^ The generated acyl radical **A** could attack the C-3 position of **1a**, forming radical intermediate **F**. Finally, the extraction of H• from intermediate **F** took place with radical R• (e.g., **A**, **B**, or other radicals generated during the decomposition of α-keto acids), leading to the formation of the final target product **3aa**.

## Conclusions

In conclusion, we have presented a novel and efficient method for visible-light-induced synthesis of functionalized 3-acyl-2*H*-indazoles. Notably, the utilization of a self-catalyzed energy transfer process between 2*H*-indazoles and α-keto acids offered this method several advantages, induced organic synthesis without photocatalyst conditions. This method demonstrated a broad substrate scope and operational simplicity under mild conditions. Further investigation on visible-light-induced organic synthesis under without photocatalyst conditions is currently underway in our laboratory.

## Experimental Section

### General Information

^1^H NMR spectra and ^13^C NMR spectra were recorded on a Bruker AVANCE NEO (400 MHz/500 MHz/600 MHz) spectrometer and Bruker AVANCE NEO (100 MHz/125 MHz/150 MHz) spectrometer at 25 °C, respectively. High resolution mass spectra (HRMS) were measured with an Agilent 6230 TOF instrument.

### Safety Statement

Caution! Aldehydes and anilines have irritating odors, and inhalation can cause damage to the body. Therefore, weighing and transferring these chemicals should be carried out within a fume hood. Additionally, exposure to light sources can be harmful to the eyes, necessitating the use of protective goggles.

### General Procedure for the Synthesis of Functionalized 3-Acyl-2*H*-indazoles

In a clean Schlenk flask, 2*H*-indazoles (0.2 mmol) and α-keto acids (5 equiv., 1.0 mmol) were placed. The Schlenk flask was evacuated and purged with nitrogen three times using a Schlenk line. Subsequently, 3 mL of degassed MeCN and 1 mL of degassed HFIP were added under nitrogen charging conditions, and the flask was tightly sealed. The reaction was conducted under 420–425 nm light for 10–20 h. After completion of the reaction, the solution was concentrated, and flash chromatography was performed using petroleum ether (PE)/ethyl acetate (EA) = 100:1–15:1 as eluent.

#### Phenyl(2-phenyl-2*H*-indazol-3-yl)methanone (**3aa**)^17^

The product **3aa** was prepared by the general procedure with **1a** (38.8 mg, 0.2 mmol). 41.7 mg (70%); white solid; ^1^H NMR (400 MHz, CDCl_3_): δ 7.91–7.86 (m, 3H), 7.62–7.58 (m, 1H), 7.56–7.53 (m, 2H), 7.48–7.37 (m, 7H), 7.20–7.17 (m, 1H); ^13^C {^1^H} NMR (100 MHz, CDCl_3_): δ 186.1, 148.7, 140.6, 137.9, 133.7, 132.4, 130.0, 129.2, 129.0, 128.8, 127.1, 125.7, 125.1, 124.2, 120.7, 118.7.

#### (5-Fluoro-2-phenyl-2*H*-indazol-3-yl)(phenyl)methanone (**3ba**)^17^

The product **3ba** was prepared by the general procedure with **1b** (42.5 mg, 0.2 mmol). 36.8 mg (59%); white solid; ^1^H NMR (400 MHz, CDCl_3_): δ 7.89–7.83 (m, 3H), 7.63–7.59 (m, 1H), 7.54–7.40 (m, 7H), 7.19 (td, *J*_*1*_ = 9.1 Hz, *J*_*2*_ = 2.4 Hz, 1H), 6.95 (dd, *J*_*1*_ = 9.2 Hz, *J*_*2*_ = 2.4 Hz, 1H); ^13^C {^1^H} NMR (100 MHz, CDCl_3_): δ 185.7, 160.4 (d, *J* = 243.4 Hz), 146.0, 140.5, 137.6, 133.8, 132.7 (d, *J* = 8.4 Hz), 129.9, 129.22, 129.17, 128.9, 125.5, 124.1 (d, *J* = 12.0 Hz), 120.9 (d, *J* = 9.9 Hz), 118.8 (d, *J* = 28.7 Hz), 103.7 (d, *J* = 25.5 Hz).

#### (5-Chloro-2-phenyl-2*H*-indazol-3-yl)(phenyl)methanone (**3ca**)

The product **3ca** was prepared by the general procedure with **1c** (45.8 mg, 0.2 mmol). 32.3 mg (49%); white solid; ^1^H NMR (400 MHz, CDCl_3_): δ 7.85–7.82 (m, 3H), 7.64–7.60 (m, 1H), 7.53–7.38 (m, 8H), 7.34–7.31 (m, 1H); ^13^C {^1^H} NMR (100 MHz, CDCl_3_): δ 185.7, 147.0, 140.3, 137.5, 134.0, 132.1, 131.1, 130.0, 129.3 (3C), 128.9, 128.7, 125.5, 124.5, 120.2, 119.5. HRMS (ESI): *m*/*z* calcd for C_20_H_14_ClN_2_O [M + H]^+^ 333.0789 Found 333.0792.

#### (5-Bromo-2-phenyl-2*H*-indazol-3-yl)(phenyl)methanone (**3da**)^29^

The product **3da** was prepared by the general procedure with **1d** (54.7 mg, 0.2 mmol). 34.1 mg (46%); white solid; ^1^H NMR (400 MHz, CDCl_3_): δ 7.85–7.82 (m, 2H), 7.77 (d, *J* = 9.1 Hz, 1H), 7.64–7.58 (m, 2H), 7.53–7.39 (m, 8H); ^13^C {^1^H} NMR (100 MHz, CDCl_3_): δ 185.8, 147.1, 140.3, 137.5, 134.0, 131.9, 131.1, 130.0, 129.3, 128.9, 125.5, 125.2, 122.9, 120.3, 119.2.

#### (5-Methoxy-2-phenyl-2*H*-indazol-3-yl)(phenyl)methanone (**3ea**)

The product **3ea** was prepared by the general procedure with **1e** (44.9 mg, 0.2 mmol). 31.0 mg (48%); yellow solid; ^1^H NMR (400 MHz, CDCl_3_): δ 7.83–7.80 (m, 2H), 7.77 (d, *J* = 9.0 Hz, 1H), 7.57–7.53 (m, 1H), 7.51–7.48 (m, 2H), 7.43–7.32 (m, 5H), 7.07 (dd, *J*_*1*_ = 9.3 Hz, *J*_*2*_ = 2.4 Hz, 1H), 6.64 (d, *J* = 2.4 Hz, 1H), 3.69 (s, 3H); ^13^C {^1^H} NMR (100 MHz, CDCl_3_): δ 186.2, 157.8, 145.6, 140.7, 138.1, 133.3, 131.6, 129.8, 129.1, 128.7, 128.6, 125.5, 125.4, 122.3, 120.0, 97.1, 55.4. HRMS (ESI): *m*/*z* calcd for C_21_H_17_N_2_O_2_ [M + H]^+^ 329.1285 Found 329.1283.

#### (6-Chloro-2-phenyl-2*H*-indazol-3-yl)(phenyl)methanone (**3fa**)

The product **3fa** was prepared by the general procedure with **1f** (45.8 mg, 0.2 mmol). 35.6 mg (54%); white solid; ^1^H NMR (400 MHz, CDCl_3_): δ 7.87–7.83 (m, 3H), 7.61 (t, *J* = 7.4 Hz, 1H), 7.53–7.40 (m, 7H), 7.32 (d, *J* = 9.0 Hz, 1H), 7.13 (d, *J* = 9.0 Hz, 1H); ^13^C {^1^H} NMR (100 MHz, CDCl_3_): δ 185.9, 148.8, 140.3, 137.6, 134.0, 133.2, 132.8, 130.0, 129.3 (3C), 128.9, 126.5, 125.6, 122.5, 122.0, 117.5. HRMS (ESI): *m*/*z* calcd for C_20_H_14_ClN_2_O [M + H]^+^ 333.0789 Found 333.0792.

#### (6-Methyl-2-phenyl-2*H*-indazol-3-yl)(phenyl)methanone (**3ga**)^17^

The product **3ga** was prepared by the general procedure with **1g** (41.7 mg, 0.2 mmol). 39.7 mg (64%); white solid; ^1^H NMR (400 MHz, CDCl_3_): δ 7.91–7.88 (m, 2H), 7.66–7.60 (m, 2H), 7.57–7.54 (m, 2H), 7.50–7.41 (m, 5H), 7.27 (d, *J* = 7.8 Hz, 1H), 7.05 (dd, *J*_*1*_ = 8.8 Hz, *J*_*2*_ = 1.5 Hz, 1H), 2.51 (s, 3H); ^13^C {^1^H} NMR (100 MHz, CDCl_3_): δ 186.0, 149.2, 140.6, 137.9, 137.1, 133.5, 132.1, 129.9, 129.1, 128.8, 128.6, 128.0, 125.5, 122.6, 120.1, 116.8, 22.1.

#### Phenyl(2-phenyl-2*H*-[1,3]dioxolo[4,5-*f*]indazol-3-yl)methanone (**3ha**)^17^

The product **3ha** was prepared by the general procedure with **1h** (47.7 mg, 0.2 mmol). 42.7 mg (63%); yellow solid; ^1^H NMR (400 MHz, CDCl_3_): δ 7.84–7.81 (m, 2H), 7.60–7.55 (m, 1H), 7.48–7.42 (m, 4H), 7.41–7.33 (m, 3H), 7.11 (s, 1H), 6.60 (s, 1H), 6.00 (s, 2H); ^13^C {^1^H} NMR (100 MHz, CDCl_3_): δ 186.2, 149.8, 148.2, 146.3, 140.6, 137.9, 133.5, 132.1, 129.9, 129.1, 128.8, 128.5, 125.3, 121.2, 101.6, 95.8, 94.7.

#### (5-Fluoro-2-(*p*-tolyl)-2*H*-indazol-3-yl)(phenyl)methanone (**3ia**)

The product **3ia** was prepared by the general procedure with **1i** (45.3 mg, 0.2 mmol). 42.6 mg (65%); white solid; ^1^H NMR (400 MHz, CDCl_3_): δ 7.88–7.84 (m, 3H), 7.65–7.60 (m, 1H), 7.48 (t, *J* = 7.9 Hz, 2H), 7.42–7.38 (m, 2H), 7.22 (d, *J* = 8.2 Hz, 2H), 7.17 (td, *J*_*1*_ = 9.2 Hz, *J*_*2*_ = 2.4 Hz, 1H), 6.92 (dd, *J*_*1*_ = 9.2 Hz, *J*_*2*_ = 2.4 Hz, 1H), 2.38 (s, 3H); ^13^C {^1^H} NMR (100 MHz, CDCl_3_): δ 185.8, 160.7 (d, *J* = 243.1 Hz), 145.9, 139.3, 138.1, 137.7, 133.8, 132.6 (d, *J* = 8.3 Hz), 129.9, 129.8, 128.9, 125.3, 124.0 (d, *J* = 12.0 Hz), 120.9 (d, *J* = 10.0 Hz), 118.7 (d, *J* = 28.8 Hz), 103.7 (d, *J* = 25.4 Hz), 21.3. HRMS (ESI): *m*/*z* calcd for C_21_H_16_FN_2_O [M + H]^+^ 331.1241. Found 331.1240.

#### (2-(4-Fluorophenyl)-2*H*-indazol-3-yl)(phenyl)methanone (**3ja**)

The product **3ja** was prepared by the general procedure with **1j** (42.5 mg, 0.2 mmol). 36.3 mg (58%); white solid; ^1^H NMR (400 MHz, (CD_3_)_2_SO): δ 7.05 (d, *J* = 8.8 Hz, 1H), 6.98–6.95 (m, 2H), 6.86–6.78 (m, 3H), 6.68 (t, *J* = 7.8 Hz, 2H), 6.60–6.56 (m, 1H), 6.50–6.44 (m, 2H), 6.39–6.38 (m, 2H); ^13^C {^1^H} NMR (100 MHz, (CD_3_)_2_SO): δ 185.4, 161.9 (d, *J* = 245.0 Hz), 147.8, 137.5, 136.7 (d, *J* = 3.1 Hz), 133.7, 132.2, 129.6, 128.8, 127.9 (d, *J* = 9.0 Hz), 127.2, 125.3, 123.5, 120.2, 118.3, 116.0 (d, *J* = 23.1 Hz). HRMS (ESI): *m*/*z* calcd for C_20_H_14_FN_2_O [M + H]^+^ 317.1085. Found 317.1090.

#### (2-(3-Fluorophenyl)-2*H*-indazol-3-yl)(phenyl)methanone (**3ka**)

The product **3ka** was prepared by the general procedure with **1k** (42.5 mg, 0.2 mmol). 33.0 mg (52%); white solid; ^1^H NMR (400 MHz, CDCl_3_): δ 7.90–7.87 (m, 3H), 7.64 (t, *J* = 7.5 Hz, 1H), 7.49 (t, *J* = 7.7 Hz, 2H), 7.41–7.29 (m, 5H), 7.20–7.17 (m, 1H), 7.15–7.10 (m, 1H); ^13^C {^1^H} NMR (100 MHz, CDCl_3_): δ 185.8, 162.6 (d, *J* = 246.9 Hz), 148.7, 141.7 (d, *J* = 10.0 Hz), 137.7, 133.8, 132.4, 130.3 (d, *J* = 8.9 Hz), 129.9, 128.8, 127.4, 125.4, 124.1, 121.4 (d, *J* = 3.3 Hz), 120.6, 118.6, 116.0 (d, *J* = 21.0 Hz), 113.3 (d, *J* = 25.0 Hz). HRMS (ESI): *m*/*z* calcd for C_20_H_14_FN_2_O [M + H]^+^ 317.1085. Found 317.1083.

#### (2-(4-Chlorophenyl)-2*H*-indazol-3-yl)(phenyl)methanone (**3la**)^18^

The product **3la** was prepared by the general procedure with **1l** (45.8 mg, 0.2 mmol). 30.2 mg (46%); white solid; ^1^H NMR (400 MHz, (CD_3_)_2_SO): δ 7.91 (d, *J* = 8.8 Hz, 1H), 7.86–7.84 (m, 2H), 7.74–7.70 (m, 1H), 7.66–7.62 (m, 2H), 7.58–7.54 (m, 4H), 7.47–7.43 (m, 1H), 7.27–7.20 (m, 2H); ^13^C {^1^H} NMR (100 MHz, (CD_3_)_2_SO): δ 185.4, 148.0, 139.1, 137.4, 133.8, 133.6, 132.1, 129.6, 129.1, 128.8, 127.4, 127.3, 125.4, 123.5, 120.2, 118.4.

#### (2-(4-Bromophenyl)-2*H*-indazol-3-yl)(phenyl)methanone (**3ma**)^17^

The product **3ma** was prepared by the general procedure with **1m** (54.7 mg, 0.2 mmol). 30.2 mg (40%); white solid; ^1^H NMR (400 MHz, (CD_3_)_2_SO): δ 7.91 (d, *J* = 8.8 Hz, 1H), 7.86–7.84 (m, 2H), 7.74–7.68 (m, 3H), 7.58–7.53 (m, 4H), 7.46–7.42 (m, 1H), 7.26–7.19 (m, 2H); ^13^C {^1^H} NMR (100 MHz, (CD_3_)_2_SO): δ 185.4, 148.0, 139.5, 137.4, 133.9, 132.10, 132.07, 129.6, 128.8, 127.7, 127.3, 125.4, 123.5, 122.1, 120.2, 118.4.

#### Phenyl(2-(4-(trifluoromethyl)phenyl)-2*H*-indazol-3-yl)methanone (**3na**)^17^

The product **3na** was prepared by the general procedure with **1n** (52.4 mg, 0.2 mmol). 51.6 mg (71%); White solid; ^1^H NMR (400 MHz, CDCl_3_): δ 7.94–7.87 (m, 3H), 7.73–7.65 (m, 5H), 7.54–7.49 (m, 2H), 7.43–7.38 (m, 1H), 7.33–7.31 (m, 1H), 7.22–7.18 (m, 1H); ^13^C {^1^H} NMR (100 MHz, CDCl_3_): δ 185.8, 149.0, 143.4, 137.7, 134.1, 132.5, 131.0 (q, *J* = 32.9 Hz), 130.1, 129.0, 127.6, 126.4 (q, *J* = 3.7 Hz), 126.1, 125.7, 124.3, 123.8 (d, *J* = 270.6 Hz), 120.7, 118.8.

#### Phenyl(2-(*p*-tolyl)-2*H*-indazol-3-yl)methanone (**3oa**)^17^

The product **3oa** was prepared by the general procedure with **1o** (41.7 mg, 0.2 mmol). 37.5 mg (60%); white solid; ^1^H NMR (400 MHz, CDCl_3_): δ 7.90–7.87 (m, 3H), 7.64–7.60 (m, 1H), 7.50–7.46 (m, 2H), 7.44–7.41 (m, 2H), 7.39–7.36 (m, 1H), 7.35–7.33 (m, 1H), 7.23 (d, *J* = 8.2 Hz, 2H), 7.19–7.15 (m, 1H), 2.39 (s, 3H); ^13^C {^1^H} NMR (100 MHz, CDCl_3_): δ 186.1, 148.6, 139.2, 138.2, 138.0, 133.7, 132.3, 130.1, 129.8, 128.8, 127.0, 125.4, 125.0, 124.1, 120.7, 118.6, 21.3.

#### Phenyl(2-(*m*-tolyl)-2*H*-indazol-3-yl)methanone (**3pa**)^30^

The product **3pa** was prepared by the general procedure with **1p** (41.7 mg, 0.2 mmol). 40.6 mg (65%); white solid; ^1^H NMR (400 MHz, CDCl_3_): δ 7.90–7.86 (m, 3H), 7.63–7.59 (m, 1H), 7.49–7.44 (m, 2H), 7.40–7.37 (m, 3H), 7.29–7.27 (m, 2H), 7.23–7.16 (m, 2H), 2.38 (s, 3H); ^13^C {^1^H} NMR (100 MHz, CDCl_3_): δ 186.2, 148.6, 140.5, 139.4, 138.0, 133.7, 132.4, 130.0, 129.9, 129.0, 128.7, 127.1, 126.2, 125.1, 124.2, 122.8, 120.7, 118.6, 21.4.

#### Phenyl(2-(*o*-tolyl)-2*H*-indazol-3-yl)methanone (**3qa**)

The product **3qa** was prepared by the general procedure with **1q** (41.7 mg, 0.2 mmol). 20.6 mg (33%); white solid; ^1^H NMR (400 MHz, CDCl_3_): δ 7.91–7.85 (m, 3H), 7.65–7.61 (m, 1H), 7.49 (t, *J* = 7.6 Hz, 2H), 7.42–7.29 (m, 6H), 7.22–7.18 (m, 1H), 2.12 (s, 3H); ^13^C {^1^H} NMR (100 MHz, CDCl_3_): δ 185.3, 148.5, 140.2, 138.1, 134.8, 133.5, 133.4, 130.9, 129.9, 129.7, 128.7, 127.2, 126.9, 126.5, 125.1, 123.0, 120.9, 118.8, 17.7. HRMS (ESI): *m*/*z* calcd for C_21_H_17_N_2_O [M + H]^+^ 313.1335. Found 313.1334.

#### (2-(4-Methoxyphenyl)-2*H*-indazol-3-yl)(phenyl)methanone (**3ra**)^17^

The product **3ra** was prepared by the general procedure with **1r** (44.9 mg, 0.2 mmol). 26.8 mg (41%); white solid; ^1^H NMR (400 MHz, CDCl_3_): δ 7.89–7.86 (m, 3H), 7.61 (t, *J* = 7.5 Hz, 1H), 7.49–7.45 (m, 4H), 7.40–7.33 (m, 2H), 7.19–7.15 (m, 1H), 6.95–6.91 (m, 2H), 3.82 (s, 3H); ^13^C {^1^H} NMR (100 MHz, CDCl_3_): δ 186.2, 160.0, 148.5, 138.0, 133.8, 133.7, 132.2, 130.1, 128.8, 127.0, 126.8, 125.0, 124.1, 120.7, 118.5, 114.4, 55.7.

#### (2-(4-(*tert*-Butyl)phenyl)-2*H*-indazol-3-yl)(phenyl)methanone (**3sa**)

The product **3sa** was prepared by the general procedure with **1s** (50.1 mg, 0.2 mmol). 49.2 mg (70%); white solid; ^1^H NMR (400 MHz, CDCl_3_): δ 7.90–7.86 (m, 3H), 7.62–7.58 (m, 1H), 7.48–7.35 (m, 8H), 7.20–7.16 (m, 1H), 1.32 (s, 9H); ^13^C {^1^H} NMR (100 MHz, CDCl_3_): δ 186.2, 152.2, 148.6, 138.1, 133.6, 132.2, 131.3, 130.0, 128.7, 127.0, 126.2, 125.2, 125.1, 124.2, 120.7, 118.6, 34.9, 31.4. HRMS (ESI): *m*/*z* calcd for C_24_H_23_N_2_O [M + H]^+^ 355.1805. Found 355.1805.

#### (2-(4-(Methylthio)phenyl)-2*H*-indazol-3-yl)(phenyl)methanone (**3ta**)

The product **3ta** was prepared by the general procedure with **1t** (48.1 mg, 0.2 mmol). 23.9 mg (35%); white solid; ^1^H NMR (400 MHz, CDCl_3_): δ 7.90–7.87 (m, 3H), 7.63 (t, *J* = 7.5 Hz, 1H), 7.52–7.44 (m, 4H), 7.40–7.36 (m, 1H), 7.32 (d, *J* = 8.5 Hz, 1H), 7.29–7.27 (m, 2H), 7.19–7.15 (m, 1H), 2.49 (s, 3H); ^13^C {^1^H} NMR (100 MHz, CDCl_3_): δ 186.0, 148.5, 140.2, 137.8, 137.5, 133.7, 132.1, 130.0, 128.7, 127.1, 126.6, 125.8, 125.1, 124.1, 120.6, 118.5, 15.7. HRMS (ESI): *m*/*z* calcd for C_21_H_17_N_2_OS [M + H]^+^ 345.1056. Found 345.1057.

#### Ethyl 4-(3-Benzoyl-2*H*-indazol-2-yl)benzoate (**3ua**)

The product **3ua** was prepared by the general procedure with **1u** (53.3 mg, 0.2 mmol). 57.8 mg (78%); white solid; ^1^H NMR (400 MHz, CDCl_3_): δ 8.14–8.11 (m, 2H), 7.92–7.87 (m, 3H), 7.66–7.61 (m, 3H), 7.49 (t, *J* = 7.9 Hz, 2H), 7.41–7.37 (m, 1H), 7.34 (d, *J* = 8.6 Hz, 1H), 7.20–7.16 (m, 1H), 4.39 (q, *J* = 7.1 Hz, 2H), 1.40 (t, *J* = 7.1 Hz, 3H); ^13^C {^1^H} NMR (100 MHz, CDCl_3_): δ 185.9, 165.7, 149.0, 143.9, 137.7, 134.0, 132.5, 130.8, 130.6, 130.1, 128.9, 127.5, 125.50, 125.45, 124.3, 120.7, 118.7, 61.4, 14.4. HRMS (ESI): *m*/*z* calcd for C_23_H_19_N_2_O_3_ [M + H]^+^ 371.1390. Found 371.1389.

#### Phenyl(2-(pyridin-2-yl)-2*H*-indazol-3-yl)methanone (**3va**)^18^

The product **3va** was prepared by the general procedure with **1v** (39.1 mg, 0.2 mmol). 27.7 mg (47%); white solid; ^1^H NMR (400 MHz, CDCl_3_): δ 8.22–8.20 (m, 1H), 8.05 (d, *J* = 8.2 Hz,1H), 7.89–7.83 (m, 4H), 7.57–7.52 (m, 1H), 7.49–7.47 (m, 1H), 7.44–7.36 (m, 3H), 7.23–7.19 (m, 1H), 7.18–7.14 (m, 1H); ^13^C {^1^H} NMR (100 MHz, CDCl_3_): 187.1, 151.8, 149.1, 148.0, 138.7, 138.0, 133.2, 132.5, 129.5, 128.6, 127.8, 124.7, 123.7, 123.3, 120.6, 118.4, 117.3.

#### (2-Cyclohexyl-2*H*-indazol-3-yl)(phenyl)methanone (**3wa**)^17^

The product **3wa** was prepared by the general procedure with **1w** (40.1 mg, 0.2 mmol). 14.1 mg (25%); white solid; ^1^H NMR (400 MHz, CDCl_3_): δ 7.90–7.85 (m, 3H), 7.67 (t, *J* = 7.5 Hz, 1H), 7.53 (t, *J* = 7.7 Hz, 2H), 7.30 (t, *J* = 7.4 Hz, 1H), 7.07 (t, *J* = 6.7 Hz, 1H), 7.02–7.00 (m, 1H), 5.23–5.15 (m, 1H), 2.25–2.15 (m, 4H), 2.00–1.95 (m, 2H), 1.81–1.77 (m, 1H), 1.58–1.47 (m, 2H), 1.44–1.36 (m, 1H); ^13^C {^1^H} NMR (100 MHz, CDCl_3_): 186.6, 147.4, 139.0, 133.3, 131.0, 129.9, 128.7, 125.8, 124.4, 123.5, 120.6, 118.4, 61.5, 33.9, 25.8, 25.4.

#### (2-(3,5-Dimethylphenyl)-2*H*-indazol-3-yl)(phenyl)methanone (**3xa**)^17^

The product **3xa** was prepared by the general procedure with **1x** (44.5 mg, 0.2 mmol). 41.7 mg (64%); white solid; ^1^H NMR (400 MHz, CDCl_3_): δ 7.92–7.86 (m, 3H), 7.64–7.60 (m, 1H), 7.50–7.45 (m, 2H), 7.42–7.38 (m, 2H), 7.22–7.18 (m, 1H), 7.16 (s, 2H), 7.03 (s, 1H), 2.33 (s, 6H); ^13^C {^1^H} NMR (100 MHz, CDCl_3_): δ 186.1, 148.4, 140.4, 139.0, 138.0, 133.5, 132.2, 130.7, 129.8, 128.6, 126.9, 125.0, 124.1, 123.3, 120.6, 118.5, 21.2.

#### (2-(3-Chloro-4-methylphenyl)-2*H*-indazol-3-yl)(phenyl)methanone (**3ya**)

The product **3ya** was prepared by the general procedure with **1y** (48.6 mg, 0.2 mmol). 51.9 mg (75%); yellow solid; ^1^H NMR (400 MHz, CDCl_3_): δ 7.88 (t, *J* = 7.3 Hz, 3H), 7.66–7.61 (m, 2H), 7.49 (t, *J* = 7.8 Hz, 2H), 7.38 (t, *J* = 8.8 Hz, 1H), 7.33–7.25 (m, 3H), 7.17 (t, *J* = 7.6 Hz, 1H), 2.40 (s, 3H); ^13^C {^1^H} NMR (100 MHz, CDCl_3_): δ 185.9, 148.7, 139.2, 137.9, 137.2, 134.8, 133.8, 132.3, 131.1, 130.0, 128.8, 127.3, 126.2, 125.3, 124.1, 123.8, 120.7, 118.7, 20.0. HRMS (ESI): *m*/*z* calcd for C_21_H_16_ClN_2_O [M + H]^+^ 347.0946. Found 347.0946.

#### (2-(3,4-Dichlorophenyl)-2*H*-indazol-3-yl)(phenyl)methanone (**3za**)

The product **3za** was prepared by the general procedure with **1z** (52.7 mg, 0.2 mmol). 55.3 mg (75%); yellow solid; ^1^H NMR (400 MHz, CDCl_3_): δ 7.91–7.85 (m, 3H), 7.74 (d, *J* = 2.5 Hz, 1H), 7.69–7.64 (m, 1H), 7.53–7.48 (m, 3H), 7.41–7.35 (m, 2H), 7.30 (d, *J* = 8.6 Hz, 1H), 7.20–7.16 (m, 1H); ^13^C {^1^H} NMR (100 MHz, CDCl_3_): δ 185.6, 148.8, 139.7, 137.6, 134.0, 133.3, 133.2, 132.3, 130.6, 130.0, 128.9, 127.54, 127.47, 125.6, 124.8, 124.1, 120.6, 118.6. HRMS (ESI): *m*/*z* calcd for C_20_H_13_Cl_2_N_2_O [M + H]^+^ 367.0399. Found 367.0403.

#### (4-Fluorophenyl)(2-phenyl-2*H*-indazol-3-yl)methanone (**3ab**)^19^

The product **3ab** was prepared by the general procedure with **2b** (168.2 mg, 1 mmol). 48.4 mg (76%); white solid; ^1^H NMR (400 MHz, CDCl_3_): δ 7.92–7.89 (m, 3H), 7.54–7.52 (m, 2H), 7.46–7.38 (m, 5H), 7.23–7.19 (m, 1H), 7.15–7.11 (m, 2H); ^13^C {^1^H} NMR (100 MHz, CDCl_3_): δ 184.6, 166.1 (d, *J* = 254.6 Hz), 148.7, 140.5, 134.2 (d, *J* = 2.9 Hz), 132.7 (d, *J* = 9.4 Hz), 132.1, 129.3, 129.2, 127.3, 125.6, 125.3, 124.1, 120.5, 118.8, 116.1 (d, *J* = 22.0 Hz).

#### (4-Chlorophenyl)(2-phenyl-2*H*-indazol-3-yl)methanone (**3ac**)^17^

The product **3ac** was prepared by the general procedure with **2c** (184.6 mg, 1 mmol). 46.2 mg (69%); white solid; ^1^H NMR (400 MHz, CDCl_3_): δ 7.89 (d, *J* = 8.8 Hz, 1H), 7.83–7.80 (m, 2H), 7.53–7.51 (m, 2H), 7.47–7.38 (m, 7H), 7.24–7.20 (m, 1H); ^13^C {^1^H} NMR (100 MHz, CDCl_3_): δ 184.8, 148.7, 140.5, 140.2, 136.2, 132.0, 131.4, 129.3, 129.24, 129.17, 127.3, 125.7, 125.5, 124.2, 120.5, 118.8.

#### (4-Bromophenyl)(2-phenyl-2*H*-indazol-3-yl)methanone (**3ad**)^17^

The product **3ad** was prepared by the general procedure with **2d** (229.1 mg, 1 mmol). 41.8 mg (56%); white solid; ^1^H NMR (400 MHz, CDCl_3_): δ 7.90 (d, *J* = 8.7 Hz, 1H), 7.74 (d, *J* = 8.4 Hz, 2H), 7.61 (d, *J* = 8.4 Hz, 2H), 7.53–7.50 (m, 2H), 7.47–7.37 (m, 5H), 7.22 (t, *J* = 6.6 Hz, 1H); ^13^C {^1^H} NMR (100 MHz, CDCl_3_): δ 184.9, 148.7, 140.5, 136.6, 132.1, 131.9, 131.5, 129.3, 129.2, 128.9, 127.3, 125.6, 125.5, 124.2, 120.5, 118.8.

#### (2-Fluorophenyl)(2-phenyl-2*H*-indazol-3-yl)methanone (**3ae**)^17^

The product **3ae** was prepared by the general procedure with **2e** (168.2 mg, 1 mmol). 42.9 mg (68%); white solid; ^1^H NMR (400 MHz, CDCl_3_): δ 7.90 (d, *J* = 8.8 Hz, 1H), 7.59 (td, *J*_*1*_ = 7.4 Hz, *J*_*2*_ = 1.8 Hz, 1H), 7.53–7.50 (m, 2H), 7.48–7.36 (m, 6H), 7.26–7.23 (m, 1H), 7.20 (td, *J*_*1*_ = 7.5 Hz, *J*_*2*_ = 1.0 Hz, 1H), 7.00–6.95 (m, 1H); ^13^C {^1^H} NMR (100 MHz, CDCl_3_): δ 182.0, 160.3 (d, *J* = 252.6 Hz), 148.7, 140.3, 134.3 (d, *J* = 8.5 Hz), 133.0, 130.7 (d, *J* = 2.1 Hz), 129.2, 129.0, 127.6 (d, *J* = 12.7 Hz), 127.3, 126.1, 126.0, 124.6 (d, *J* = 3.6 Hz), 124.4, 120.4, 118.8, 116.4 (d, *J* = 21.4 Hz).

#### (2-Phenyl-2*H*-indazol-3-yl)(2-(trifluoromethyl)phenyl)methanone (**3af**)^17^

The product **3af** was prepared by the general procedure with **2f** (218.2 mg, 1 mmol). 37.9 mg (52%); white solid; ^1^H NMR (400 MHz, CDCl_3_): δ 7.89 (d, *J* = 8.7 Hz, 1H), 7.79 (d, *J* = 6.6 Hz, 1H), 7.65–7.52 (m, 4H), 7.46–7.44 (m, 4H), 7.40–7.36 (m, 1H), 7.19–7.15 (m, 1H), 6.85 (d, *J* = 8.7 Hz, 1H); ^13^C {^1^H} NMR (100 MHz, CDCl_3_): δ 183.3, 148.6, 140.7, 138.4, 131.9, 131.5, 130.8, 129.4, 129.0 (2C), 128.8, 128.1 (q, *J* = 32.0 Hz), 127.1 (q, *J* = 4.1 Hz), 126.3, 126.1, 125.0, 123.5 (q, *J* = 272.0 Hz), 120.2, 119.0.

#### (2-Phenyl-2*H*-indazol-3-yl)(*p*-tolyl)methanone (**3ag**)^17^

The product **3ag** was prepared by the general procedure with **2g** (164.2 mg, 1 mmol). 36.7 mg (59%); white solid; ^1^H NMR (400 MHz, CDCl_3_): δ 7.91 (d, *J* = 8.9 Hz, 1H), 7.83 (d, *J* = 8.1 Hz, 2H), 7.59–7.56 (m, 2H), 7.48–7.39 (m, 5H), 7.29 (d, *J* = 8.0 Hz, 2H), 7.19 (t, *J* = 8.6 Hz, 1H), 2.47 (s, 3H); ^13^C {^1^H} NMR (100 MHz, CDCl_3_): δ 185.8, 148.6, 144.9, 140.6, 135.3, 132.6, 130.3, 129.5, 129.2, 129.0, 127.1, 125.6, 124.9, 124.0, 120.8, 118.6, 21.9.

#### (2-Phenyl-2*H*-indazol-3-yl)(*m*-tolyl)methanone (**3ah**)^17^

The product **3ah** was prepared by the general procedure with **2h** (164.2 mg, 1 mmol). 36.0 mg (58%); white solid; ^1^H NMR (400 MHz, CDCl_3_): δ 7.91–7.88 (m, 1H), 7.69–7.67 (m, 2H), 7.56–7.53 (m, 2H), 7.45–7.33 (m, 7H), 7.20–7.17 (m, 1H), 2.37 (s, 3H); ^13^C {^1^H} NMR (100 MHz, CDCl_3_): δ 186.3, 148.7, 140.6, 138.7, 137.9, 134.5, 132.6, 130.5, 129.2, 129.0, 128.6, 127.4, 127.1, 125.6, 125.1, 124.2, 120.8, 118.6, 21.4.

#### (3-Methoxyphenyl)(2-phenyl-2*H*-indazol-3-yl)methanone (**3ai**)^17^

The product **3ai** was prepared by the general procedure with **2i** (180.2 mg, 1 mmol). 26.1 mg (40%); white solid; ^1^H NMR (400 MHz, CDCl_3_): δ 7.89 (d, *J* = 8.6 Hz, 1H), 7.56–7.54 (m, 2H), 7.47–7.34 (m, 8H), 7.21–7.14 (m, 2H), 3.82 (s, 3H); ^13^C {^1^H} NMR (100 MHz, CDCl_3_): δ 185.8, 159.9, 148.7, 140.6, 139.2, 132.4, 129.8, 129.2, 129.1, 127.2, 125.6, 125.2, 124.2, 123.0, 120.8, 120.6, 118.6, 113.7, 55.6.

#### Benzo[*d*][1,3]dioxol-5-yl(2-phenyl-2*H*-indazol-3-yl)methanone (**3aj**)^17^

The product **3aj** was prepared by the general procedure with **2j** (194.2 mg, 1 mmol). 33.7 mg (50%); white solid; ^1^H NMR (400 MHz, CDCl_3_): δ 7.87 (d, *J* = 8.7 Hz, 1H), 7.56–7.52 (m, 2H), 7.50–7.47 (m, 1H), 7.46–7.36 (m, 6H), 7.19 (t, *J* = 7.8 Hz, 1H), 6.83 (d, *J* = 8.2 Hz, 1H), 6.07 (s, 2H); ^13^C {^1^H} NMR (100 MHz, CDCl_3_): δ 184.4, 152.6, 148.5, 148.4, 140.5, 132.4, 132.3, 129.2, 128.9, 127.4, 127.0, 125.4, 124.8, 123.8, 120.6, 118.5, 109.3, 108.1, 102.1.

#### Naphthalen-2-yl(2-phenyl-2*H*-indazol-3-yl)methanone (**3ak**)^17^

The product **3ak** was prepared by the general procedure with **2k** (200.2 mg, 1 mmol). 43.1 mg (62%); yellow solid; ^1^H NMR (400 MHz, CDCl_3_): δ 8.41 (s, 1H), 8.01–7.99 (m, 1H), 7.95–7.87 (m, 4H), 7.66–7.54 (m, 4H), 7.45–7.36 (m, 5H), 7.18–7.14 (m, 1H); ^13^C {^1^H} NMR (100 MHz, CDCl_3_): δ 185.9, 148.6, 140.6, 135.9, 135.1, 132.52, 132.48, 132.4, 129.7, 129.2, 129.0, 128.9, 128.8, 127.9, 127.11, 127.08, 125.5, 125.1, 125.0, 124.1, 120.6, 118.6.

### General Procedure for the synthesis of 2*H*-indazoles^31^

Aryl aldehyde (52.5 mmol), aniline (1.2 equiv., 63 mmol), sodium azide (2 equiv., 6.83 g, 105 mmol), cuprous iodide (10 mol %, 1.00 g, 5.25 mmol), tetramethylethylenediamine (10 mol %, 0.61 g, 5.25 mmol), and DMSO (120 mL) were added to a clean round-bottom flask. Then, the reaction mixture was heated to 120 °C (the heat source is an oil bath) for 24 h. After cooling to room temperature, diatomaceous earth was used for the filtration of the reaction mixture. The filter cake was washed with ethyl acetate, and the organic phase was combined and extracted with saturated saline solution. Then, the upper organic phase was collected, dried with anhydrous NaSO_4_, and filtered. Then, the organic phase was concentrated, followed by flash chromatography using EA and PE as eluents. The obtained product from column chromatography were collected and further recrystallized using PE/EA. Then, the solid were filtered, washed with PE, and dried under high-vacuum to obtain 2*H*-indazoles.

#### 2-Phenyl-2*H*-indazole (**1a**)^31^

**1a** was prepared by the general procedure. White solid; ^1^H NMR (600 MHz, CDCl_3_): δ 8.39 (s, 1H), 7.91–7.89 (m, 2H), 7.83–7.81 (m, 1H), 7.71 (d, *J* = 8.5 Hz, 1H), 7.53–7.50 (m, 2H), 7.41–7.38 (m, 1H), 7.35–7.32 (m, 1H), 7.14–7.11 (m, 1H); ^13^C {^1^H} NMR (150 MHz, CDCl_3_): δ 149.9, 140.6, 129.7, 128.0, 126.9, 122.9, 122.6, 121.1, 120.5 (2C), 118.0.

#### 5-Fluoro-2-phenyl-2*H*-indazole (**1b**)^17^

**1b** was prepared by the general procedure with 2-bromo-5-fluorobenzaldehyde. White solid; ^1^H NMR (500 MHz, CDCl_3_): δ 8.34 (s, 1H), 7.88–7.86 (m, 2H), 7.78–7.75 (m, 1H), 7.53–7.50 (m, 2H), 7.42–7.38 (m, 1H), 7.27–7.25 (m, 1H), 7.15–7.11 (m, 1H); ^13^C {^1^H} NMR (125 MHz, CDCl_3_): δ 158.83 (d, *J* = 241.9 Hz), 147.3, 140.5, 129.7, 128.2, 122.2 (d, *J* = 11.3 Hz), 121.0, 120.6 (d, *J* = 8.8 Hz), 120.2 (d, *J* = 10.1 Hz), 118.6 (d, *J* = 29.0 Hz), 102.8 (d, *J* = 25.2 Hz).

#### 5-Chloro-2-phenyl-2*H*-indazole (**1c**)

**1c** was prepared by the general procedure with 2-bromo-5-chlorobenzaldehyde. White solid; ^1^H NMR (500 MHz, CDCl_3_): δ 8.32 (s, 1H), 7.88–7.87 (m, 2H), 7.74 (d, *J* = 9.2 Hz, 1H), 7.66 (d, *J* = 1.3 Hz, 1H), 7.54–7.52 (m, 2H), 7.43–7.40 (m, 1H), 7.27–7.25 (m, 1H); ^13^C {^1^H} NMR (125 MHz, CDCl_3_): δ 148.0, 140.2, 129.6, 128.19, 128.16, 128.1, 123.1, 120.9, 119.9, 119.5, 119.0. HRMS (ESI): *m*/*z* calcd for C_13_H_10_ClN_2_ [M + H]^+^ 229.0527. Found 229.0518.

#### 5-Bromo-2-phenyl-2*H*-indazole (**1d**)^32^

**1d** was prepared by the general procedure with 2,5-dibromobenzaldehyde. White solid; ^1^H NMR (500 MHz, CDCl_3_): δ 8.33 (s, 1H), 7.88–7.86 (m, 3H), 7.67 (d, *J* = 9.2 Hz, 1H), 7.54–7.51 (m, 2H), 7.43–7.40 (m, 1H), 7.38–7.36 (m, 1H); ^13^C {^1^H} NMR (125 MHz, CDCl_3_): δ 148.3, 140.4, 130.7, 129.8, 128.4, 124.1, 122.7, 121.2, 120.0, 119.9, 116.1.

#### 5-Methoxy-2-phenyl-2*H*-indazole (**1e**)^32^

**1e** was prepared by the general procedure with 2-bromo-5-methoxybenzaldehyde. White solid; ^1^H NMR (500 MHz, CDCl_3_): δ 8.24 (s, 1H), 7.87–7.85 (m, 2H), 7.69 (d, *J* = 9.4 Hz, 1H), 7.51–7.48 (m, 2H), 7.38–7.35 (m, 1H), 7.05–7.03 (m, 1H), 6.88–6.87 (m, 1H), 3.84 (s, 3H); ^13^C {^1^H} NMR (125 MHz, CDCl_3_): δ 155.8, 147.0, 140.8, 129.7, 127.7, 123.0, 122.2, 120.8, 119.5, 119.4, 96.5, 55.5.

#### 6-Chloro-2-phenyl-2*H*-indazole (**1f**)^32^

The **1f** was prepared by the general procedure with 2-bromo-4-chlorobenzaldehyde. White solid; ^1^H NMR (500 MHz, CDCl_3_): δ 8.36 (s, 1H), 7.86 (d, *J* = 7.7 Hz, 2H), 7.78 (s, 1H), 7.62 (d, *J* = 8.9 Hz, 1H), 7.52 (t, *J* = 7.9 Hz, 2H), 7.40 (t, *J* = 7.4 Hz, 1H), 7.06–7.04 (m, 1H); ^13^C {^1^H} NMR (125 MHz, CDCl_3_): δ 150.0, 140.4, 132.9, 129.8, 128.4, 124.2, 121.9, 121.3, 121.1, 121.0, 117.1.

#### 6-Methyl-2-phenyl-2*H*-indazole (**1g**)^17^

The **1g** was prepared by the general procedure with 2-bromo-4-methylbenzaldehyde. White solid; ^1^H NMR (400 MHz, CDCl_3_): δ 8.33 (s, 1H), 7.88 (d, *J* = 7.3 Hz, 2H), 7.59 (d, *J* = 8.6 Hz, 1H), 7.55–7.49 (m, 3H), 7.40–7.36 (m, 1H), 6.97–6.95 (m, 1H), 2.47 (s, 3H); ^13^C {^1^H} NMR (100 MHz, CDCl_3_): δ 150.6, 140.7, 136.9, 129.6, 127.8, 125.6, 121.3, 121.0, 120.3, 120.0, 116.3, 22.4.

#### 2-Phenyl-2*H*-[1,3]dioxolo[4,5-*f*]indazole (**1h**)^17^

**1h** was prepared by the general procedure with 6-bromobenzo[*d*][1,3]dioxole-5-carbaldehyde. White solid; ^1^H NMR (400 MHz, CDCl_3_): δ 8.18 (s, 1H), 7.82–7.80 (m, 2H), 7.50–7.46 (m, 2H), 7.35–7.31 (m, 1H), 7.04 (s, 1H), 6.88 (m, 1H), 5.96 (s, 2H); ^13^C {^1^H} NMR (100 MHz, CDCl_3_): δ 149.9, 147.6, 146.3, 140.7, 129.7, 127.3, 120.3, 119.9, 118.8, 101.2, 95.1, 94.4.

#### 5-Fluoro-2-(*p*-tolyl)-2*H*-indazole (**1i**)^31^

**1i** was prepared by the general procedure. White solid; ^1^H NMR (500 MHz, CDCl_3_): δ 8.30 (s, 1H), 7.77–7.73 (m, 3H), 7.31–7.25 (m, 3H), 7.14–7.10 (m, 1H), 2.42 (s, 3H); ^13^C {^1^H} NMR (125 MHz, CDCl_3_): δ 158.8 (d, *J* = 240.7 Hz), 147.2, 138.3, 138.2, 130.2, 122.1 (d, *J* = 11.3 Hz), 120.9, 120.5 (d, *J* = 8.8 Hz), 120.1 (d, *J* = 8.8 Hz), 118.4 (d, *J* = 29.0 Hz), 102.8 (d, *J* = 25.2 Hz), 21.13.

#### 2-(4-Fluorophenyl)-2H-indazole (**1j**)^33^

**1j** was prepared by the general procedure with 4-fluoroaniline. White solid; ^1^H NMR (500 MHz, CDCl_3_): δ 8.32 (s, 1H), 7.86–7.84 (m, 2H), 7.79 (d, *J* = 8.8 Hz, 1H), 7.69 (d, *J* = 8.5 Hz, 1H), 7.34–7.31 (m, 1H), 7.22–7.19 (m, 2H), 7.14–7.11 (m, 1H); ^13^C {^1^H} NMR (125 MHz, CDCl_3_): δ 162.1 (d, *J* = 248.2 Hz), 149.9, 137.0, 127.1, 122.9, 122.8 (d, *J* = 8.8 Hz), 122.7, 120.6, 120.5, 118.0, 116.5 (d, *J* = 22.7 Hz).

#### 2-(3-Fluorophenyl)-2*H*-indazole (**1k**)^33^

**1k** was prepared by the general procedure with 3-fluoroaniline. White solid; ^1^H NMR (400 MHz, CDCl_3_): δ 8.39 (s, 1H), 7.79–7.77 (m, 1H), 7.72–7.67 (m, 3H), 7.51–7.45 (m, 1H), 7.35–7.31 (m, 1H), 7.14–7.07 (m, 2H); ^13^C {^1^H} NMR (100 MHz, CDCl_3_): δ 163.2 (d, *J* = 246.0 Hz), 149.9, 141.9 (d, *J* = 10.0 Hz), 130.9 (d, *J* = 9.0 Hz), 127.3, 122.9, 120.5, 118.0, 116.1 (d, *J* = 3.0 Hz), 114.8, 114.6, 108.8, 108.6.

#### 2-(4-Chlorophenyl)-2*H*-indazole (**1l**)^33^

**1l** was prepared by the general procedure with 4-chloroaniline. Yellow solid; ^1^H NMR (600 MHz, CDCl_3_): δ 8.35 (s, 1H), 7.85–7.83 (m, 2H), 7.78–7.77 (m, 1H), 7.68 (d, *J* = 8.5 Hz, 1H), 7.49–7.46 (m, 2H), 7.34–7.32 (m, 1H), 7.13–7.10 (m, 1H); ^13^C {^1^H} NMR (150 MHz, CDCl_3_): δ 150.0, 139.2, 133.7, 129.8, 127.2, 123.0, 122.9, 122.1, 120.5, 120.4, 118.0.

#### 2-(4-Bromophenyl)-2*H*-indazole (**1m**)^17^

**1m** was prepared by the general procedure with 4-bromoaniline. Yellow solid; ^1^H NMR (600 MHz, CDCl_3_): δ 8.35 (s, 1H), 7.79–7.76 (m, 3H), 7.69–7.67 (m, 1H), 7.64–7.62 (m, 2H), 7.34–7.31 (m, 1H), 7.13–7.10 (m, 1H), ^13^C {^1^H} NMR (150 MHz, CDCl_3_): δ 150.0, 139.6, 132.8, 127.3, 123.0, 122.9, 122.4, 121.6, 120.5, 120.3, 118.1.

#### 2-(4-(Trifluoromethyl)phenyl)-2*H*-indazole (**1n**)^31^

**1n** was prepared by the general procedure with 4-(trifluoromethyl)aniline. White solid; ^1^H NMR (400 MHz, CDCl_3_): δ 8.46 (s, 1H), 8.06–8.04 (m, 2H), 7.80–7.77 (m, 3H), 7.71–7.69 (m, 1H), 7.37–7.33 (m, 1H), 7.15–7.11 (m, 1H); ^13^C {^1^H} NMR (100 MHz, CDCl_3_): δ 150.3, 143.0, 129.9 (q, *J* = 33.0 Hz), 127.7, 127.0 (q, *J* = 3.0 Hz), 123.21, 123.15, 122.6, 120.9, 120.6, 120.6, 118.2.

#### 2-(*p*-tolyl)-2*H*-indazole (**1o**)^31^

**1o** was prepared by the general procedure with *p*-toluidine. White solid; ^1^H NMR (500 MHz, CDCl_3_): δ 8.35 (s, 1H), 7.82–7.77 (m, 3H), 7.70 (d, *J* = 8.4 Hz, 1H), 7.34–7.30 (m, 3H), 7.13–7.10 (m, 1H), 2.42 (s, 3H); ^13^C {^1^H} NMR (125 MHz, CDCl_3_): δ 149.6, 138.4, 138.0, 130.2, 126.8, 122.8, 122.4, 120.9, 120.43, 120.37, 118.0, 21.1.

#### 2-(*m*-tolyl)-2*H*-indazole (**1p**)^34^

**1p** was prepared by the general procedure with *m*-toluidine. White solid; ^1^H NMR (400 MHz, CDCl_3_): δ 8.40 (s, 1H), 7.81–7.76 (m, 2H), 7.72–7.70 (m, 1H), 7.67–7.65 (m, 1H), 7.40 (t, *J* = 7.8 Hz, 1H), 7.35–7.31 (m, 1H), 7.23–7.20 (m, 1H), 7.14–7.10 (m, 1H), 2.47 (s, 3H); ^13^C {^1^H} NMR (100 MHz, CDCl_3_): δ 149.7, 140.5, 139.8, 129.4, 128.7, 126.8, 122.7, 122.4, 121.8, 120.5, 120.4, 118.0, 117.9, 21.5.

#### 2-(*o*-Tolyl)-2*H*-indazole (**1q**)^34^

**1q** was prepared by the general procedure with *o*-toluidine. White solid; ^1^H NMR (500 MHz, CDCl_3_): δ 8.10 (s, 1H), 7.83–7.81 (m, 1H), 7.75 (d, *J* = 8.5 Hz, 1H), 7.45–7.32 (m, 5H), 7.17–7.14 (m, 1H), 2.26 (s, 3H); ^13^C {^1^H} NMR (125 MHz, CDCl_3_): δ 149.3, 140.4, 133.9, 131.3, 129.2, 126.62, 126.59, 126.4, 124.3, 122.2, 122.0, 120.3, 118.0, 17.9.

#### 2-(4-Methoxyphenyl)-2*H*-indazole (**1r**)^17^

**1r** was prepared by the general procedure with 4-methoxyaniline. Yellow solid; ^1^H NMR (500 MHz, CDCl_3_): δ 8.29 (s, 1H), 7.79 (d, *J* = 8.7 Hz, 3H), 7.69 (d, *J* = 8.5 Hz, 1H), 7.33–7.30 (m, 1H), 7.10 (t, *J* = 7.5 Hz, 1H), 7.02–7.00 (m, 2H), 3.85 (s, 3H); ^13^C {^1^H} NMR (125 MHz, CDCl_3_): δ 159.4, 149.7, 134.2, 126.6, 122.8, 122.5, 122.3, 120.41, 120.37, 117.9, 114.8, 55.7.

#### 2-(4-(*tert*-Butyl)phenyl)-2*H*-indazole (**1s**)^35^

**1s** was prepared by the general procedure with 4-(*tert*-butyl)aniline. White solid; ^1^H NMR (400 MHz, CDCl_3_): δ 8.38 (s, 1H), 7.84–7.79 (m, 3H), 7.72–7.70 (m, 1H), 7.55–7.52 (m, 2H), 7.34–7.31 (m, 1H), 7.14–7.10 (m, 1H), 1.38 (s, 9H); ^13^C {^1^H} NMR (100 MHz, CDCl_3_): δ 151.3, 149.8, 138.2, 126.8, 126.6, 122.8, 122.4, 120.8, 120.5, 120.4, 118.0, 34.8, 31.4.

#### 2-(4-(Methylthio)phenyl)-2*H*-indazole (**1t**)^31^

**1t** was prepared by the general procedure with 4-(methylthio)aniline. White solid; ^1^H NMR (400 MHz, CDCl_3_): δ 8.36 (s, 1H), 7.84–7.77 (m, 3H), 7.70–7.68 (m, 1H), 7.39–7.35 (m, 2H), 7.34–7.30 (m, 1H), 7.13–7.09 (m, 1H), 2.53 (s, 3H); ^13^C {^1^H} NMR (100 MHz, CDCl_3_): δ 149.7, 138.7, 137.8, 127.3, 126.9, 122.8, 122.5, 121.3, 120.4, 120.2, 117.9, 15.9.

#### Ethyl 4-(2*H*-Indazol-2-yl)benzoate (**1u**)^31^

**1u** was prepared by the general procedure with ethyl 4-aminobenzoate. White solid; ^1^H NMR (500 MHz, CDCl_3_): δ 8.45 (s, 1H), 8.20–8.18 (m, 2H), 8.00–7.98 (m, 2H), 7.78–7.76 (m, 1H), 7.68 (d, *J* = 8.5 Hz, 1H), 7.34–7.31 (m, 1H), 7.13–7.10 (m, 1H), 4.41 (q, *J* = 7.1 Hz, 2H), 1.42 (t, *J* = 7.1 Hz, 3H); ^13^C {^1^H} NMR (125 MHz, CDCl_3_): δ 165.9, 150.4, 143.8, 131.3, 129.8, 127.6, 123.21, 123.15, 120.7, 120.4, 118.3, 61.5, 14.5.

#### 2-(Pyridin-2-yl)-2*H*-indazole (**1v**)^31^

**1v** was prepared by the general procedure with pyridin-2-amine. White solid; ^1^H NMR (500 MHz, CDCl_3_): δ 9.10 (s, 1H), 8.49 (d, *J* = 4.6 Hz, 1H), 8.27 (d, *J* = 8.2 Hz, 1H), 7.86 (t, *J* = 7.8 Hz, 1H), 7.77–7.71 (m, 2H), 7.32 (t, *J* = 7.7 Hz, 1H), 7.26 (t, *J* = 6.1 Hz, 1H), 7.09 (t, *J* = 7.5 Hz, 1H); ^13^C {^1^H} NMR (125 MHz, CDCl_3_): δ 152.1, 150.5, 148.5, 139.0, 127.7, 122.91, 122.86, 122.6, 121.4, 120.8, 118.2, 114.3.

#### 2-Cyclohexyl-2*H*-indazole (**1w**)^17^

**1w** was prepared by the general procedure with cyclohexanamine. White solid; ^1^H NMR (400 MHz, CDCl_3_): δ 7.94 (s, 1H), 7.73–7.71 (m, 1H), 7.66–7.64 (m, 1H), 7.29–7.25 (m, 1H), 7.08–7.04 (m, 1H), 4.43–4.36 (m, 1H), 2.29–2.24 (m, 2H), 1.98–1.88 (m, 4H), 1.80–1.76 (m, 1H), 1.54–1.42 (m, 2H), 1.37–1.26 (m, 1H); ^13^C {^1^H} NMR (100 MHz, CDCl_3_): δ 148.2, 125.6, 121.41, 121.38, 120.2, 120.1, 117.4, 62.9, 34.0, 25.5, 25.4.

#### 2-(3,5-Dimethylphenyl)-2*H*-indazole (**1x**)^17^

**1x** was prepared by the general procedure with 3,5-dimethylaniline. White solid; ^1^H NMR (400 MHz, CDCl_3_): δ 8.38 (s, 1H), 7.82–7.79 (m, 1H), 7.71–7.69 (m, 1H), 7.52 (s, 2H), 7.34–7.30 (m, 1H), 7.13–7.09 (m, 1H), 7.03 (m, 1H), 2.42 (s, 6H); ^13^C {^1^H} NMR (100 MHz, CDCl_3_): δ 149.7, 140.5, 139.6, 129.7, 126.8, 122.7, 122.4, 120.6, 120.5, 118.9, 118.0, 21.5.

#### 2-(3-Chloro-4-methylphenyl)-2*H*-indazole (**1y**)^36^

**1y** was prepared by the general procedure with 3-chloro-4-methylaniline. White solid; ^1^H NMR (500 MHz, CDCl_3_): δ 8.35 (s, 1H), 7.94 (d, *J* = 2.1 Hz, 1H), 7.77 (d, *J* = 8.8 Hz, 1H), 7.69–7.67 (m, 2H), 7.36–7.31 (m, 2H), 7.11 (t, *J* = 7.5 Hz, 1H), 2.43 (s, 3H); ^13^C {^1^H} NMR (125 MHz, CDCl_3_): δ 145.0, 139.5, 136.0, 135.5, 131.8, 127.2, 123.0, 122.8, 121.7, 120.6, 120.5, 119.0, 118.1, 19.9.

#### 2-(3,4-Dichlorophenyl)-2*H*-indazole (**1z**)^37^

**1z** was prepared by the general procedure with 3,4-dichloroaniline. White solid; ^1^H NMR (500 MHz, CDCl_3_): δ 8.34 (s, 1H), 8.06 (d, *J* = 2.6 Hz, 1H), 7.76–7.72 (m, 2H), 7.67 (d, *J* = 8.5 Hz, 1H), 7.56 (d, *J* = 8.7 Hz, 1H), 7.35–7.32 (m, 1H), 7.14–7.10 (m, 1H); ^13^C {^1^H} NMR (125 MHz, CDCl_3_): δ 150.1, 139.6, 133.7, 131.8, 131.2, 127.5, 123.04, 122.98, 122.6, 120.4, 120.3, 119.6, 118.0.

### General Procedure for the Synthesis of α-Keto Acids^38^

Aryl ketones (32 mmol) and SeO_2_ (2 equiv., 7.10 g, 64 mmol) were added to a round-bottom flask under a nitrogen atmosphere. Then, degassed pyridine (30 mL) was added as the solvent. The reaction mixture was heated to 120 °C using an oil bath for 15 h. When reaction was finished, the reaction mixture was filtered and washed with EA, and the filtrate was collected and evaporated using a rotavapor. Saturated aqueous NaOH solution was added to this concentrated reaction mixture to adjust the pH to 12. Then, the aqueous phase was collected and extracted with EA and saturated NaCl solution. After the aqueous phase was collected and adjusted to pH < 2 using 2 M hydrochloric acid, the aqueous phase was extracted with EA. Then, the organic layer was collected, dried with anhydrous NaSO_4_, and filtered before it was concentrated and purified by flash chromatography (EA and PE).

#### 2-(4-Fluorophenyl)-2-oxoacetic Acid (**2b**)^39^

**2b** was prepared by the general procedure with 1-(4-fluorophenyl)ethan-1-one. White solid; ^1^H NMR (400 MHz, CDCl_3_): δ 8.43–8.39 (m, 2H), 7.24–7.18 (m, 2H); ^13^C {^1^H} NMR (100 MHz, CDCl_3_): δ 182.7, 167.4 (d, *J* = 258.5 Hz), 161.9, 134.4 (d, *J* = 9.0 Hz), 128.2 (d, *J* = 2.9 Hz), 116.5 (d, *J* = 22.0 Hz).

#### 2-(4-Chlorophenyl)-2-oxoacetic acid (**2c**)^38^

**2c** was prepared by the general procedure with 1-(4-chlorophenyl)ethan-1-one. White solid; ^1^H NMR (400 MHz, CDCl_3_): δ 8.29–8.26 (m, 2H), 7.53–7.50 (m, 2H); ^13^C {^1^H} NMR (100 MHz, CDCl_3_): δ 183.4, 162.2, 142.8, 132.7, 130.2, 129.6.

#### 2-(4-Bromophenyl)-2-oxoacetic Acid (**2d**)^39^

**2d** was prepared by the general procedure with 1-(4-bromophenyl)ethan-1-one. White solid; ^1^H NMR (400 MHz, CDCl_3_): δ 8.22–8.19 (m, 2H), 7.70–7.67 (m, 2H); ^13^C {^1^H} NMR (100 MHz, CDCl_3_): δ 183.6, 162.0, 132.7, 132.6, 131.8, 130.6.

#### 2-(2-Fluorophenyl)-2-oxoacetic Acid (**2e**)^17^

**2e** was prepared by the general procedure with 1-(2-fluorophenyl)ethan-1-one. Brown solid; ^1^H NMR (400 MHz, CDCl_3_): δ 8.00–7.96 (m, 1H), 7.70–7.64 (m, 1H), 7.34–7.30 (m, 1H), 7.22–7.17 (m, 1H); ^13^C {^1^H} NMR (100 MHz, CDCl_3_): δ 183.6, 166.4, 162.9 (d, *J* = 258.0 Hz), 137.2 (d, *J* = 9.0 Hz), 131.4, 125.0 (d, *J* = 3.0 Hz), 121.3 (d, *J* = 10.0 Hz), 116.9 (d, *J* = 21.0 Hz).

#### 2-Oxo-2-(2-(trifluoromethyl)phenyl)acetic Acid (**2f**)^17^

**2f** was prepared by the general procedure with 1-(2-(trifluoromethyl)phenyl)ethan-1-one. Brown liquid; ^1^H NMR (400 MHz, CDCl_3_): δ 7.78–7.75 (m, 1H), 7.68–7.67 (m, 3H); ^13^C {^1^H} NMR (100 MHz, CDCl_3_): δ 187.6, 162.2, 132.5, 132.1, 130.1, 129.0 (q, *J* = 33.0 Hz), 127.2 (q, *J* = 5.0 Hz), 123.6 (q, *J* = 272.0 Hz).

#### 2-Oxo-2-(*p*-tolyl)acetic Acid (**2g**)^39^

The **2g** was prepared by the general procedure with 1-(*p*-tolyl)ethan-1-one. Brown solid; ^1^H NMR (400 MHz, CDCl_3_): δ 8.21 (d, *J* = 8.0 Hz, 2H), 7.32 (d, *J* = 8.0 Hz, 2H), 2.45 (s, 3H); ^13^C {^1^H} NMR (100 MHz, CDCl_3_): δ 184.2, 162.9, 147.4, 131.5, 129.9, 129.4, 22.2.

#### 2-Oxo-2-(*m*-tolyl)acetic Acid (**2h**)^17^

**2h** was prepared by the general procedure with 1-(*m*-tolyl)ethan-1-one. Brown liquid; ^1^H NMR (400 MHz, CDCl_3_): δ 7.98–7.91 (m, 2H), 7.48–7.36 (m, 2H), 2.40 (s, 3H); ^13^C {^1^H} NMR (100 MHz, CDCl_3_): δ 186.1, 165.0, 139.0, 136.4, 132.0, 131.1, 129.0, 128.2, 21.3.

#### 2-(3-Methoxyphenyl)-2-oxoacetic Acid (**2i**)^17^

The **2i** was prepared by the general procedure with 1-(3-methoxyphenyl)ethan-1-one. Yellow solid; ^1^H NMR (400 MHz, CDCl_3_): δ 7.85 (d, *J* = 7.8 Hz, 2H), 7.43 (t, *J* = 7.9 Hz, 1H), 7.26–7.22 (m, 1H), 3.87 (s, 3H); ^13^C {^1^H} NMR (100 MHz, CDCl_3_): δ 185.1, 163.5, 160.0, 133.1, 130.2, 124.2, 122.7, 114.3, 55.7.

#### 2-(Benzo[d][1,3]dioxol-5-yl)-2-oxoacetic Acid (**2j**)^39^

**2j** was prepared by the general procedure with 1-(benzo[*d*][1,3]dioxol-5-yl)ethan-1-one. White solid; ^1^H NMR (400 MHz, (CD_3_)_2_SO): δ 7.54–7.51 (m, 1H), 7.38 (d, *J* = 1.7 Hz, 1H), 7.12 (d, *J* = 8.2 Hz, 1H), 6.20 (s, 2H); ^13^C {^1^H} NMR (100 MHz, (CD_3_)_2_SO): δ 186.9, 166.3, 153.4, 148.5, 127.7, 126.4, 108.7, 107.3, 102.7.

#### 2-(Naphthalen-2-yl)-2-oxoacetic Acid (**2k**)^39^

**2k** was prepared by the general procedure with 1-(naphthalen-2-yl)ethan-1-one. Yellow solid; ^1^H NMR (400 MHz, CDCl_3_): δ 9.10 (s, 1H), 8.20–8.17 (m, 1H), 8.03 (d, *J* = 8.2 Hz, 1H), 7.94–7.88 (m, 2H), 7.70–7.66 (m, 1H), 7.62–7.58 (m, 1H); ^13^C {^1^H} NMR (100 MHz, CDCl_3_): δ 184.4, 162.4, 136.7, 135.7, 132.4, 130.6, 130.3, 129.1, 128.03, 127.99, 127.4, 124.8.

## Data Availability

The data underlying this study are available in the published article and its Supporting Information

## Supporting Information Available

Investigation of the large-scale reaction, product inhibition effect, UV/vis spectra, luminescence quenching, headspace gas analysis, and ^1^H/^13^C {^1^H} NMR spectra (PDF)
