# Supplementary material for: Visible-Light-Driven Decarboxylative Coupling of 2H-Indazoles with α-Keto Acids without Photocatalysts and Oxidants
Source: J Org Chem. 2024 Apr 20;89(9):6159–68. doi: 10.1021/acs.joc.4c00176 (PMC11077484; doi:10.1021/acs.joc.4c00176)
Supplement: Supplementary file 1 — jo4c00176_si_001.pdf [file jo4c00176_si_001.pdf]

Supporting Information

Visible-Light-Driven Decarboxylative Coupling of 2*H*-Indazoles with *α*-Keto Acids without Photocatalysts and Oxidants

Mengyu Niu,[a] Chen Yang,[a] Mingzhu Leng,[a] Qun Cao,[*b] Meichao Li[*a] and Zhenlu Shen[*a]

[a] College of Chemical Engineering, Zhejiang University of Technology, Hangzhou, 310014,China.

[b] School of Chemistry, University of Leicester, Leicester, LE1 7RH.

Email: limc@zjut.edu.cn

Email: zhenlushen@zjut.edu.cn

Email: qc52@le.ac.uk

1. **General Information**……………………………………………………………………….........S3

2. **Experimental Details**..………........S4

Figure S1……………………………………………………………………………...…...….S6

Figure S2………………………………………………………………………………........…S7

Figure S3………………………………………………………………………………........…S8

Figure S4………………………………………………………………………………........…S8

Figure S5……………………………………………………….....……..………….........…S9

Figure S6……………………………………………………….....……..………….......….S10

Figure S7……………………………………………………….....……..………….......….S12

3. **NMR data**………………….……………………………...…………………….................S13

1 General Information

Unless stated otherwise, all reagents were purchased from commercial sources and used without further purification. Thin layer chromatography (TLC) was carried out using Merck TLC silica gel 60 sheet and visualized with ultraviolet light (254/365 nm). Flash column chromatography (FCC) was performed on silica gel (200-300 mesh) as the stationary phase and the solvents employed were of analytical grade. The air, oxygen, nitrogen and helium cylinder were supplied by Hangzhou Jingong Special Gases.

^{1}H NMR spectra and ^{13}C NMR spectra were recorded on a Bruker AVANCE NEO (400 MHz/ 500 MHz/ 600 MHz) spectrometer and Bruker AVANCE NEO (100 MHz/ 125 MHz/ 150Hz) spectrometer at 25 ^{o}C, respectively.

Gas chromatography analysis was carried out using Shimadzu GC2010 Plus with AT•SE-54 GC capillary column (30 m × 250 μm × 0.33 μm) was employed for all the separations using the following conditions: initial column temperature 80 °C; initial hold time 2 min; final temperature 280 °C; hold time 10 min; temperature ramp 15 °C/min; detector temperature: 300 °C, injection temperature 280 °C; injection volume 1 μL; split ratio 30:1; column flow rate 1 mL/min. The effluent was combusted in a H_2/Air flame and detected using an FID (flame ionization detector).

Gas chromatography-mass spectrometry (GC-MS) was carried out using Thermo Fisher Trace ISQ with TG-5MS GC capillary column (60 m × 250 μm × 0.25 μm) was employed for all the separations using the following conditions: the initial column temperature 80 °C, initial held time 2 min; final temperature was increased to 280 °C at 15 °C/min and held for 10 min. Injection temperature 250 °C, ion source temperature 200 °C, EI ionization method with electron energy 70 eV and mass-to-charge ratio: 40-500.

High resolution mass spectra (HRMS) were measured with an Agilent 6230 TOF instrument.

2 Experimental Details

Large-Scale Reaction

1) Large-Scale Reaction with 3 mmol of 1a

In order to explore the applicability of the reaction, we used 2-phenyl-2*H*-indazole (**1a**) and benzoylformic acid (**2a**) as examples to carry out gram-scale experiments on the reaction.

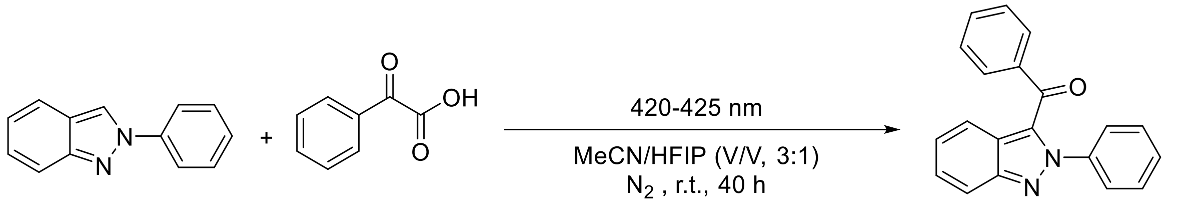

1a (0.58 g, 3 mmol) **2a** (5 equiv., 2.25 g, 15 mmol) **3aa** (0.57 g, 1.91 mmol), 64%

Experimental steps: 2-phenyl-2*H*-indazole (**1a**, 0.58 g, 3 mmol) and benzoylformic acid (**2a**, 5.0 equiv., 2.25 g, 15 mmol) were added to the dried 100 mL Schlenk flask. The Schlenk flask was evacuated and purged with nitrogen three times using a Schlenk line. Subsequently, 15 mL of degassed MeCN and 5 mL of degassed HFIP were added under nitrogen charging conditions, and the flask was tightly sealed. The reaction was conducted under 420-425 nm light for 40 hours. After completion of the reaction, the solution was concentrated, and flash chromatography was performed using petroleum ether (PE)/ethyl acetate (EA) = 100:1-15:1 as eluent. Finally, 0.57 g of the target product **3aa** was obtained, and the separation yield was 64%.

2) Large-Scale Reaction with 6 mmol of 1a

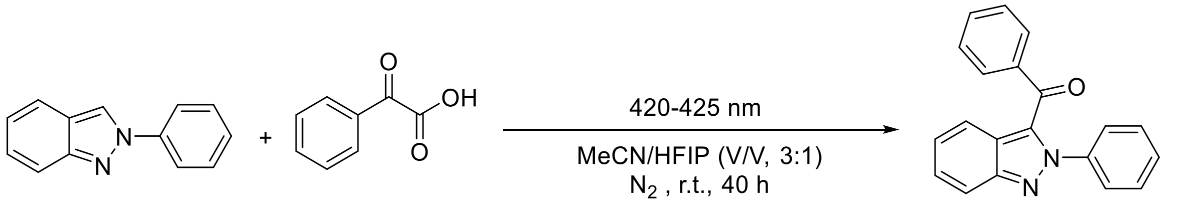

1a (1.17 g, 6 mmol) **2a** (5 equiv., 4.50 g, 30 mmol) **3aa** (0.99 g, 3.33 mmol), 56%

Experimental steps: 2-phenyl-2*H*-indazole (**1a**, 1.17 g, 6 mmol) and benzoylformic acid (**2a**, 5.0 equiv., 4.50 g, 30 mmol) were added to the dried 100 mL Schlenk flask. The Schlenk flask was evacuated and purged with nitrogen three times using a Schlenk line. Subsequently, 30 mL of de-

gassed MeCN and 10 mL of degassed HFIP were added under nitrogen charging conditions, and the flask was tightly sealed. The reaction was conducted under 420-425 nm light for 40 hours. After completion of the reaction, the solution was concentrated, and flash chromatography was performed using petroleum ether (PE)/ethyl acetate (EA) = 100:1-15:1 as eluent. Finally, 0.99 g of the target product **3aa** was obtained, and the separation yield was 56%.

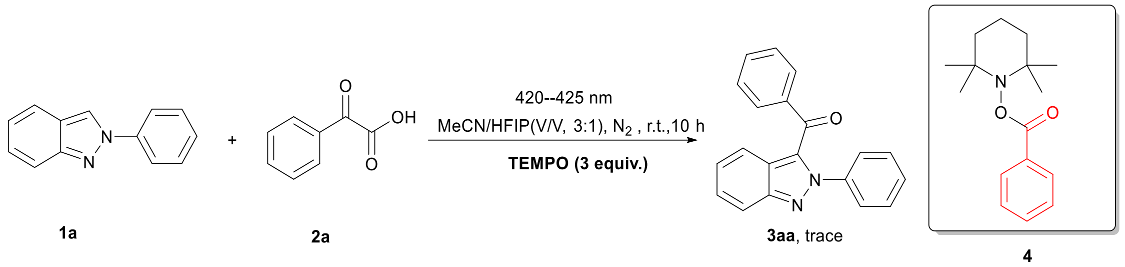

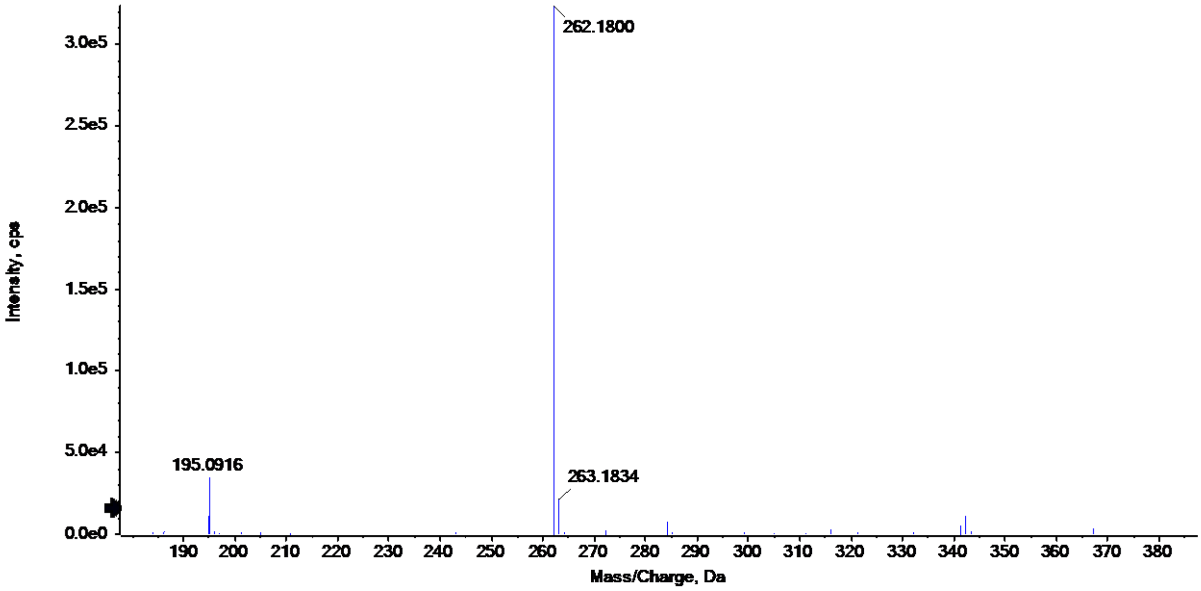

Figure S1. Confirmation of compound **4** by HRMS (ESI). Calcd. for $[M+H]^+$ $C_{16}H_{23}NO_2$: 262.1802, found: 262.1800

Product inhabitation experiments:

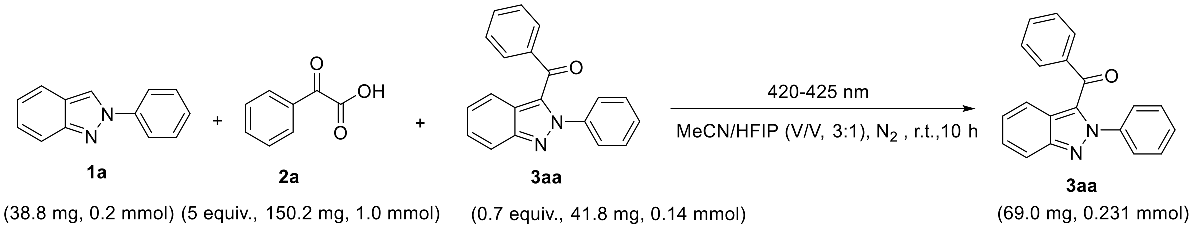

Figure S2. Study of product inhabitation effect.

Under the optimized reaction conditions, **3aa** (0.7 equiv., 41.8 mg, 0.14 mmol) along with **1a** (38.8 mg, 0.2 mmol) and **2a** (150.2 mg, 1.0 mmol) were introduced. Following a 10 h reaction period, 69.0 mg of **3aa** (0.231 mmol) was isolated. Subtracting the initial **3aa** quantity before the reaction, 0.0913 mmol of the **3aa** product was generated during the reaction. Consequently, the yield of **3aa** was 46%, significantly lower than the standard reaction (Figure 2 in the manuscript), suggesting the presence of a product inhibition effect in the reaction system.

UV/Vis Absorption Experiment

The UV/Vis absorption spectrum of **1a**, **2a**, **3aa, 1w,** and **3wa** in MeCN were recorded in 3 cm path quartz cuvettes by using a UV-2600 UV-visible spectrophotometer. The obtained UV-Vis absorption spectrum were shown in Figure S3 & S4.

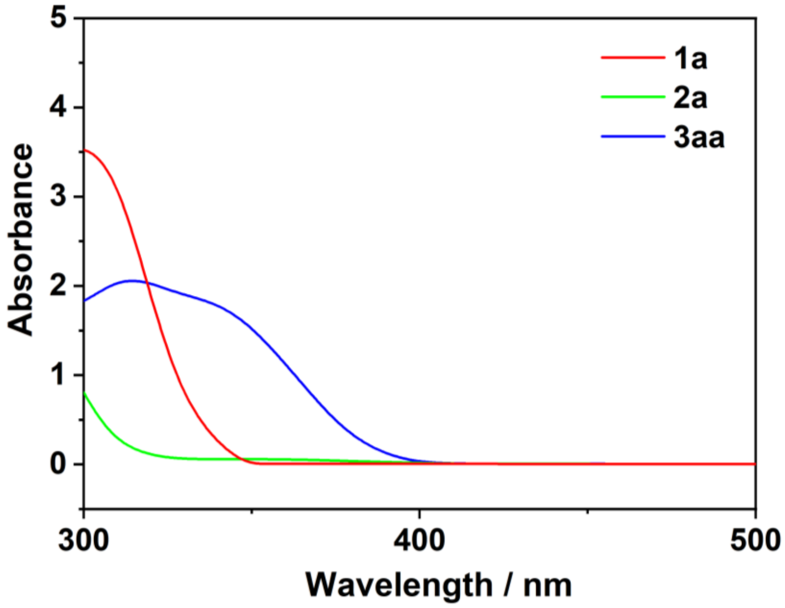

Figure S3 UV-Vis spectrum of **1a**, **2a** and **3aa** in acetonitrile.

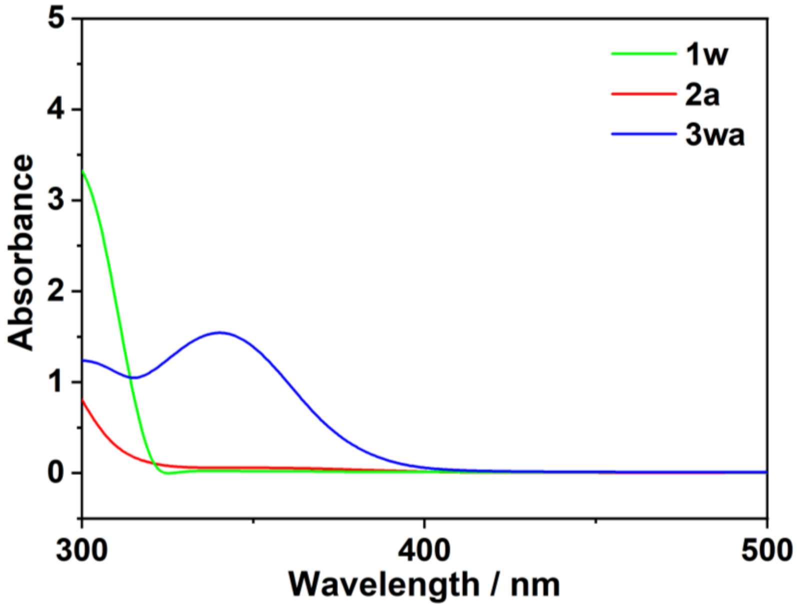

Figure S4 UV-Vis spectrum of on **1w**, **2a** and **3wa** in acetonitrile.

Luminescence Quenching Screening Studies

Solution of **1a** or **3aa** was irradiated at 370 nm approximately and the emission intensity from 380 nm to 650 nm was recorded by F-7000 FL Spectrophotometer. A 3 mL solution of **1a** or **3aa** in MeCN (0.01 mmol/mL) was added **2a** (0.1 equiv., 0.2 equiv., 0.4 equiv., 0.6 equiv., 0.8 equiv. in turn), and emission spectra of the sample were collected instantly after each addition.

For the fluorescence quenching experiment, we configured three groups of samples and made a line diagram with error bars in the manuscript (Figure 3). Each fluorescence quenching experiment was shown below:

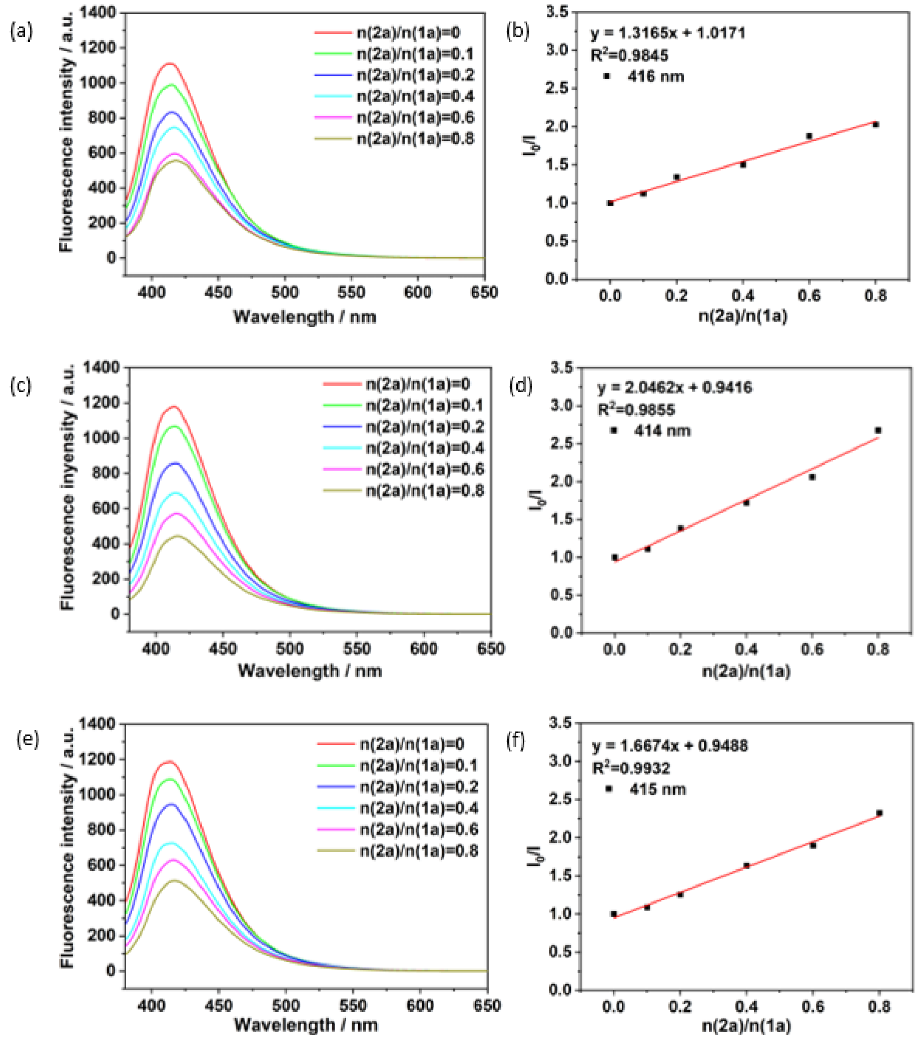

Figure S5. Luminescence Quenching Screening Studies using 2a. (a, b) The fluorescence quenching experiment No.1 and its corresponding Stern-Volmer plot. (c, d) The fluorescence quenching experiment No.2 and its corresponding Stern-Volmer plot. (e, f) The fluorescence quenching experiment No.3 and its corresponding Stern-Volmer plot.

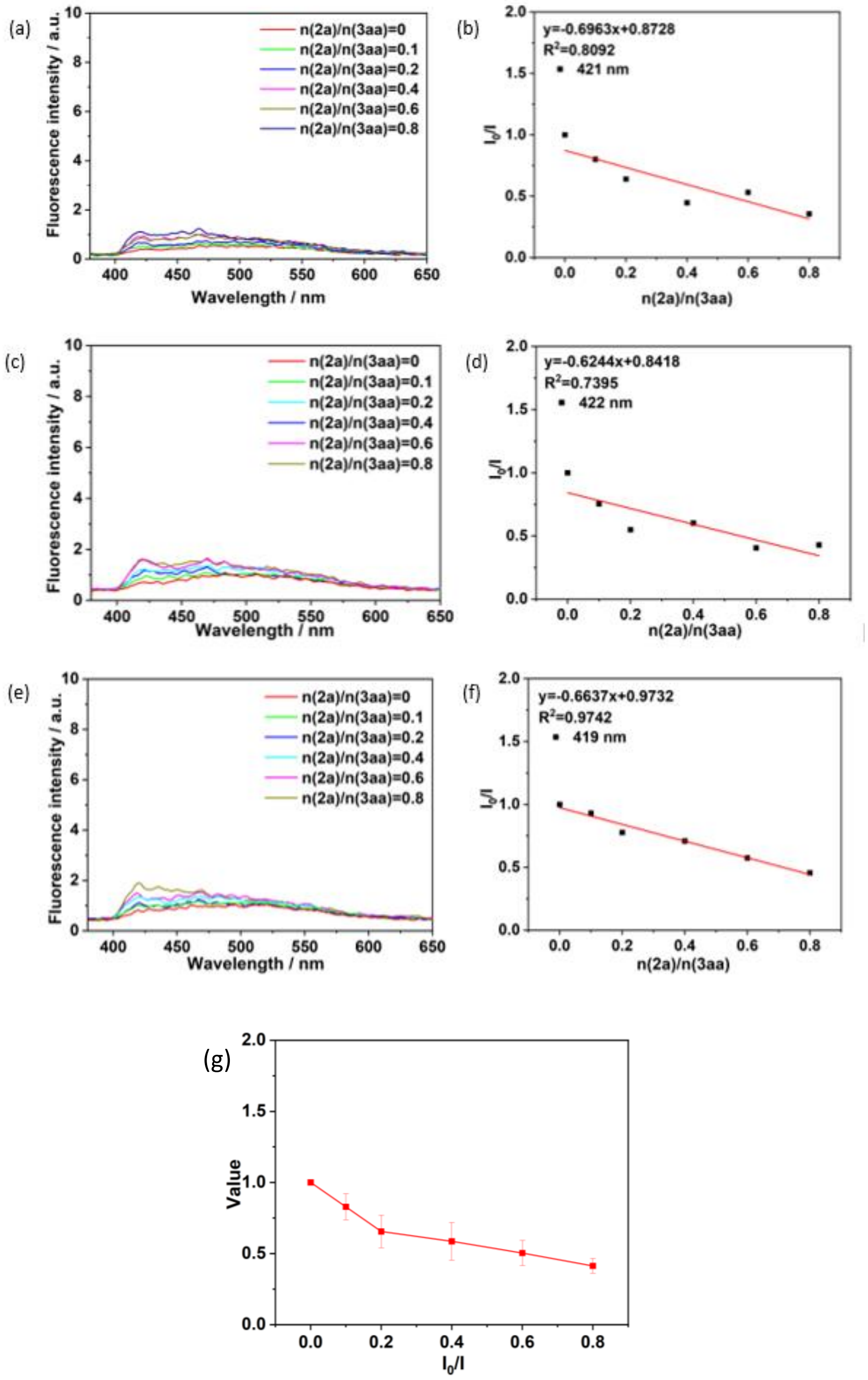

Figure S6. Luminescence Quenching Screening Studies using 3aa. (a, b) The fluorescence quenching experiment No.1 and its corresponding Stern-Volmer plot. (c, d) The fluorescence quenching experiment NO.2 and its corresponding Stern-Volmer plot. (e, f) The third group fluorescence quenching experiment NO.3 and its corresponding Stern-Volmer plot. (g) Stern-Volmer plot with error bars.

Headspace gas analysis utilizing GC-TCD.

Under standard conditions the transformation of **1a** and **2a** was carried out in closed Schlenk flask. After 10 h, the gas sample was collected by the closed quartz tube with rubber plug and subsequently submitted for GC-TCD analysis.

The GC-TCD analysis was carried out using the Agilent 7890A with a dual detector system consisting of a three-valve four-column-FID and TCD. Four capillary columns were employed, including an HP-AL/S capillary column (50 m×0.53 mm×15 mm), an HP-Plot/Q capillary column (2 m×0.53 mm×40 mm), an HP-Plot/Q capillary column (30 m×0.53 mm×40 mm), and an HP-molesieve capillary column (30 m×0.53 mm×25 mm). The temperature program involved an initial column temperature of 60 °C, held for 10 minutes, followed by a ramp at a rate of 20 °C/min to 180 °C, and a final hold for 5 minutes. The TCD detector operated at a temperature of 250 °C with a compensation gas flow of 2 mL/min. High-purity helium was used as the carrier gas with a flow rate of 5 mL/min. The injection method was valve injection with a 1 mL quantitative loop, and the valve chamber temperature was set at 100 °C. During the period from 2.8 minutes to 7 minutes, the HP-molesieve capillary column was closed. Subsequently, direct chromatographic analysis of CO_2, N_2O, Xe, H_2S, etc., was conducted through the HP-Plot/Q capillary column. After 7 minutes, the HP-molesieve capillary column was opened to separate hydrogen, oxygen, nitrogen, methane, and NO, CO.

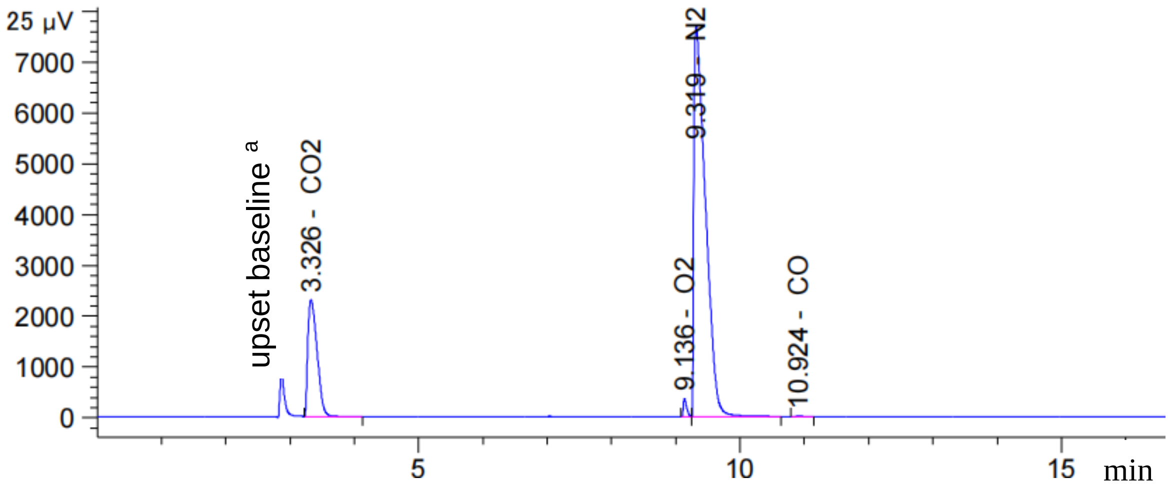

retention time (min)	percent contents (%)	gas composition
3.326	16.76413	CO_2
9.136	1.15843	O_2
9.319	82.02727	N_2
10.924	4.76100e-2	CO

Figure S7. Headspace gas analysis utilizing GC-TCD

[a] Disturbed baseline attributed to valve switching, possibly caused by an unstable carrier gas supply.

3 NMR data

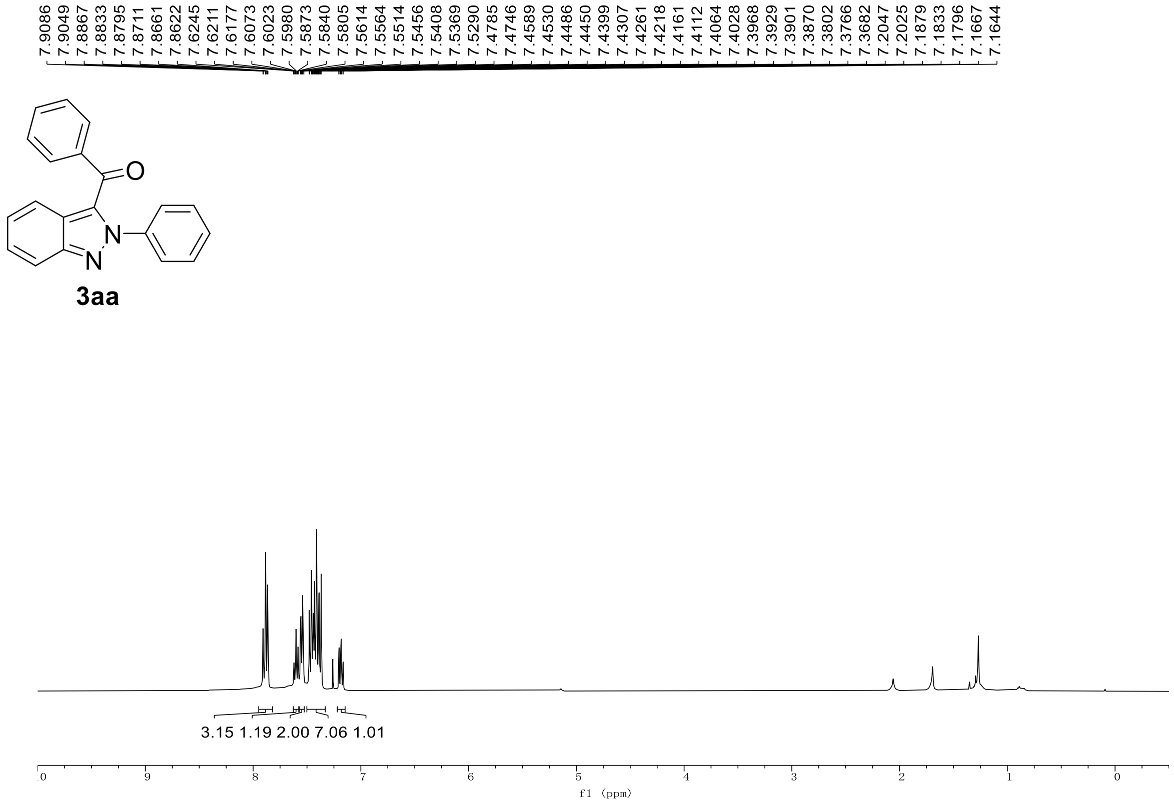

Figure S8 :^{1}H NMR spectrum of **3aa** (400 MHz, $CDCl_3$)

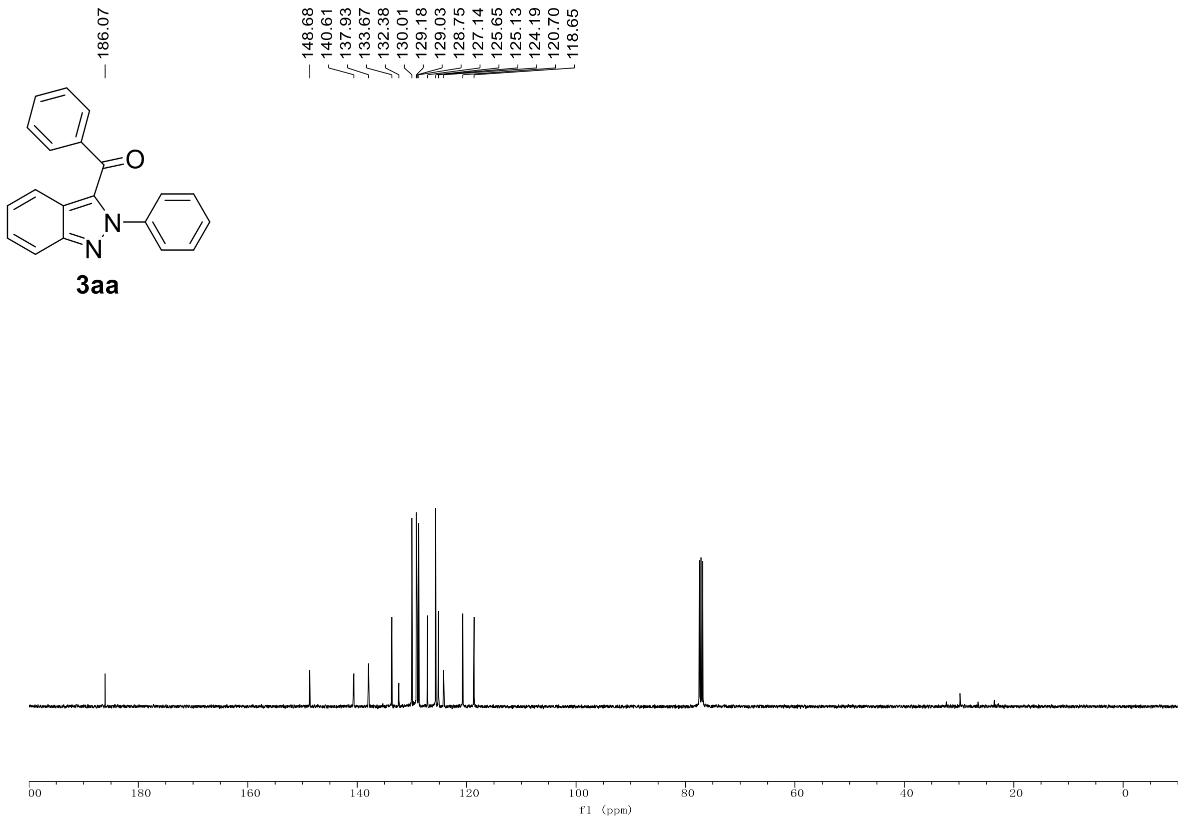

Figure S9 :^{13}C {^{1}H} NMR spectrum of **3aa** (100 MHz, $CDCl_3$)

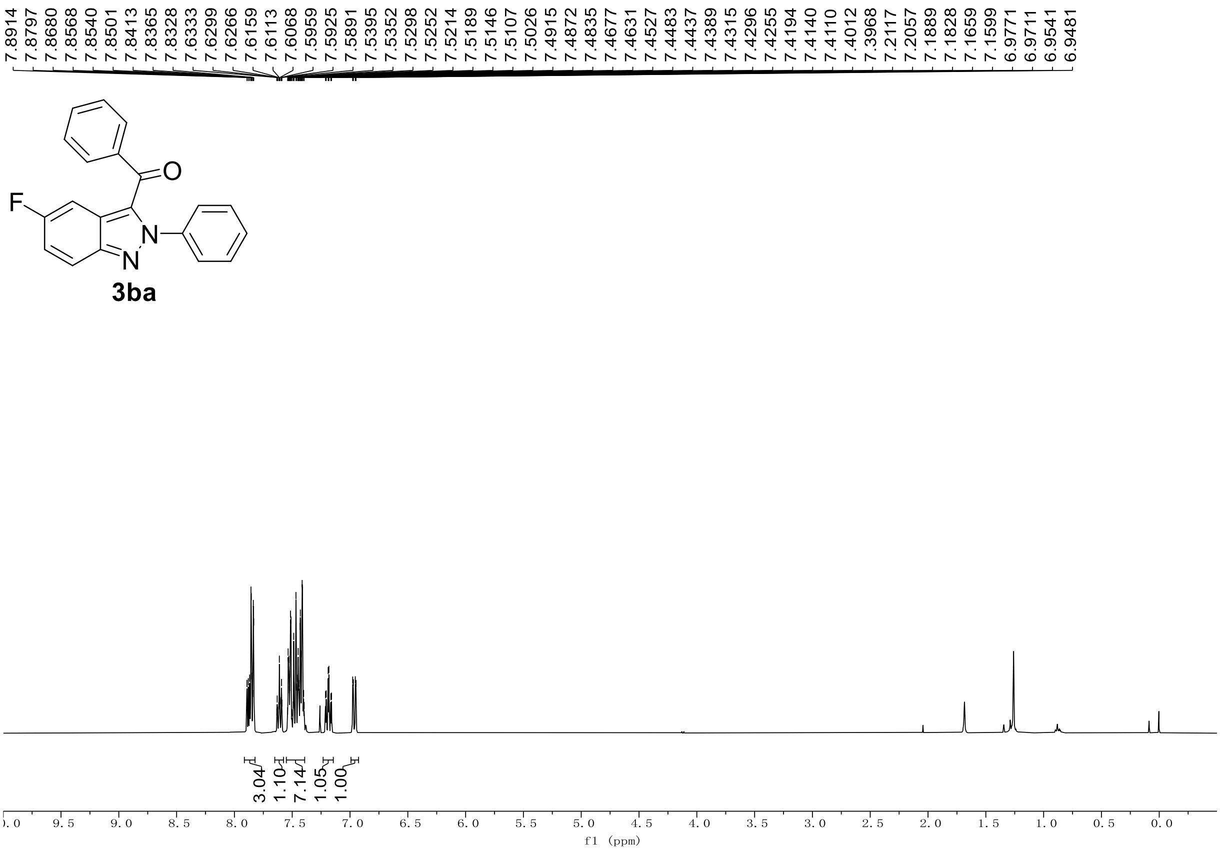

Figure S10 :^{1}H NMR spectrum of **3ba** (400 MHz, $CDCl_3$)

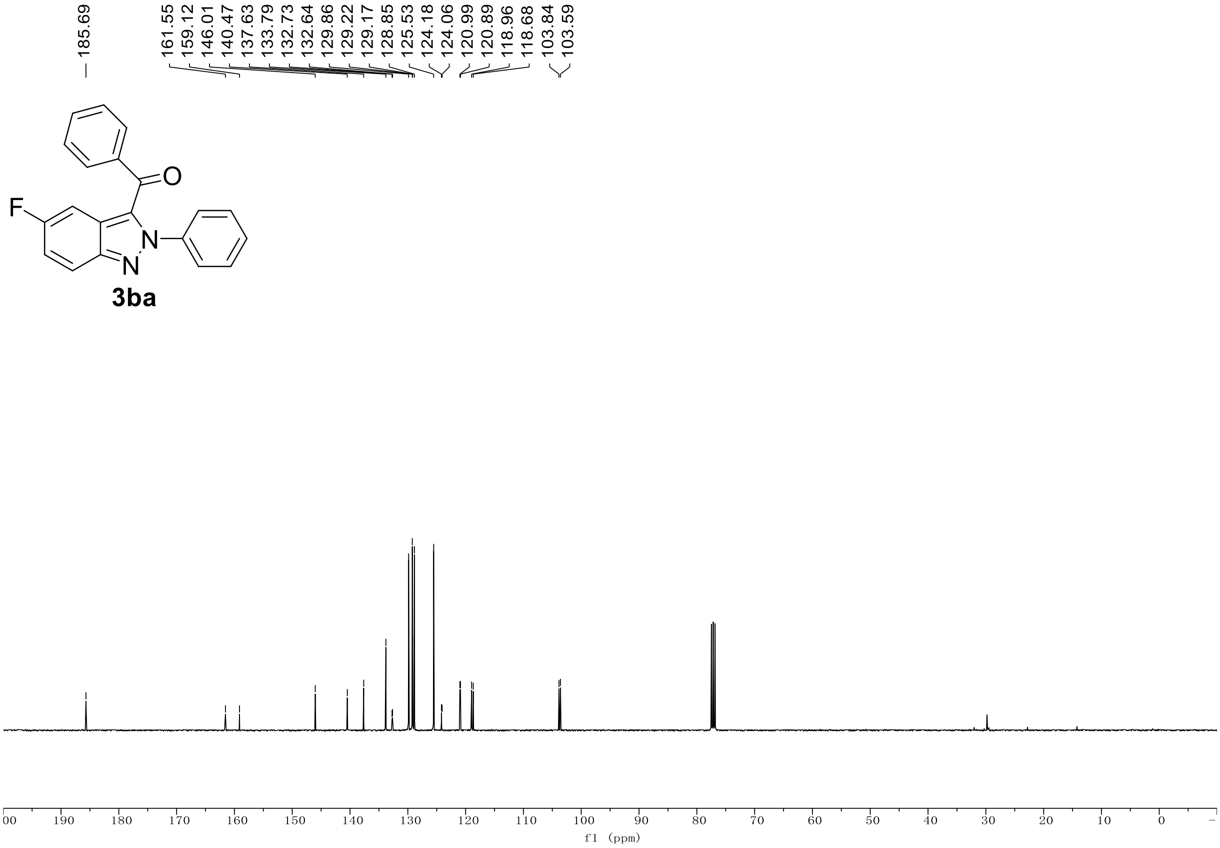

Figure S11 :^{13}C {^{1}H} NMR spectrum of **3ba** (100 MHz, $CDCl_3$)

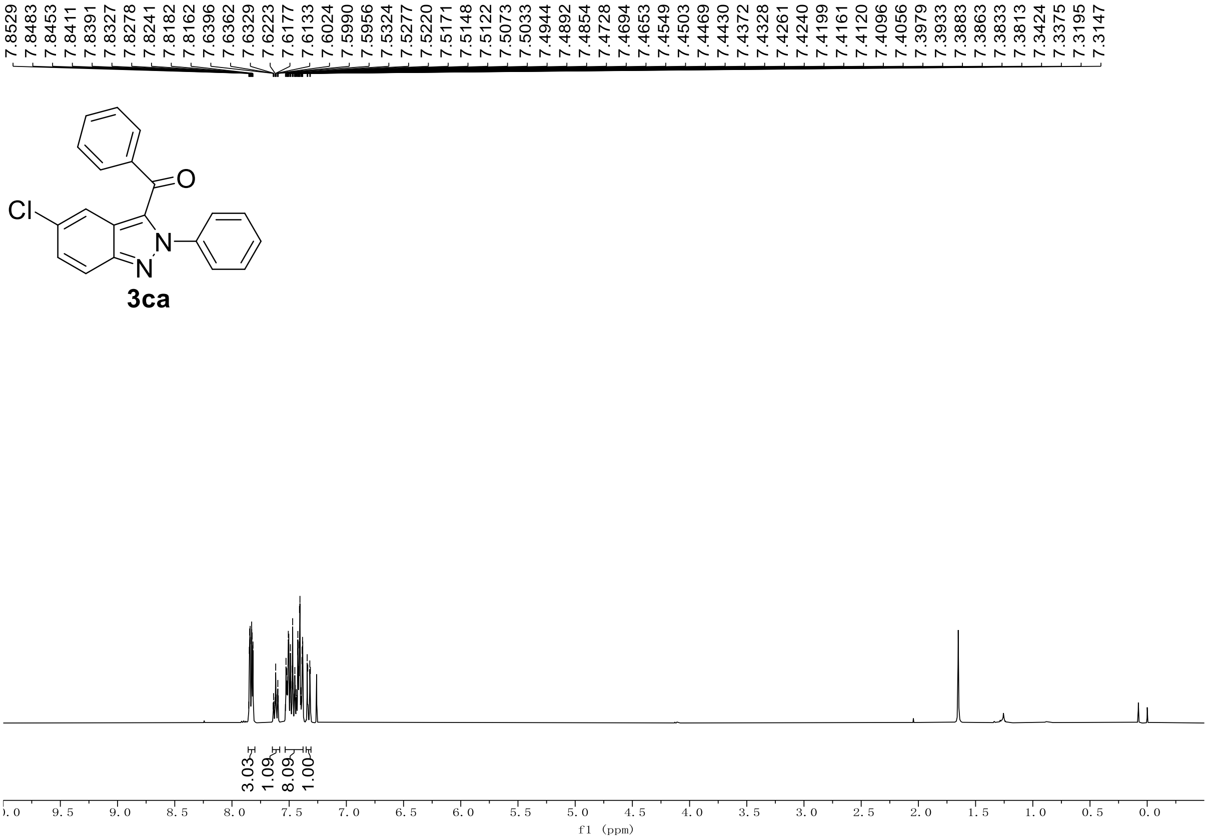

Figure S12 :^{1}H NMR spectrum of **3ca** (400 MHz, $CDCl_3$)

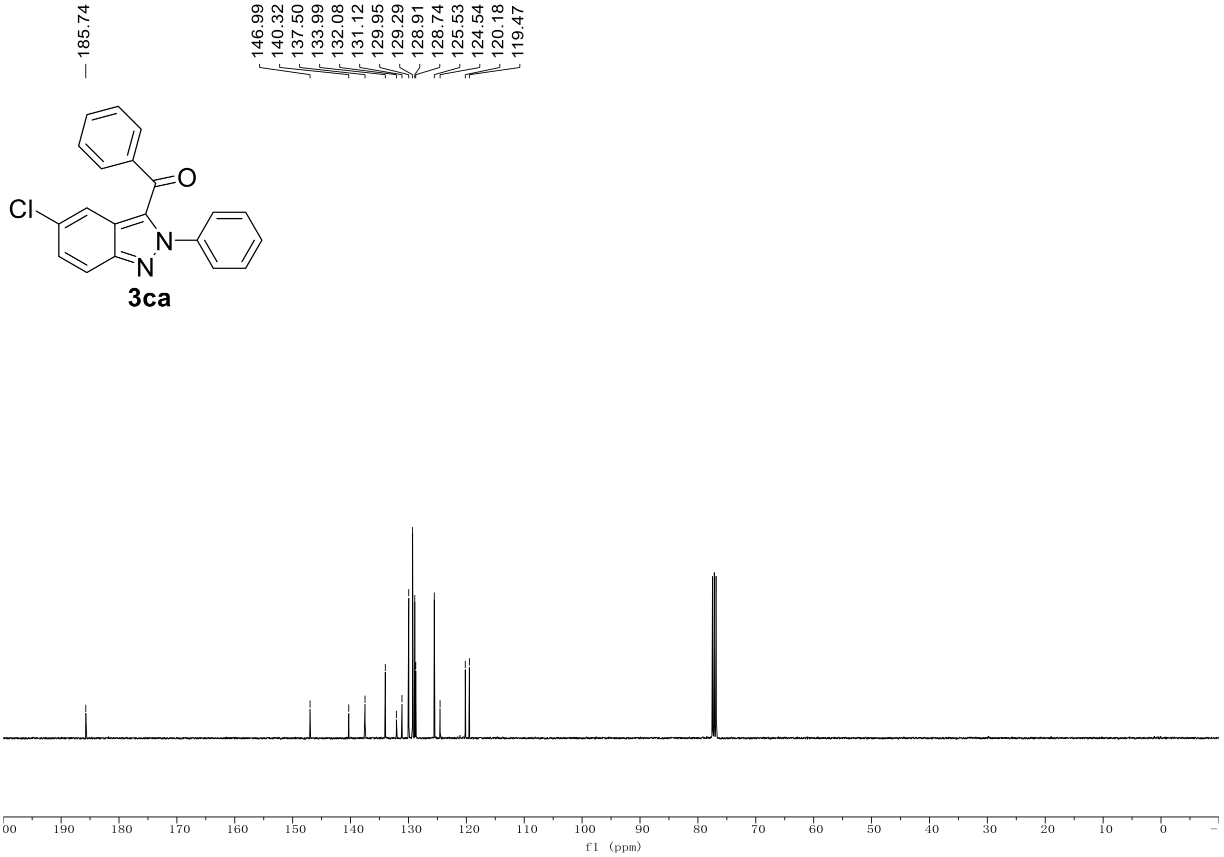

Figure S13 :^{13}C {^{1}H} NMR spectrum of **3ca** (100 MHz, $CDCl_3$)

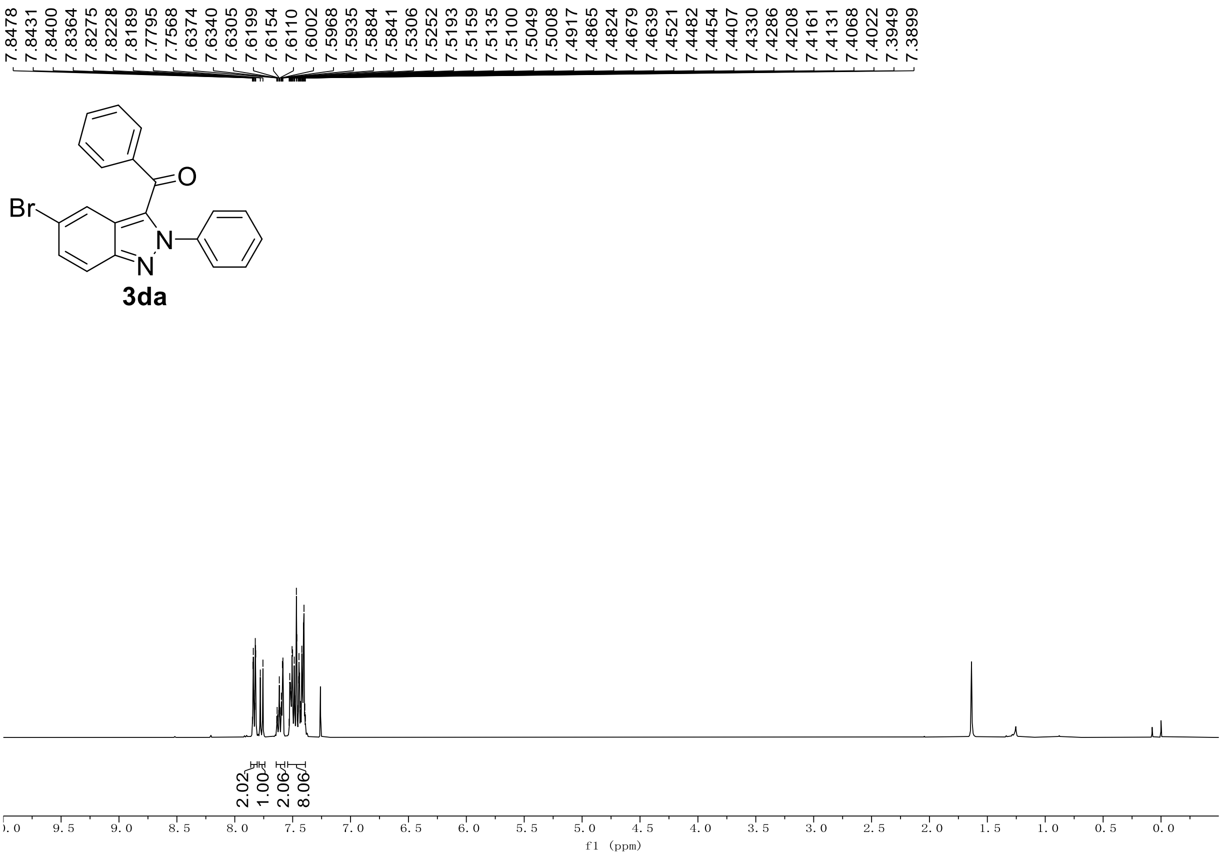

Figure S14 :^{1}H NMR spectrum of **3da** (400 MHz, $CDCl_3$)

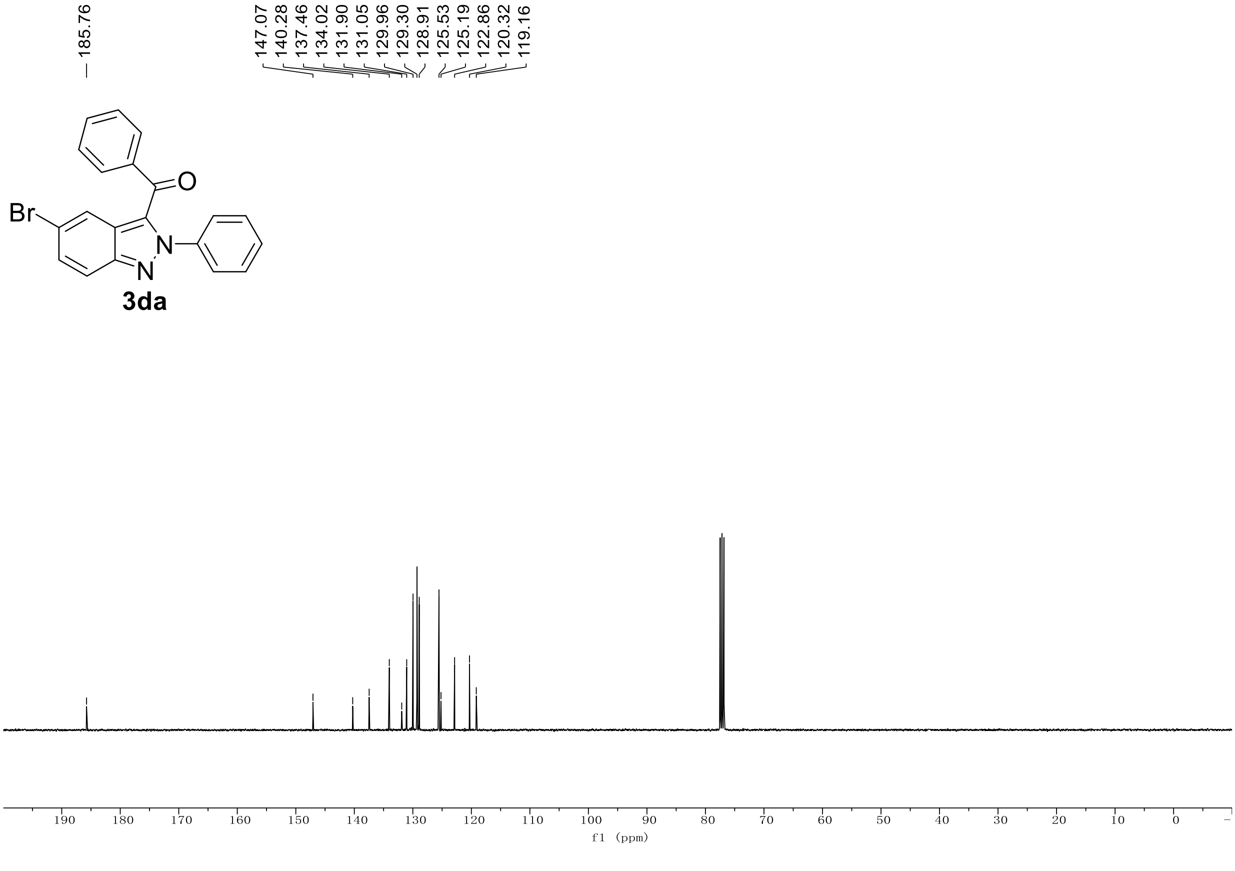

Figure S15 :^{13}C {^{1}H} NMR spectrum of **3da** (100 MHz, $CDCl_3$)

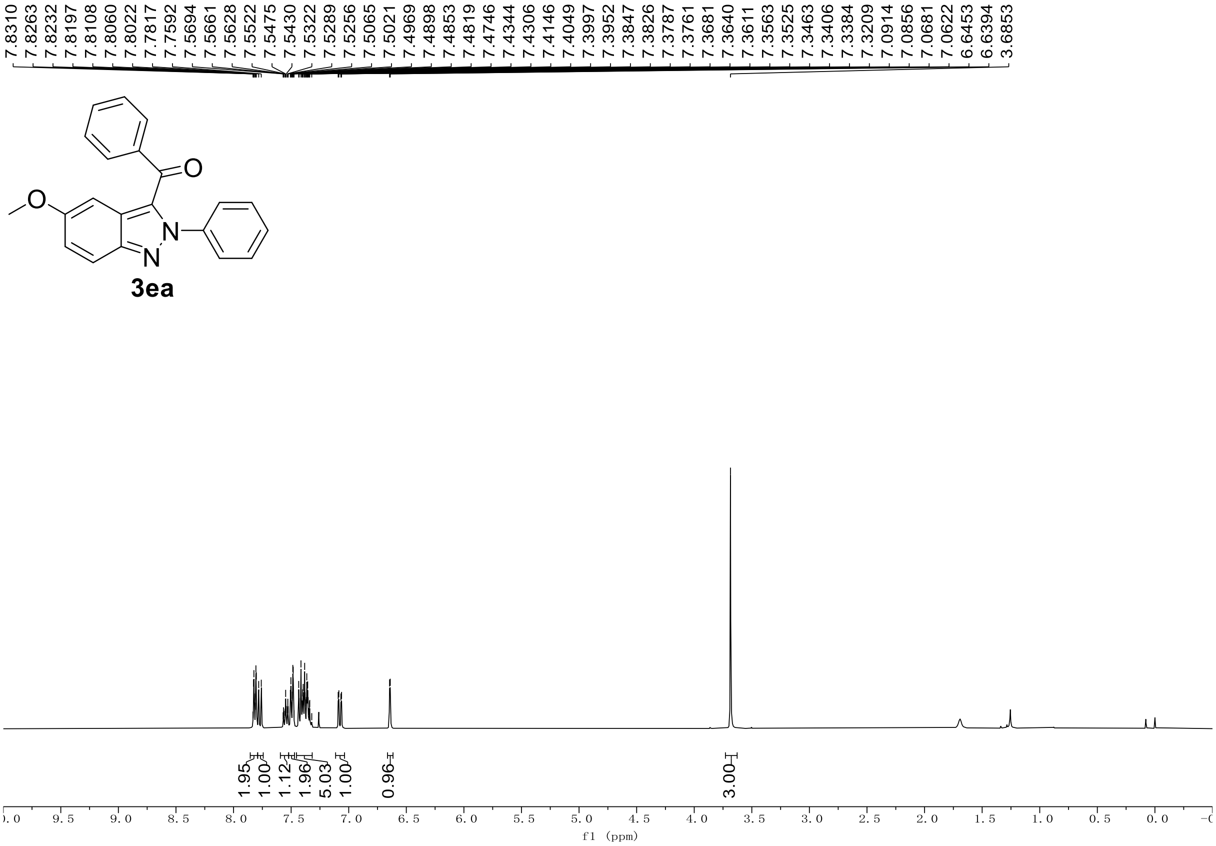

Figure S16 :^{1}H NMR spectrum of **3ea** (400 MHz, $CDCl_3$)

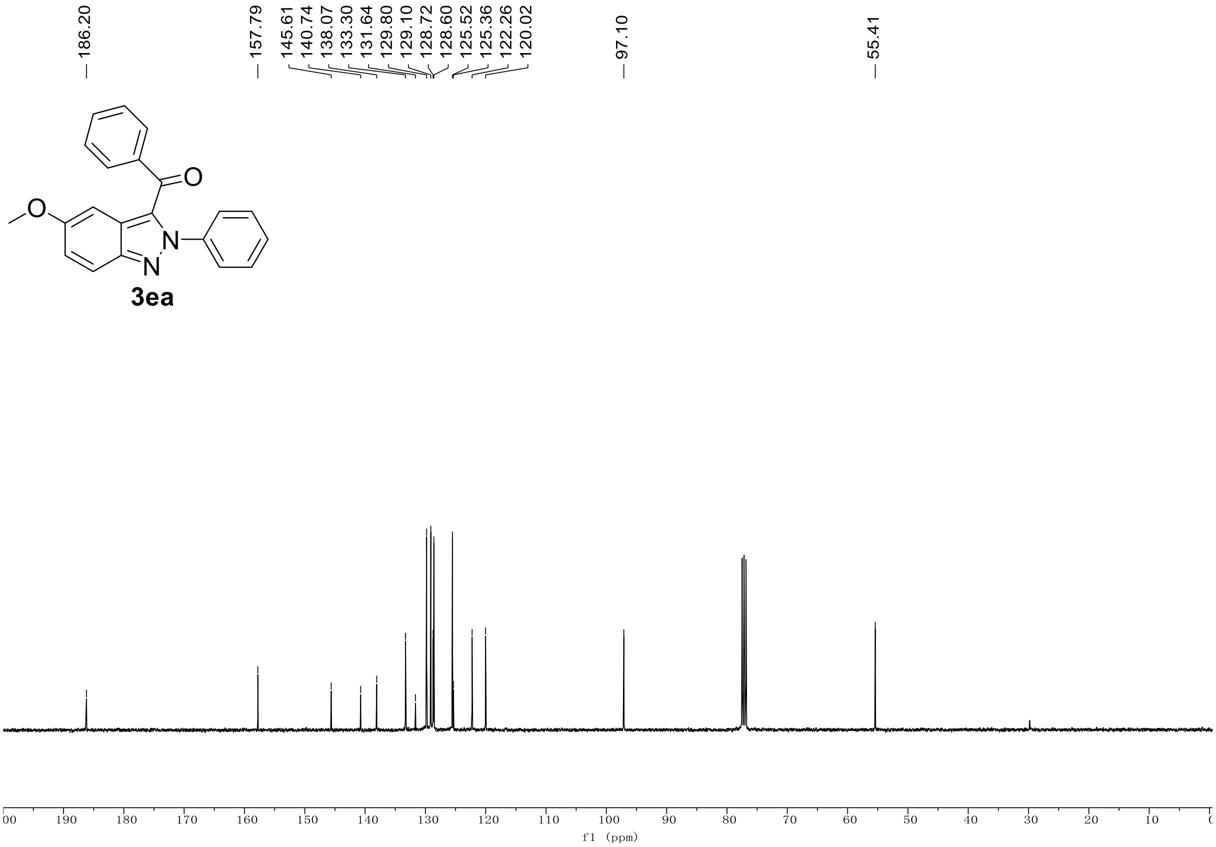

Figure S17 :^{13}C {^{1}H} NMR spectrum of **3ea** (100 MHz, $CDCl_3$)

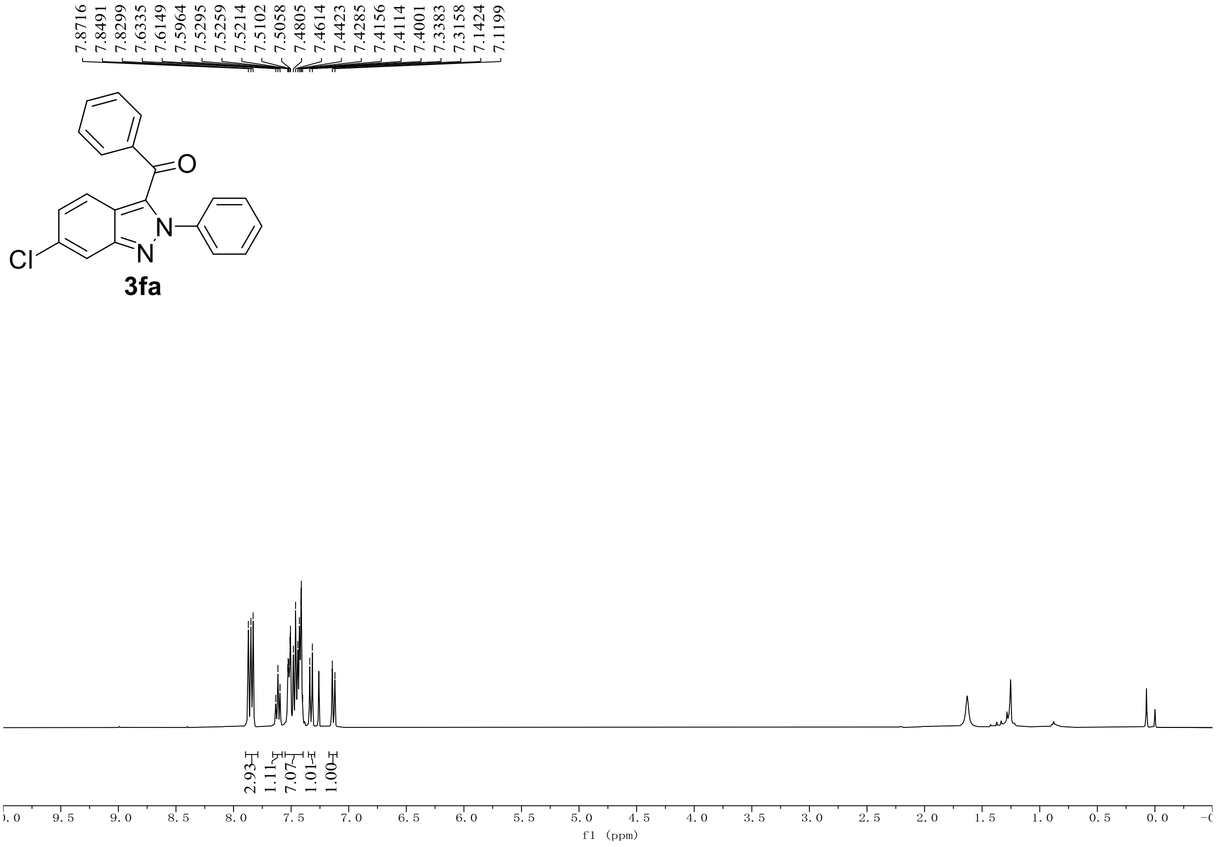

Figure S18 :^{1}H NMR spectrum of **3fa** (400 MHz, $CDCl_3$)

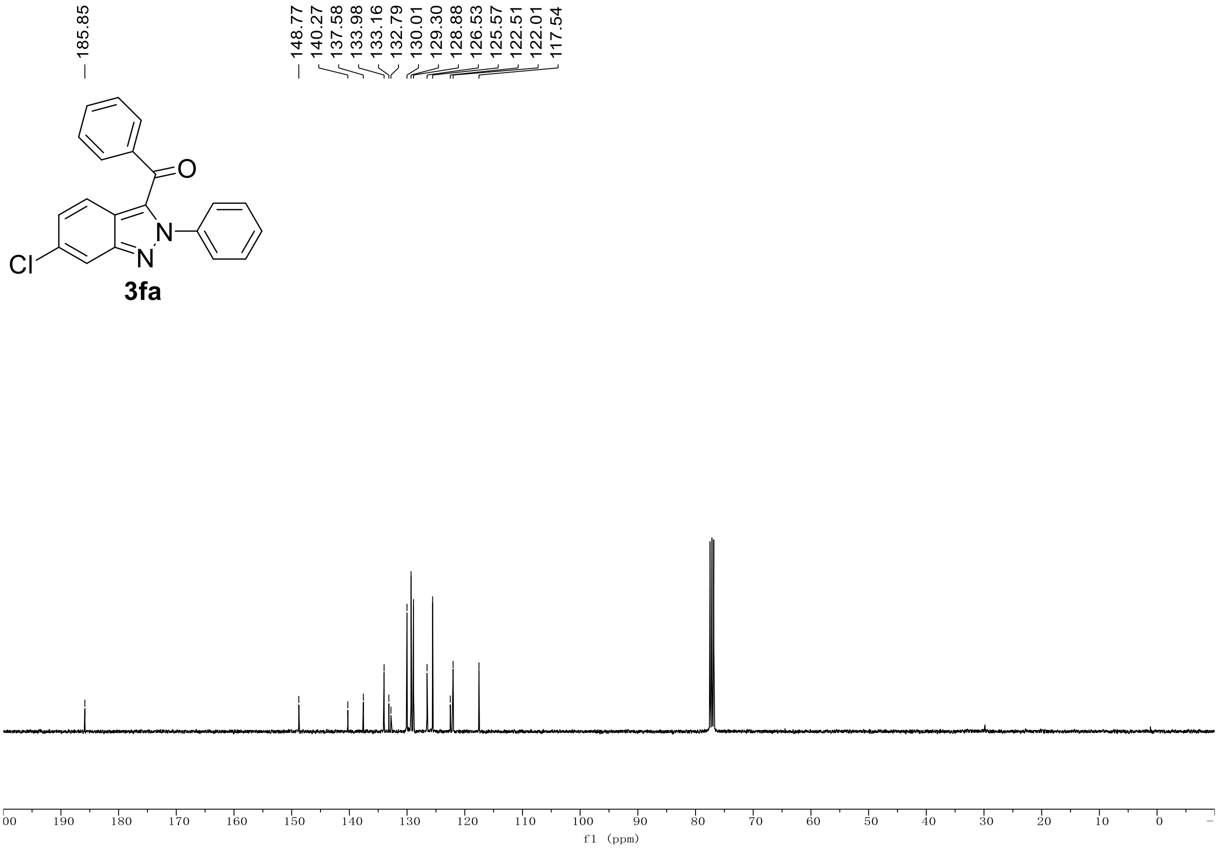

Figure S19 :^{13}C {^{1}H} NMR spectrum of **3fa** (100 MHz, $CDCl_3$)

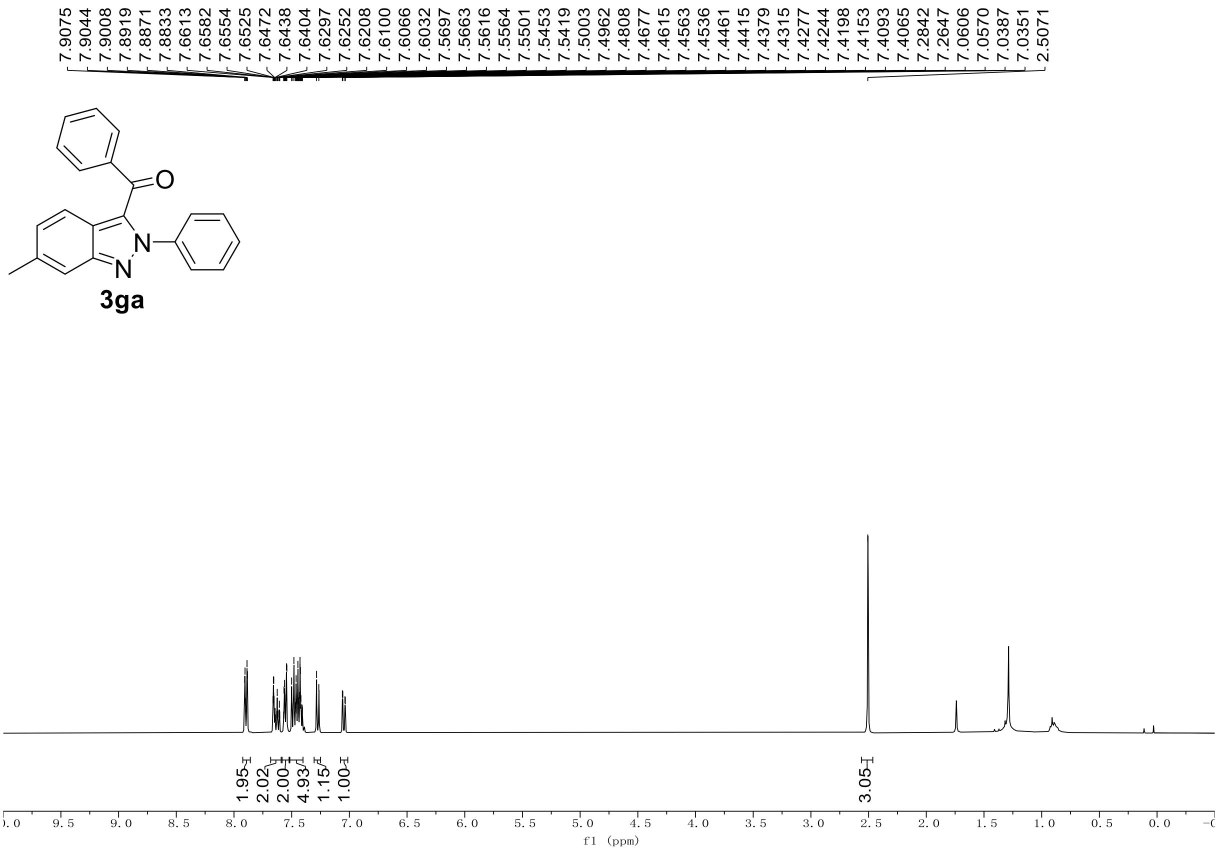

Figure S20 :^{1}H NMR spectrum of **3ga** (400 MHz, $CDCl_3$)

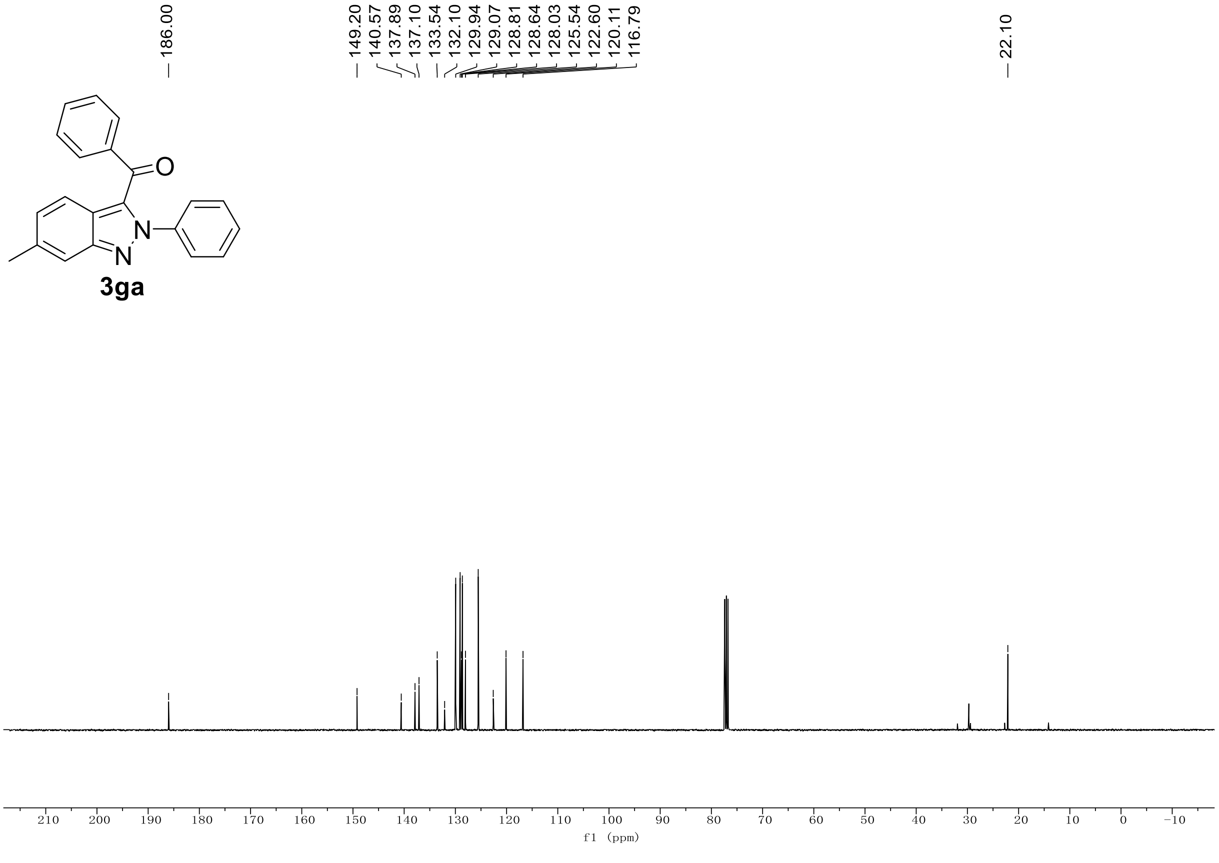

Figure S21 :^{13}C {^{1}H} NMR spectrum of **3ga** (100 MHz, $CDCl_3$)

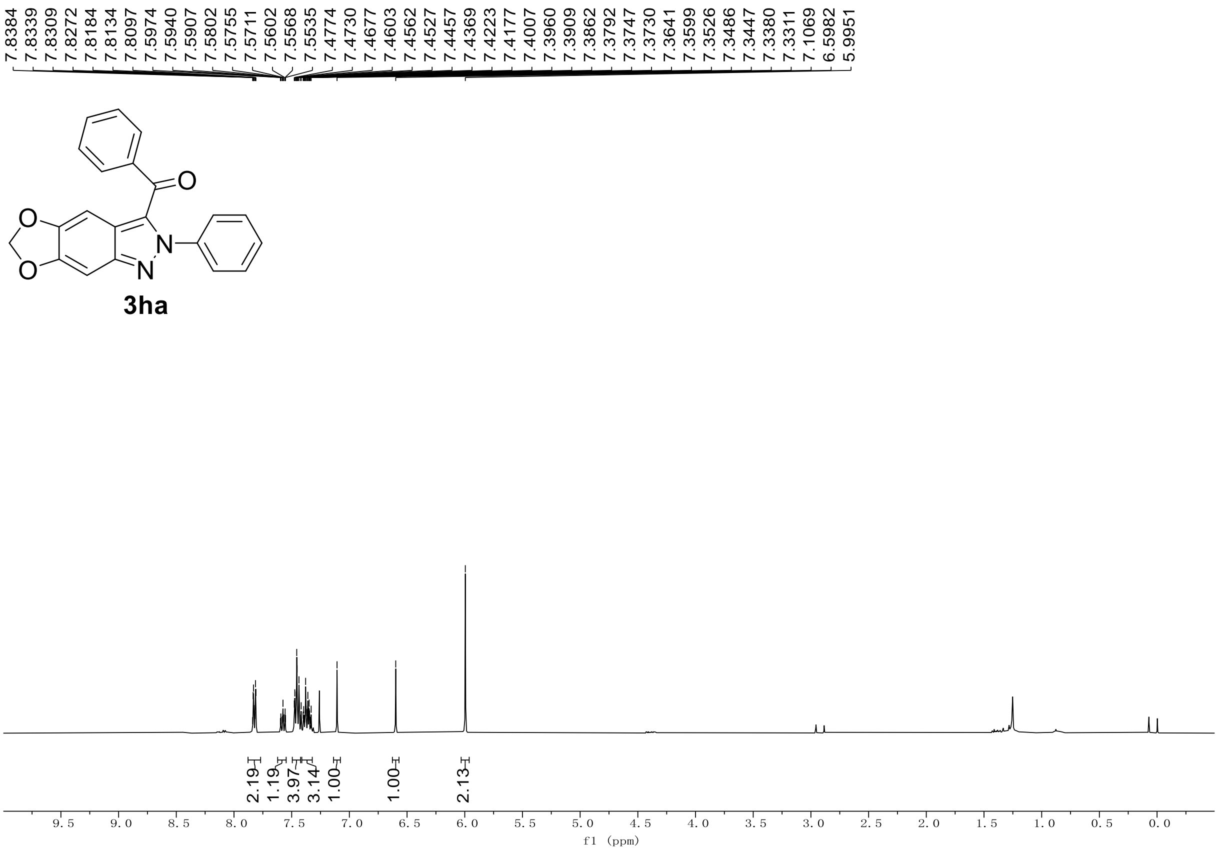

Figure S22 :^{1}H NMR spectrum of **3ha** (400 MHz, $CDCl_3$)

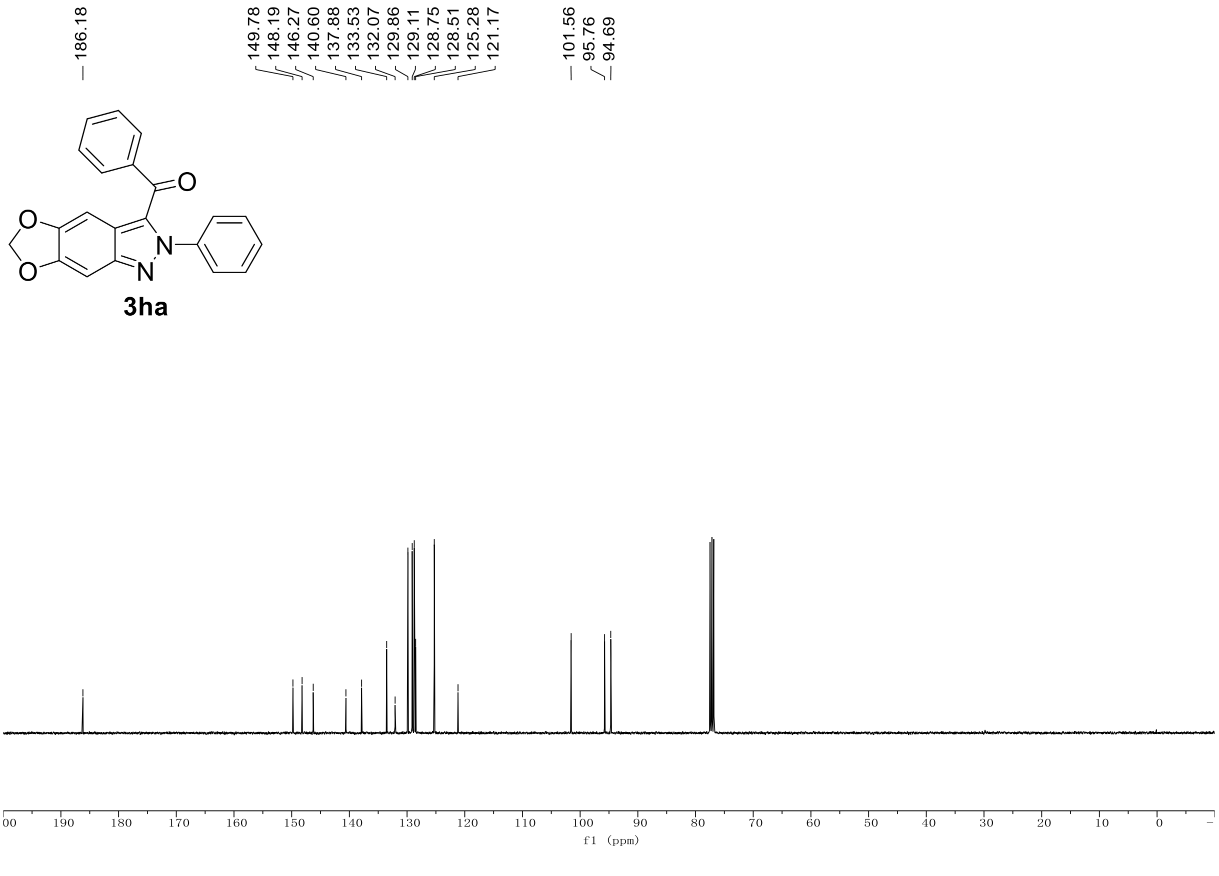

Figure S23 :^{13}C {^{1}H} NMR spectrum of **3ha** (100 MHz, $CDCl_3$)

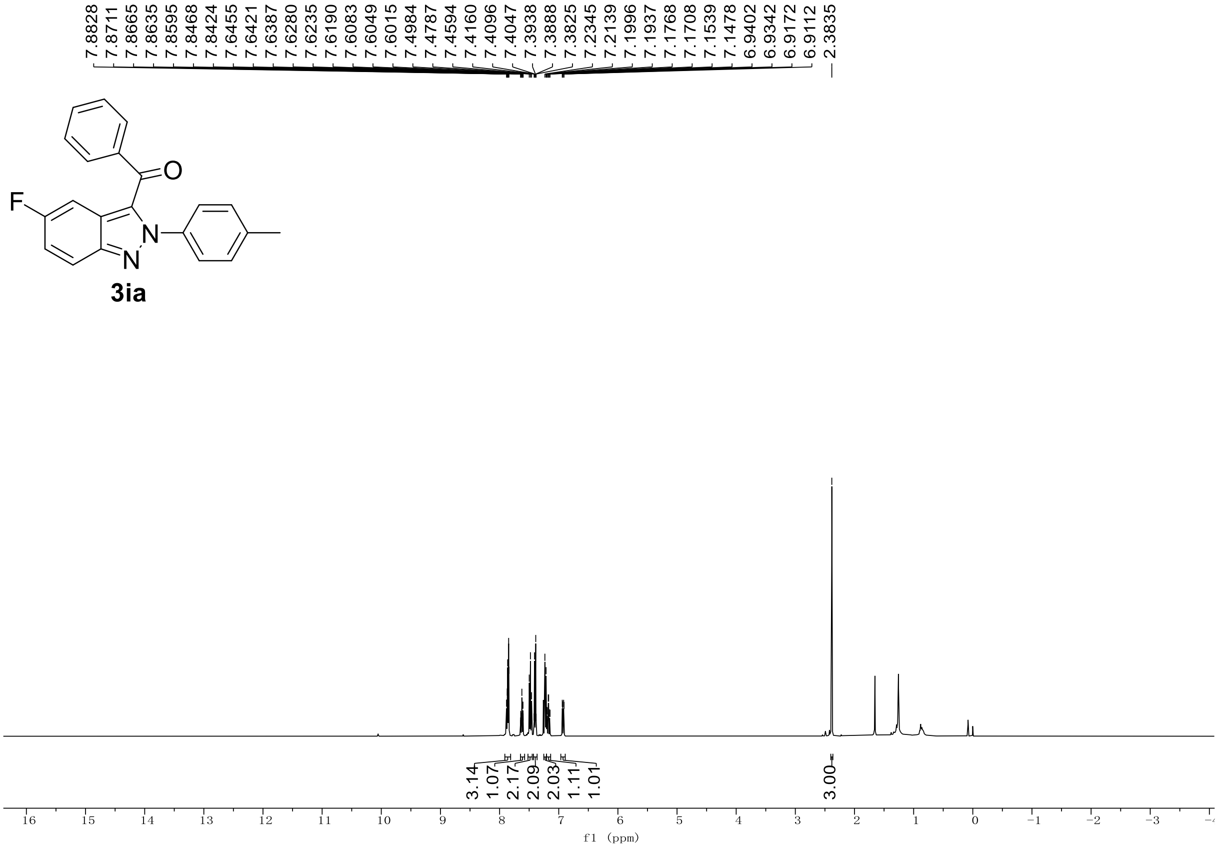

3ia

Figure S24 :^{1}H NMR spectrum of **3ia** (400 MHz, $CDCl_3$)

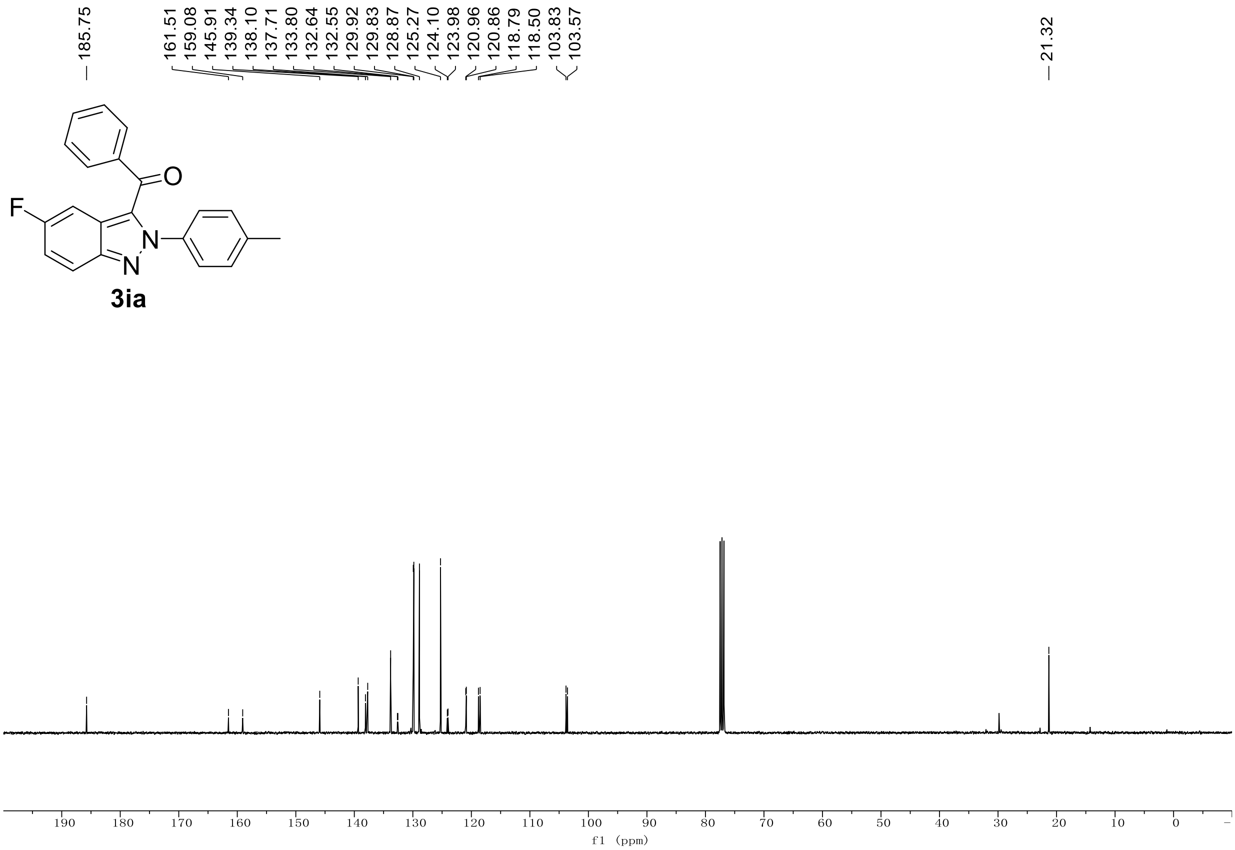

3ia

Figure S25 :^{13}C {^{1}H} NMR spectrum of **3ia** (100 MHz, $CDCl_3$)

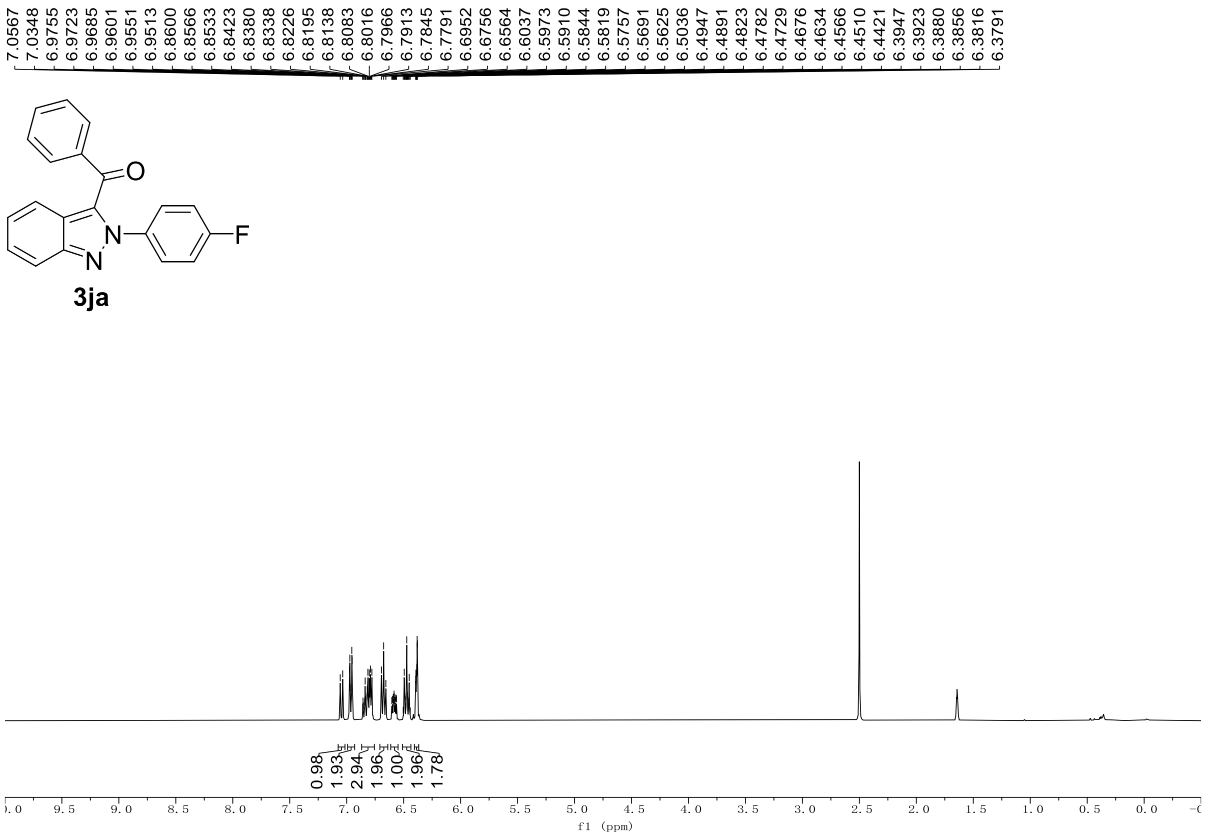

Figure S26 :^{1}H NMR spectrum of **3ja** (400 MHz, $(CD_3)_2SO$)

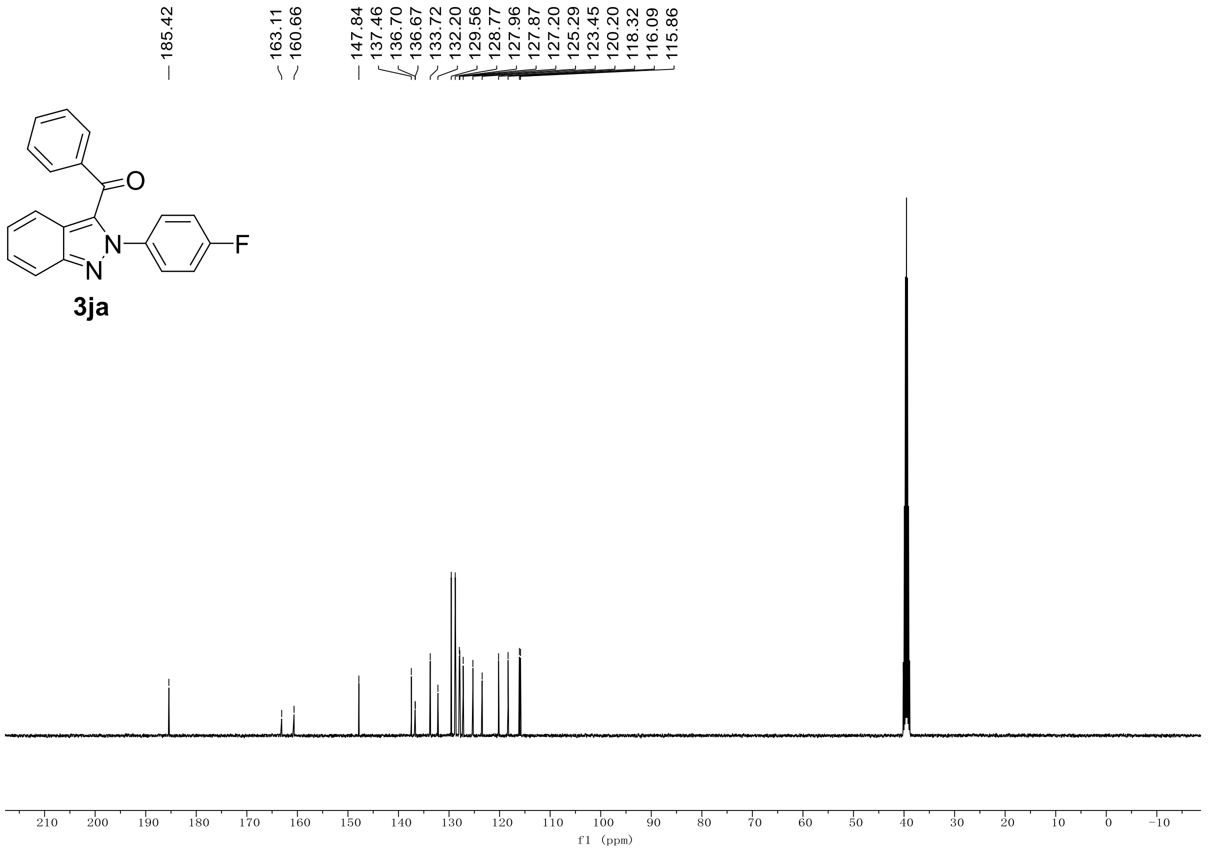

Figure S27 :^{13}C {^{1}H} NMR spectrum of **3ja** (100 MHz, $(CD_3)_2SO$)

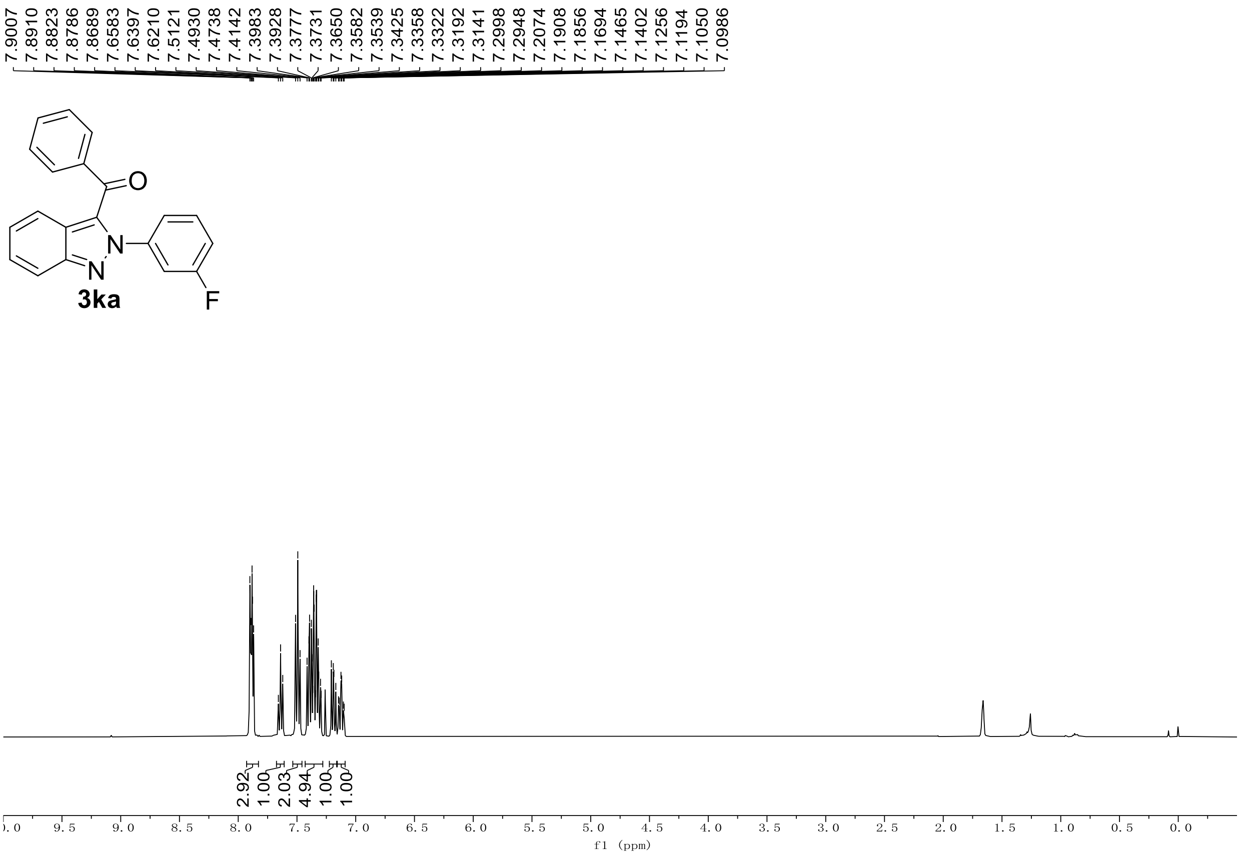

Figure S28 :^{1}H NMR spectrum of **3ka** (400 MHz, $CDCl_3$)

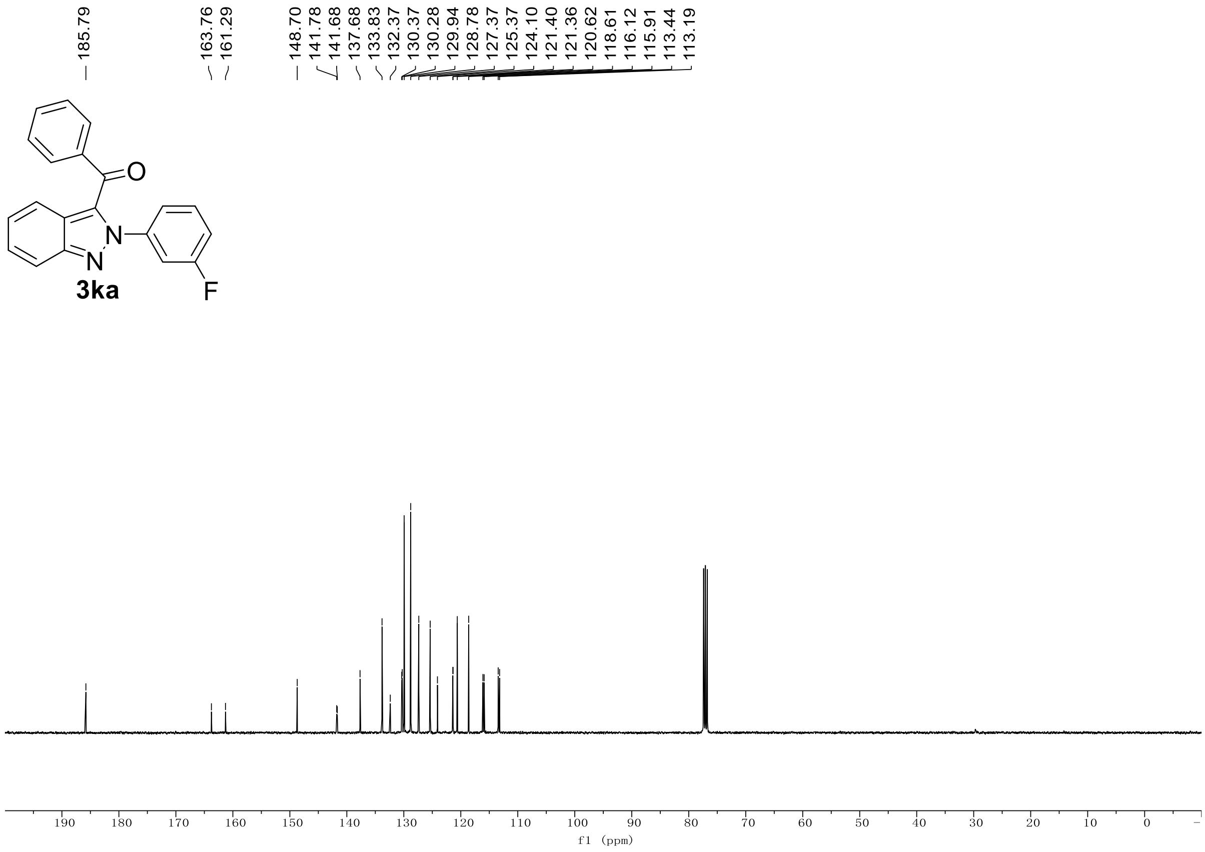

Figure S29 :^{13}C {^{1}H} NMR spectrum of **3ka** (100 MHz, $CDCl_3$)

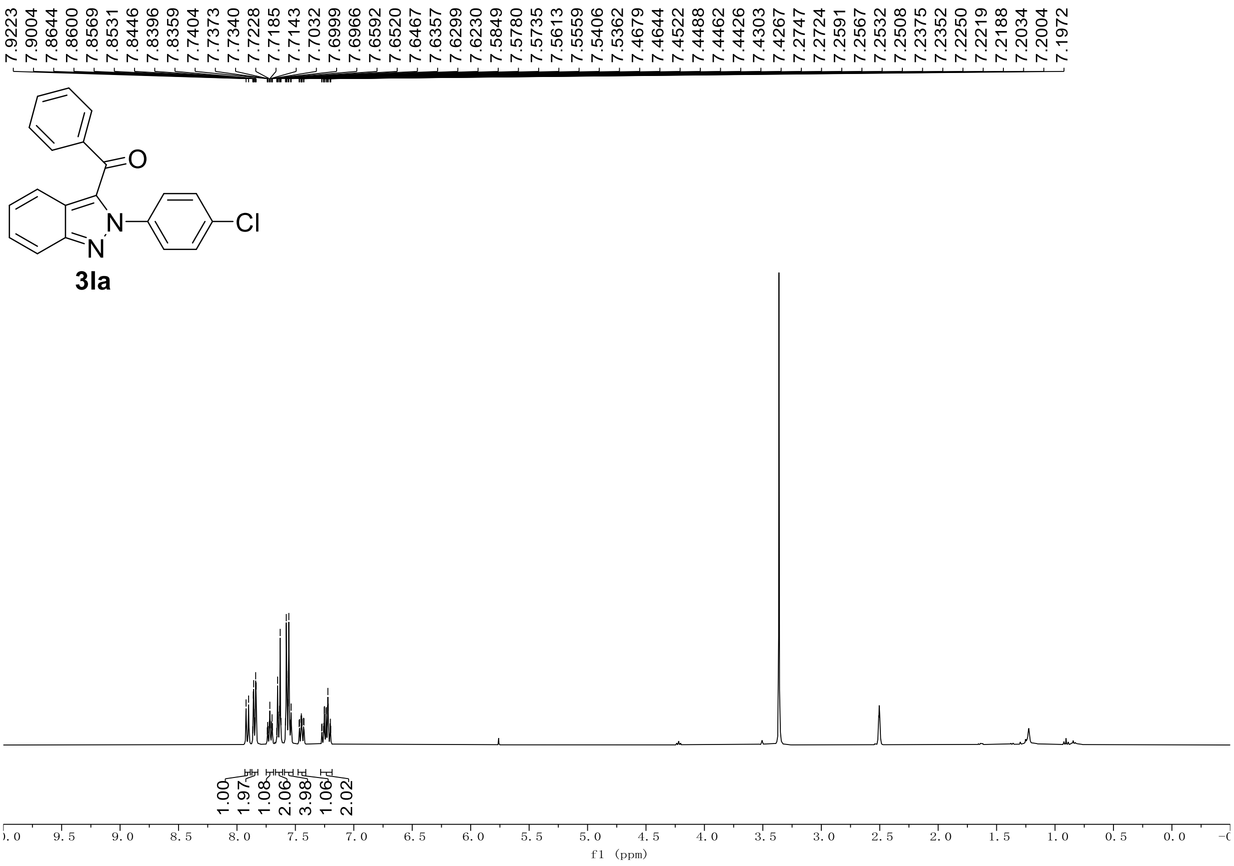

Figure S30 :^{1}H NMR spectrum of **3la** (400 MHz, $(CD_3)_2SO$)

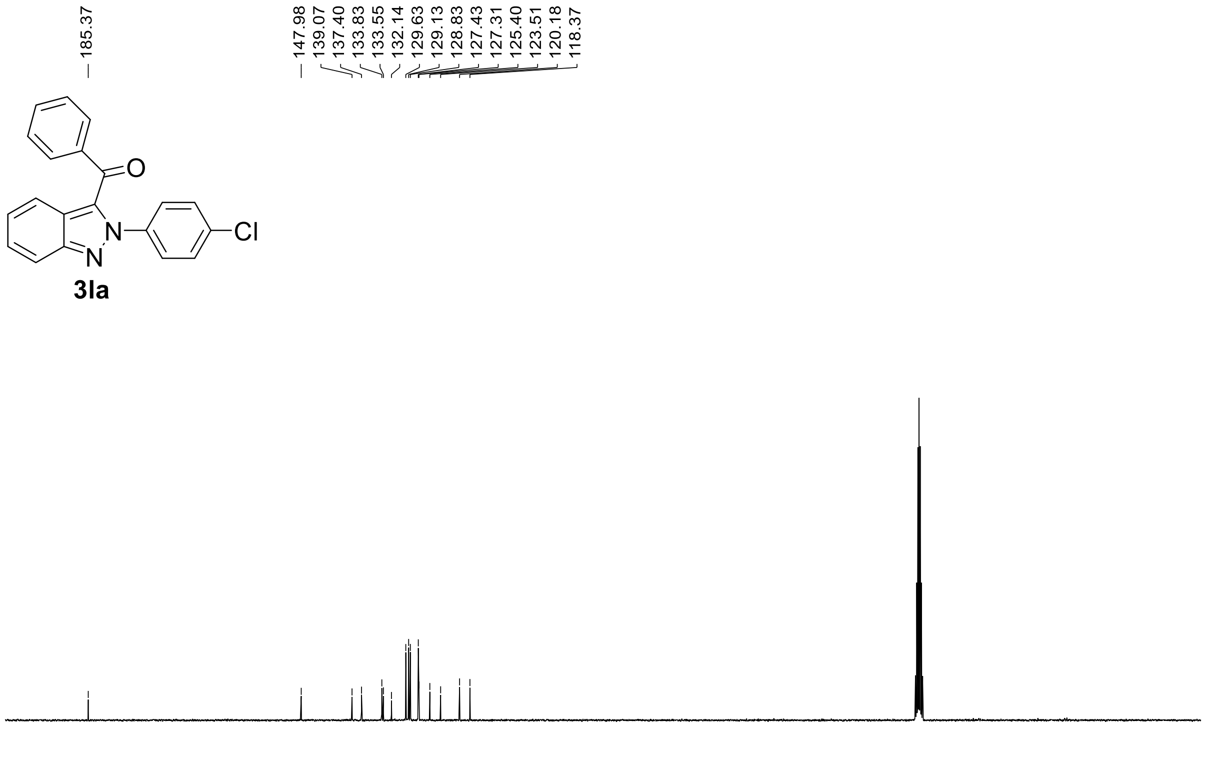

Figure S31 :^{13}C {^{1}H} NMR spectrum of **3la** (100 MHz, $(CD_3)_2SO$)

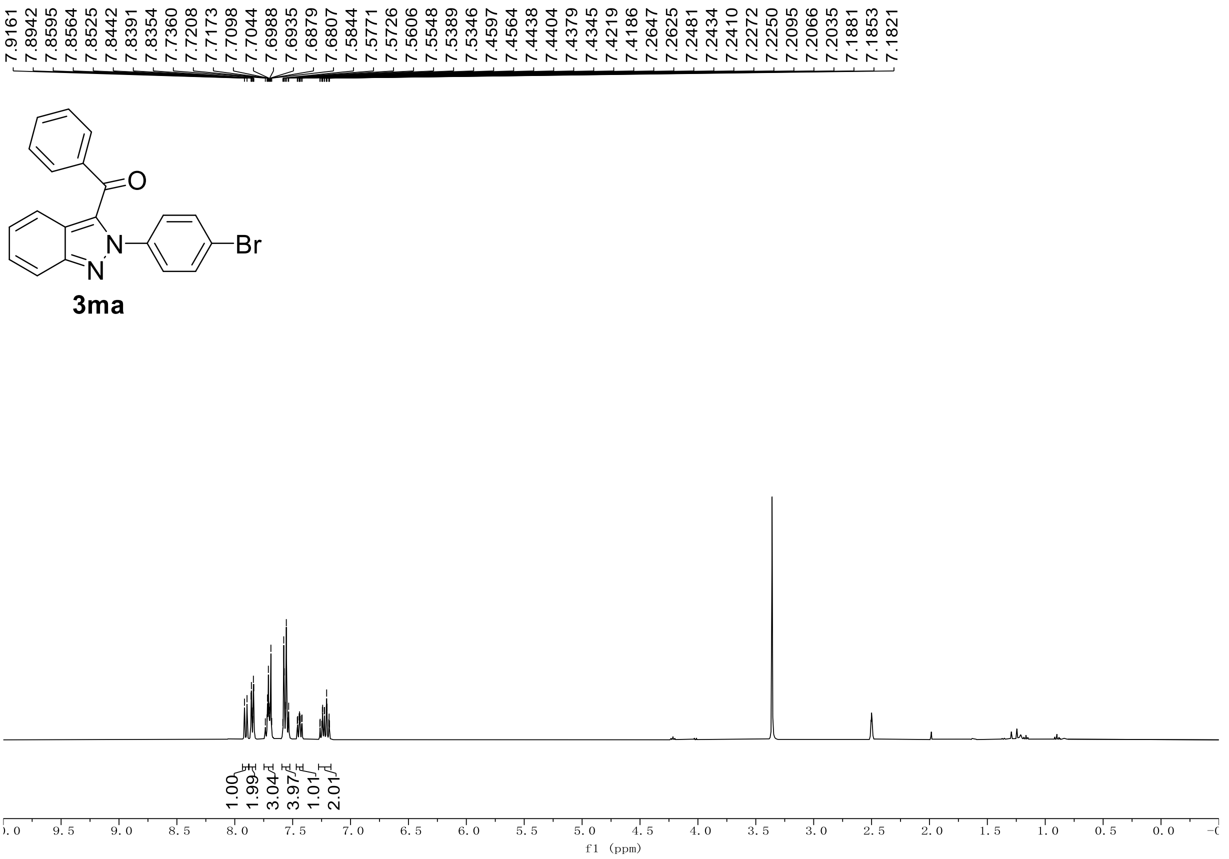

Figure S32 :^{1}H NMR spectrum of **3ma** (400 MHz, $(CD_3)_2SO$)

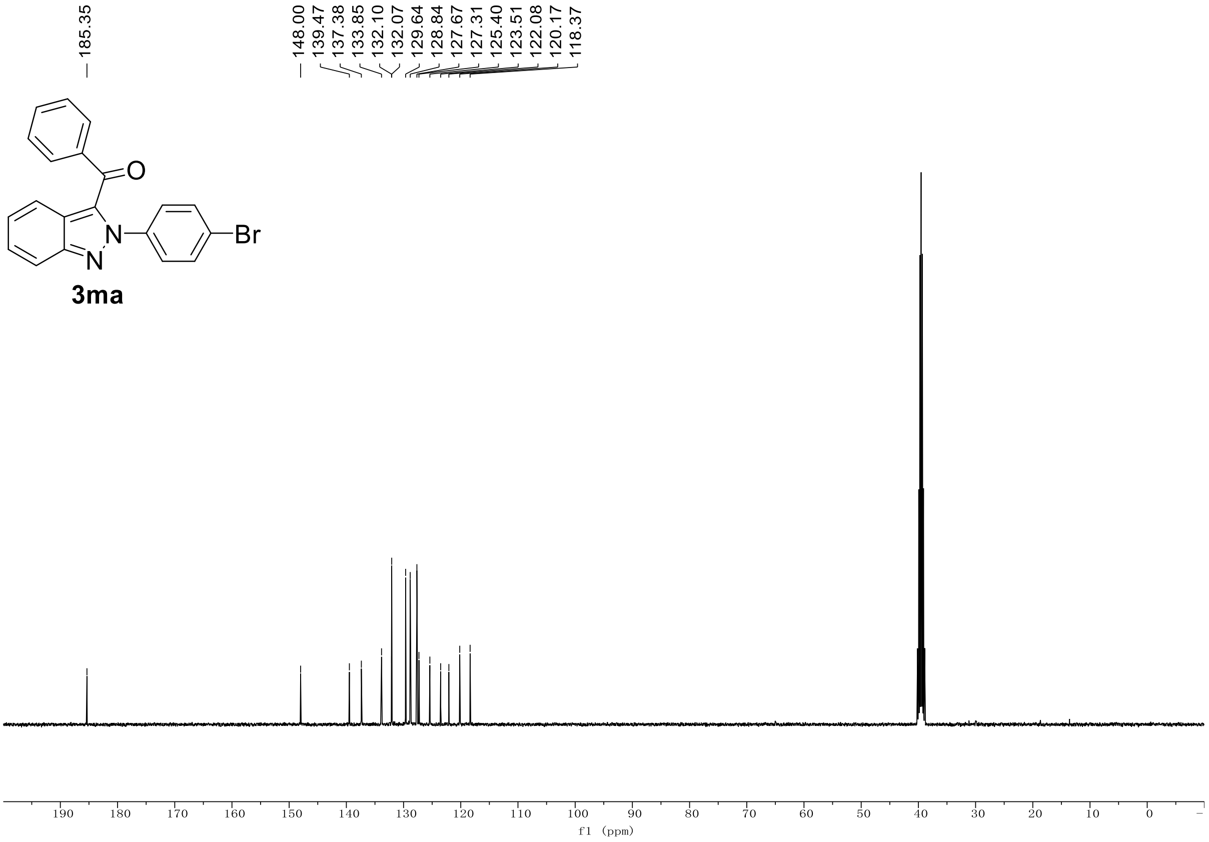

Figure S33 :^{13}C {^{1}H} NMR spectrum of **3ma** (100 MHz, $(CD_3)_2SO$)

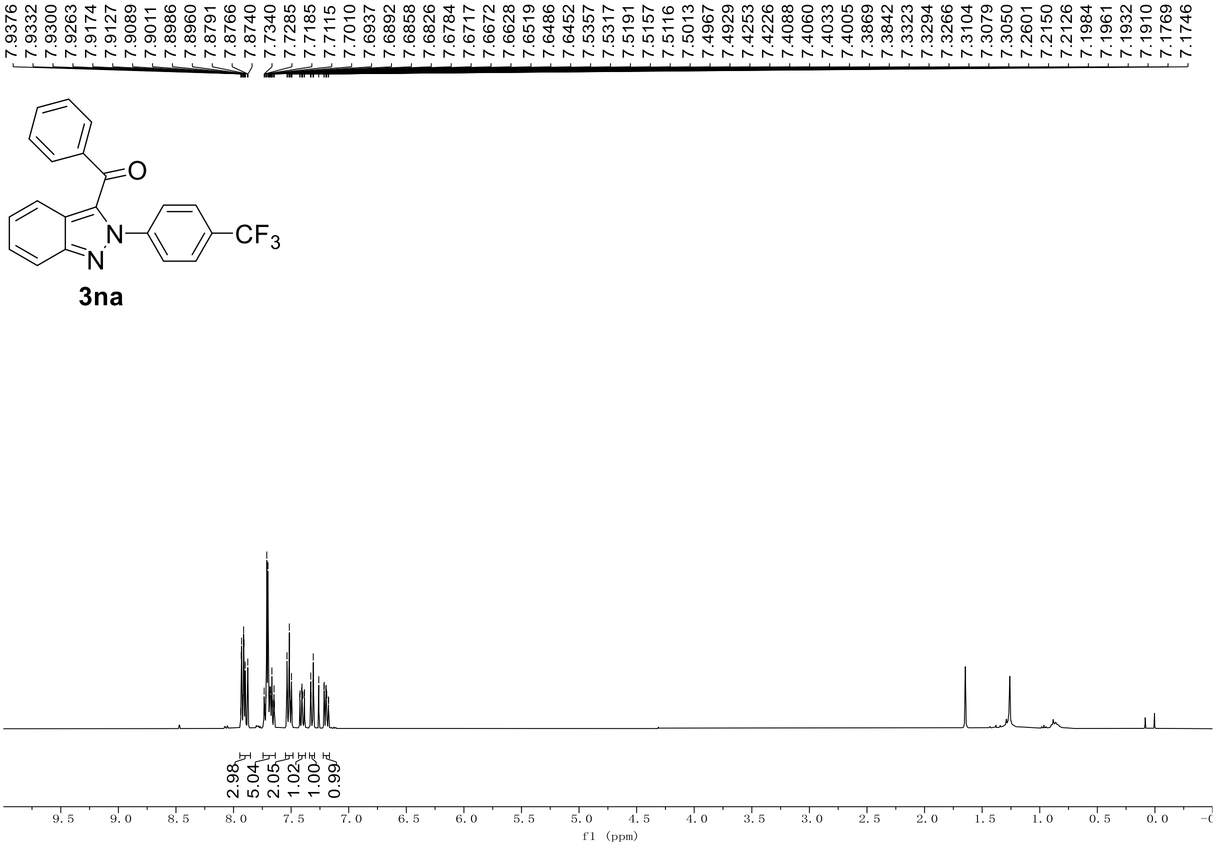

Figure S34 :^{1}H NMR spectrum of **3na** (400 MHz, $CDCl_3$)

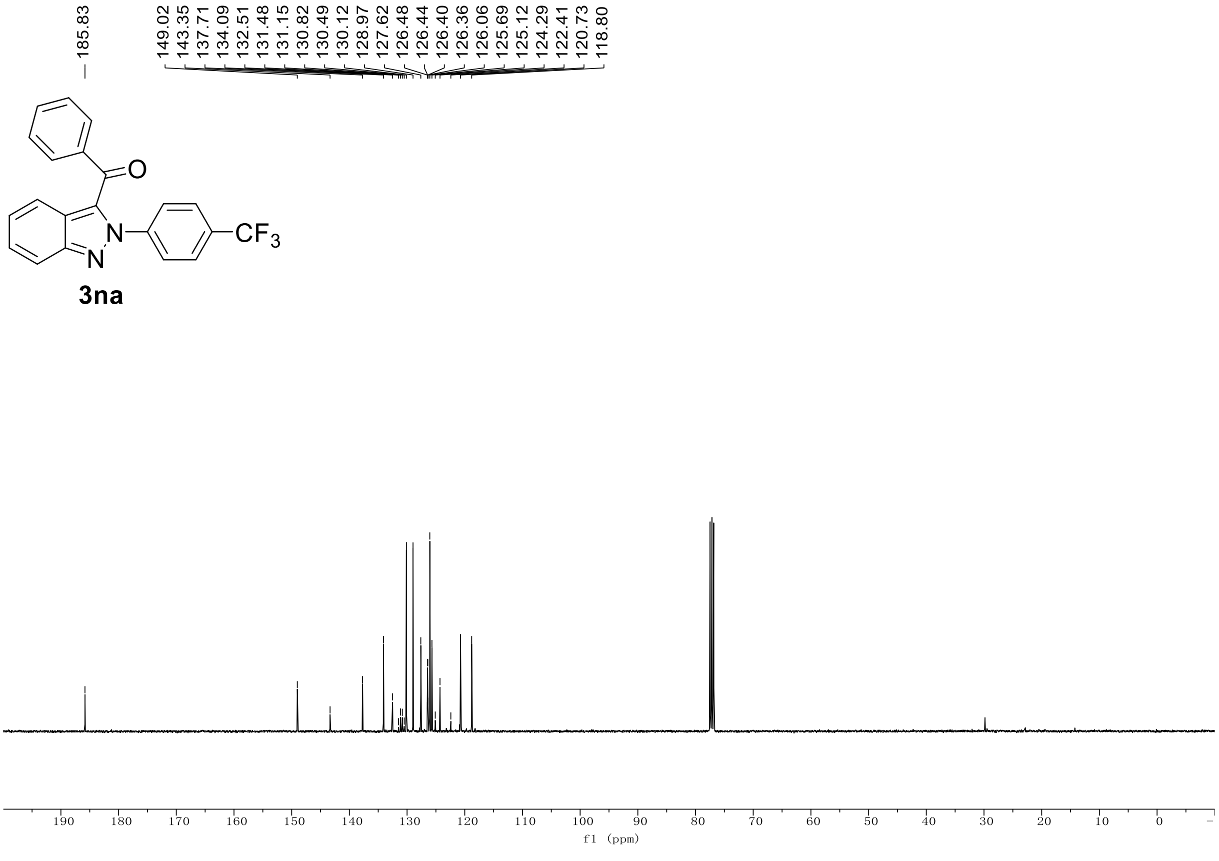

Figure S35 :^{13}C {^{1}H} NMR spectrum of **3na** (100 MHz, $CDCl_3$)

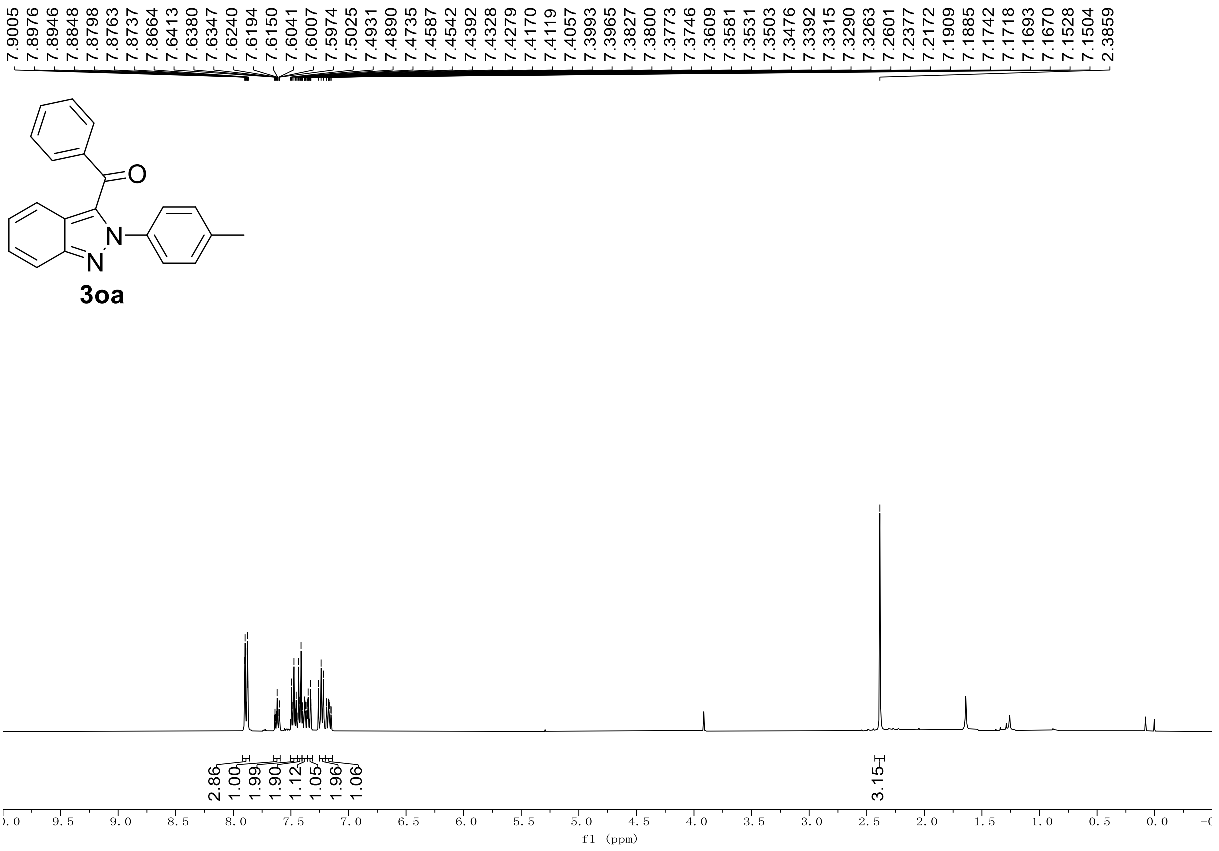

Figure S36 :^{1}H NMR spectrum of **3oa** (400 MHz, $CDCl_3$)

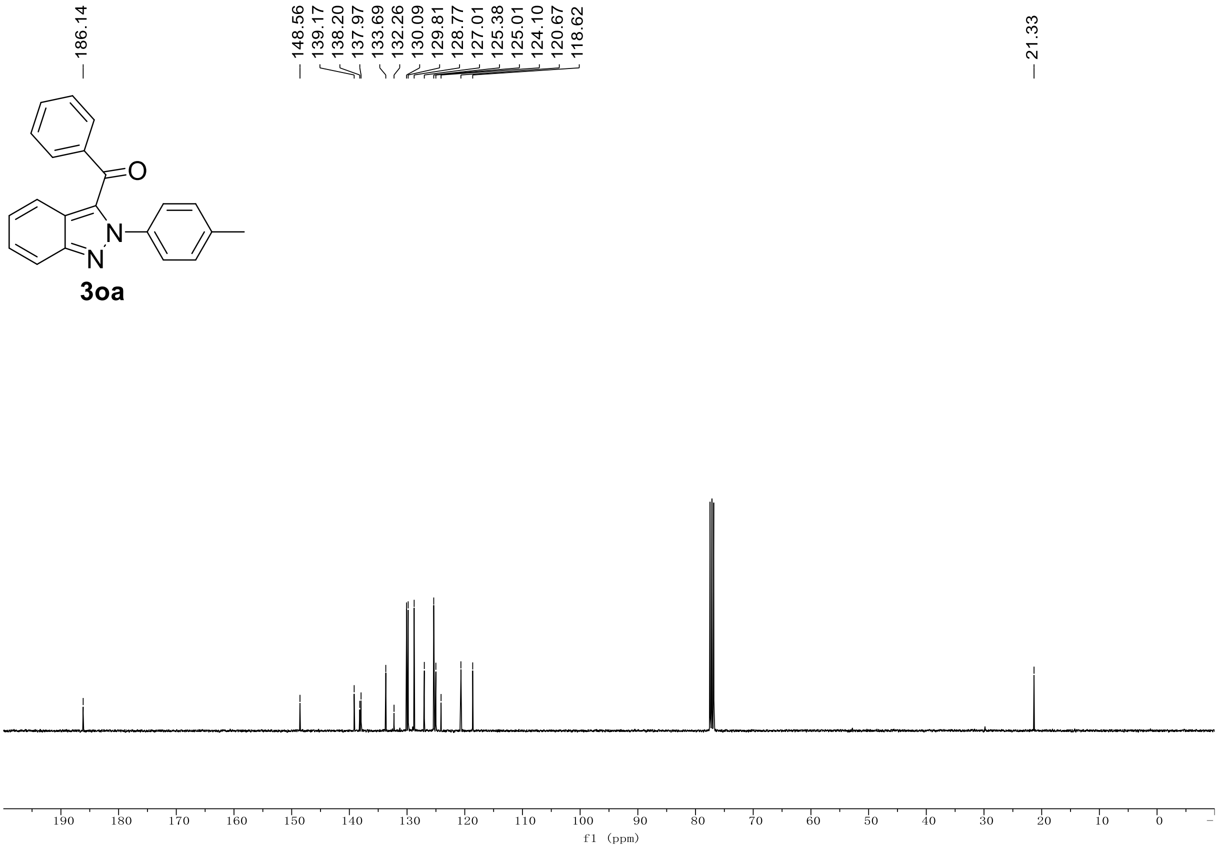

Figure S37 :^{13}C {^{1}H} NMR spectrum of **3oa** (100 MHz, $CDCl_3$)

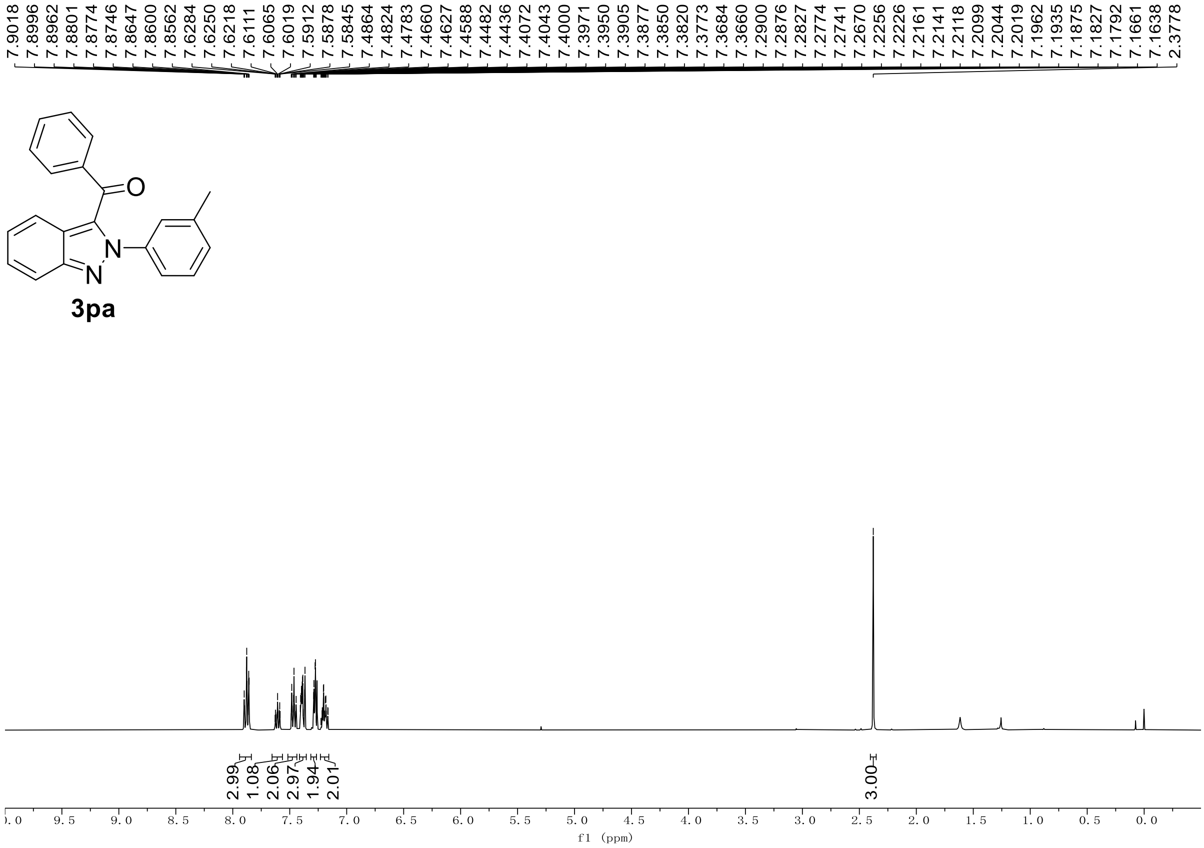

Figure S38 :^{1}H NMR spectrum of **3pa** (400 MHz, $CDCl_3$)

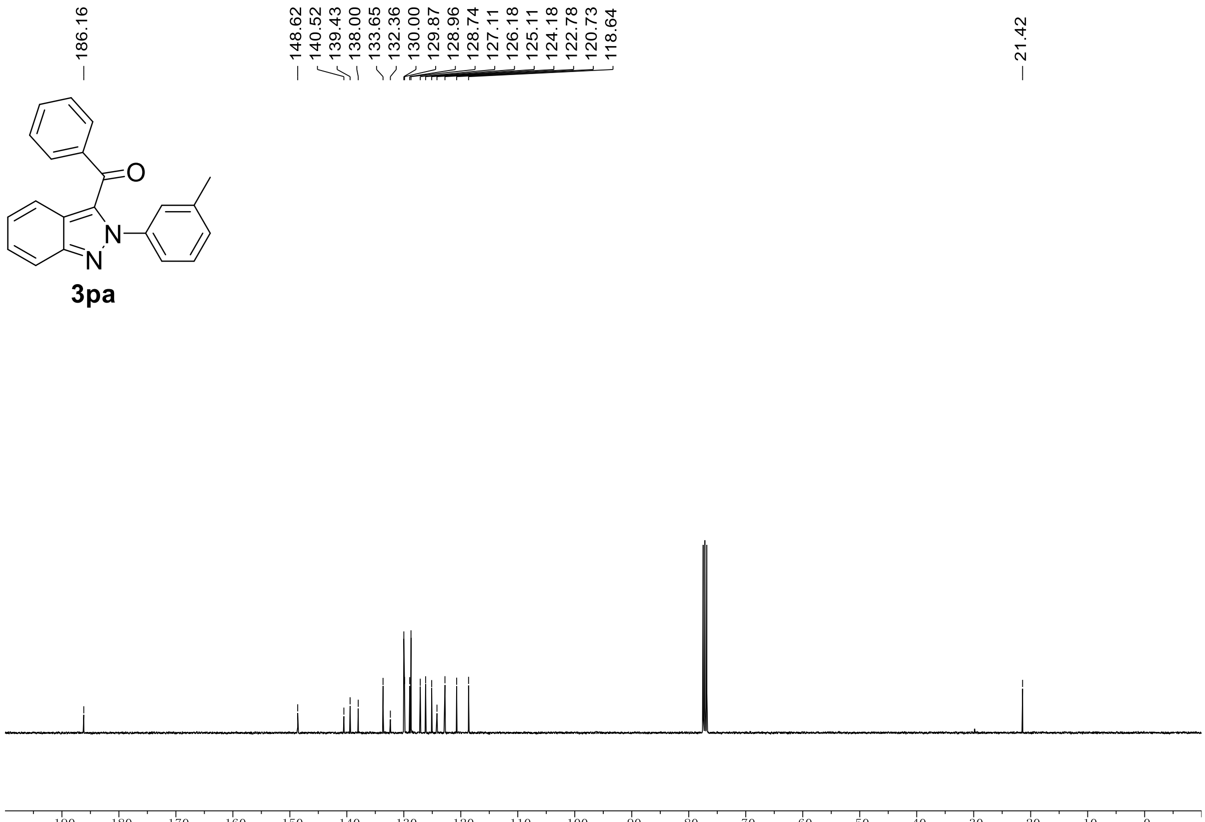

Figure S39 :^{13}C {^{1}H} NMR spectrum of **3pa** (100 MHz, $CDCl_3$)

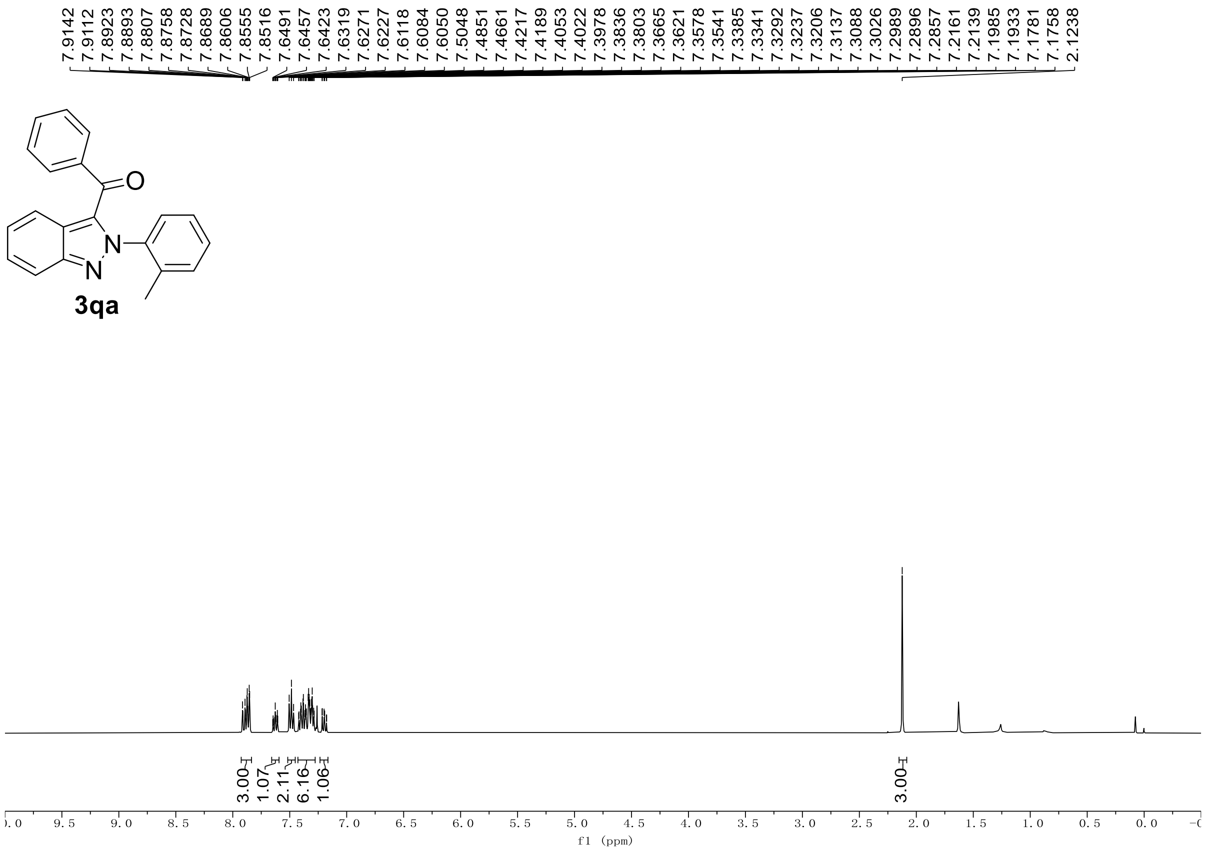

Figure S40 :^{1}H NMR spectrum of **3qa** (400 MHz, $CDCl_3$)

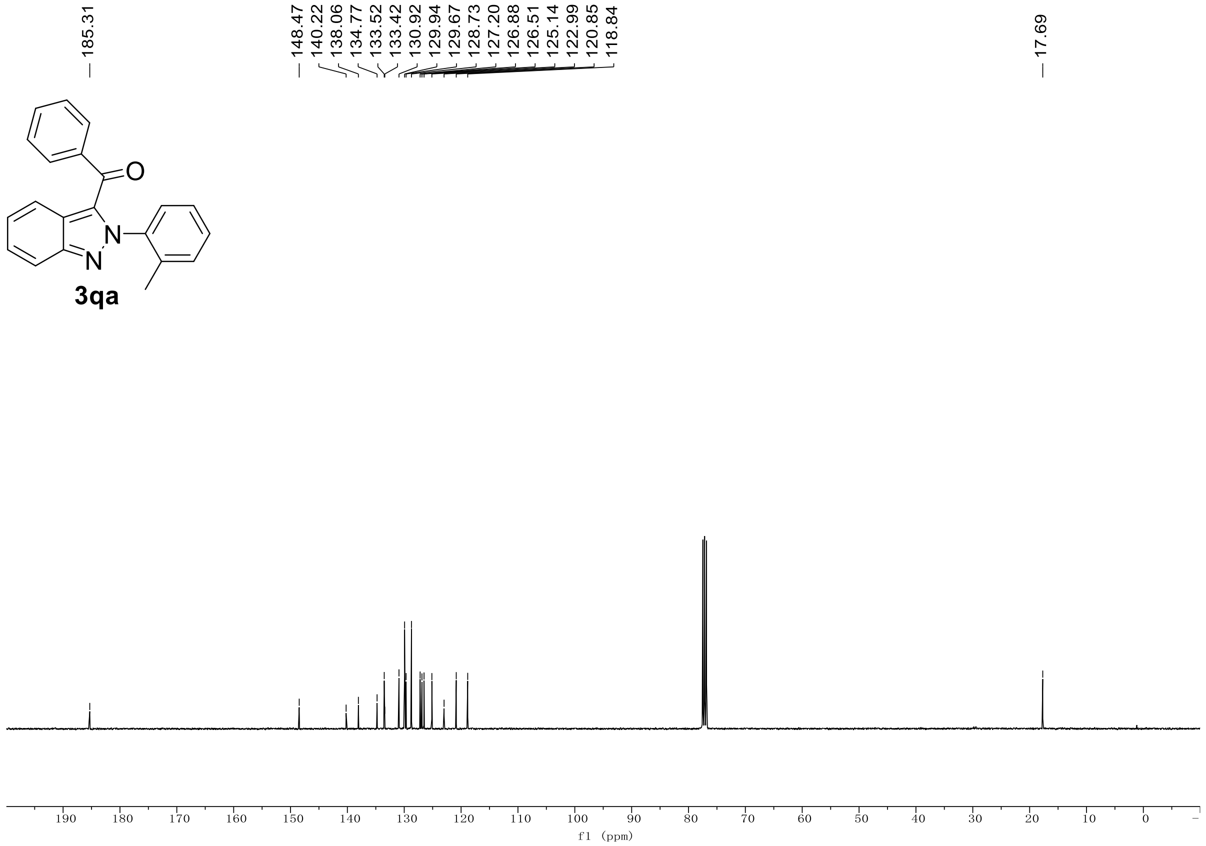

Figure S41 :^{13}C {^{1}H} NMR spectrum of **3qa** (100 MHz, $CDCl_3$)

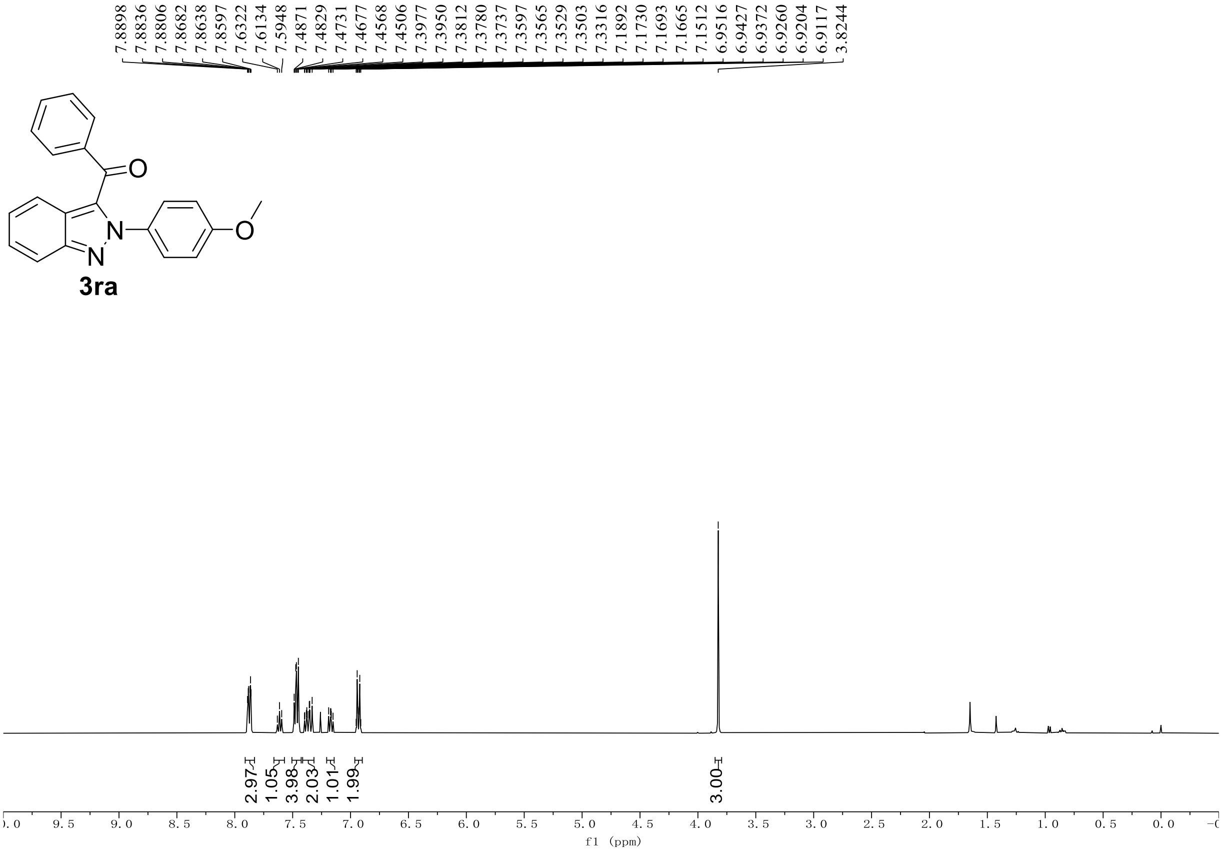

Figure S42 :^{1}H NMR spectrum of **3ra** (400 MHz, $CDCl_3$)

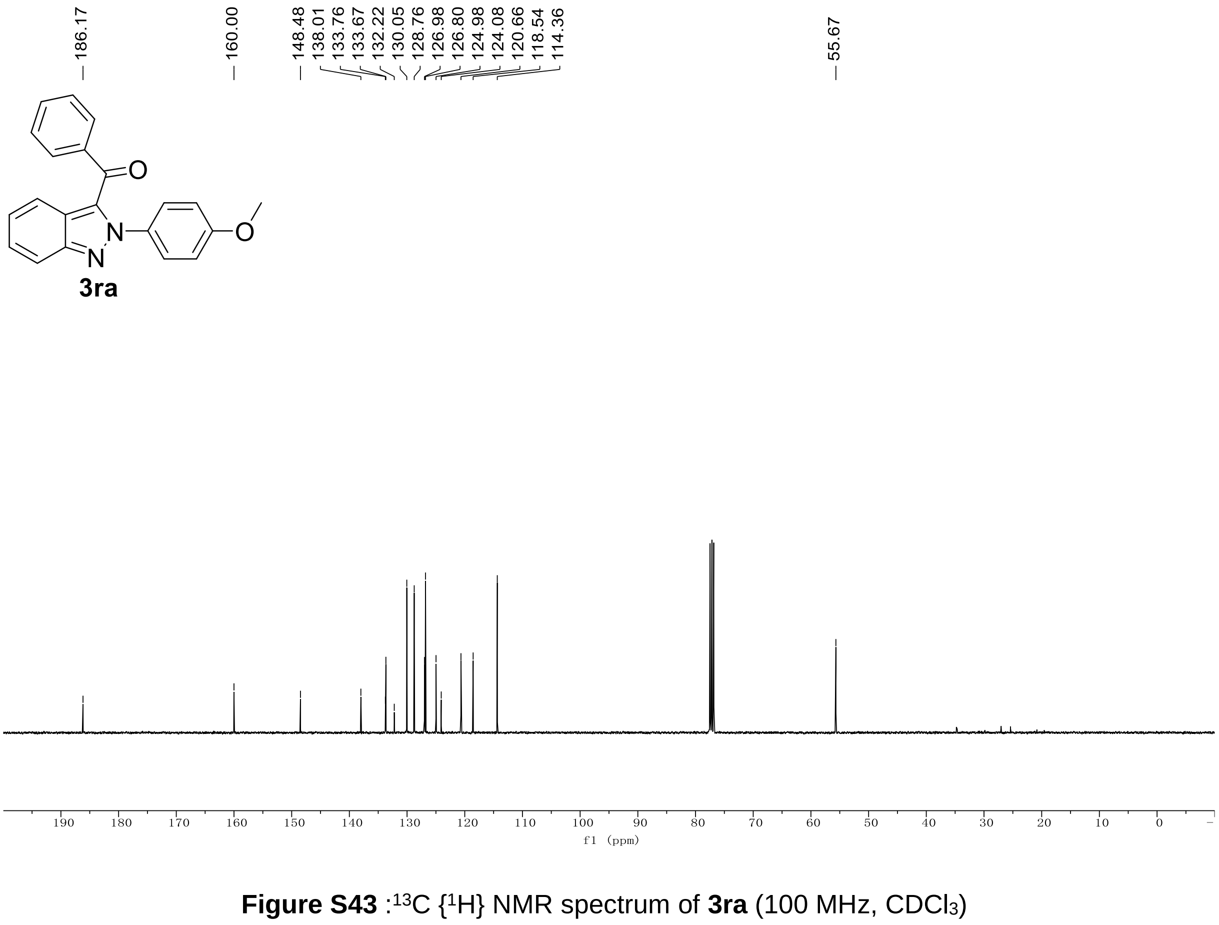

Figure S43 :^{13}C {^{1}H} NMR spectrum of **3ra** (100 MHz, $CDCl_3$)

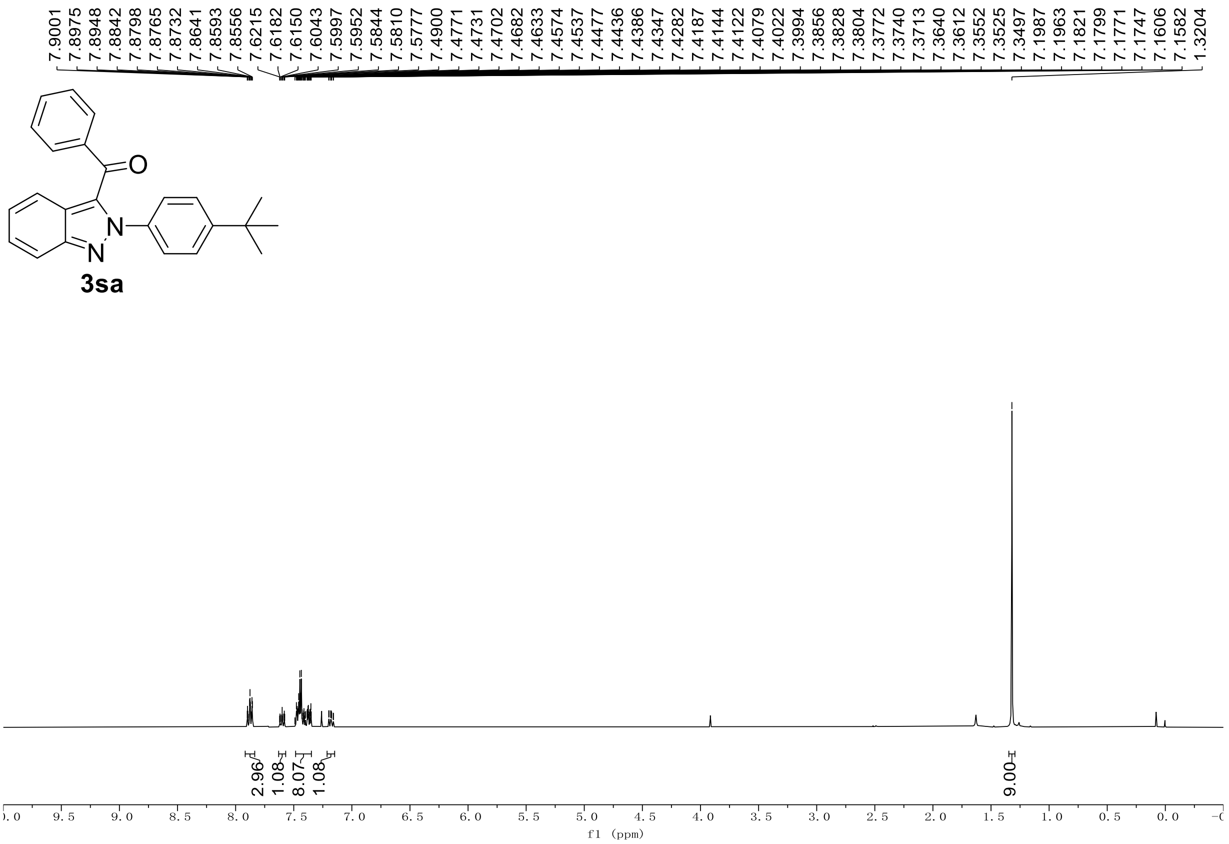

Figure S44 :^{1}H NMR spectrum of **3sa** (400 MHz, $CDCl_3$)

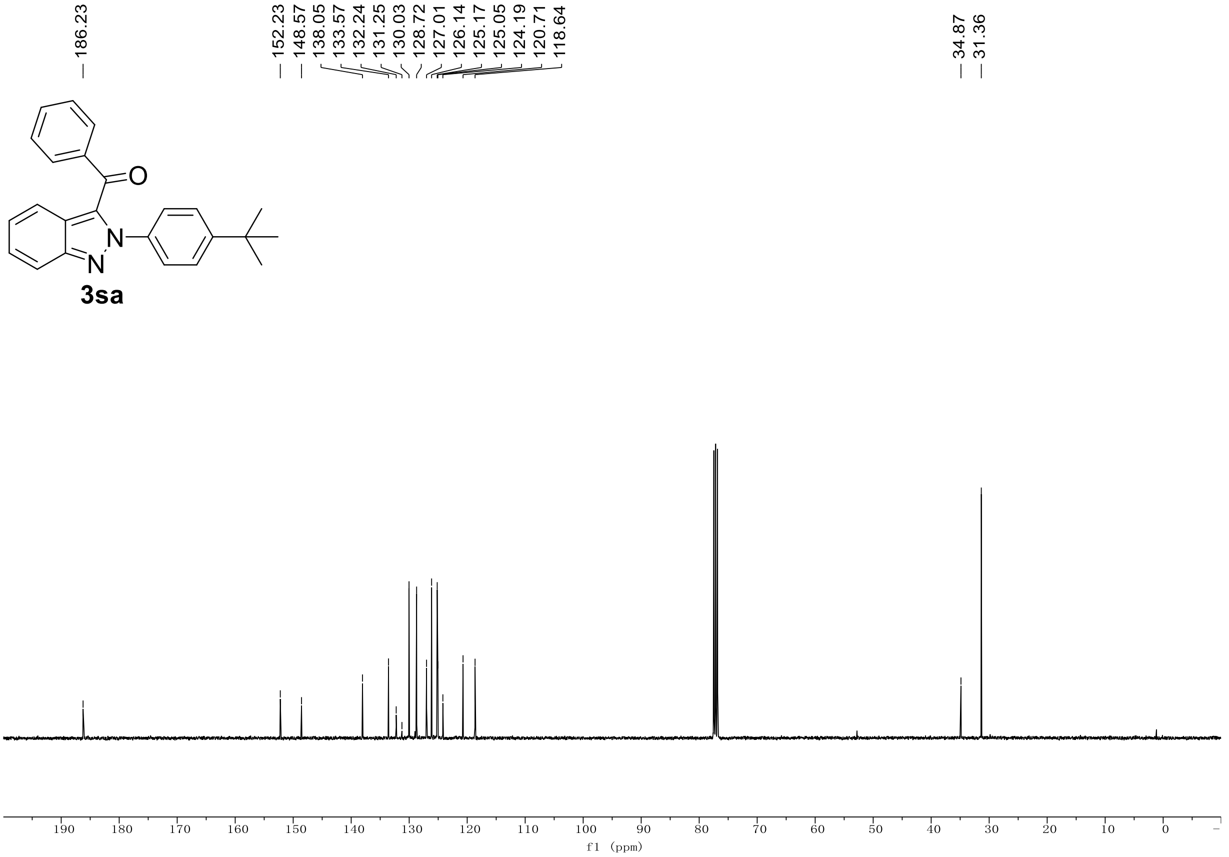

Figure S45 :^{13}C {^{1}H} NMR spectrum of **3sa** (100 MHz, $CDCl_3$)

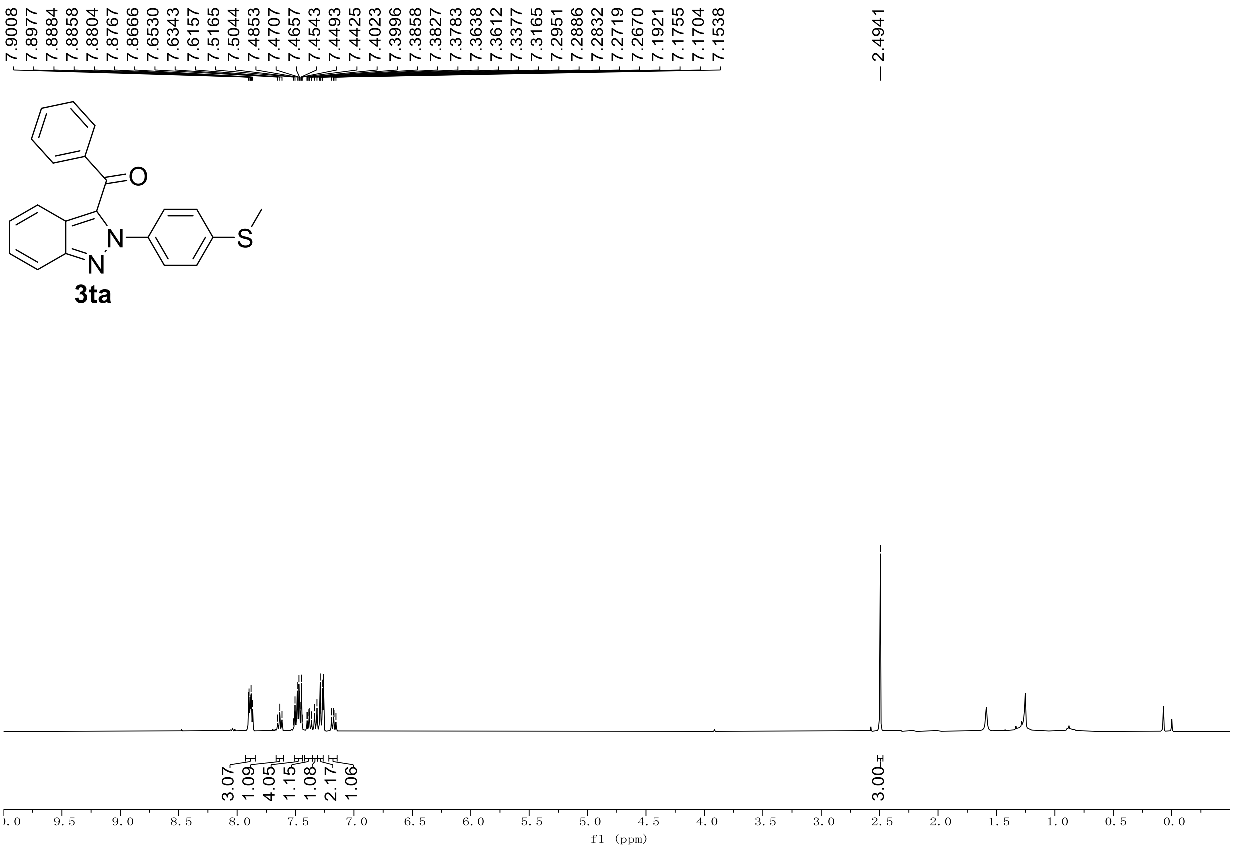

Figure S46 :^{1}H NMR spectrum of **3ta** (400 MHz, $CDCl_3$)

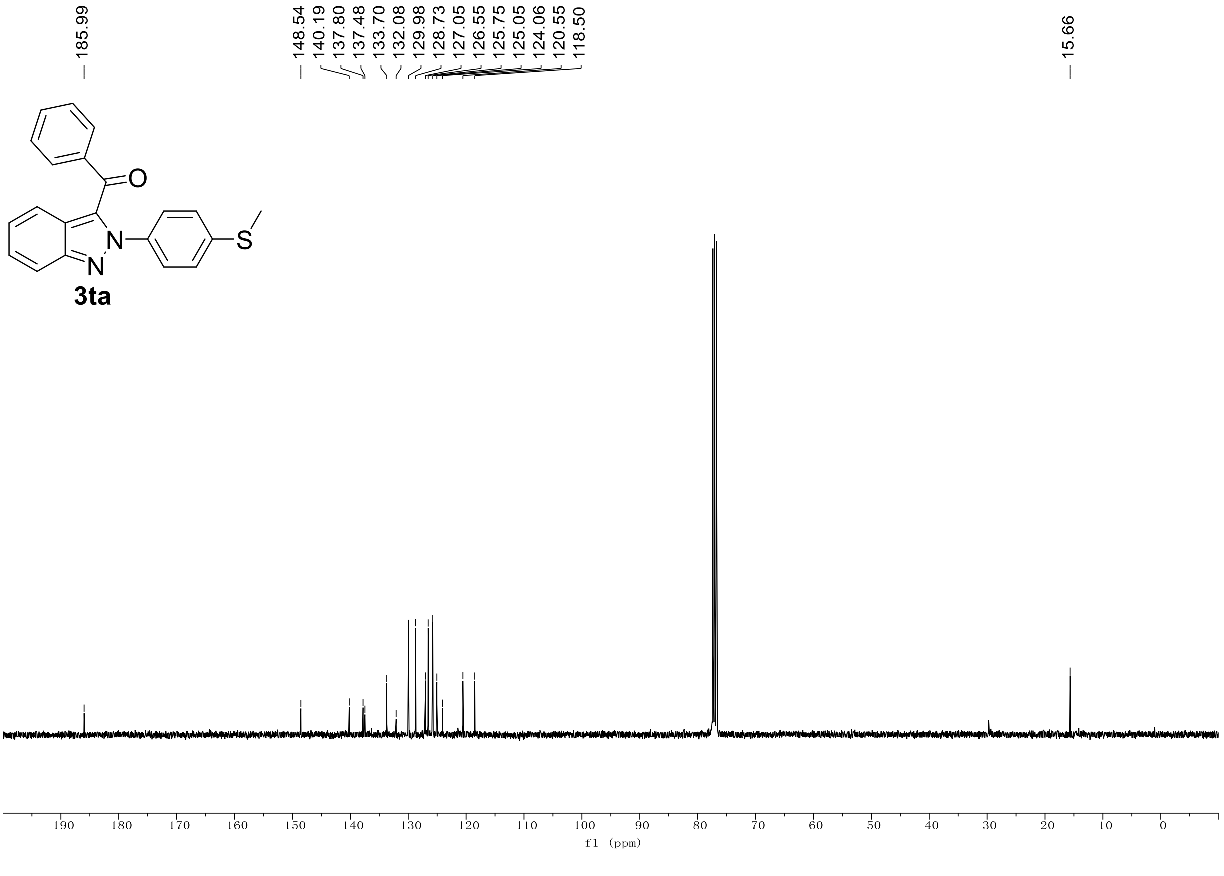

Figure S47 :^{13}C {^{1}H} NMR spectrum of **3ta** (100 MHz, $CDCl_3$)

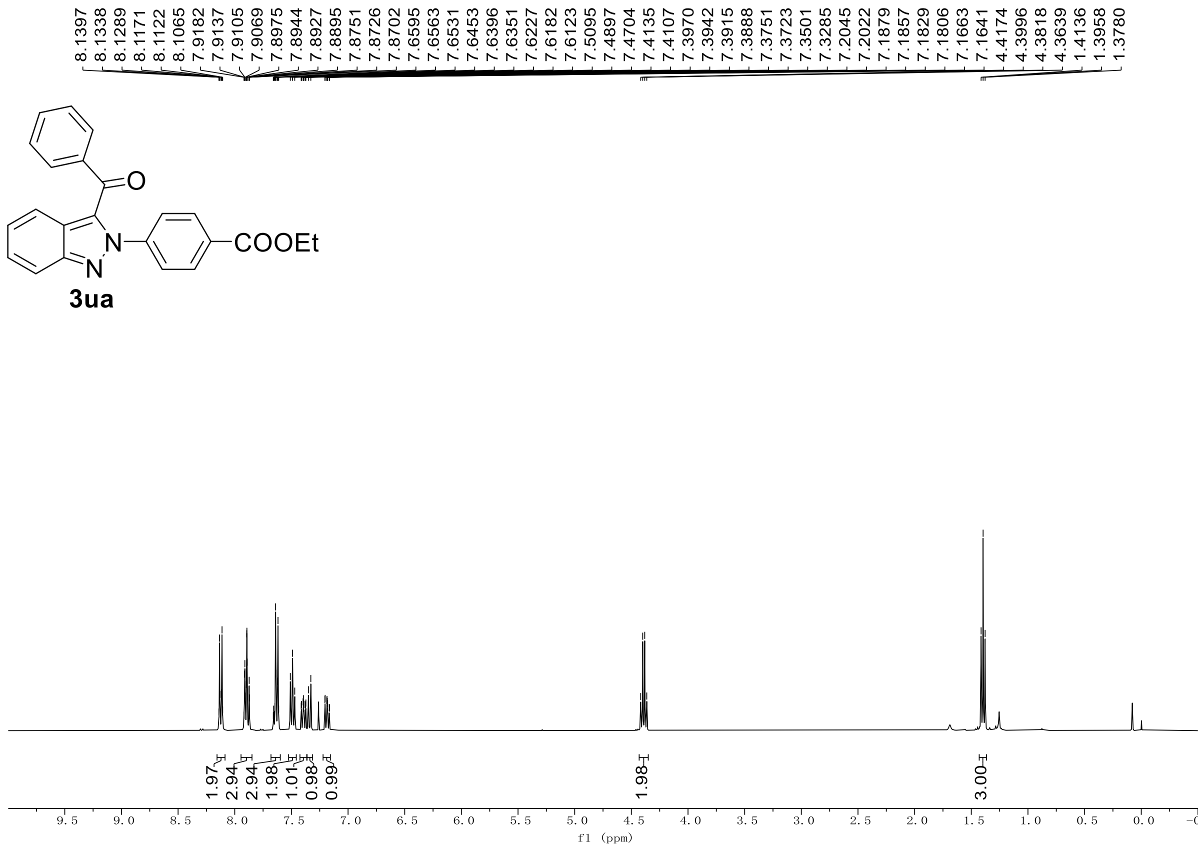

Figure S48 :^{1}H NMR spectrum of **3ua** (400 MHz, $CDCl_3$)

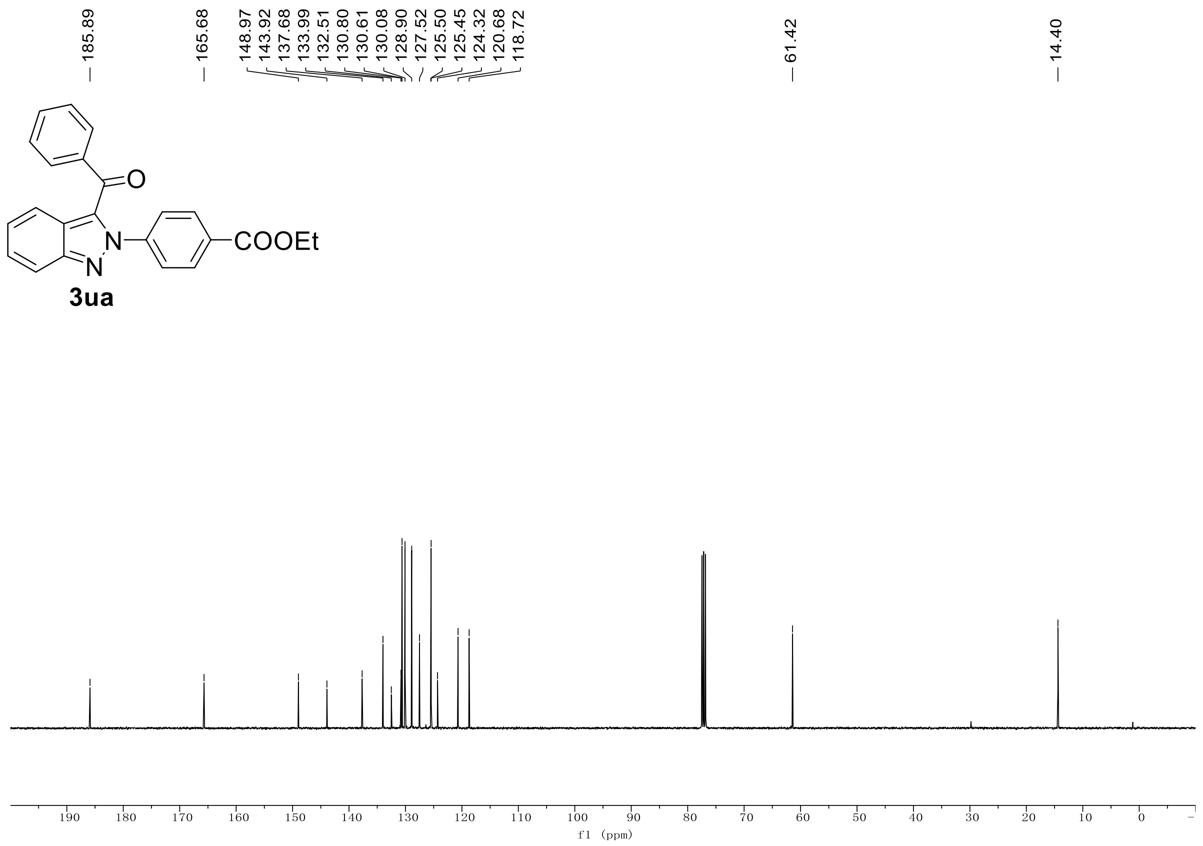

Figure S49 :^{13}C {^{1}H} NMR spectrum of **3ua** (100 MHz, $CDCl_3$)

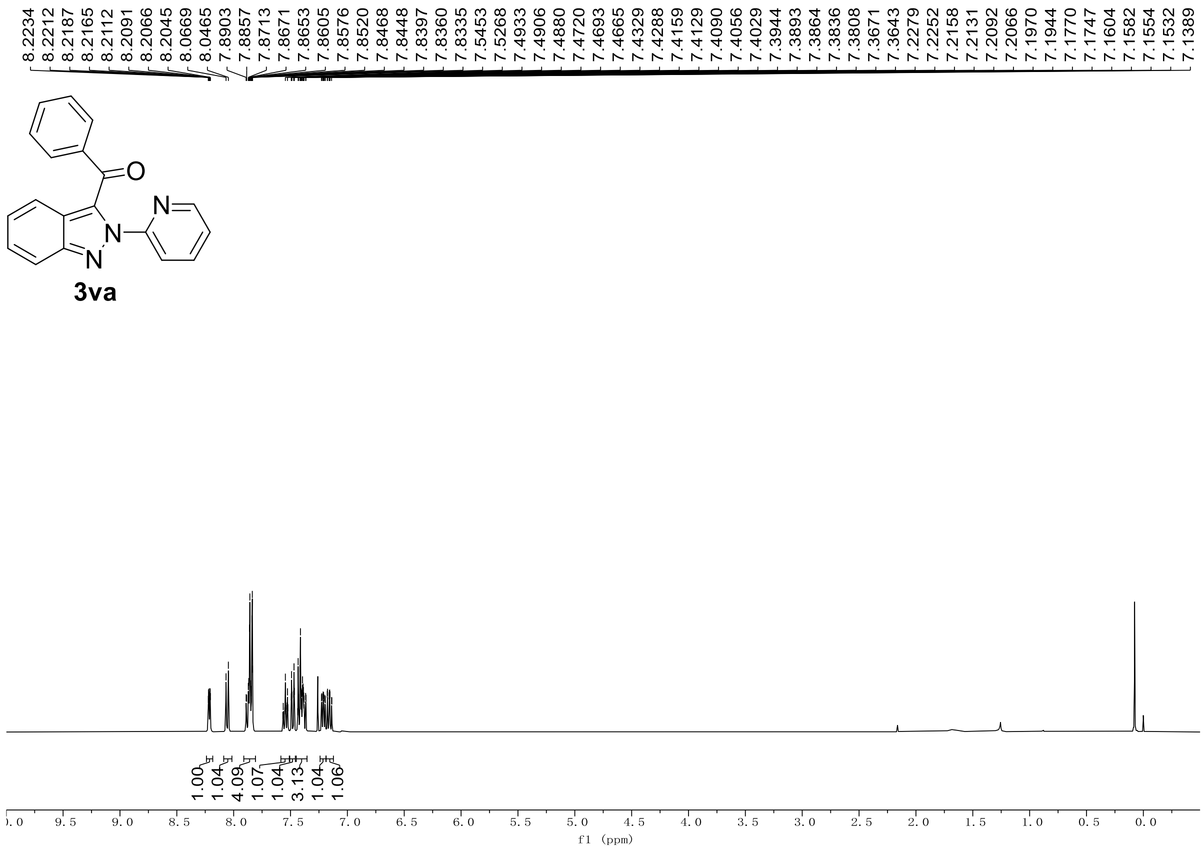

Figure S50 :^{1}H NMR spectrum of **3va** (400 MHz, $CDCl_3$)

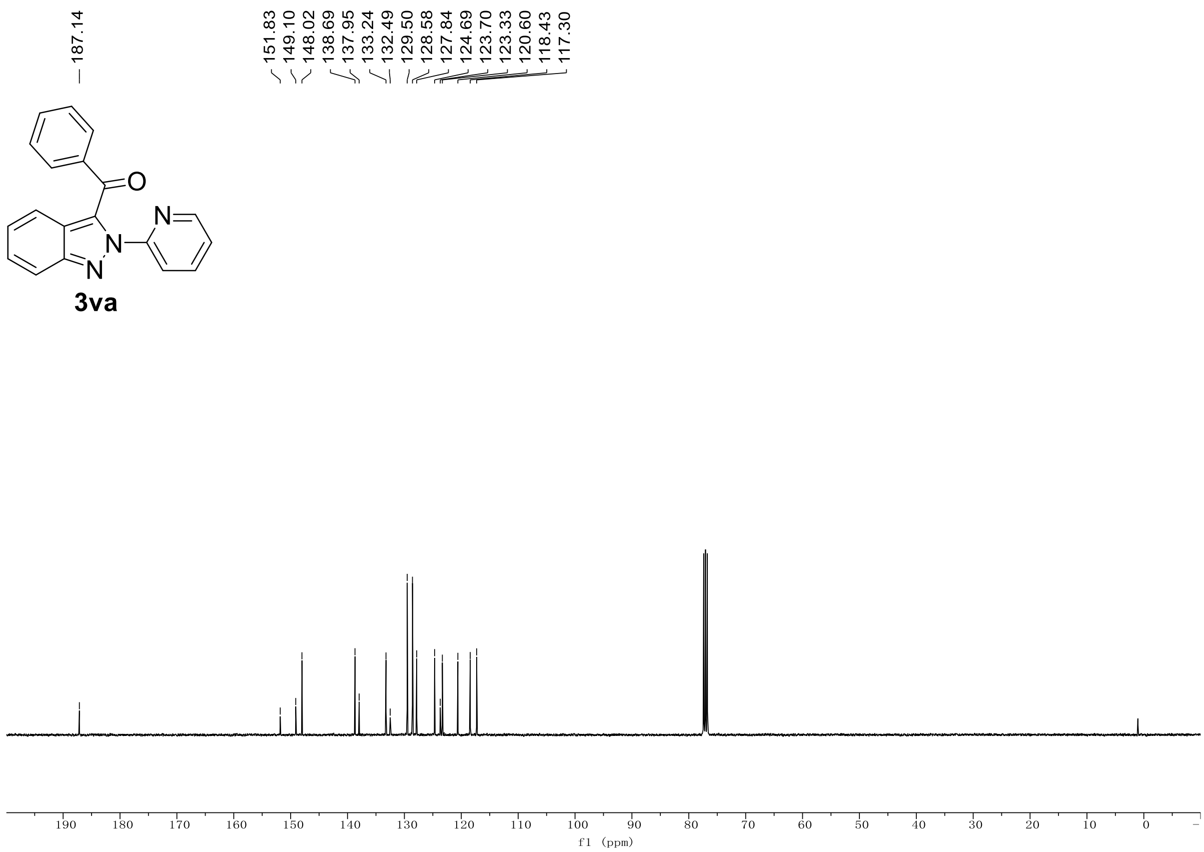

Figure S51 :^{13}C {^{1}H} NMR spectrum of **3va** (100 MHz, $CDCl_3$)

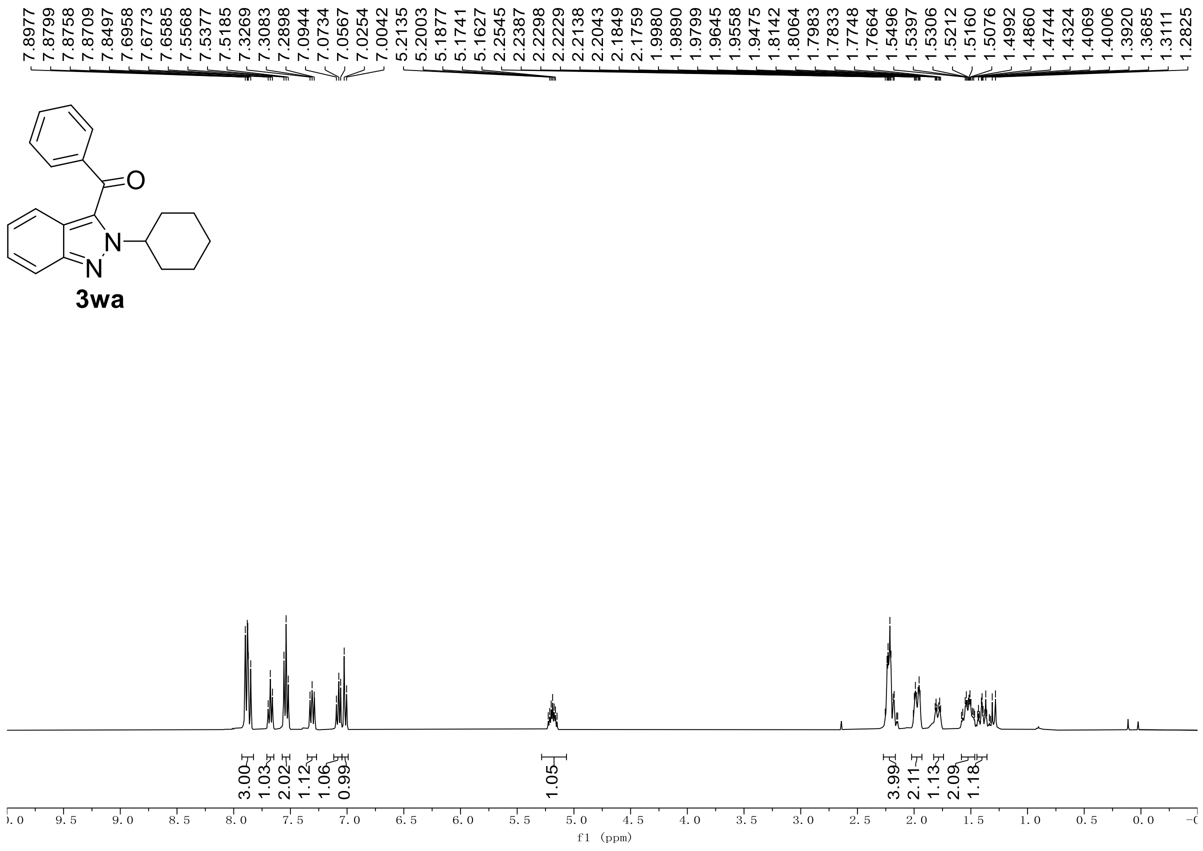

Figure S52 :^{1}H NMR spectrum of **3wa** (400 MHz, $CDCl_3$)

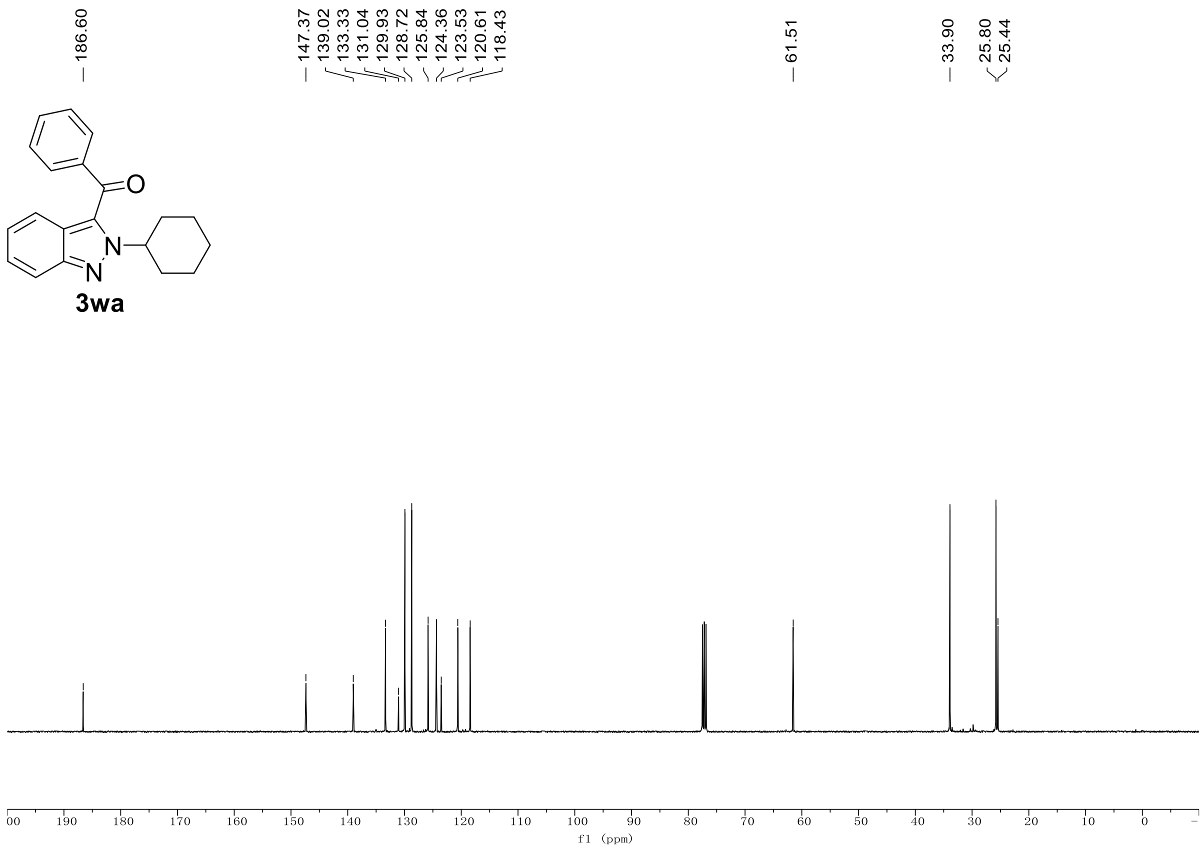

Figure S53 :^{13}C {^{1}H} NMR spectrum of **3wa** (100 MHz, $CDCl_3$)

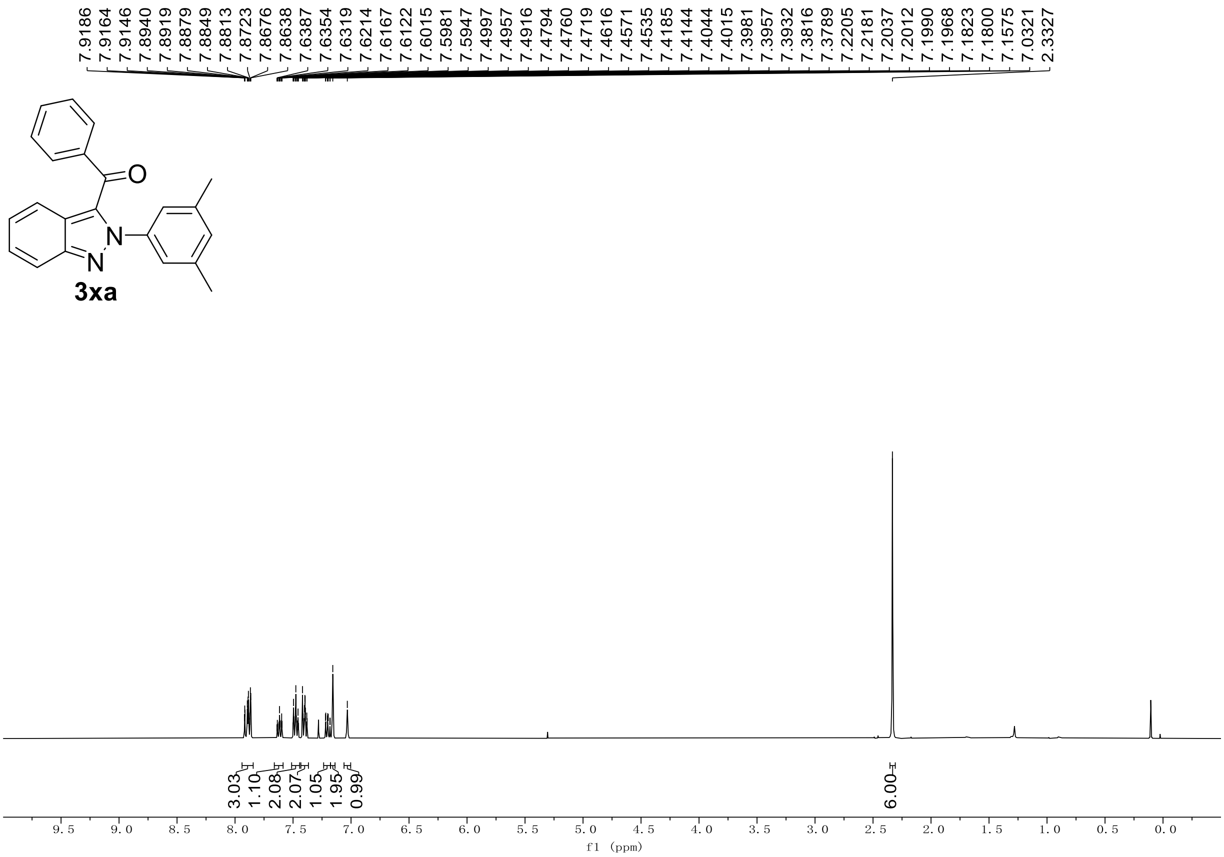

Figure S54 :^{1}H NMR spectrum of **3xa** (400 MHz, $CDCl_3$)

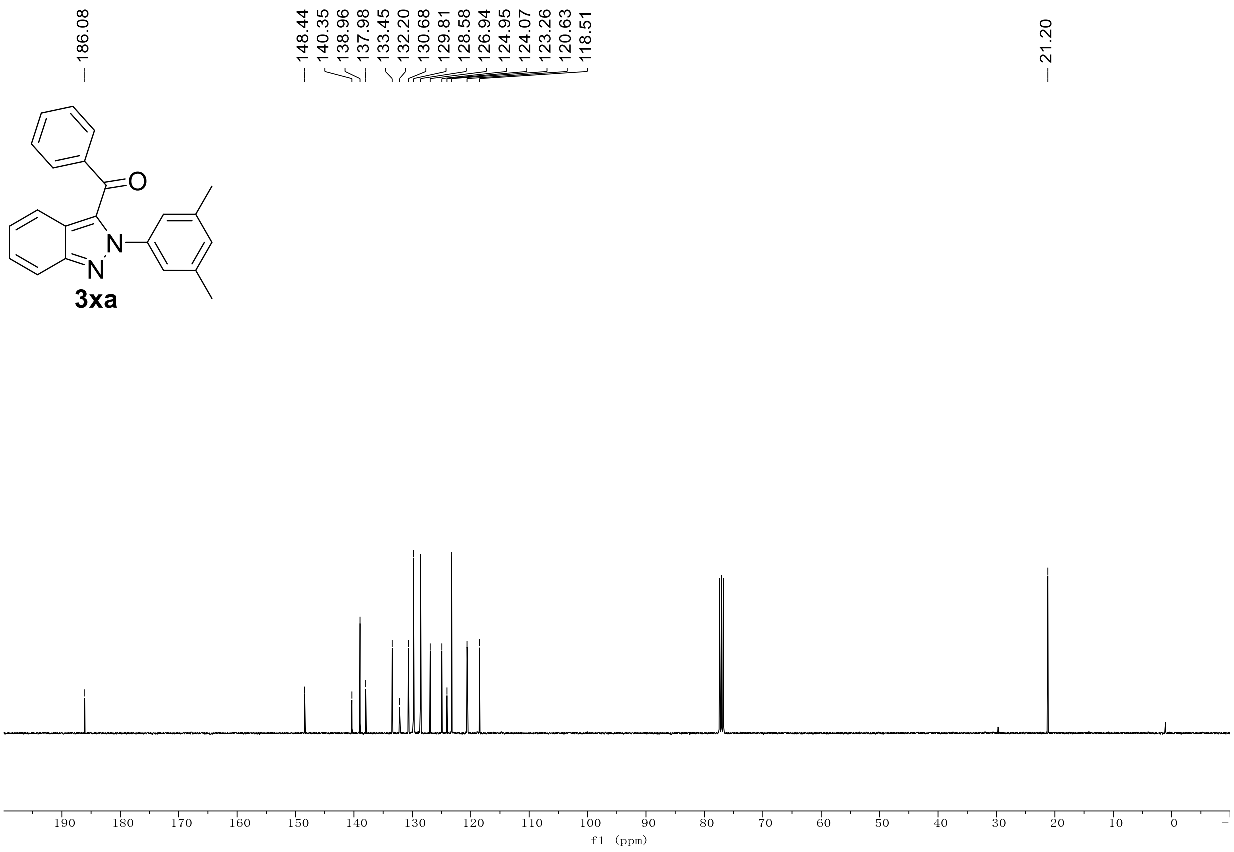

Figure S55 :^{13}C {^{1}H} NMR spectrum of **3xa** (100 MHz, $CDCl_3$)

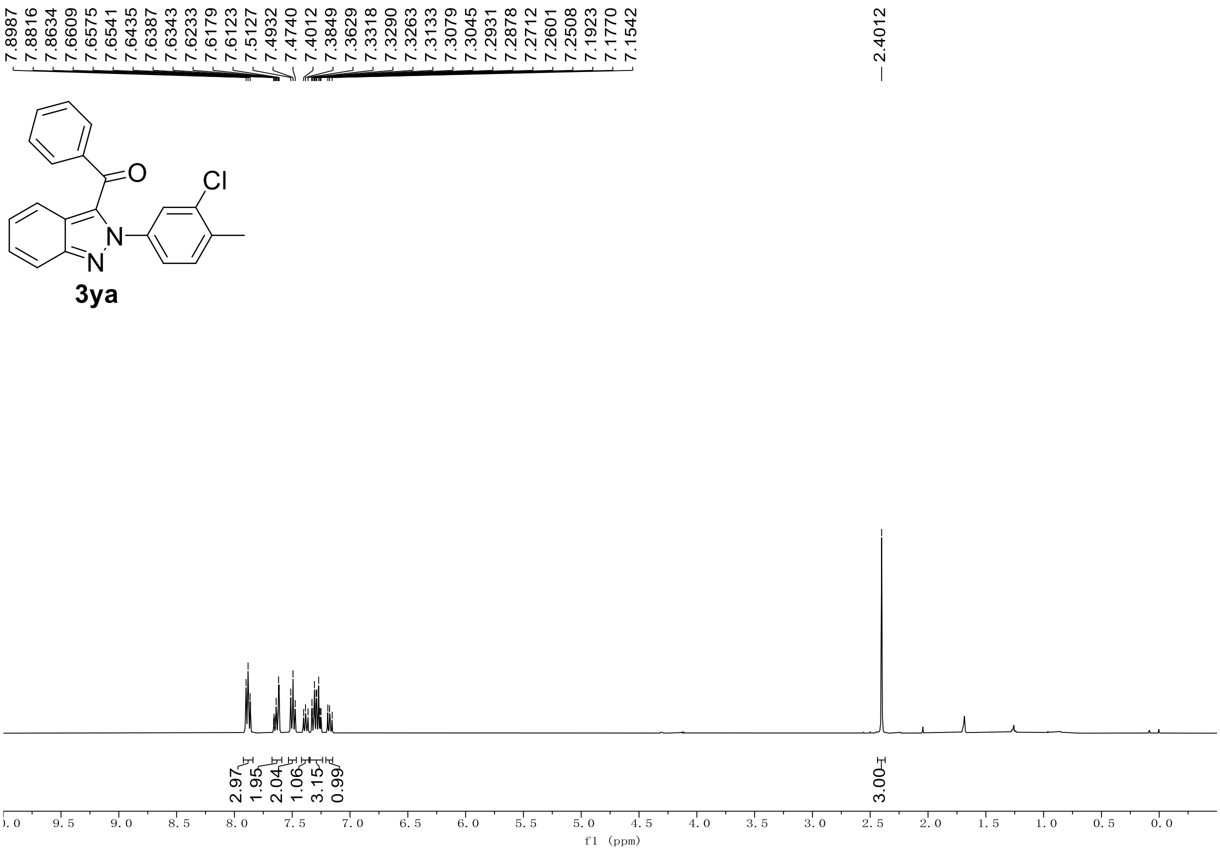

Figure S56 :^{1}H NMR spectrum of **3ya** (400 MHz, $CDCl_3$)

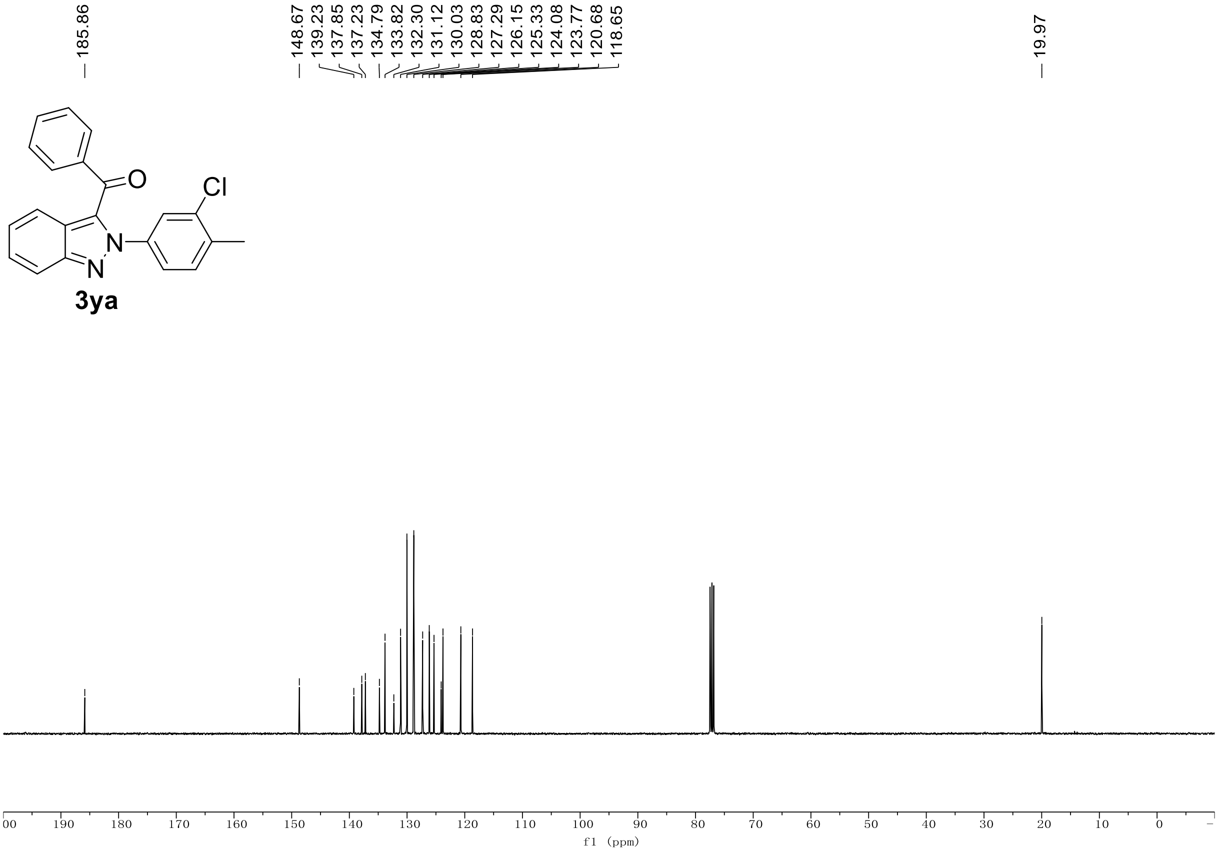

Figure S57 :^{13}C {^{1}H} NMR spectrum of **3ya** (100 MHz, $CDCl_3$)

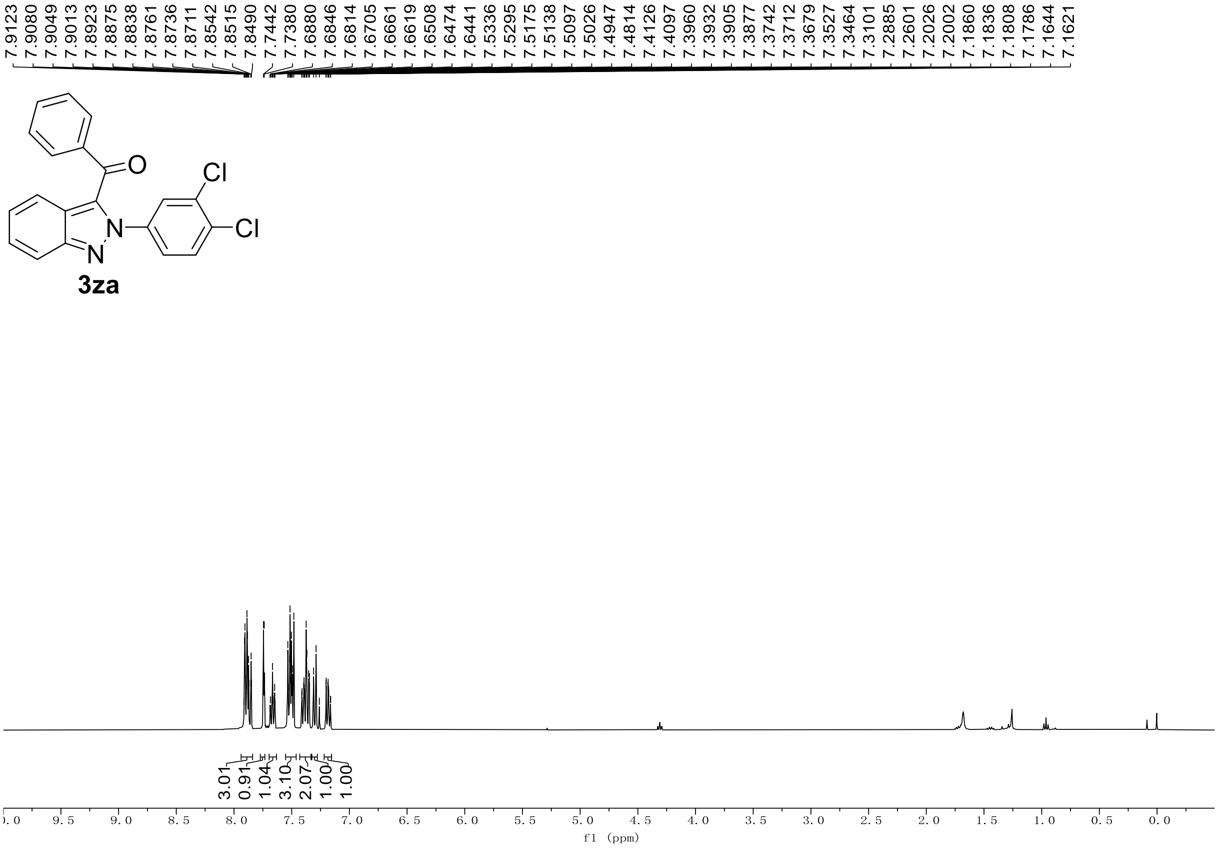

Figure S58 :^{1}H NMR spectrum of **3za** (400 MHz, $CDCl_3$)

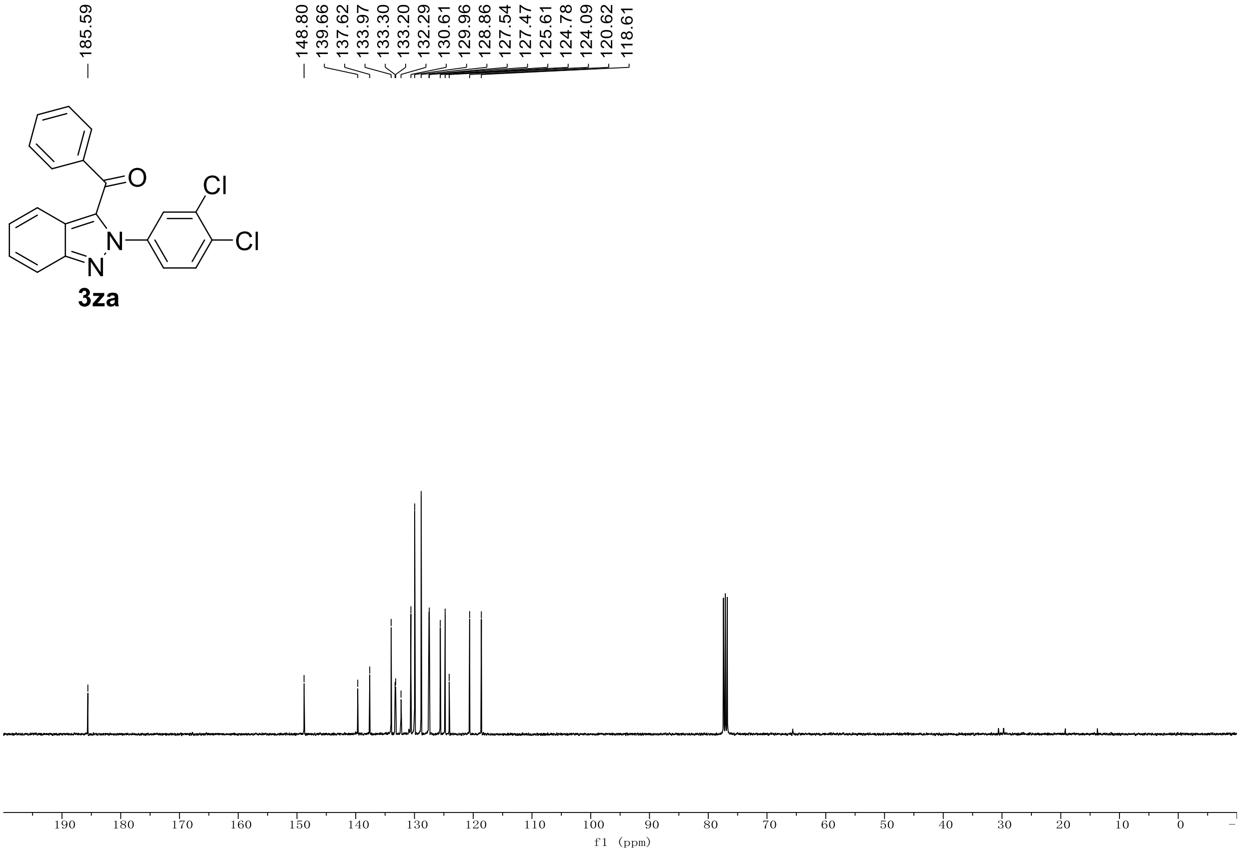

Figure S59 :^{13}C {^{1}H} NMR spectrum of **3za** (100 MHz, $CDCl_3$)

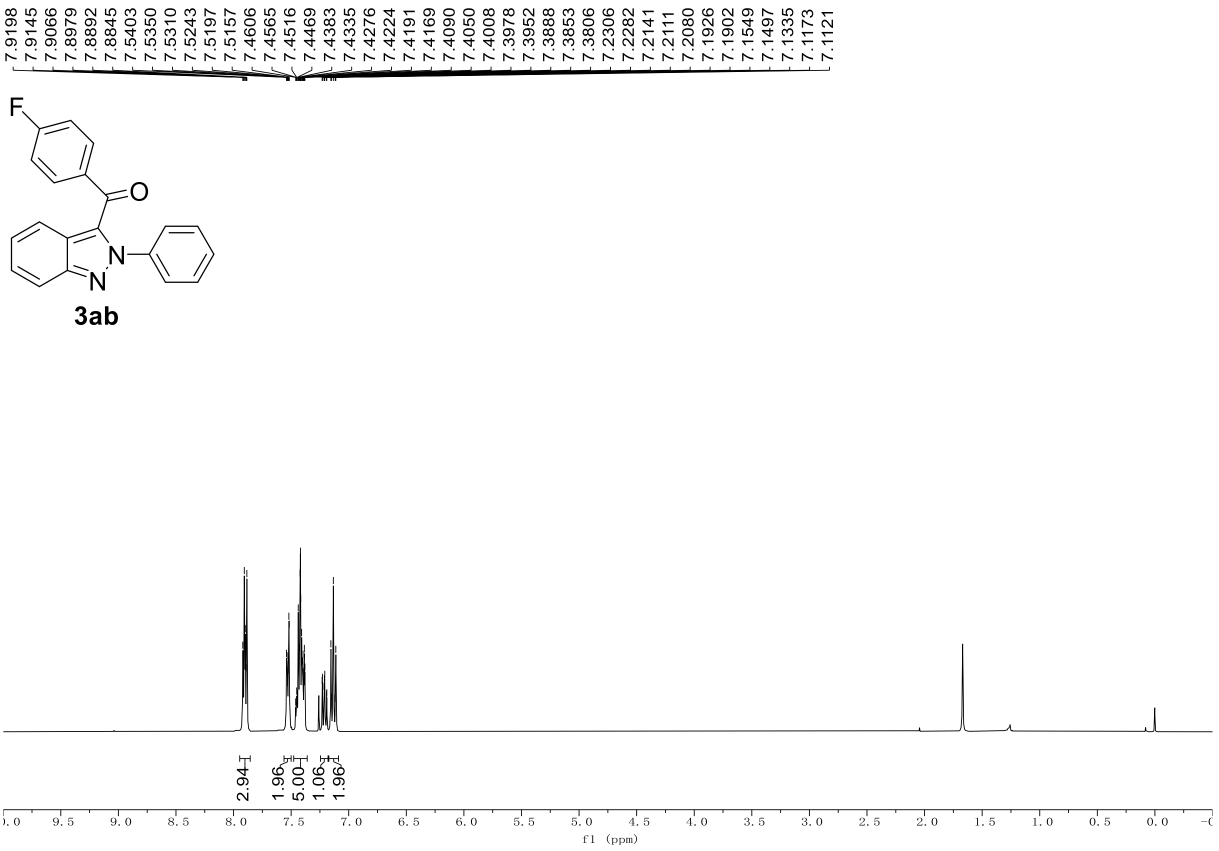

Figure S60 :^{1}H NMR spectrum of **3ab** (400 MHz, $CDCl_3$)

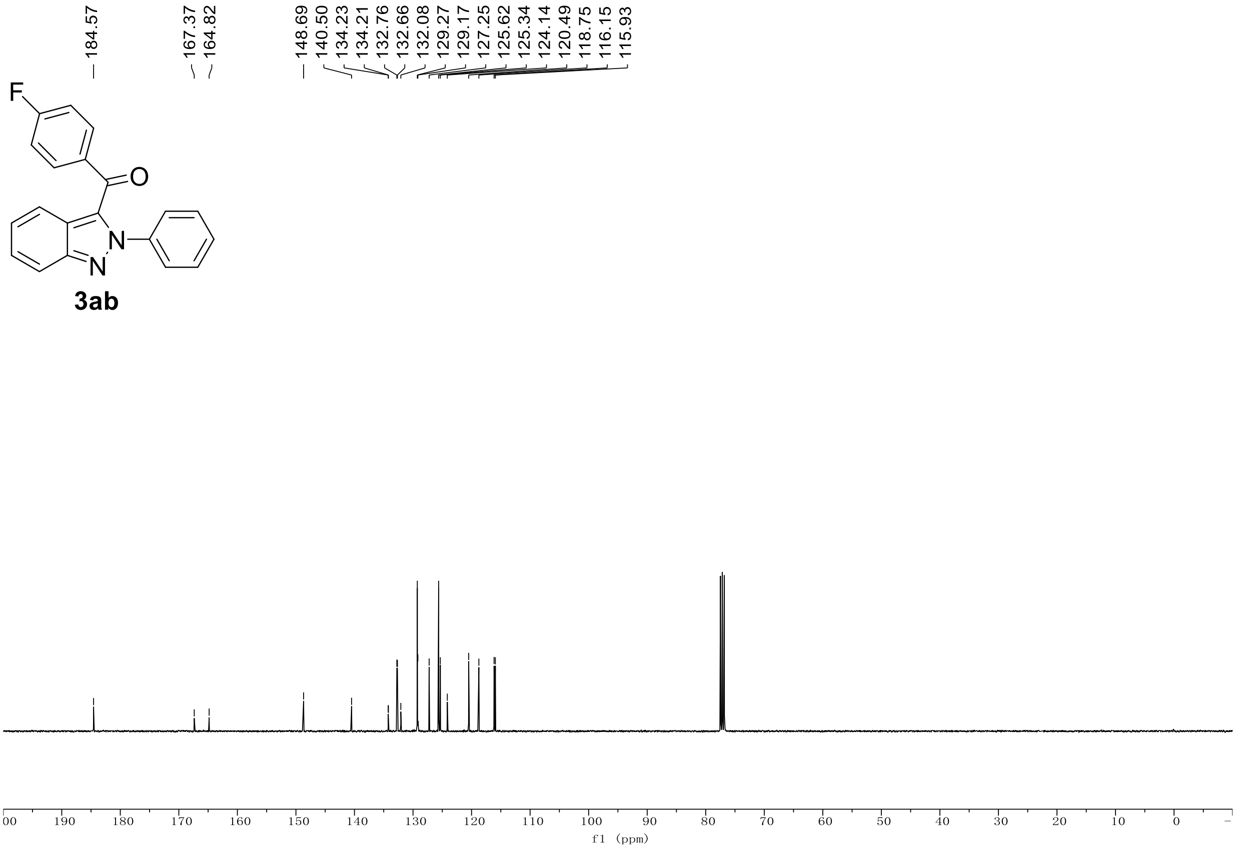

Figure S61 :^{13}C {^{1}H} NMR spectrum of **3ab** (100 MHz, $CDCl_3$)

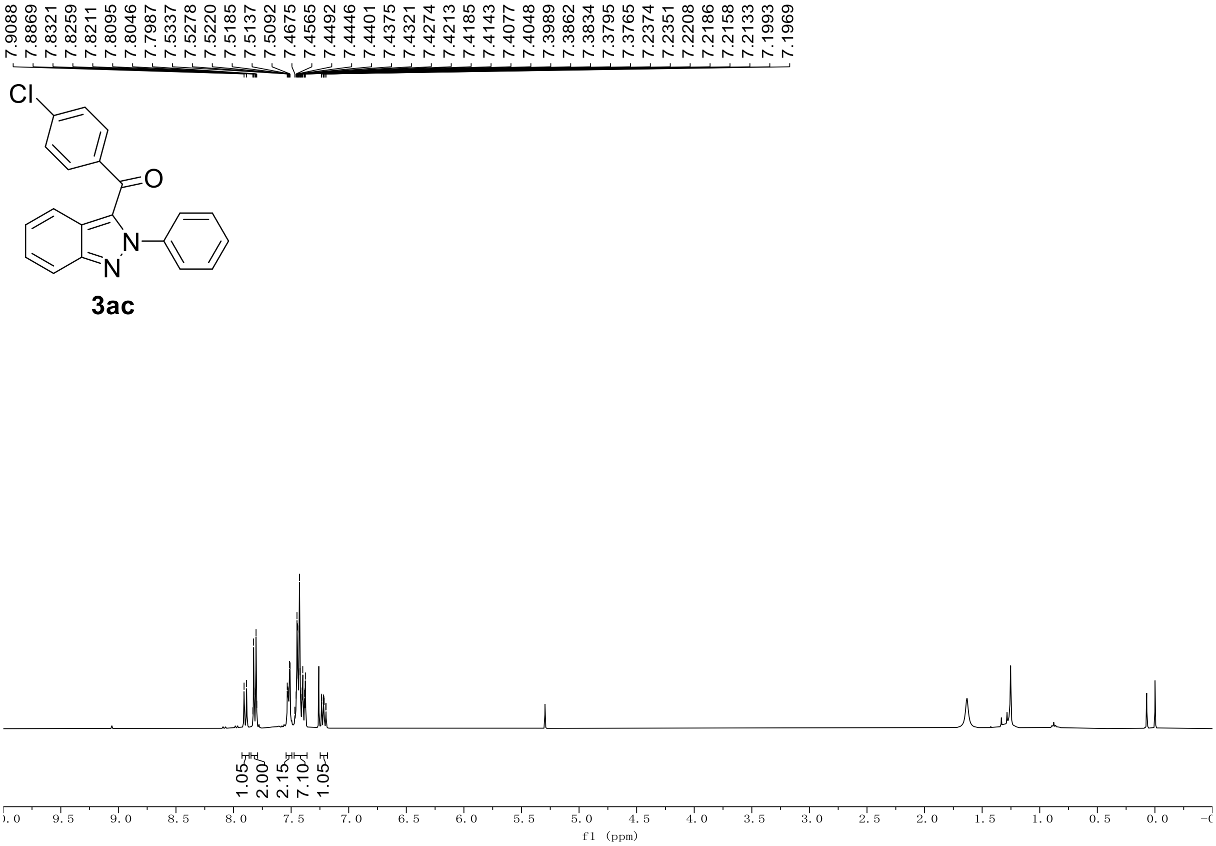

Figure S62 :^{1}H NMR spectrum of **3ac** (400 MHz, $CDCl_3$)

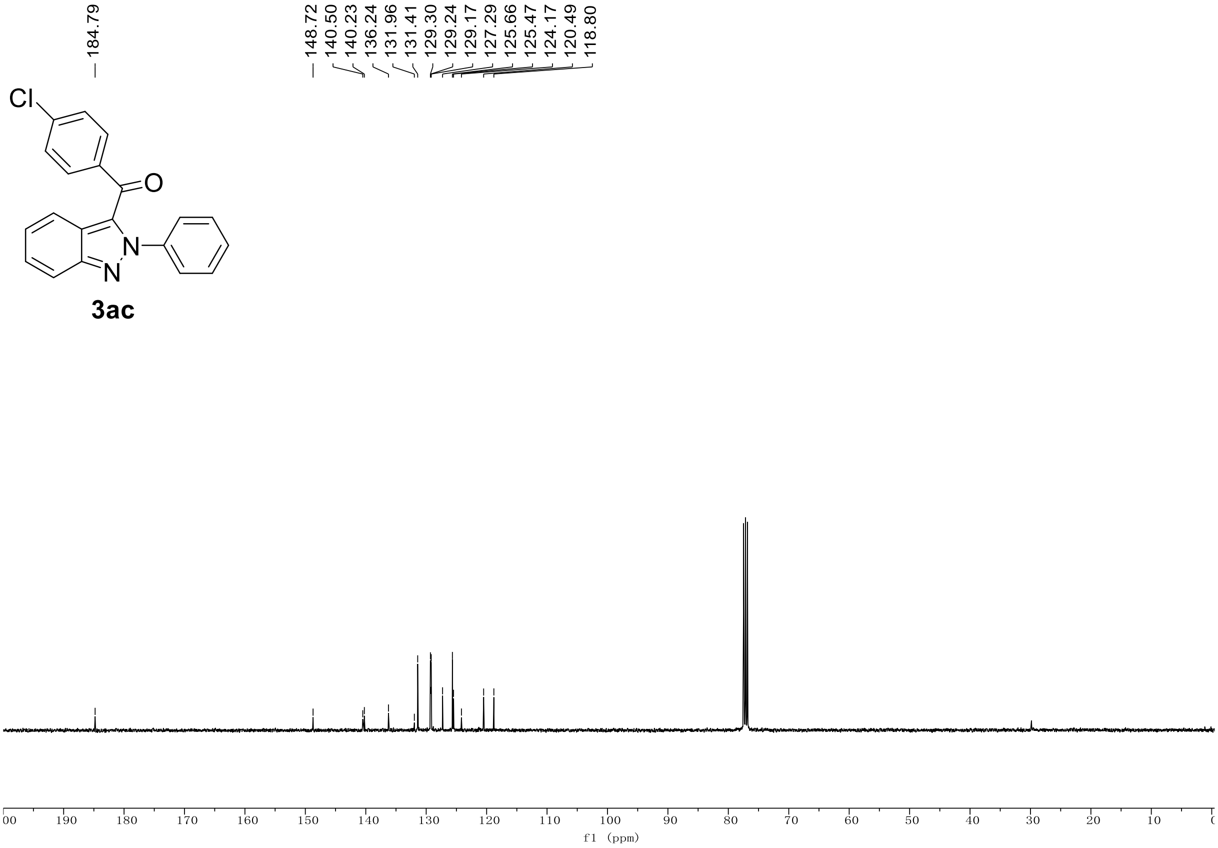

Figure S63 :^{13}C {^{1}H} NMR spectrum of **3ac** (100 MHz, $CDCl_3$)

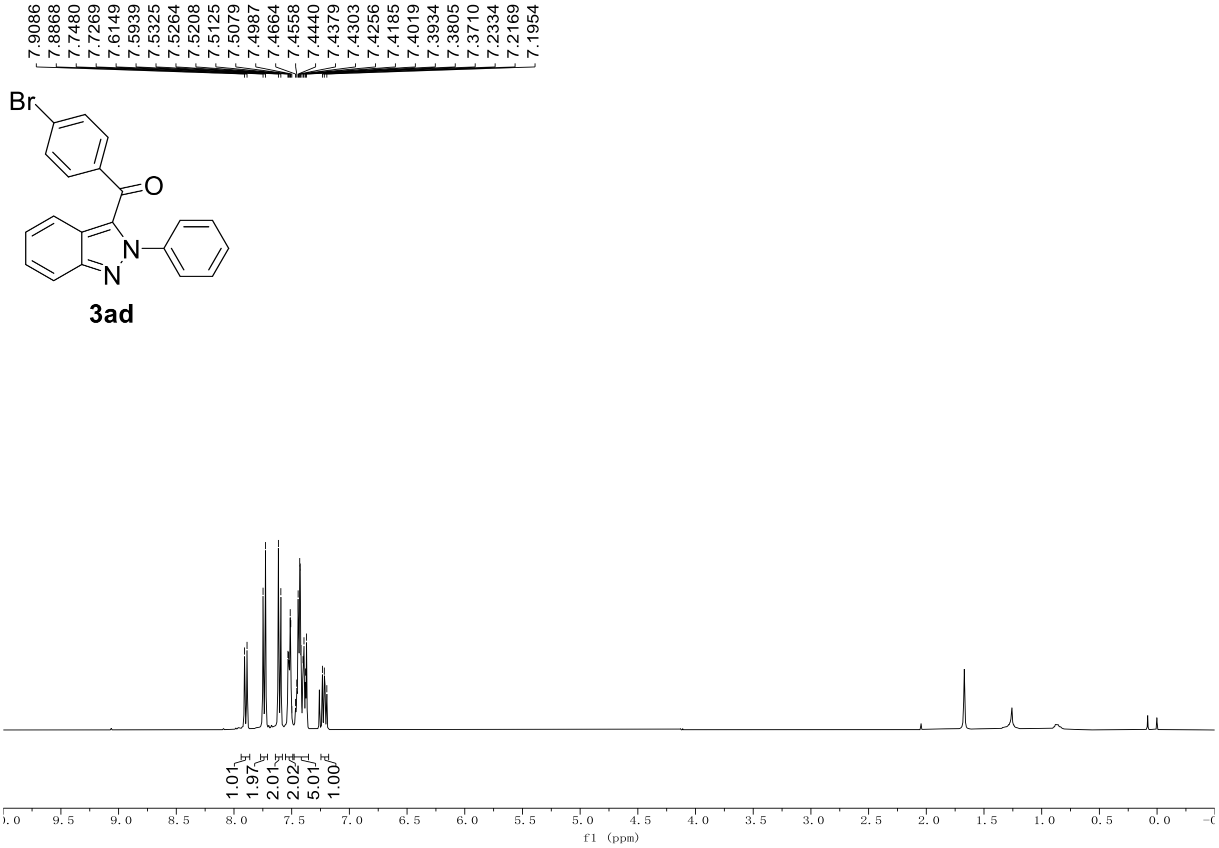

Figure S64 :^{1}H NMR spectrum of **3ad** (400 MHz, $CDCl_3$)

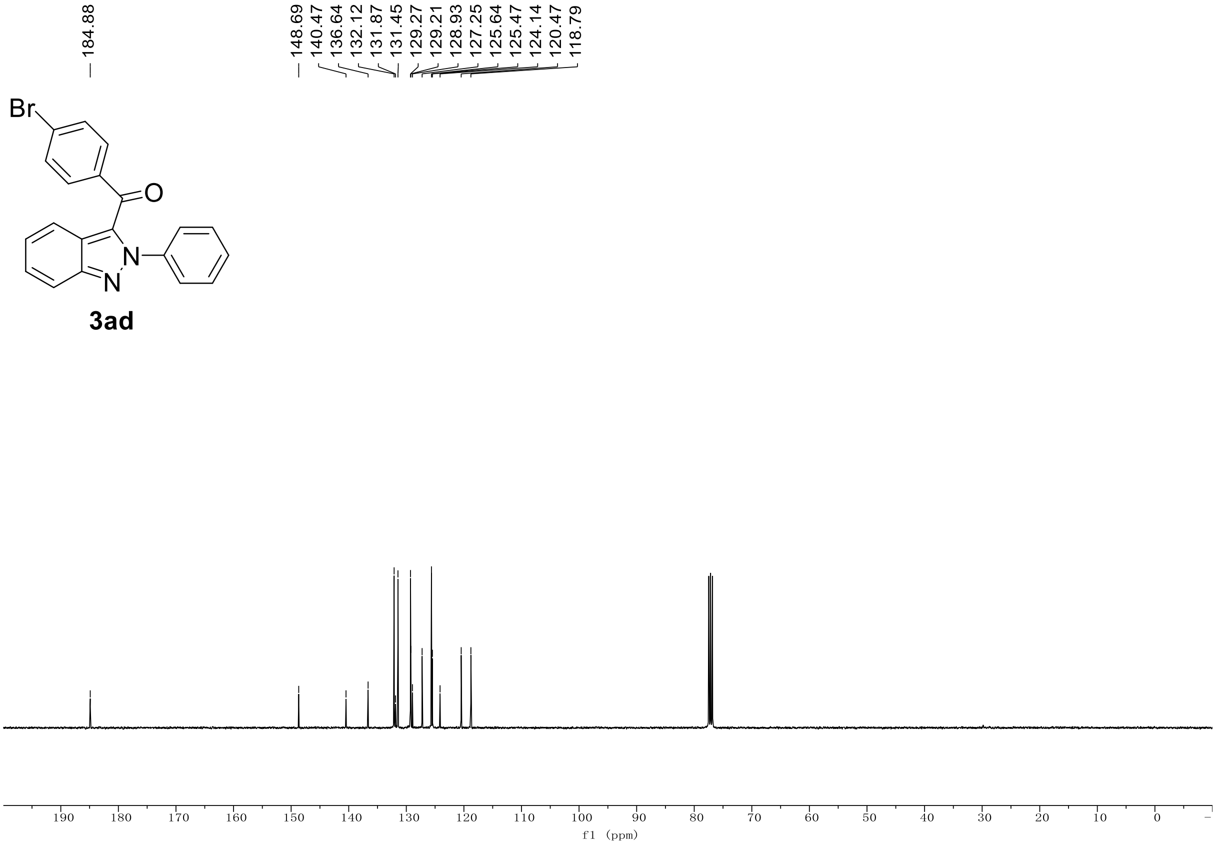

Figure S65 :^{13}C {^{1}H} NMR spectrum of **3ad** (100 MHz, $CDCl_3$)

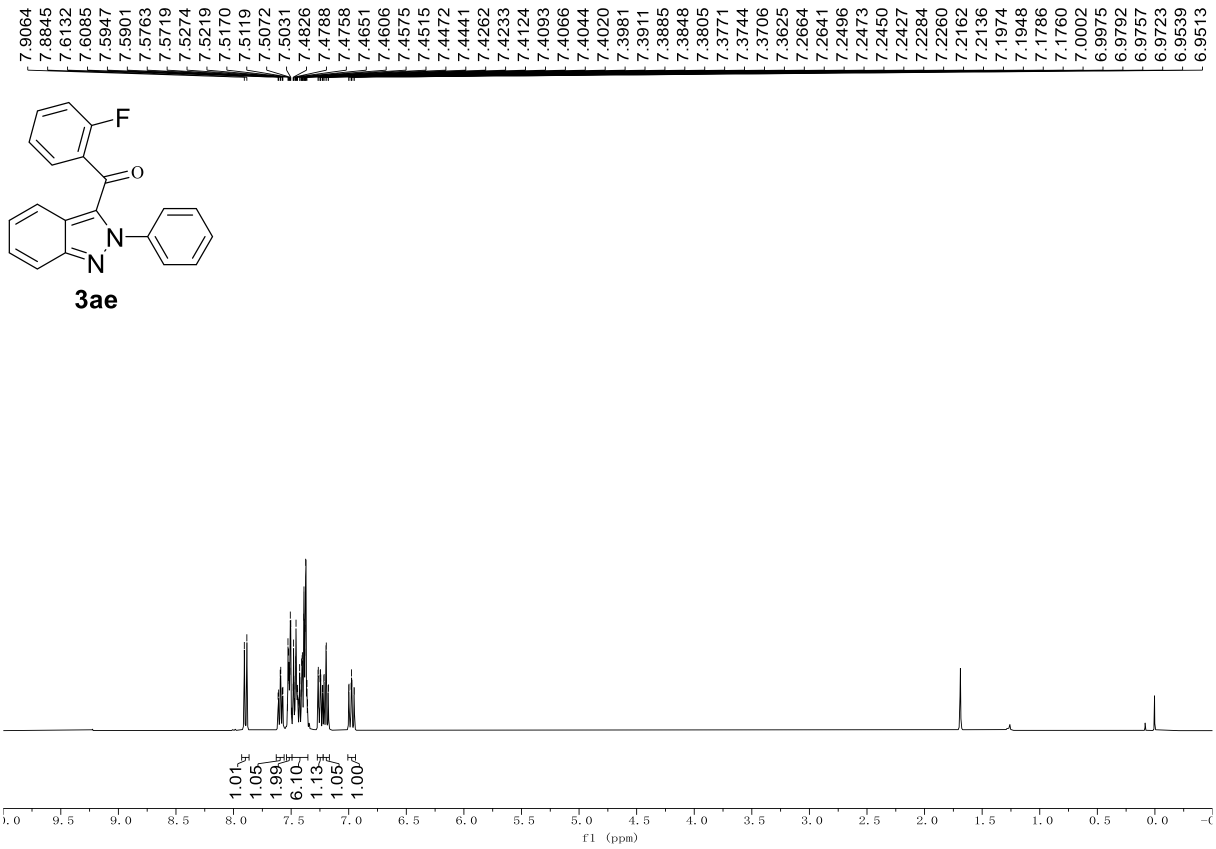

Figure S66 :^{1}H NMR spectrum of **3ae** (400 MHz, $CDCl_3$)

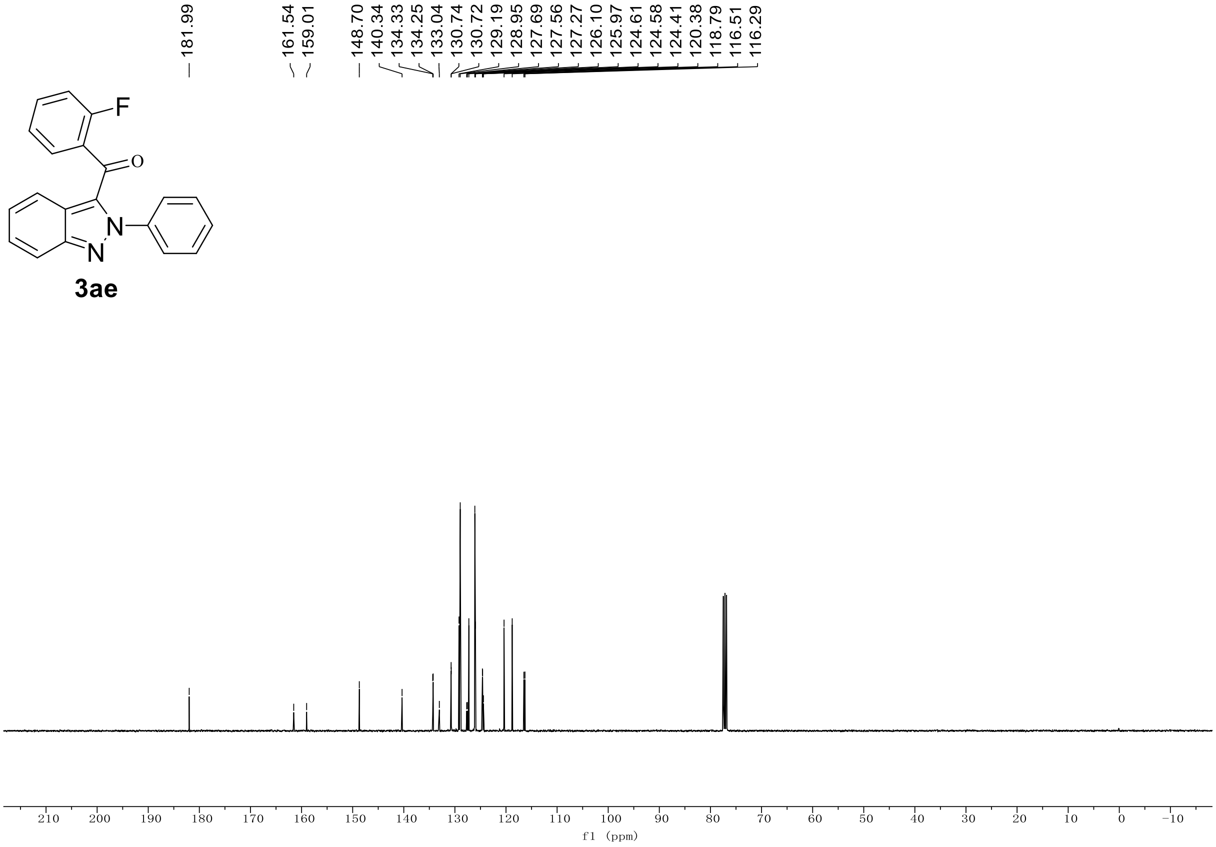

Figure S67 :^{13}C {^{1}H} NMR spectrum of **3ae** (100 MHz, $CDCl_3$)

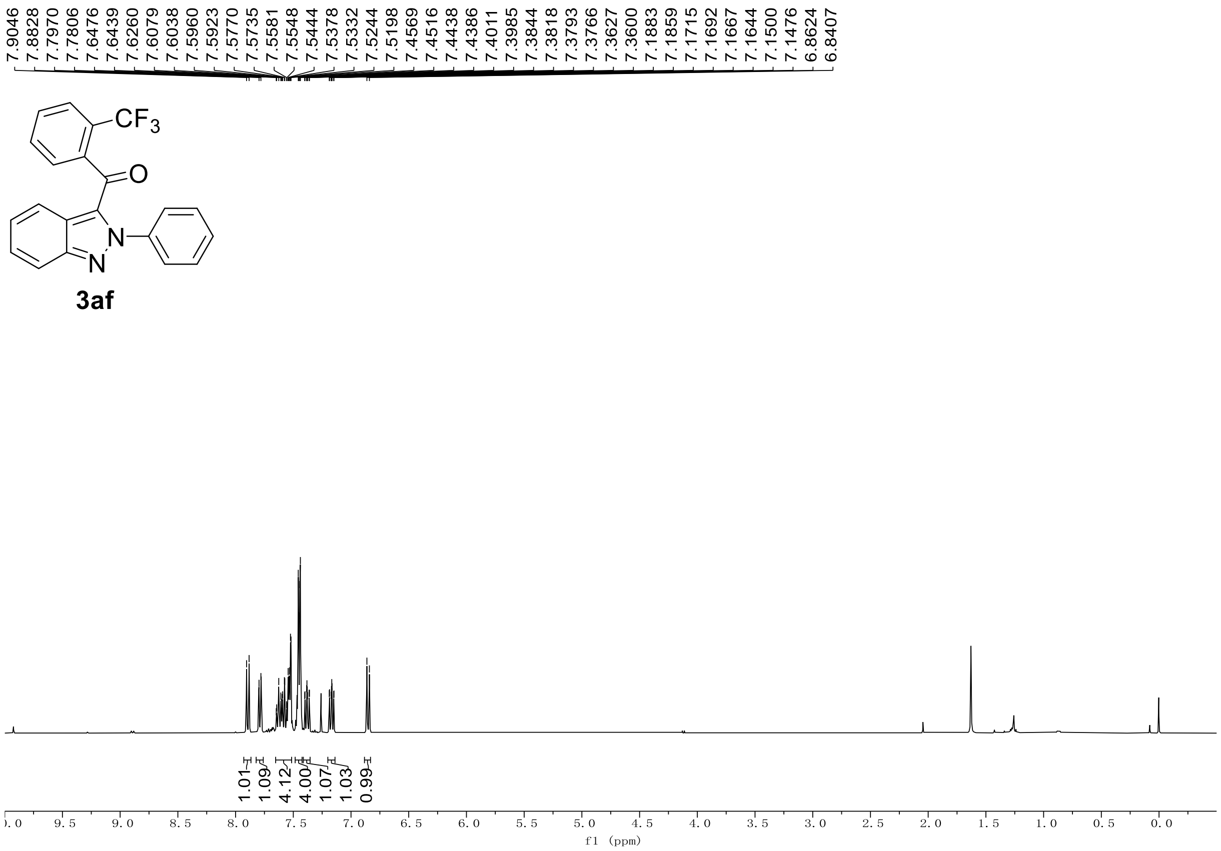

Figure S68 :^{1}H NMR spectrum of **3af** (400 MHz, $CDCl_3$)

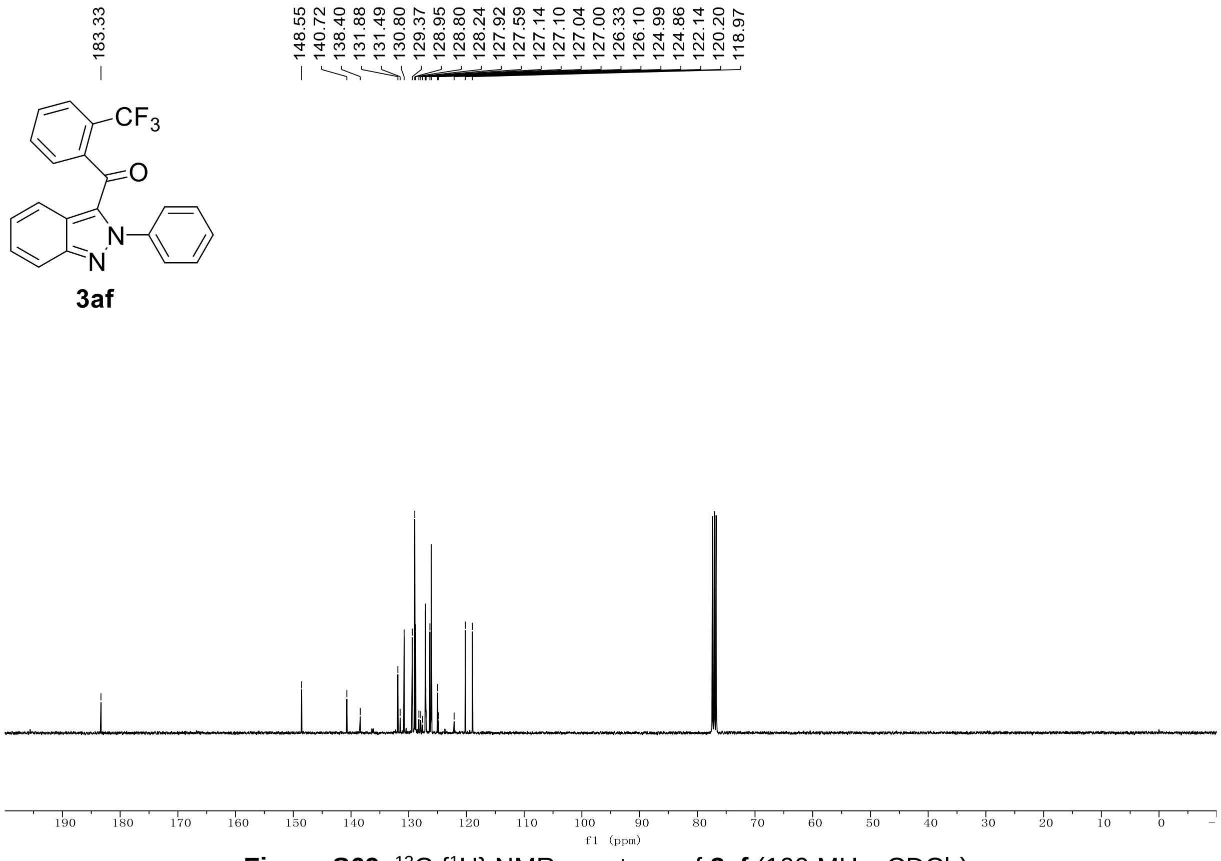

Figure S69 :^{13}C {^{1}H} NMR spectrum of **3af** (100 MHz, $CDCl_3$)

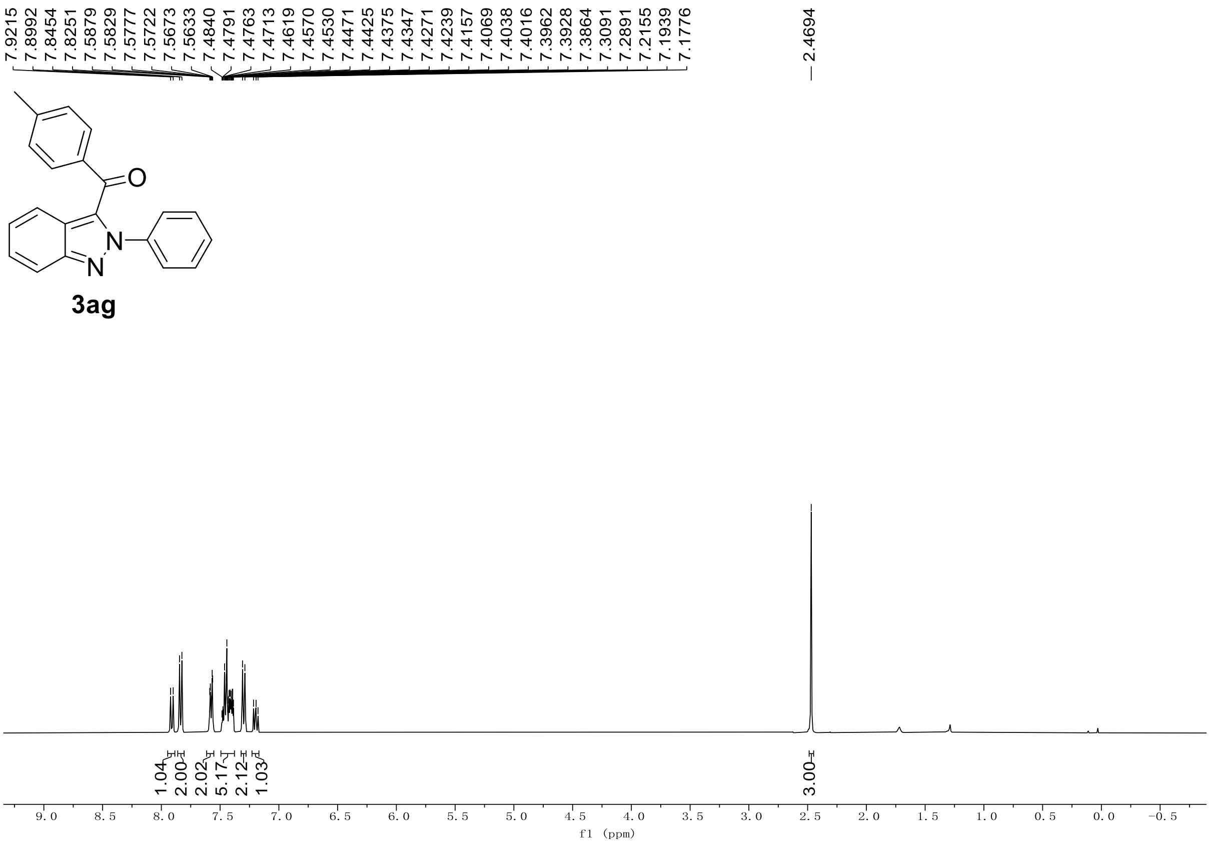

Figure S70 :^{1}H NMR spectrum of **3ag** (400 MHz, $CDCl_3$)

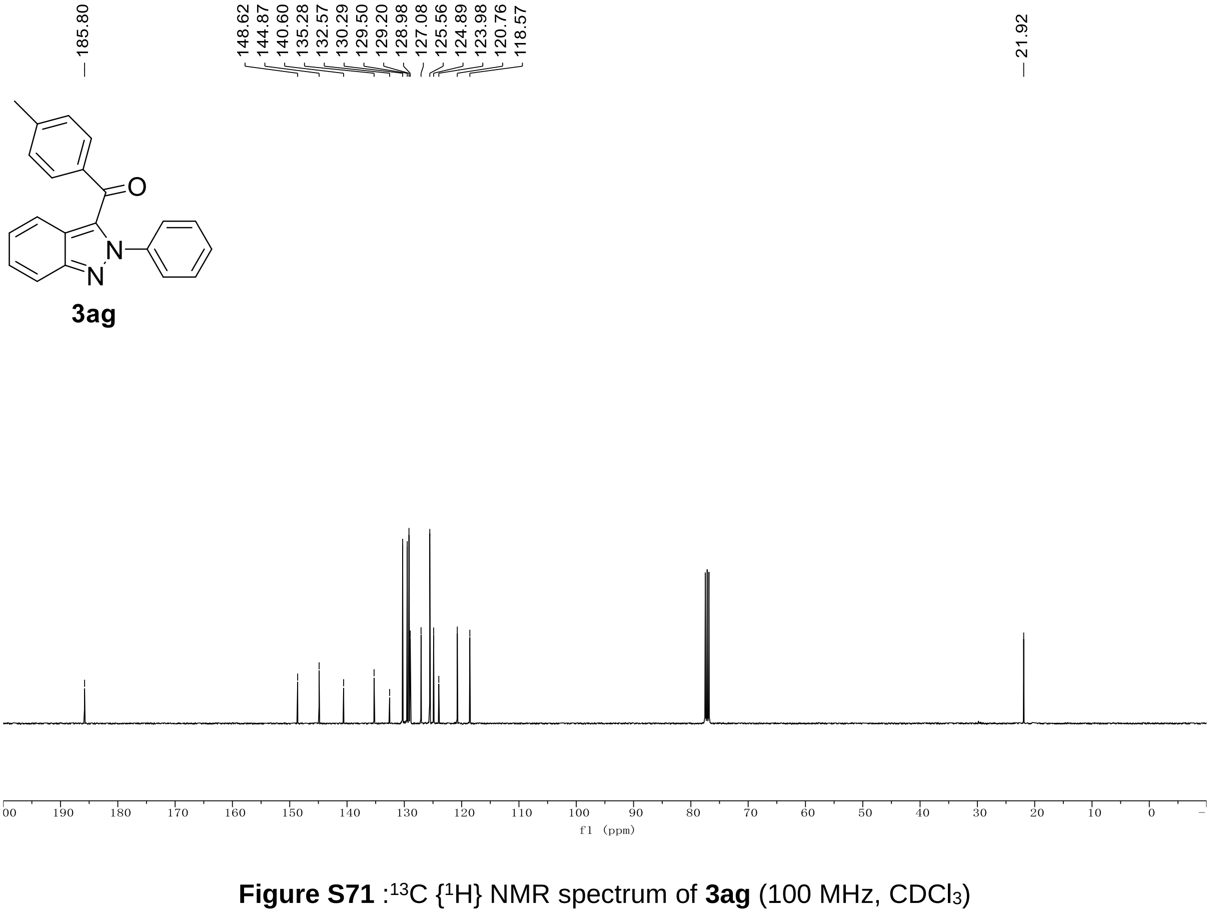

Figure S71 :^{13}C {^{1}H} NMR spectrum of **3ag** (100 MHz, $CDCl_3$)

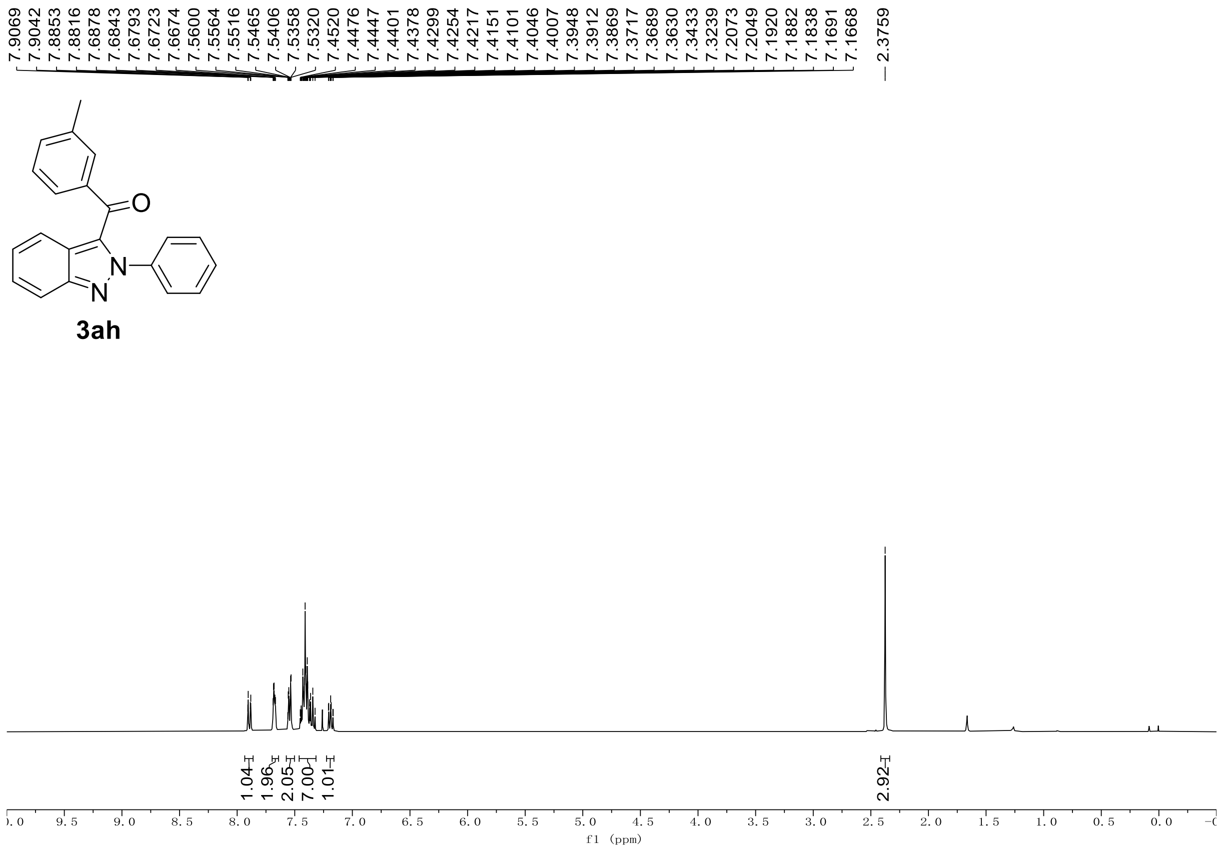

Figure S72 :^{1}H NMR spectrum of **3ah** (400 MHz, $CDCl_3$)

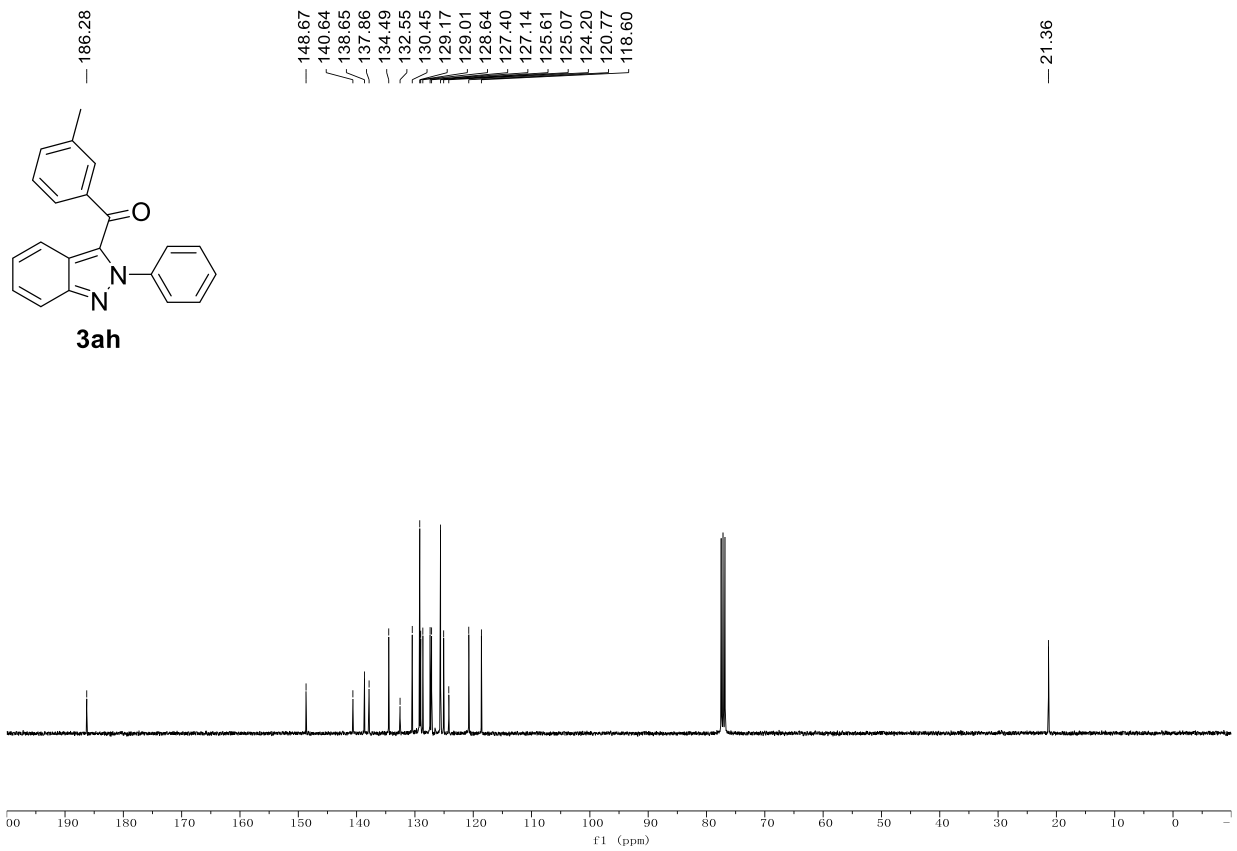

Figure S73 :^{13}C {^{1}H} NMR spectrum of **3ah** (100 MHz, $CDCl_3$)

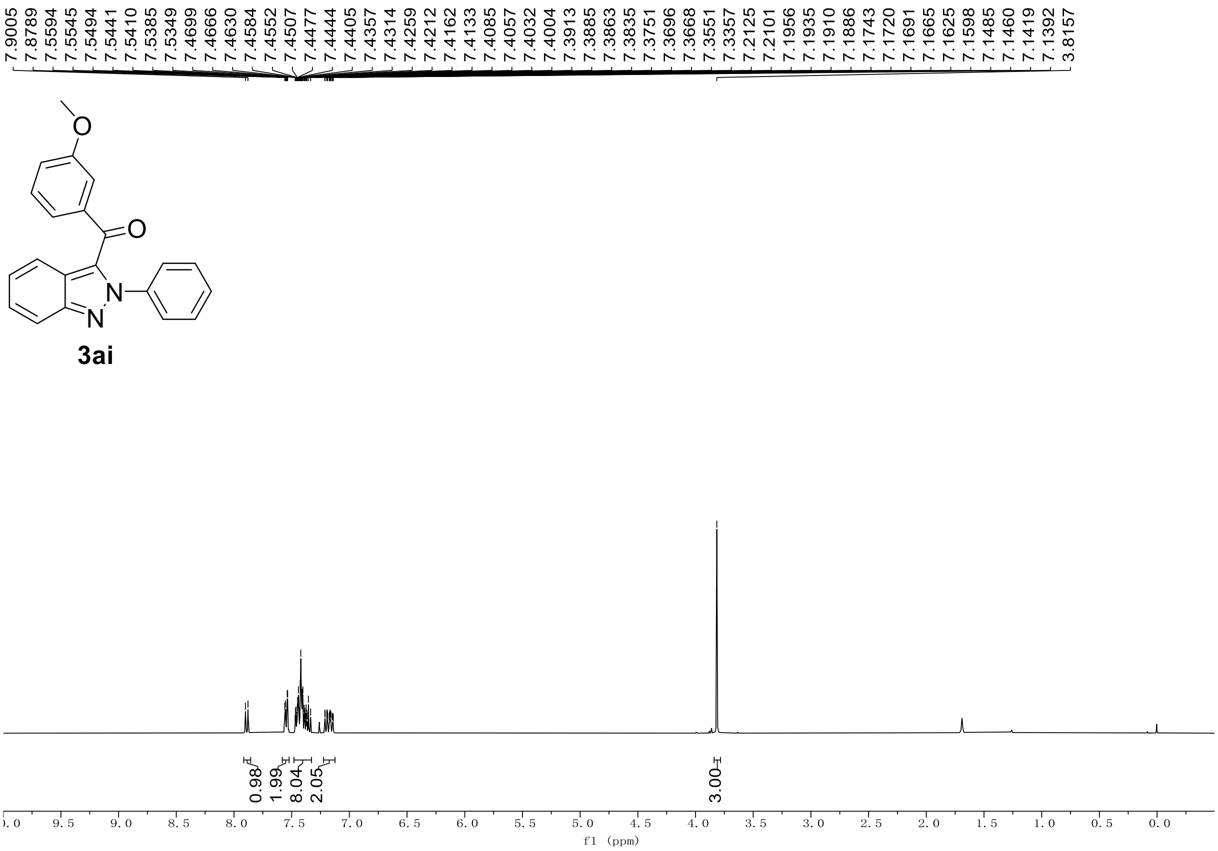

Figure S74 :^{1}H NMR spectrum of **3ai** (400 MHz, $CDCl_3$)

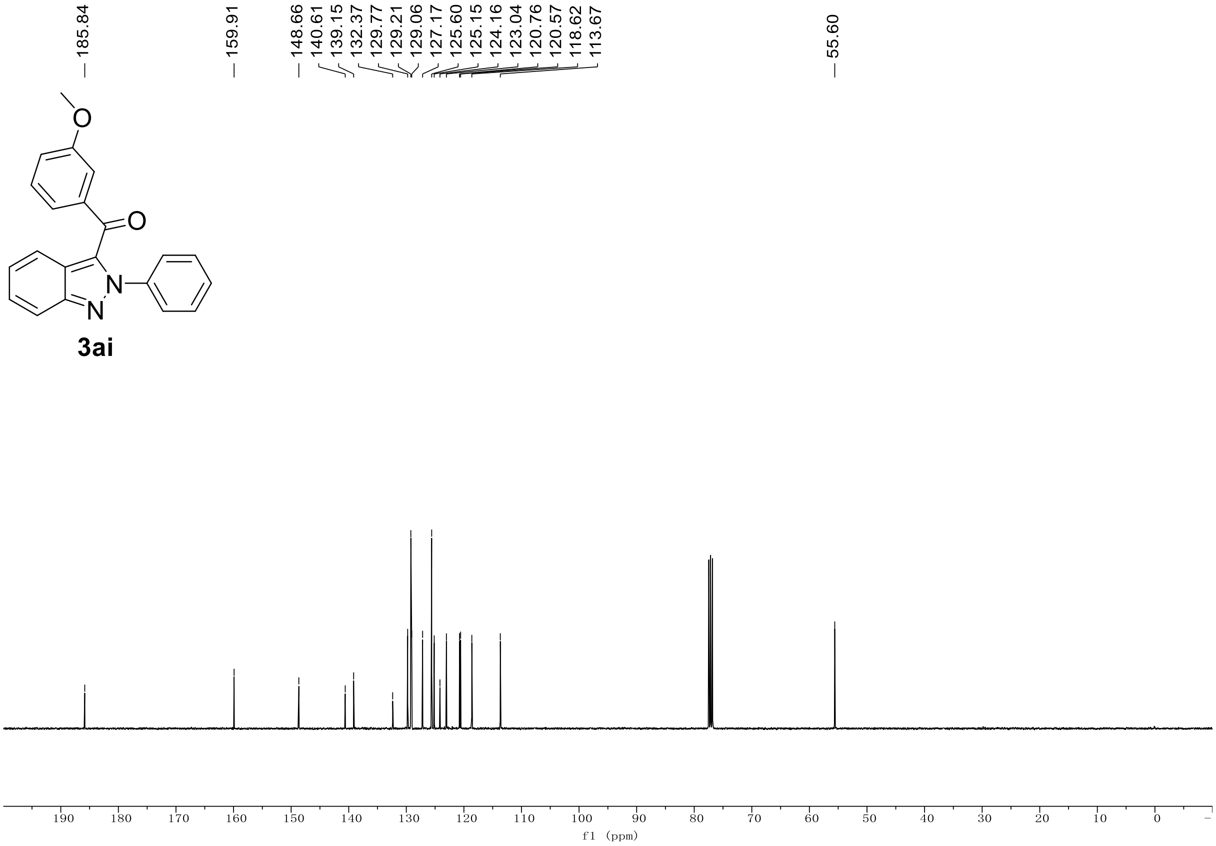

Figure S75 :^{13}C {^{1}H} NMR spectrum of **3ai** (100 MHz, $CDCl_3$)

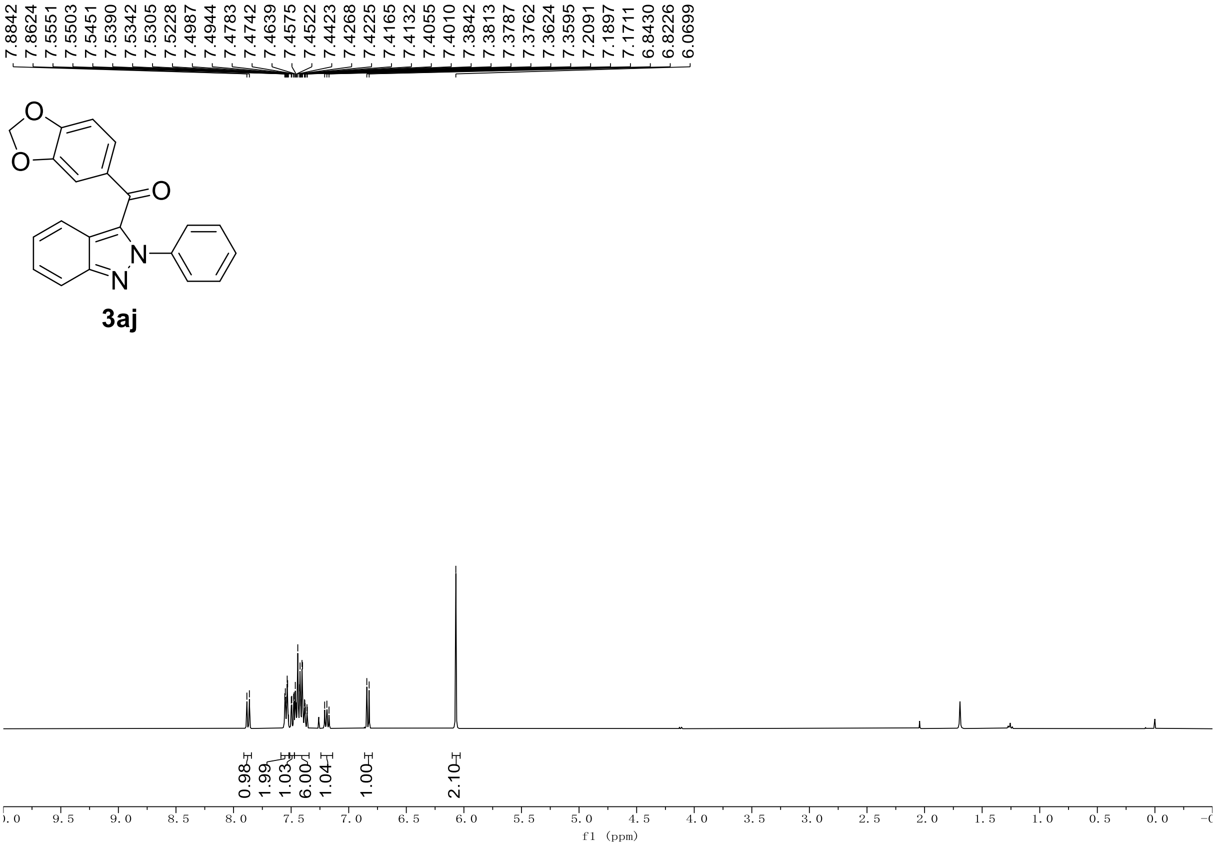

Figure S76 :^{1}H NMR spectrum of **3aj** (400 MHz, $CDCl_3$)

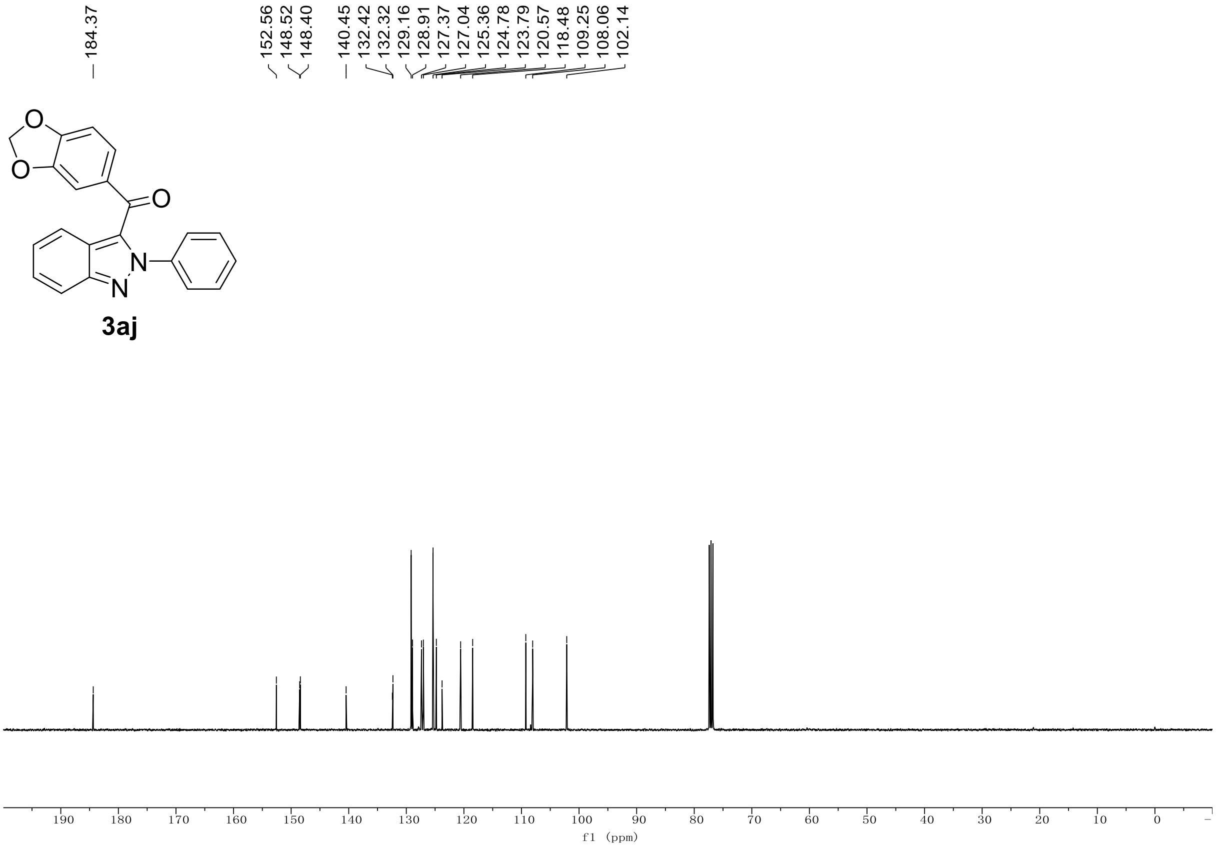

Figure S77 :^{13}C {^{1}H} NMR spectrum of **3aj** (100 MHz, $CDCl_3$)

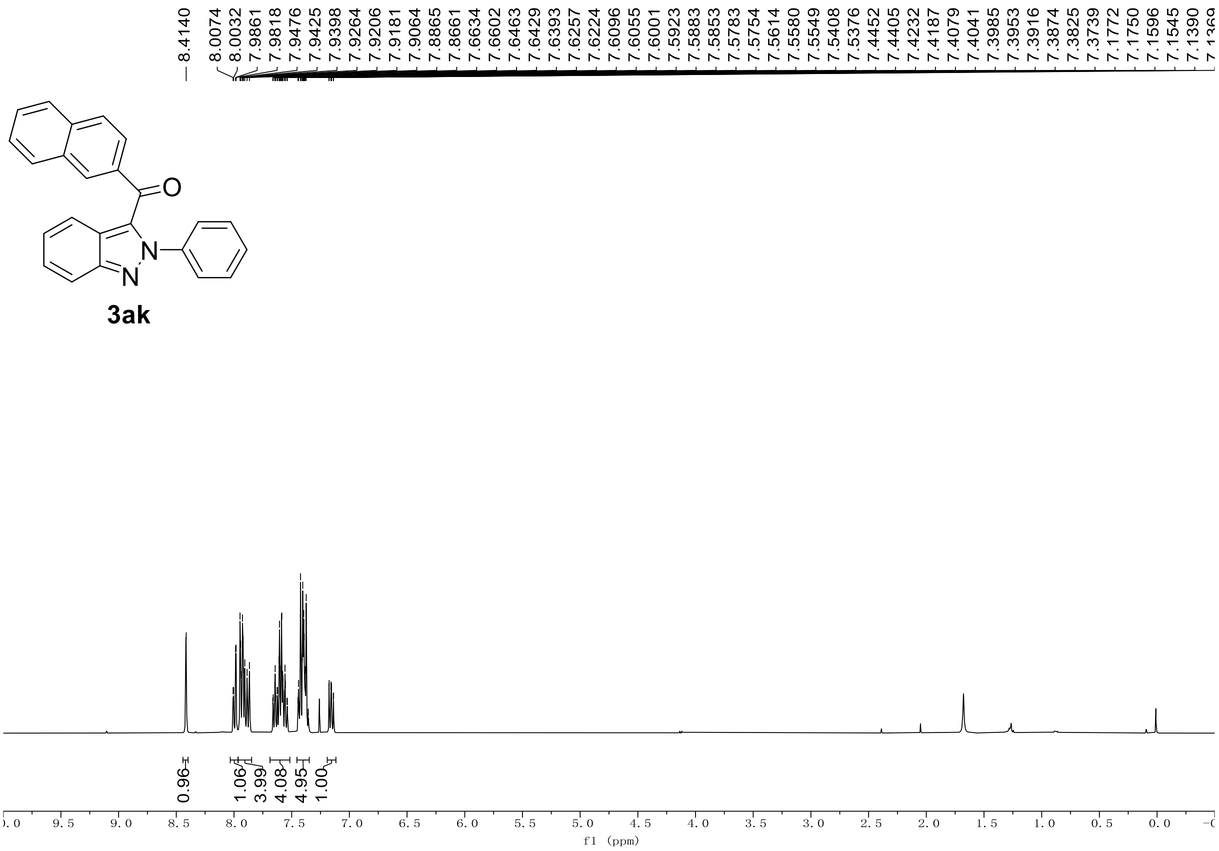

Figure S78 :^{1}H NMR spectrum of **3ak** (400 MHz, $CDCl_3$)

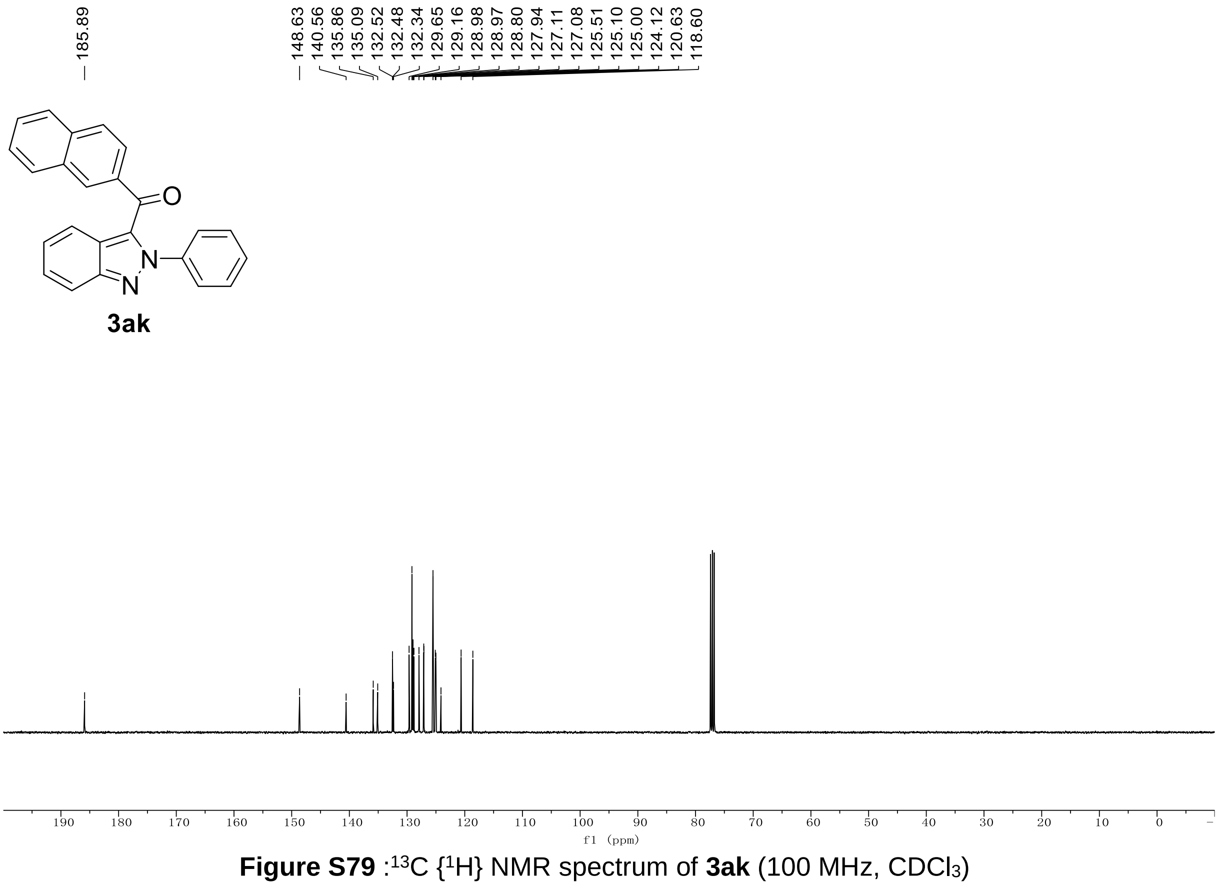

Figure S79 :^{13}C {^{1}H} NMR spectrum of **3ak** (100 MHz, $CDCl_3$)

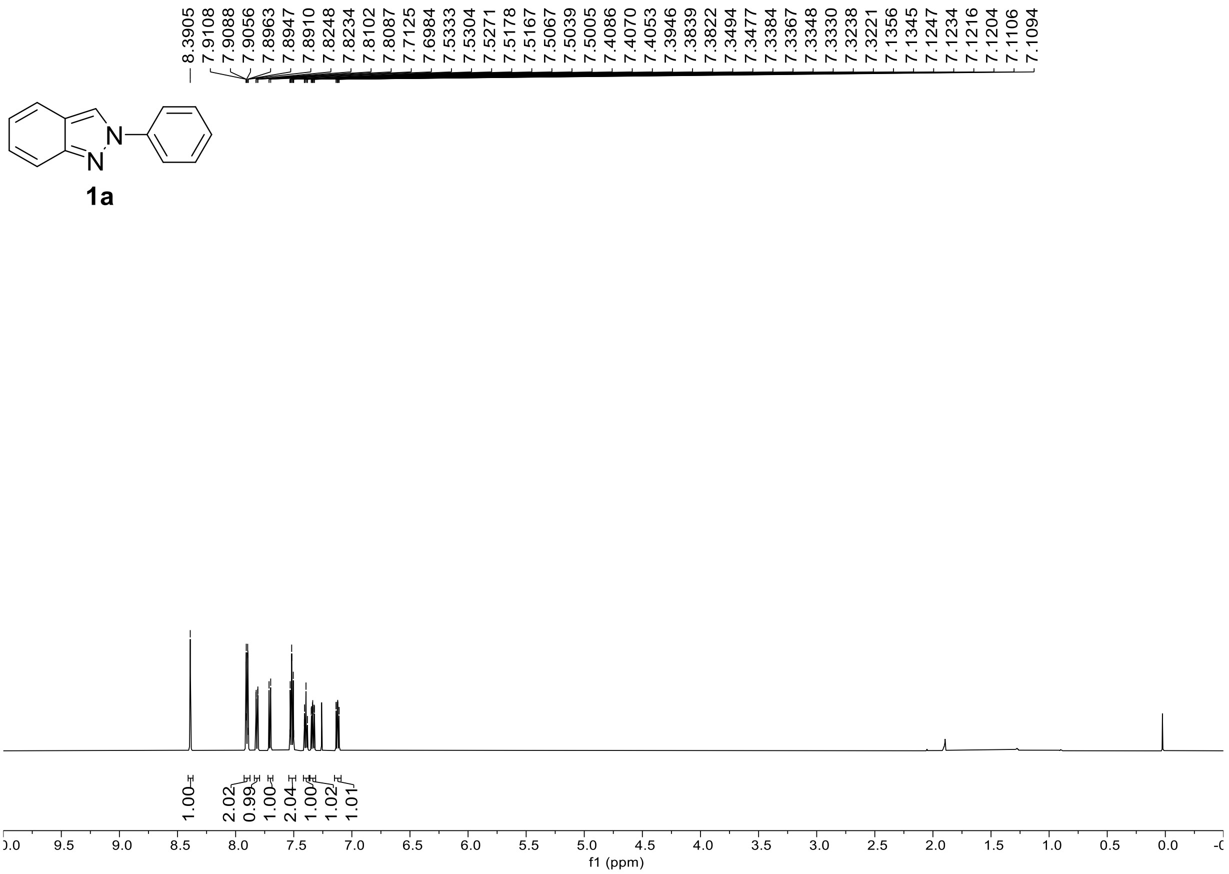

1a

Figure S80 :^{1}H NMR spectrum of **1a** (600 MHz, $CDCl_3$)

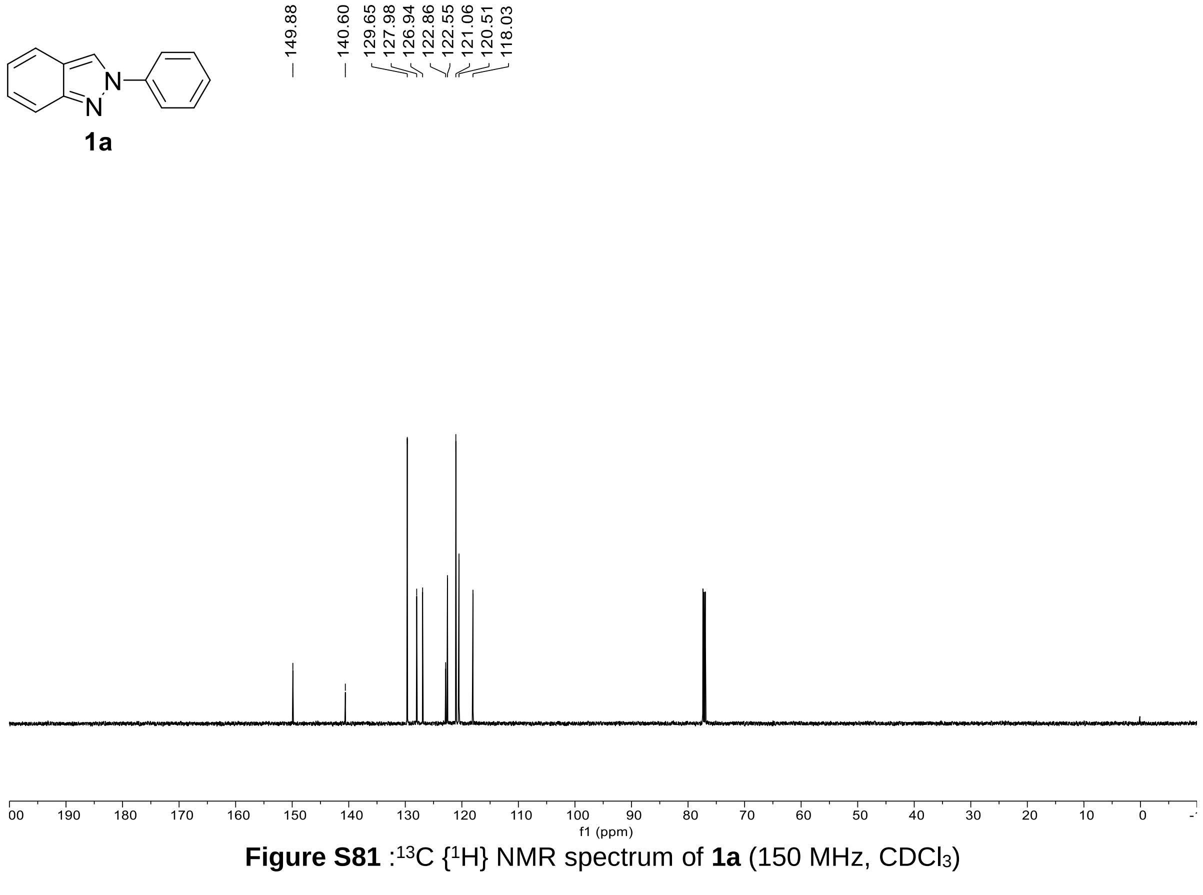

1a

Figure S81 :^{13}C {^{1}H} NMR spectrum of **1a** (150 MHz, $CDCl_3$)

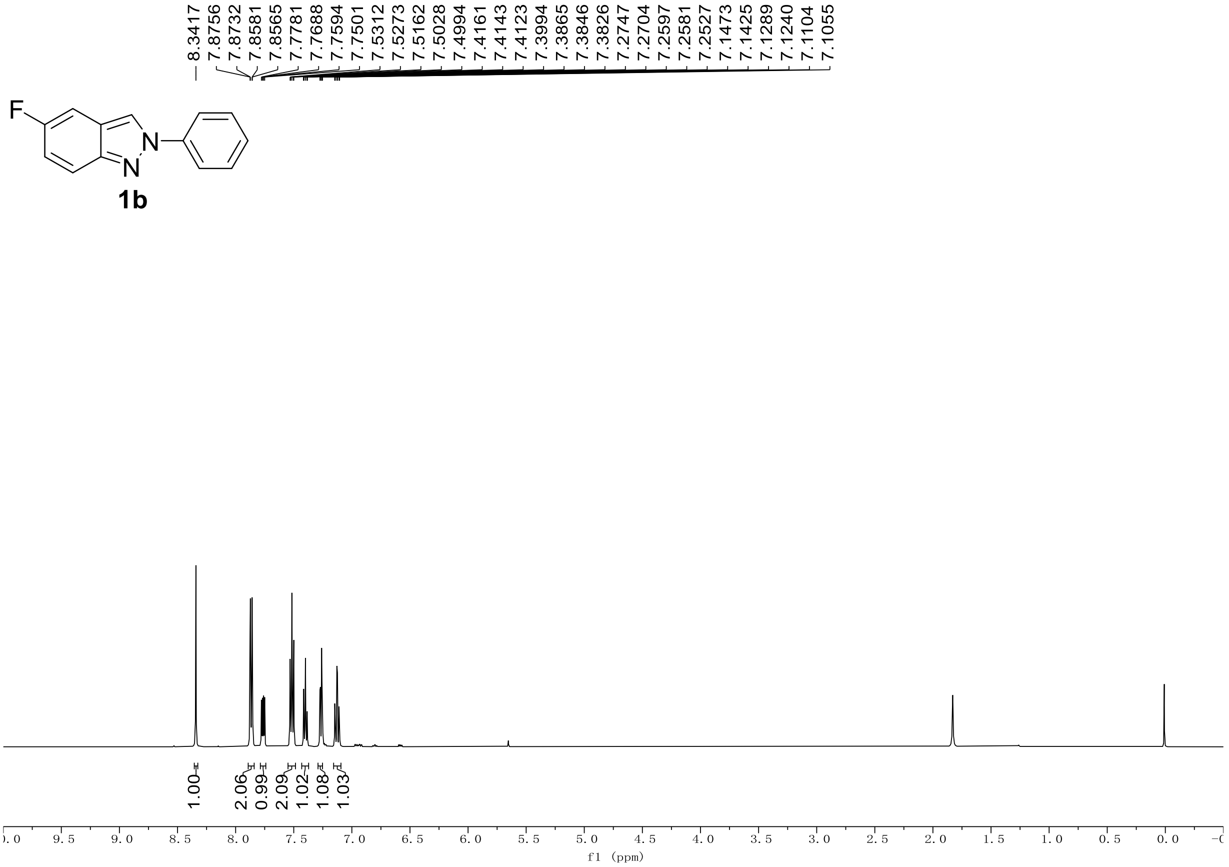

Figure S82 :^{1}H NMR spectrum of **1b** (500 MHz, $CDCl_3$)

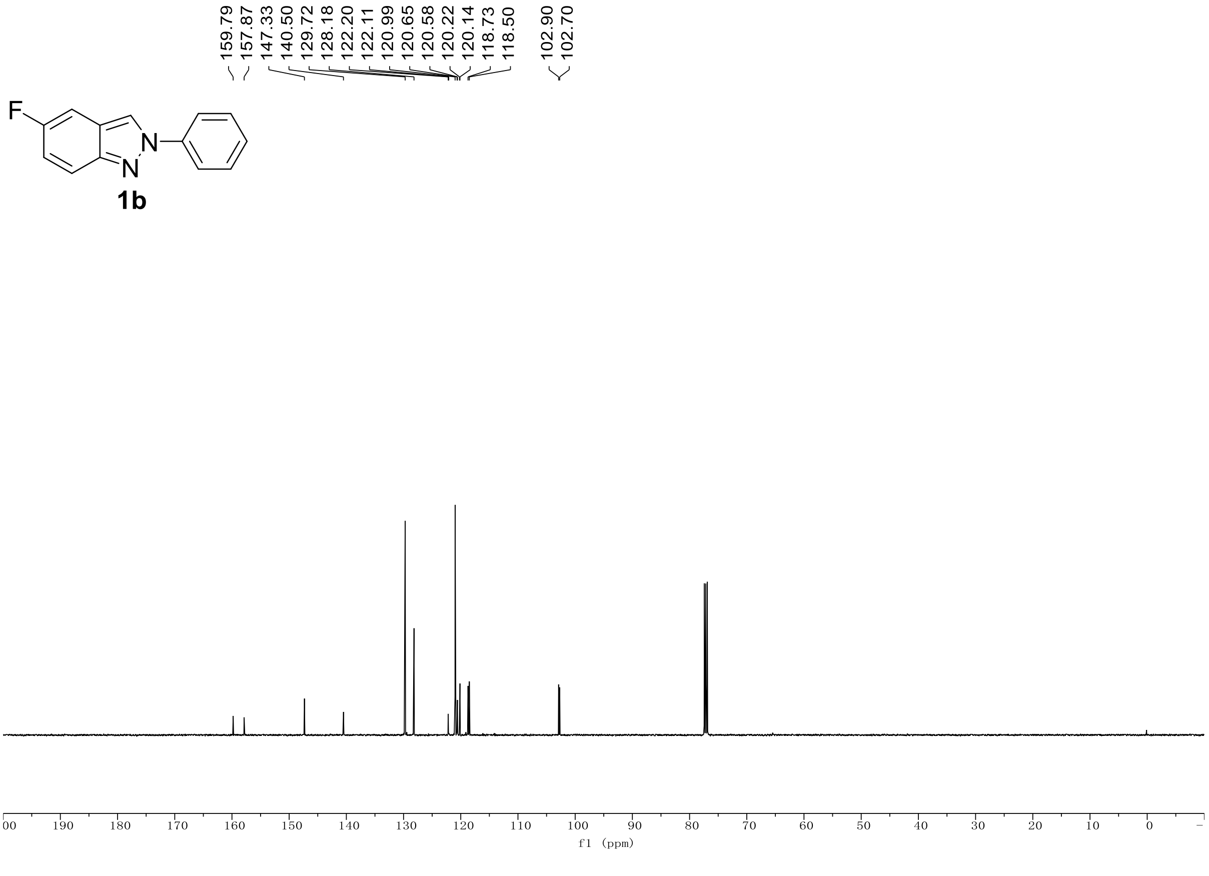

Figure S83 :^{13}C {^{1}H} NMR spectrum of **1b** (125 MHz, $CDCl_3$)

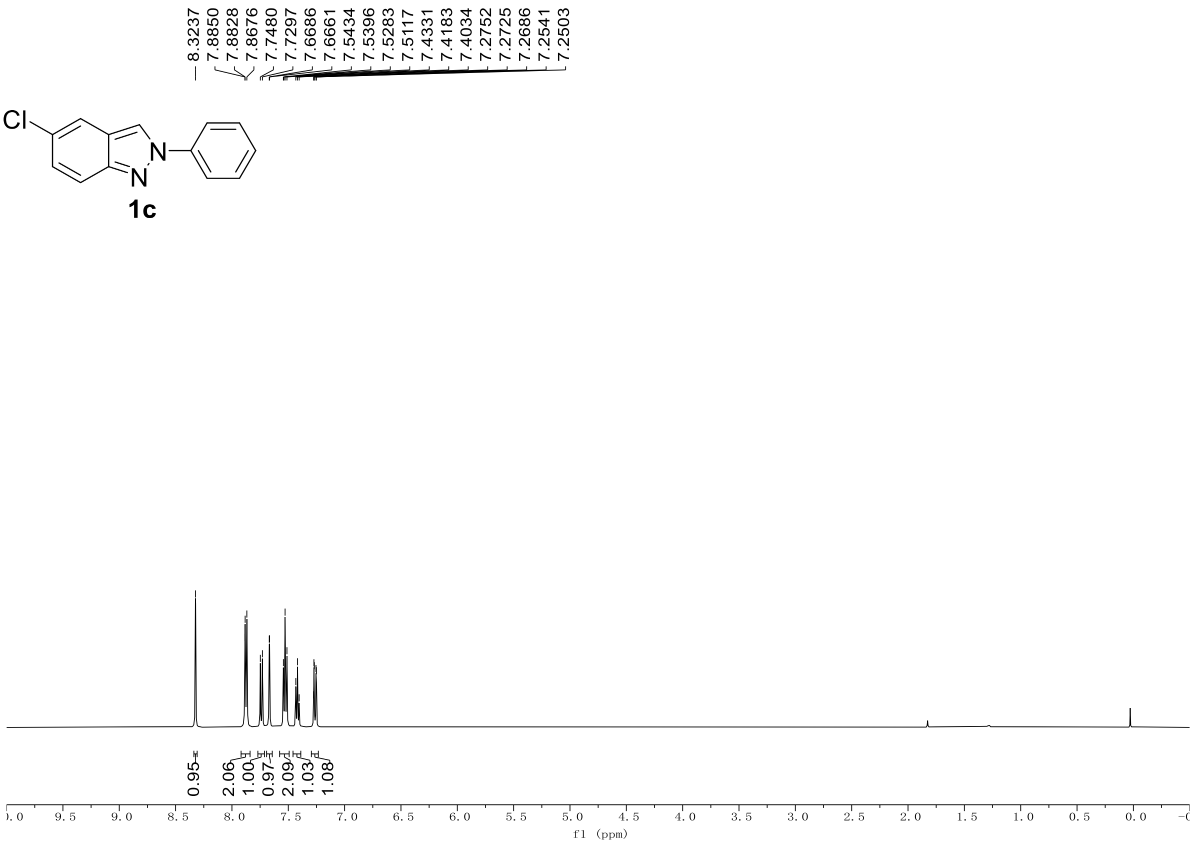

Figure S84 :^{1}H NMR spectrum of **1c** (500 MHz, $CDCl_3$)

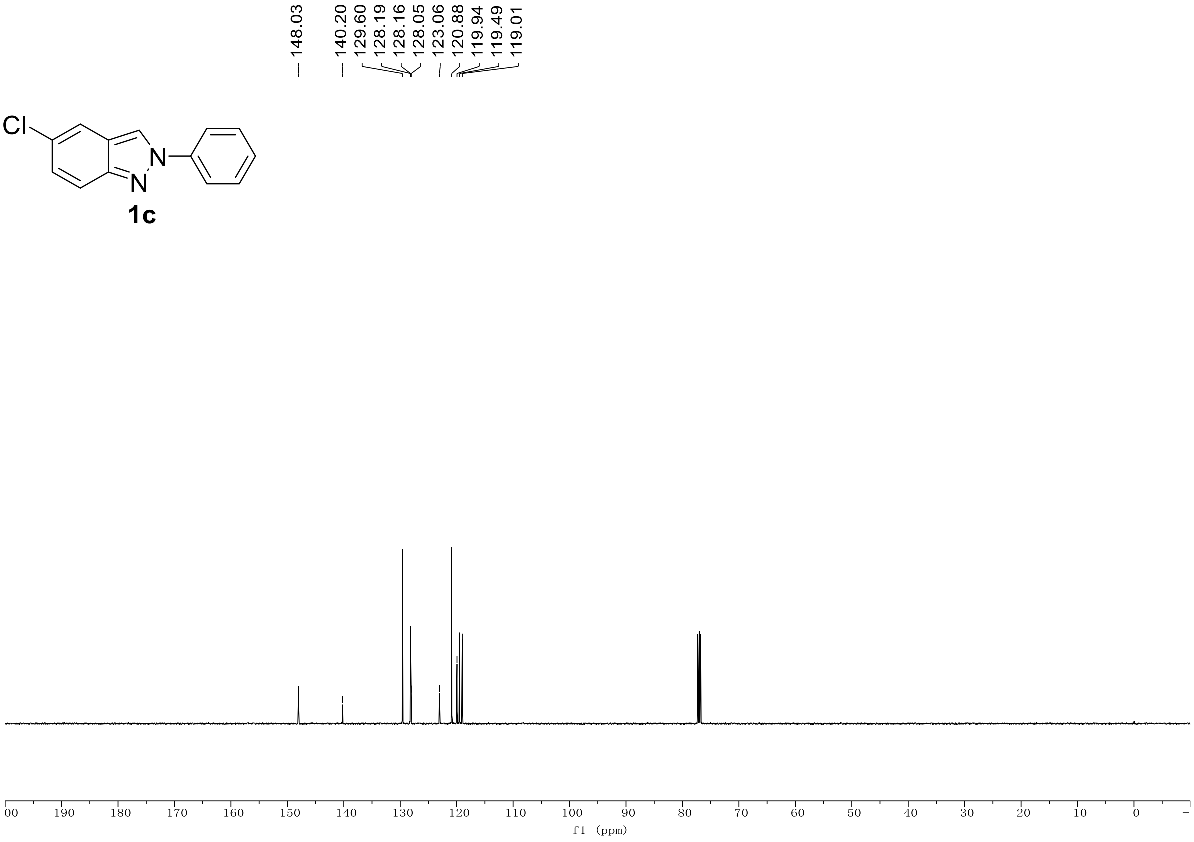

Figure S85 :^{13}C {^{1}H} NMR spectrum of **1c** (125 MHz, $CDCl_3$)

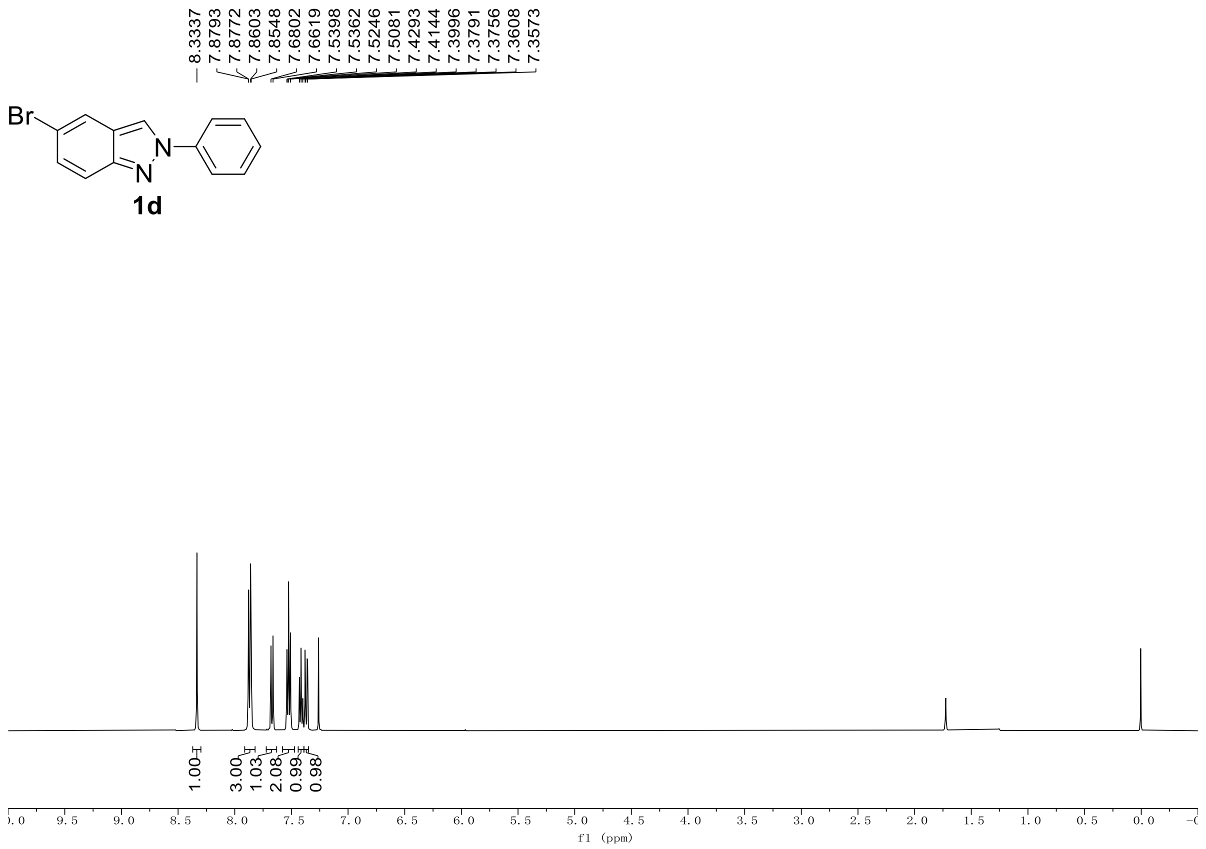

Figure S86 :^{1}H NMR spectrum of **1d** (500 MHz, $CDCl_3$)

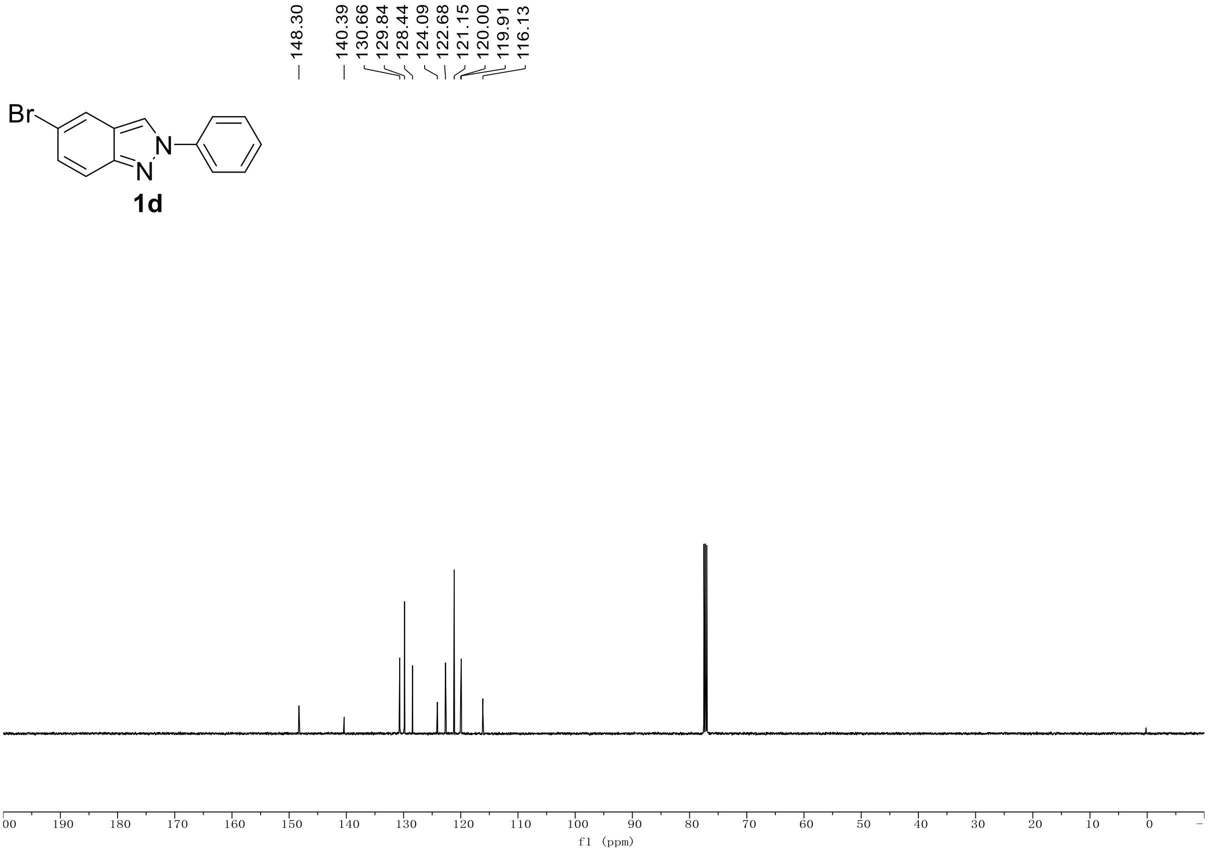

Figure S87 :^{13}C {^{1}H} NMR spectrum of **1d** (125 MHz, $CDCl_3$)

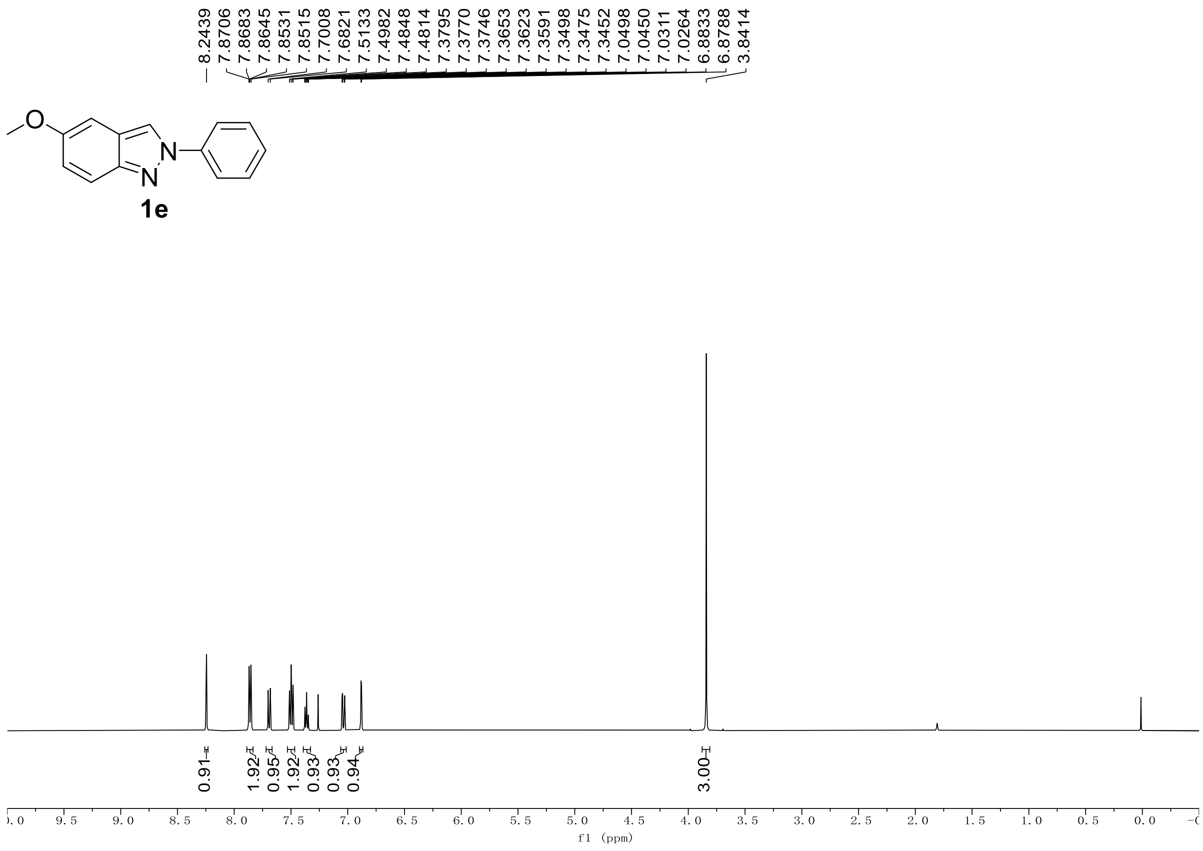

Figure S88 :^{1}H NMR spectrum of **1e** (500 MHz, $CDCl_3$)

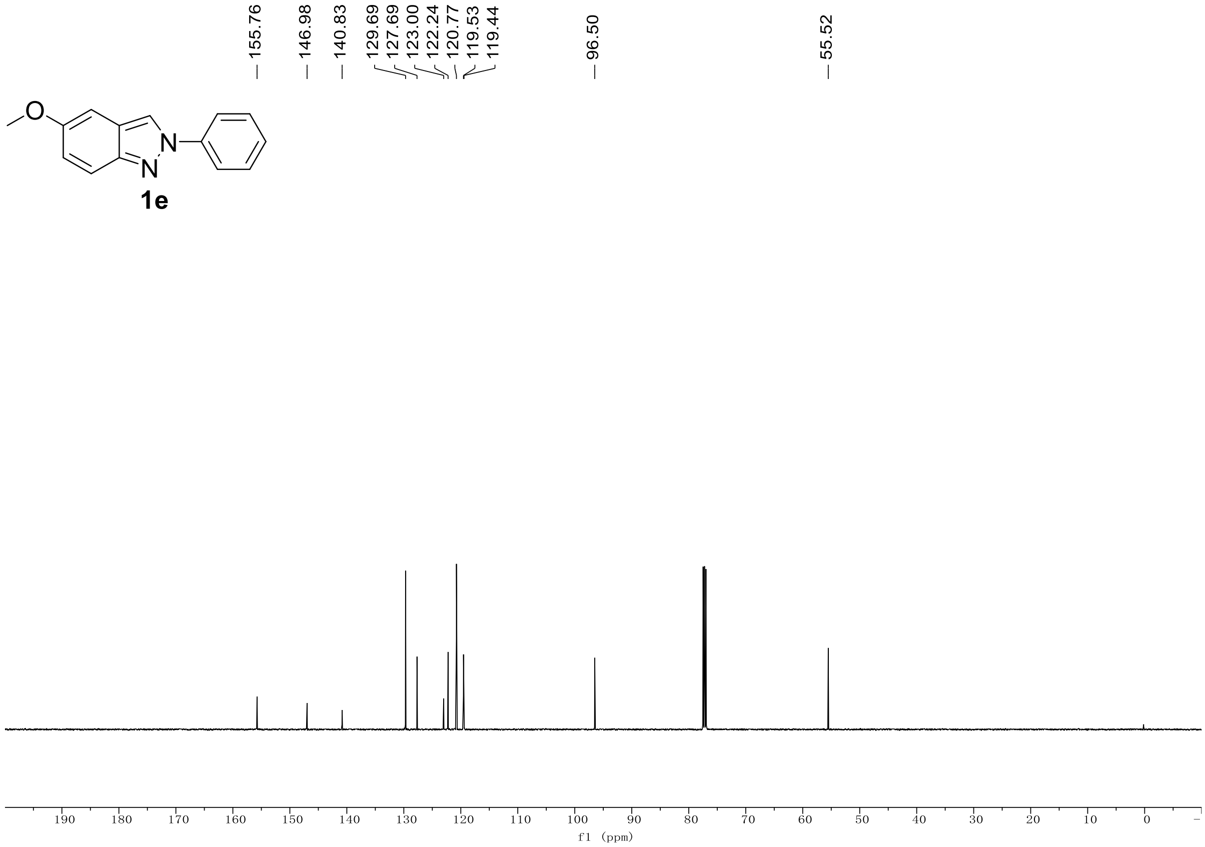

Figure S89 :^{13}C {^{1}H} NMR spectrum of **1e** (125 MHz, $CDCl_3$)

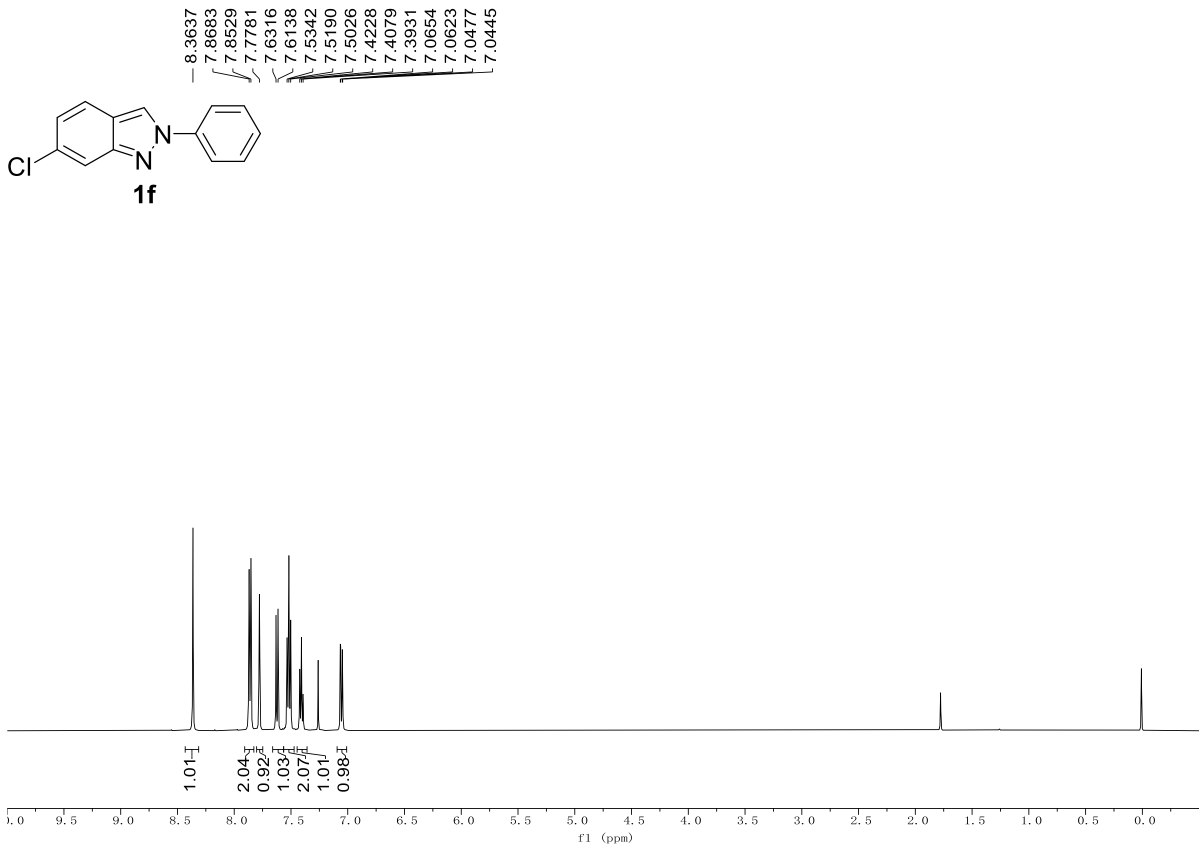

Figure S90 :^{1}H NMR spectrum of **1f** (500 MHz, $CDCl_3$)

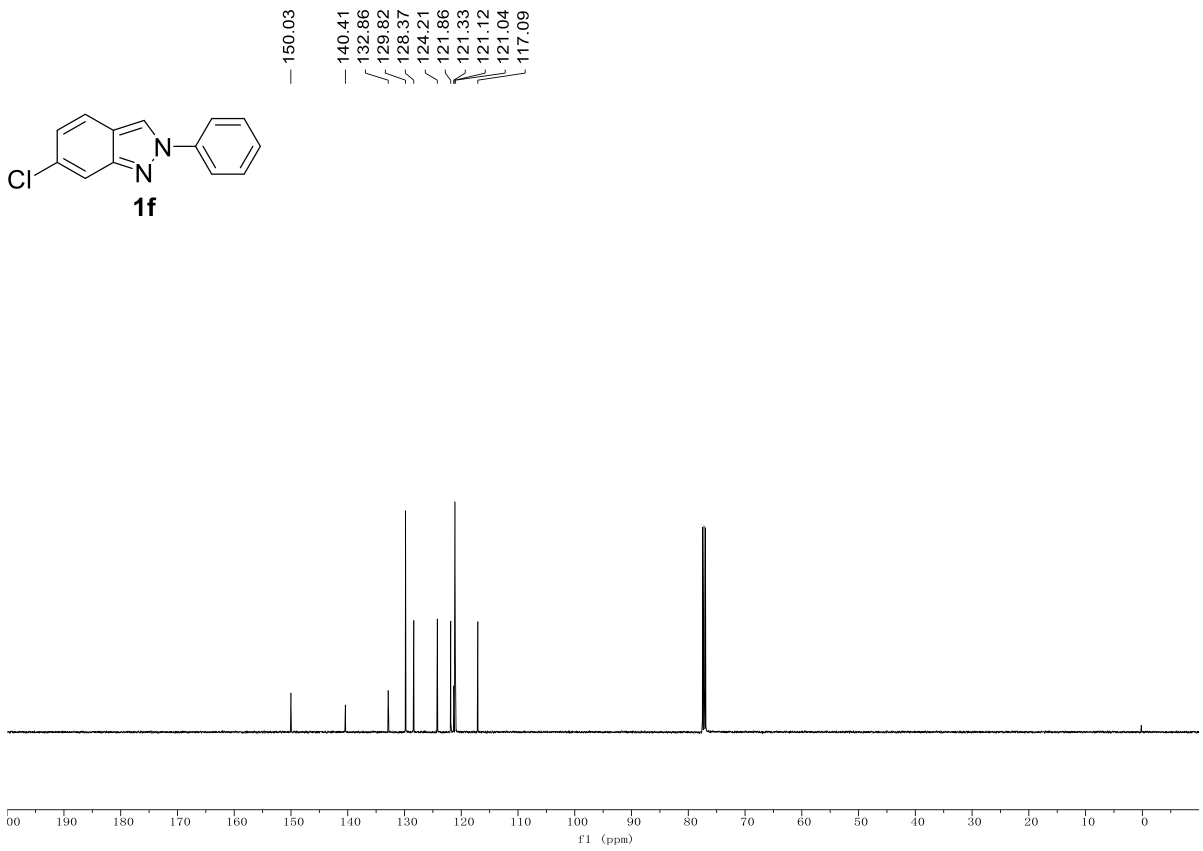

Figure S91 :^{13}C {^{1}H} NMR spectrum of **1f** (125 MHz, $CDCl_3$)

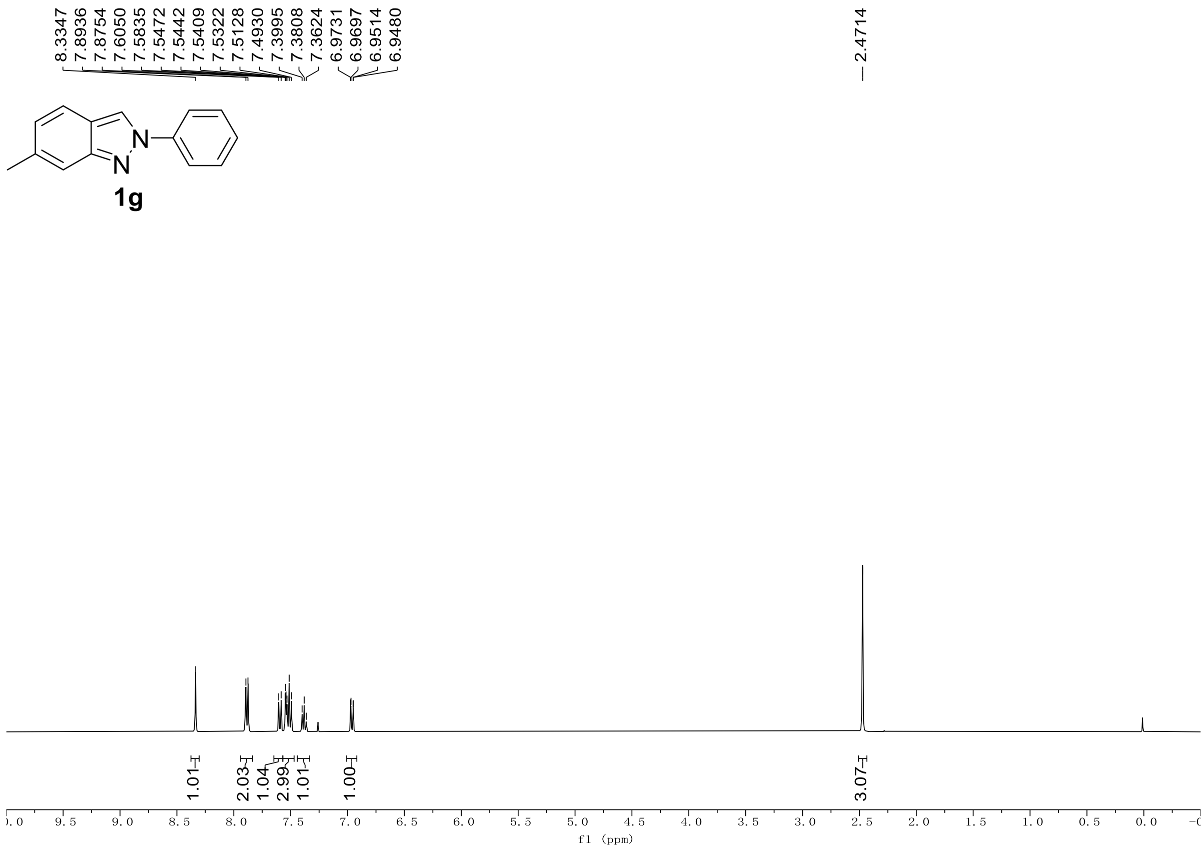

Figure S92 :^{1}H NMR spectrum of **1g** (400 MHz, $CDCl_3$)

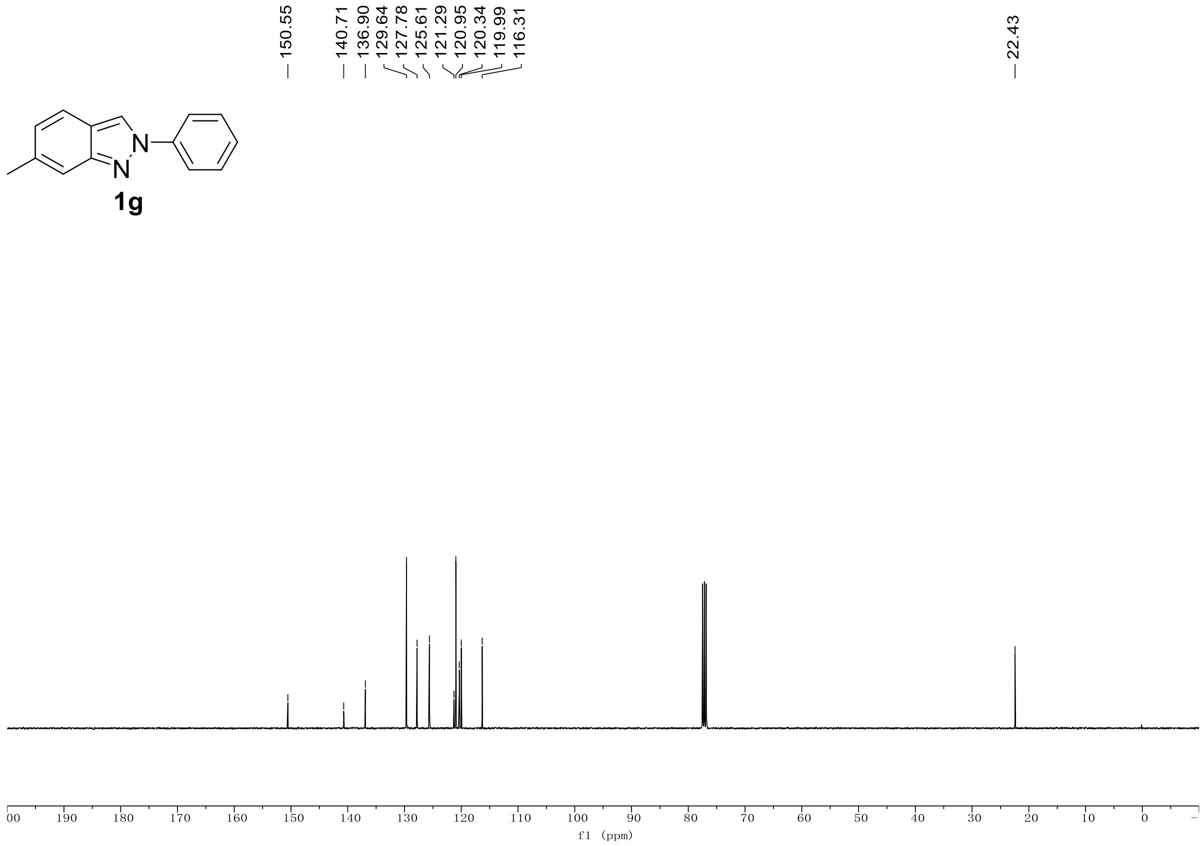

Figure S93 :^{13}C {^{1}H} NMR spectrum of **1g** (100 MHz, $CDCl_3$)

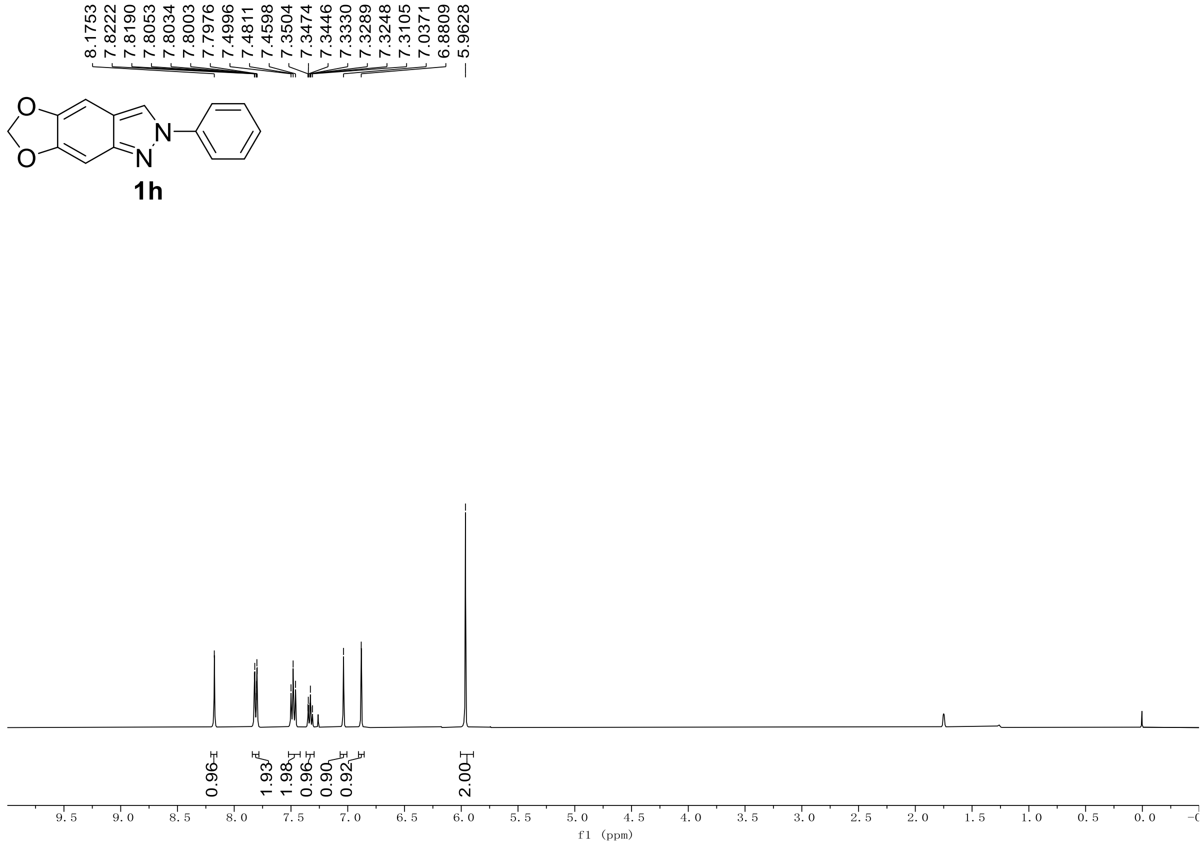

Figure S94 :^{1}H NMR spectrum of **1h** (400 MHz, $CDCl_3$)

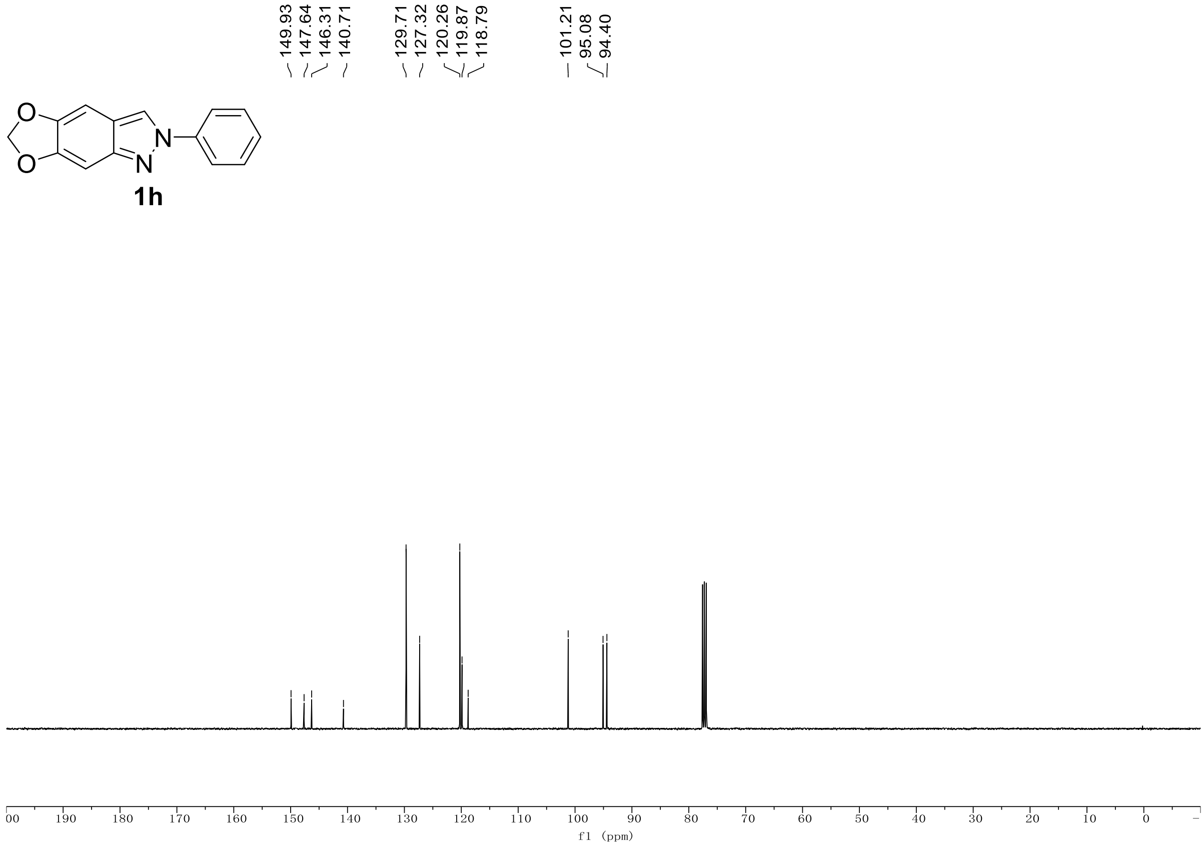

Figure S95 :^{13}C {^{1}H} NMR spectrum of **1h** (100 MHz, $CDCl_3$)

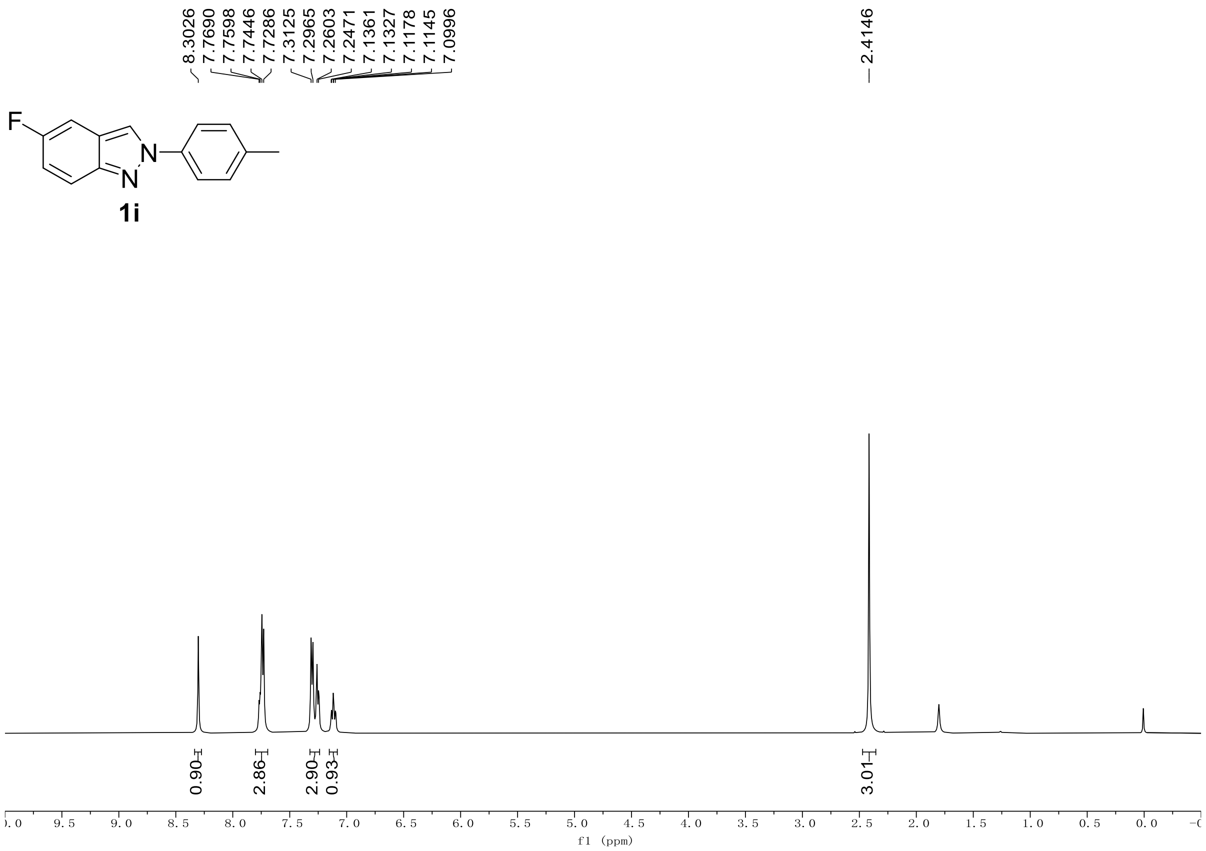

Figure S96 :^{1}H NMR spectrum of **1i** (500 MHz, $CDCl_3$)

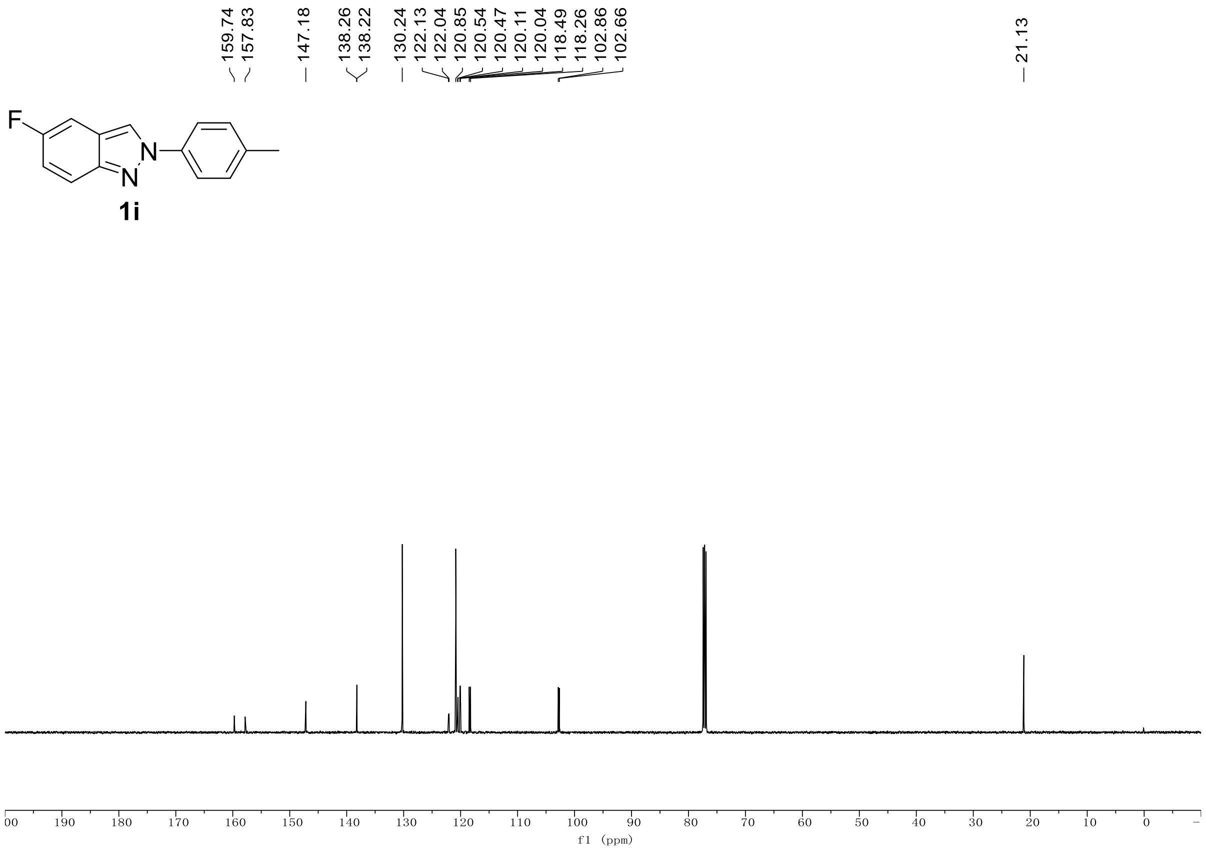

Figure S97 :^{13}C {^{1}H} NMR spectrum of **1i** (125 MHz, $CDCl_3$)

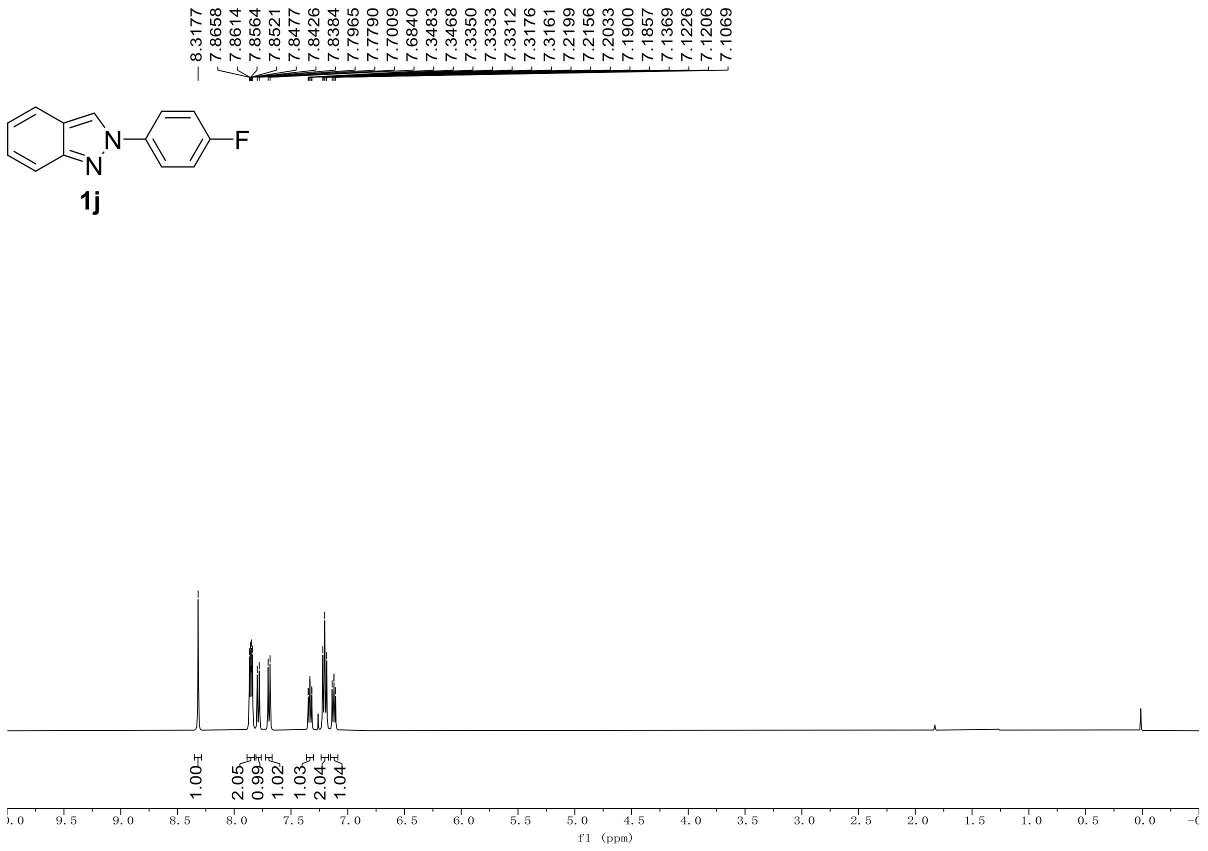

Figure S98 :^{1}H NMR spectrum of **1j** (500 MHz, $CDCl_3$)

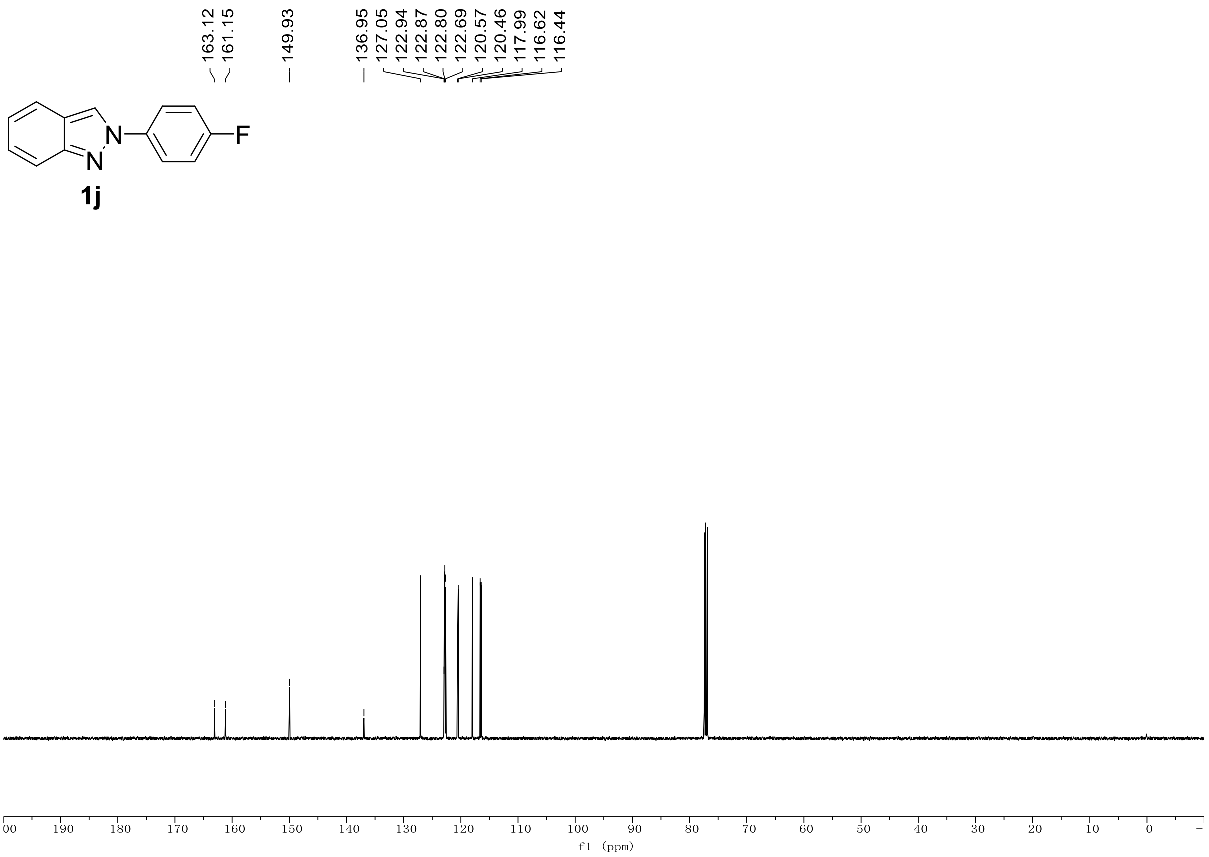

Figure S99 :^{13}C {^{1}H} NMR spectrum of **1j** (125 MHz, $CDCl_3$)

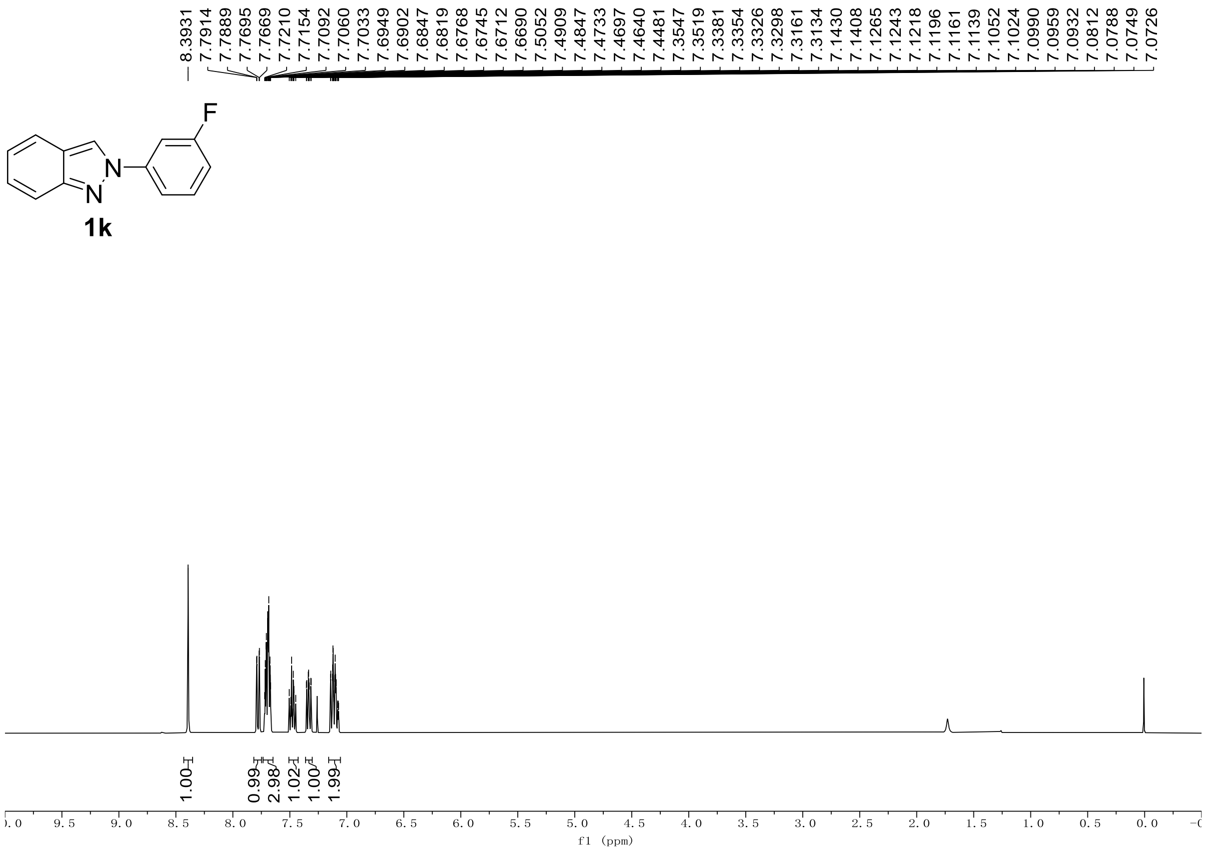

Figure S100 :^{1}H NMR spectrum of **1k** (400 MHz, $CDCl_3$)

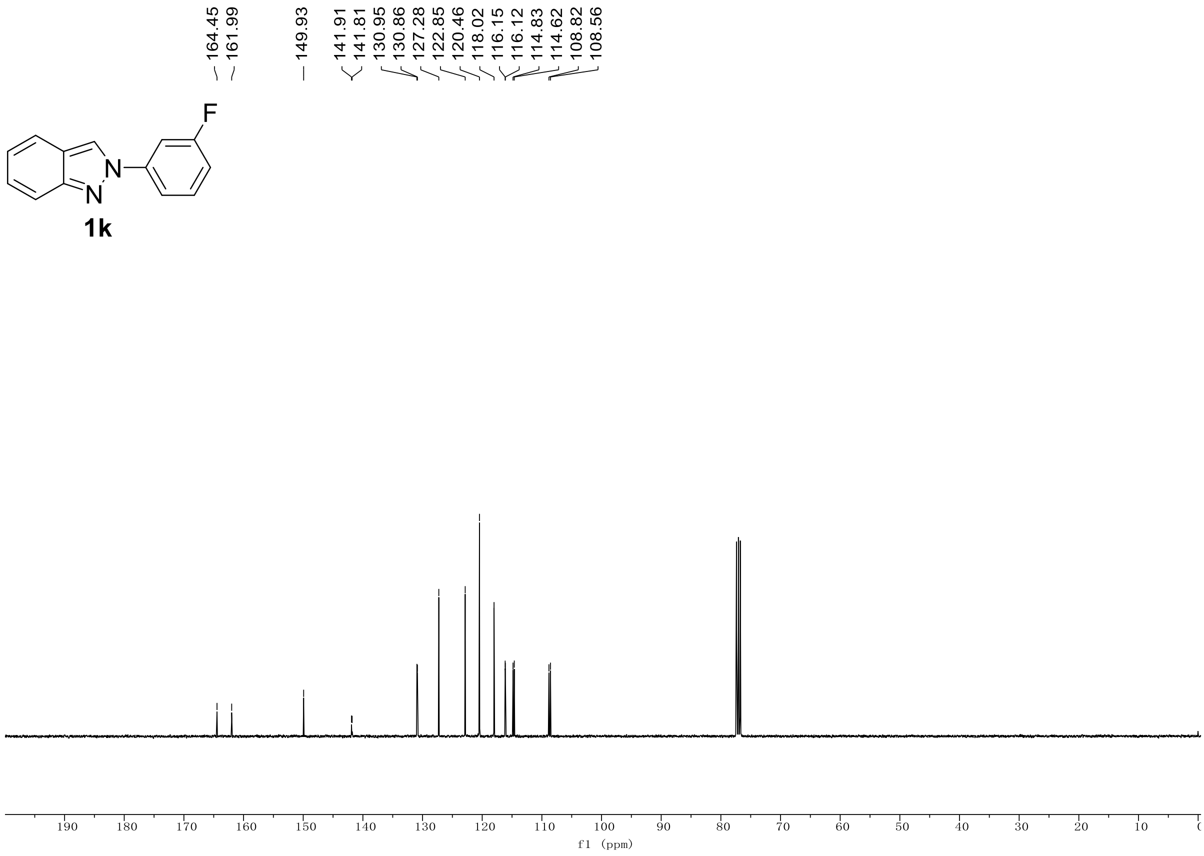

Figure S101 :^{13}C {^{1}H} NMR spectrum of **1k** (100 MHz, $CDCl_3$)

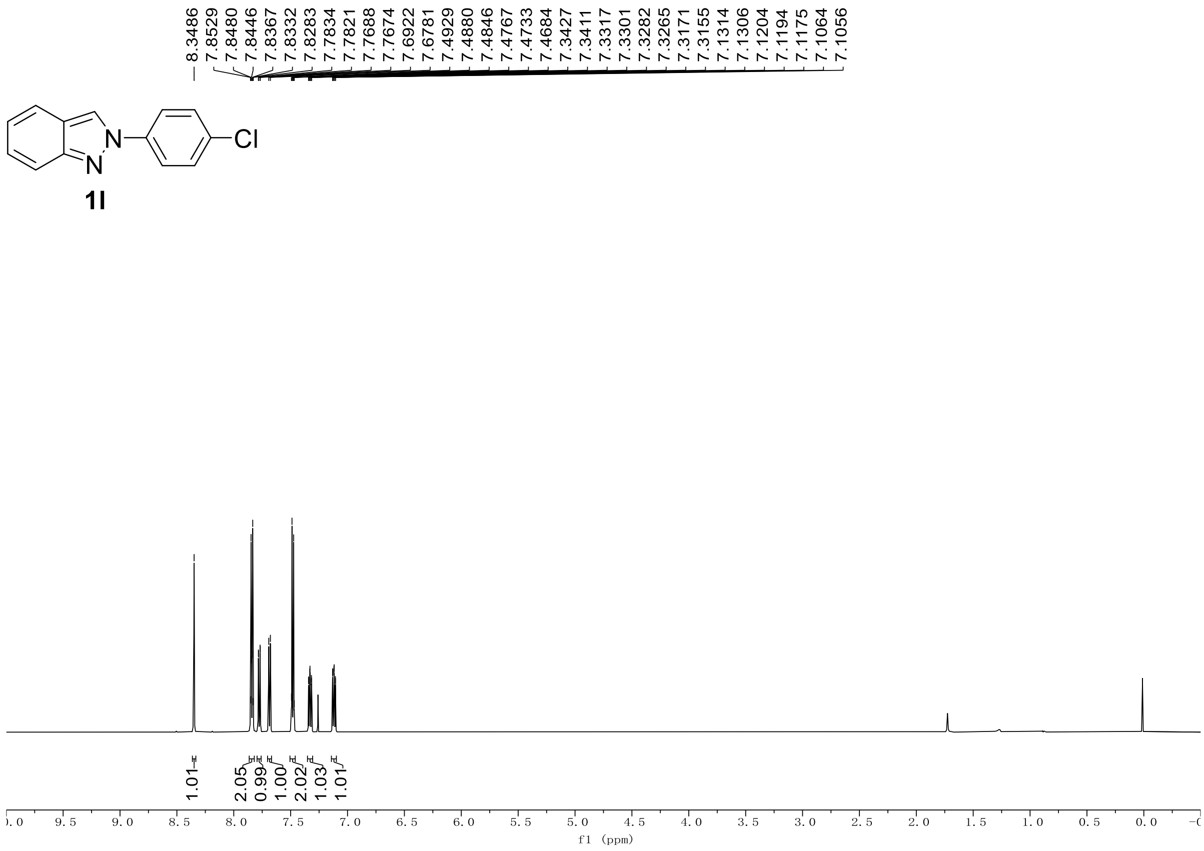

Figure S102 :^{1}H NMR spectrum of **1l** (600 MHz, $CDCl_3$)

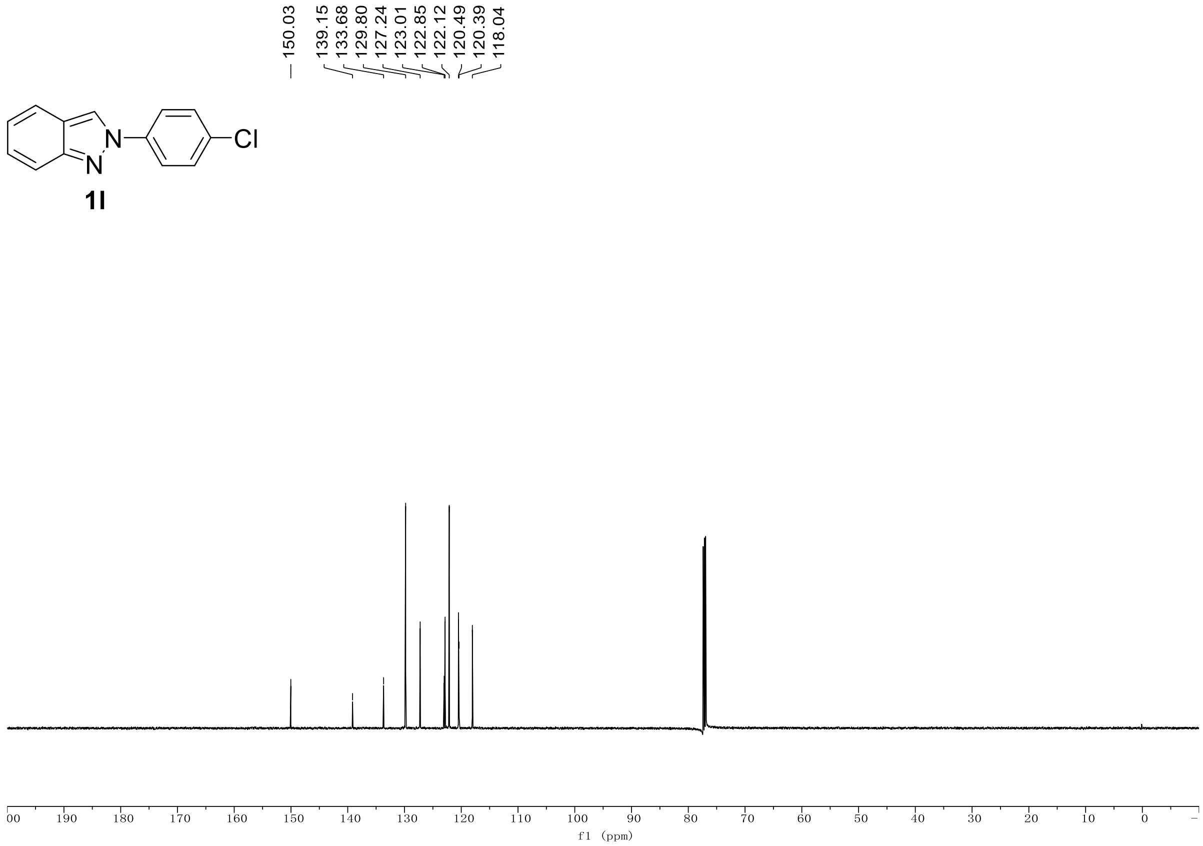

Figure S103 :^{13}C {^{1}H} NMR spectrum of **1l** (150 MHz, $CDCl_3$)

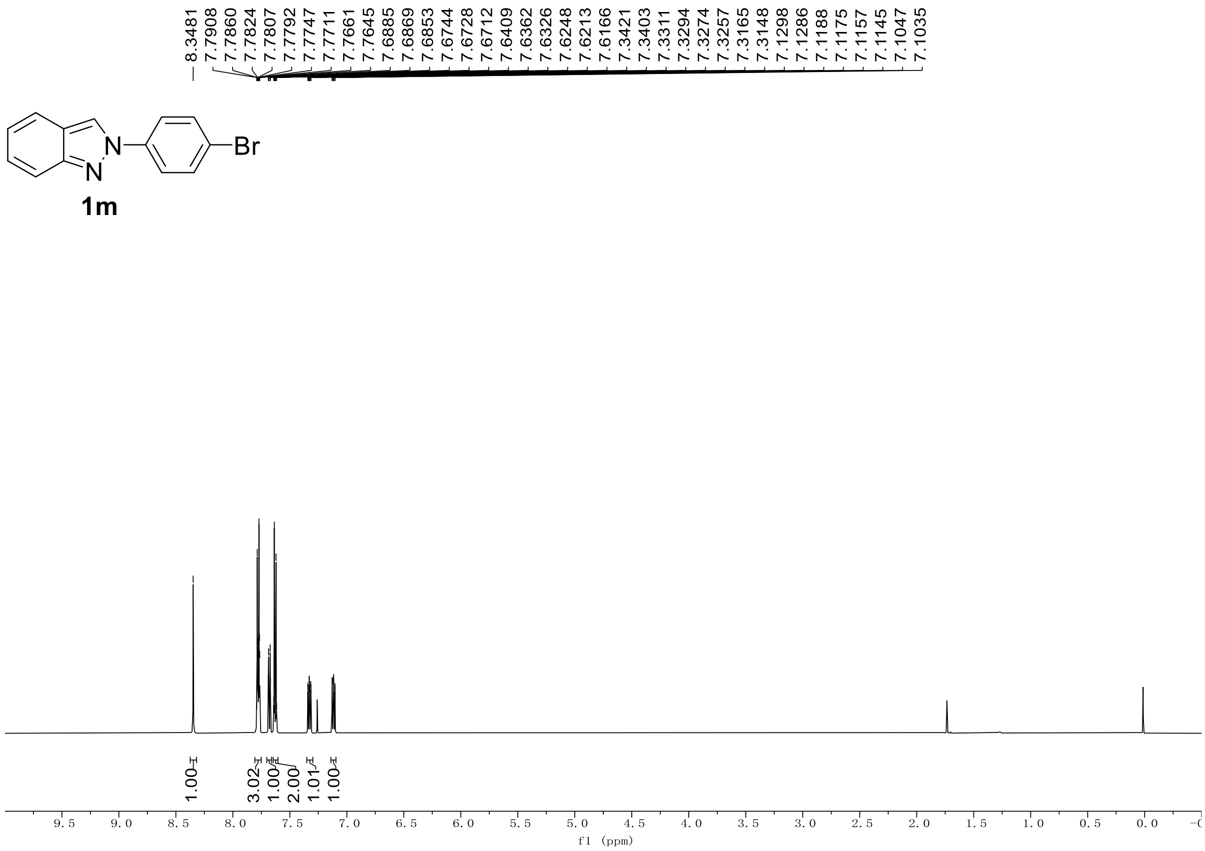

Figure S104 :^{1}H NMR spectrum of **1m** (600 MHz, $CDCl_3$)

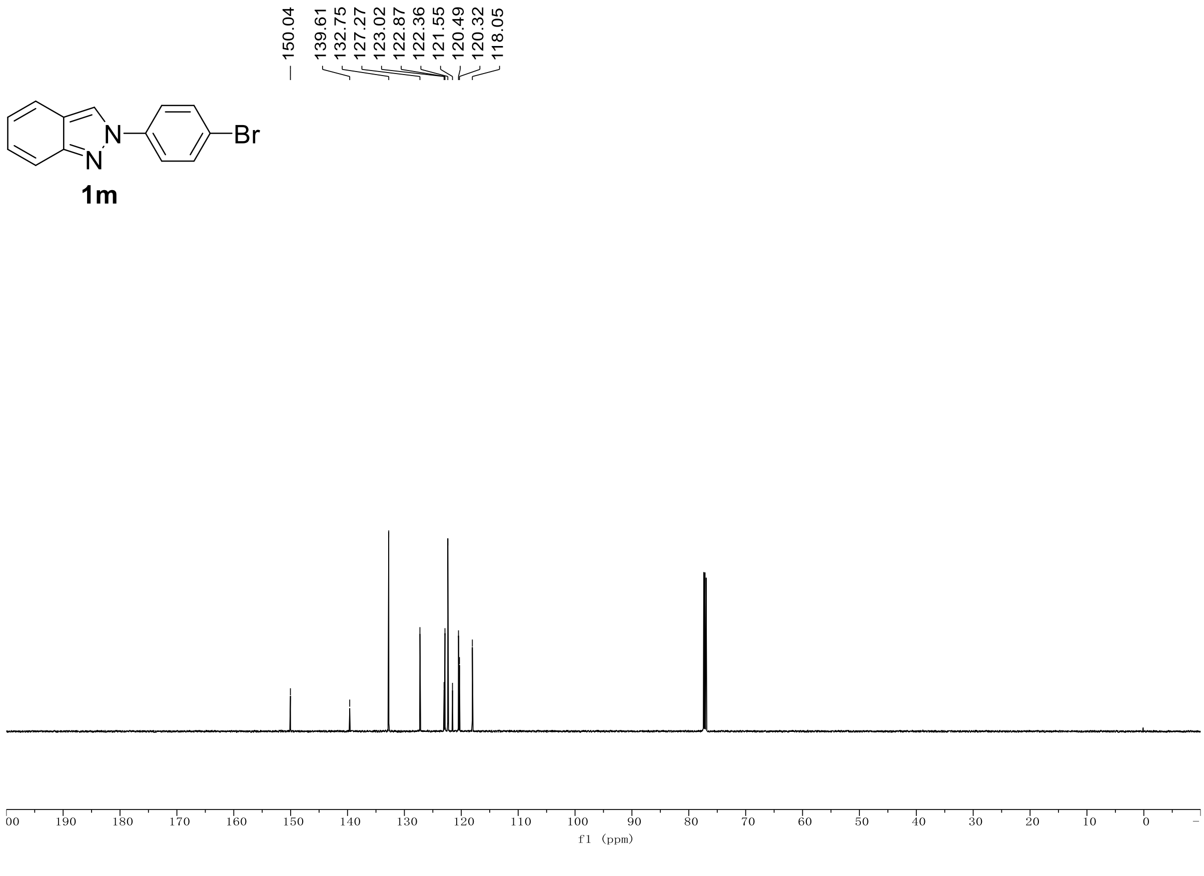

Figure S105 :^{13}C {^{1}H} NMR spectrum of **1m** (150 MHz, $CDCl_3$)

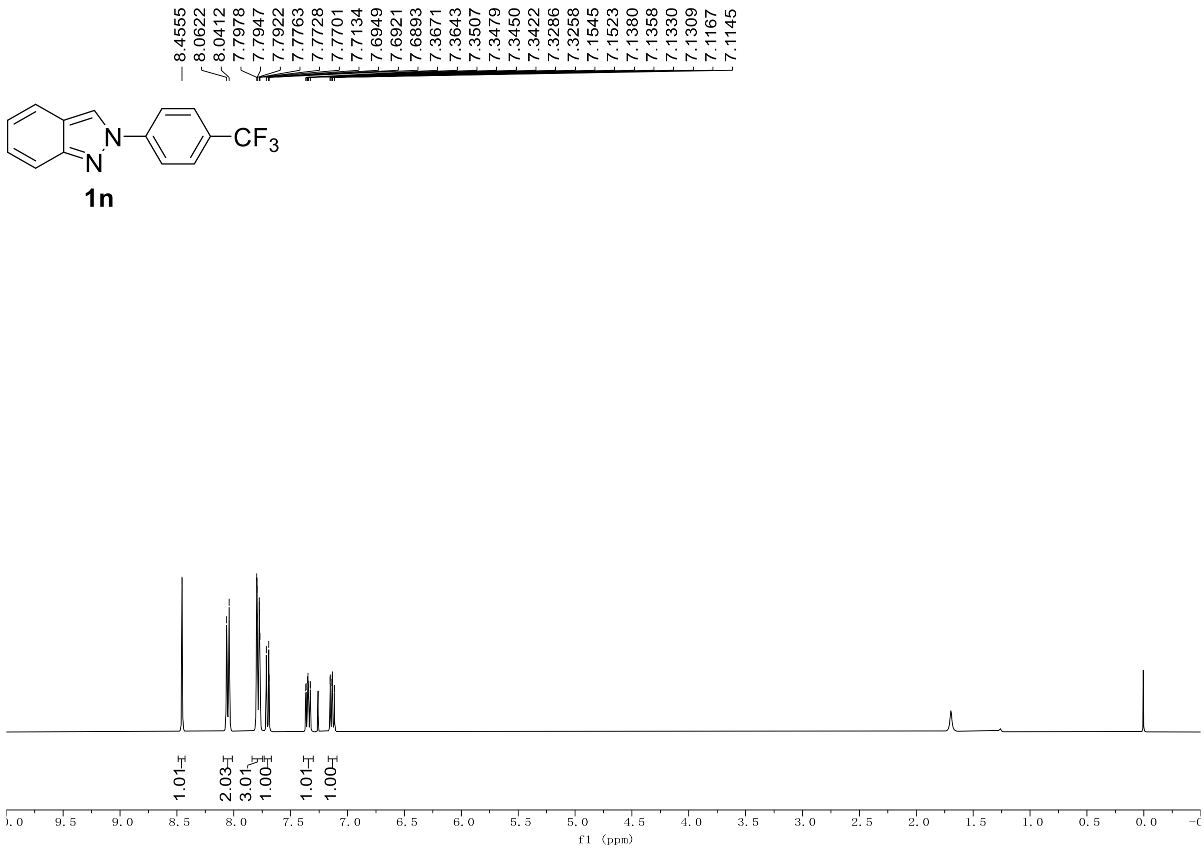

Figure S106 :^{1}H NMR spectrum of **1n** (400 MHz, $CDCl_3$)

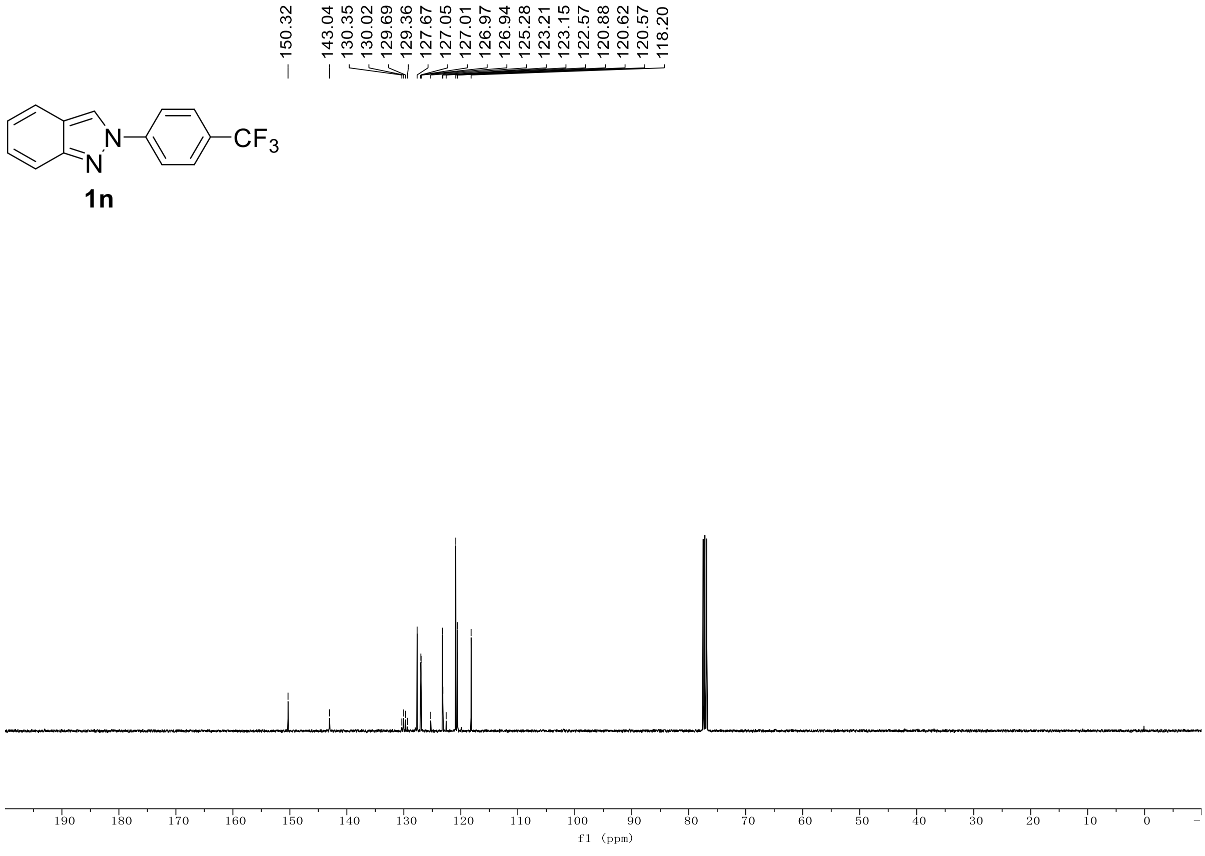

Figure S107 :^{13}C {^{1}H} NMR spectrum of **1n** (100 MHz, $CDCl_3$)

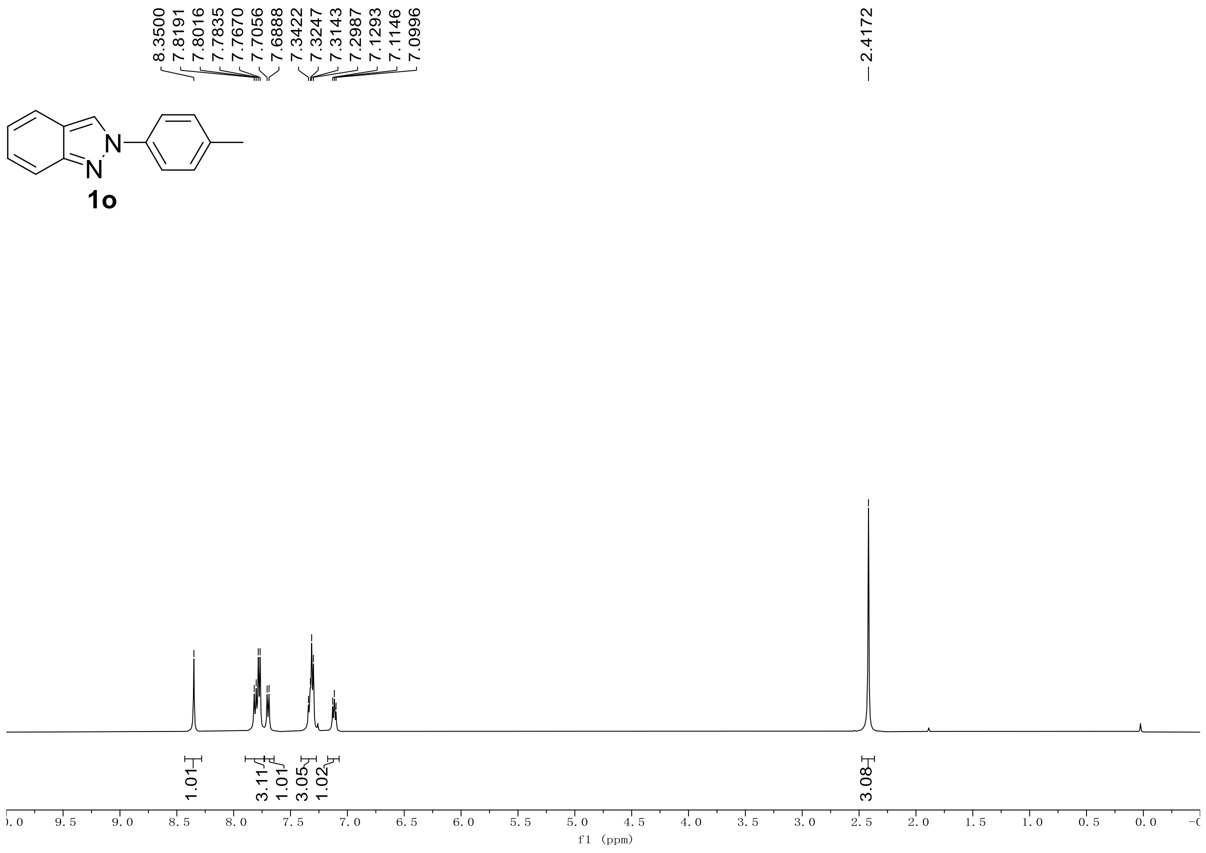

Figure S108 :^{1}H NMR spectrum of **1o** (500 MHz, $CDCl_3$)

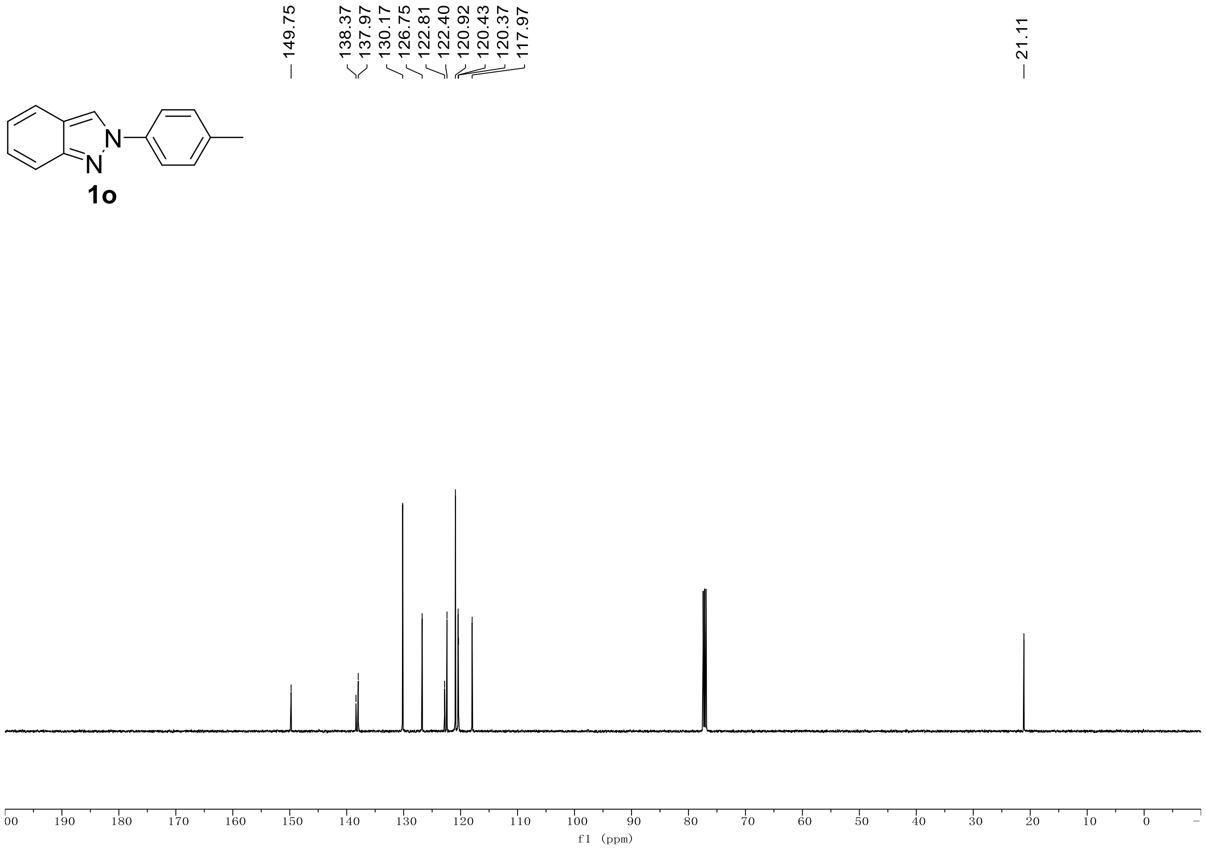

Figure S109 :^{13}C {^{1}H} NMR spectrum of **1o** (125 MHz, $CDCl_3$)

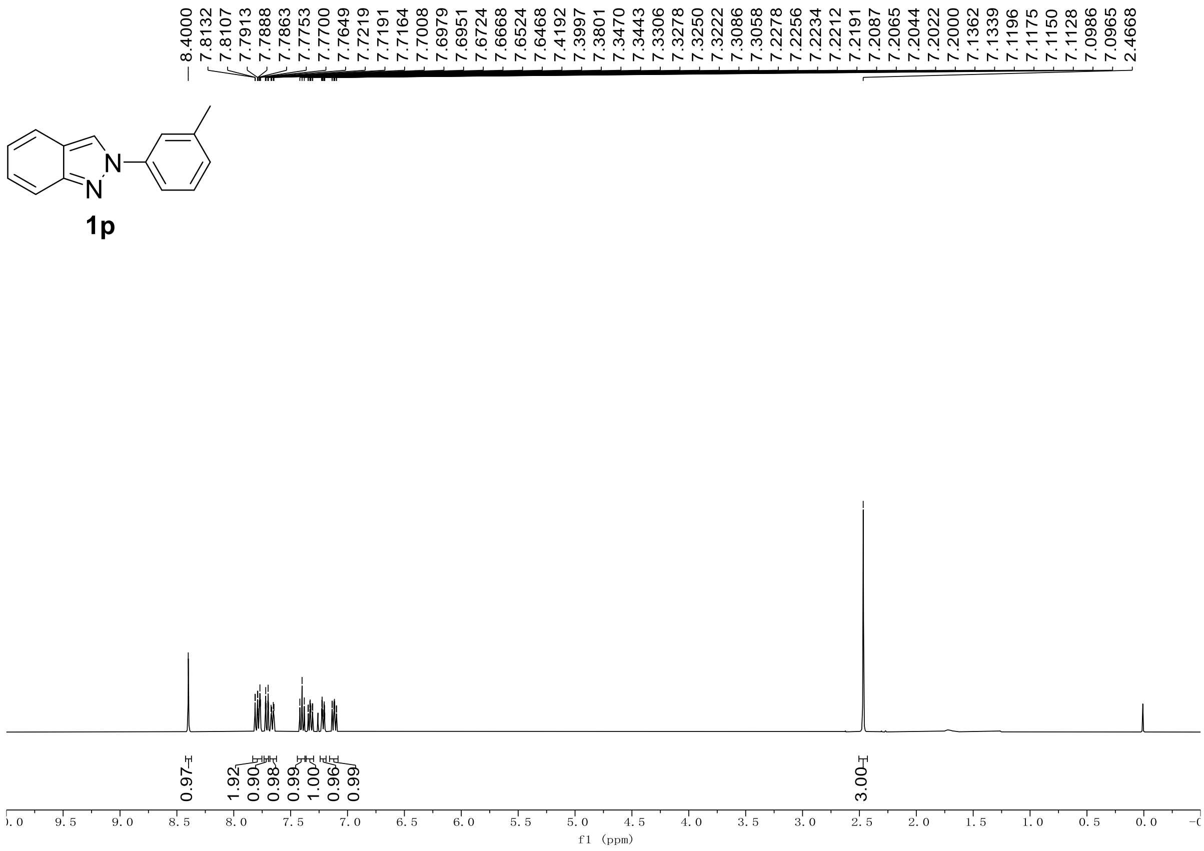

Figure S110 :^{1}H NMR spectrum of **1p** (400 MHz, $CDCl_3$)

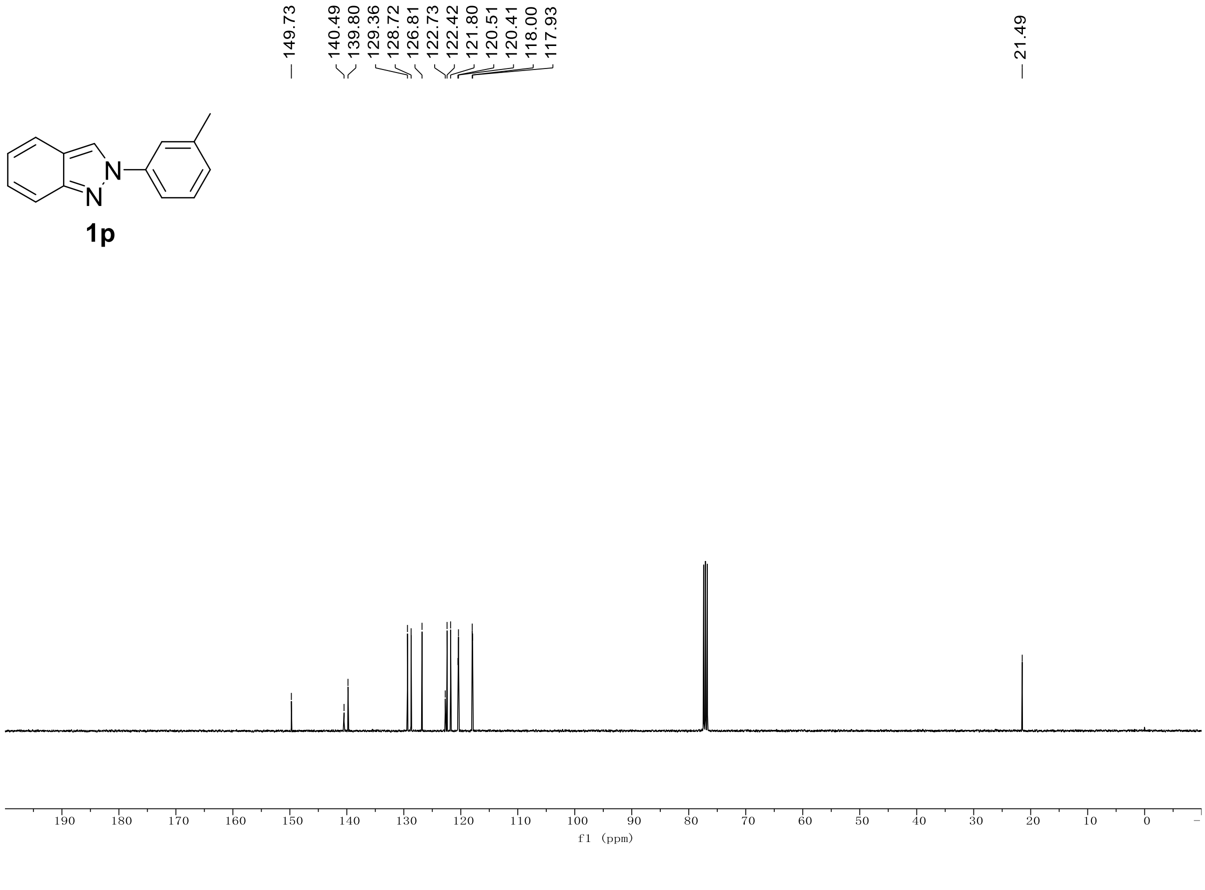

Figure S111 :^{13}C {^{1}H} NMR spectrum of **1p** (100 MHz, $CDCl_3$)

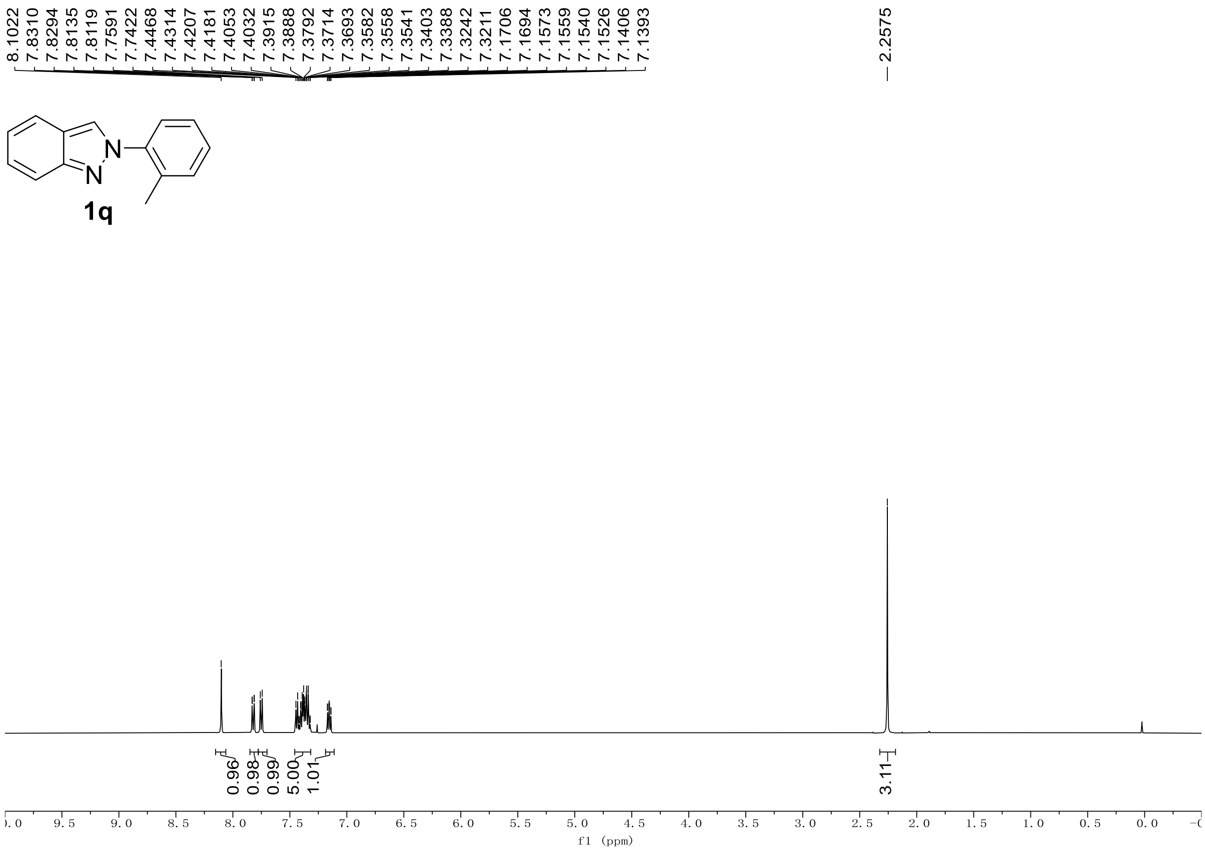

Figure S112 :^{1}H NMR spectrum of **1q** (500 MHz, $CDCl_3$)

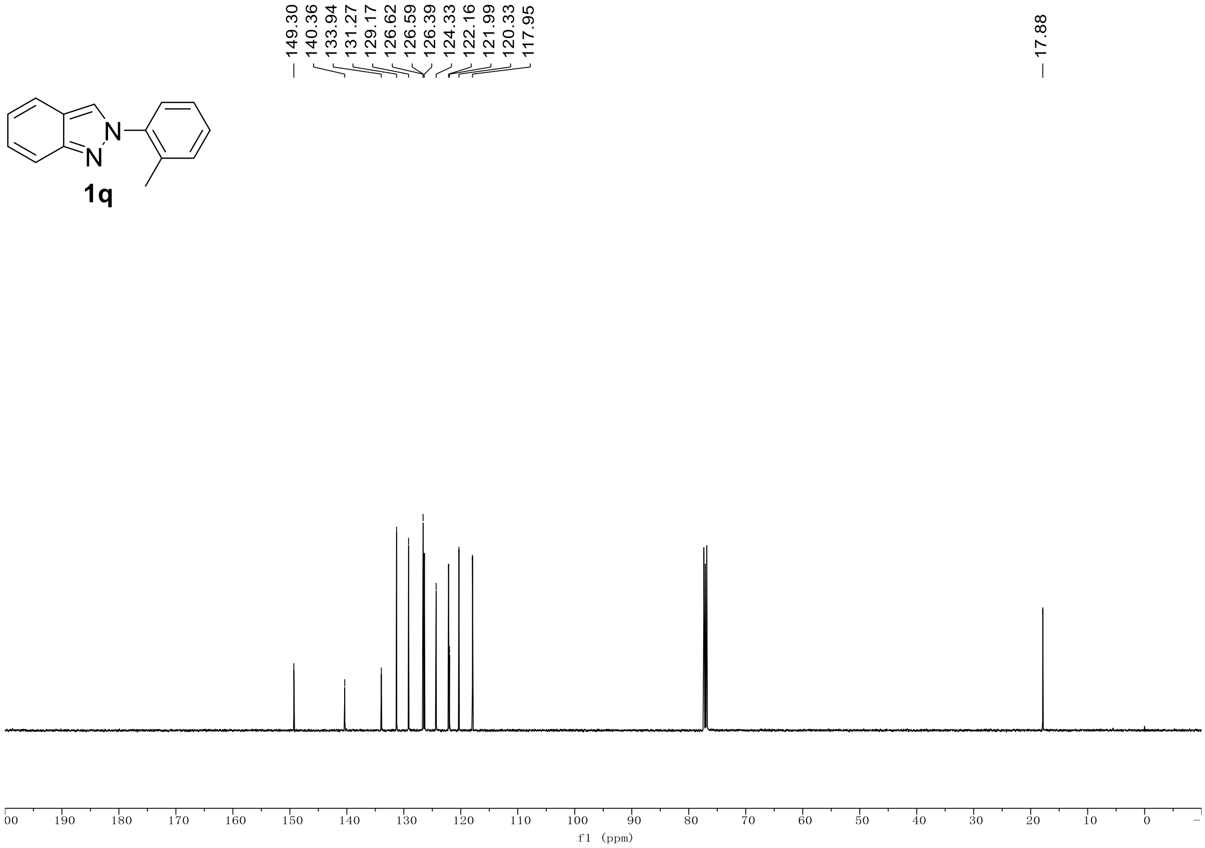

Figure S113 :^{13}C {^{1}H} NMR spectrum of **1q** (125 MHz, $CDCl_3$)

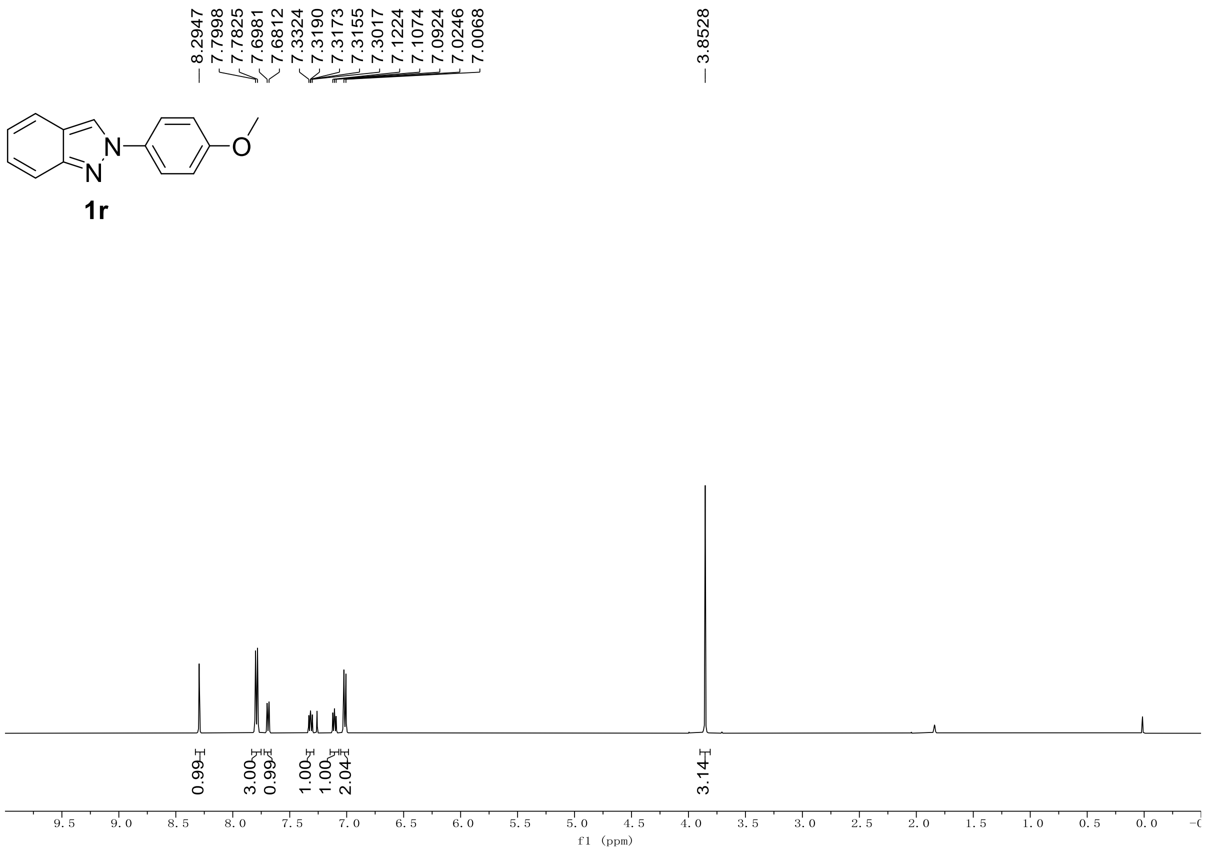

Figure S114 :¹H NMR spectrum of **1r** (500 MHz, $CDCl_3$)

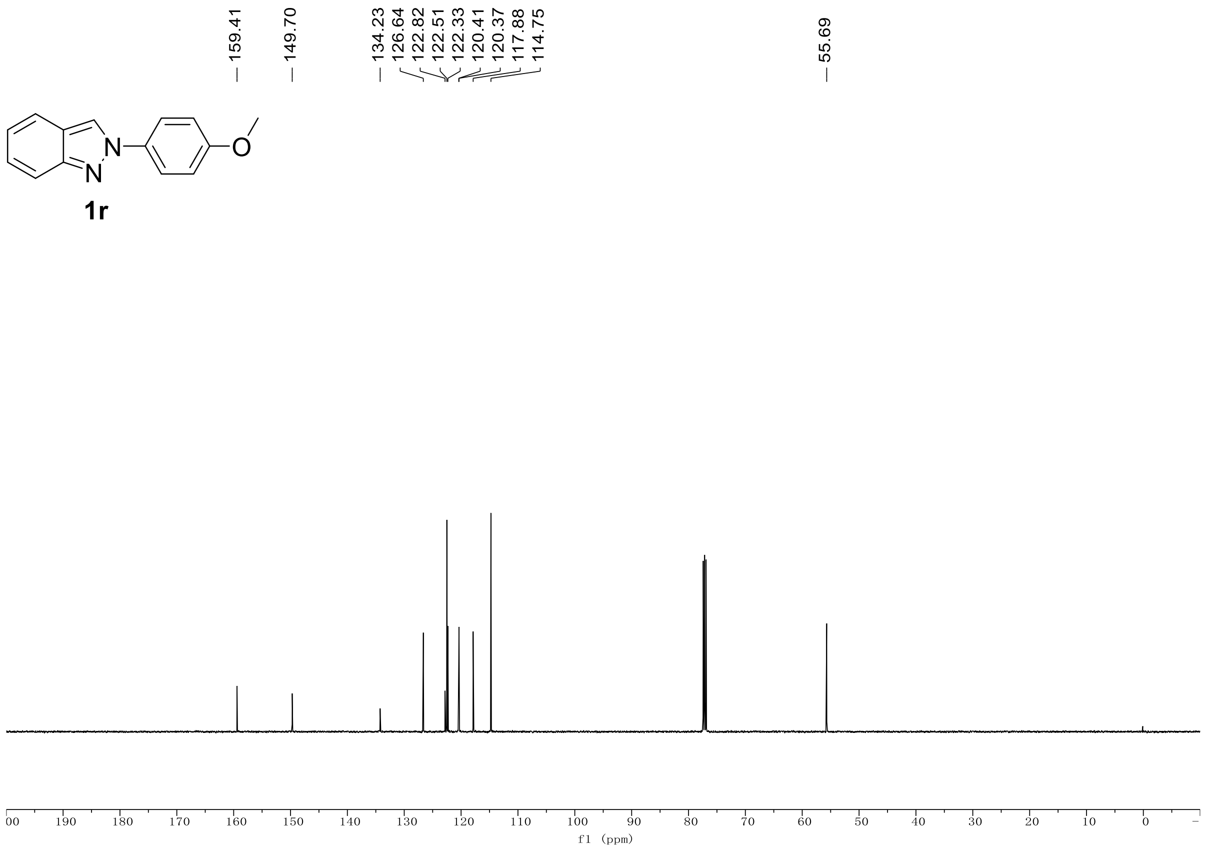

Figure S115 :^{13}C {¹H} NMR spectrum of **1r** (125 MHz, $CDCl_3$)

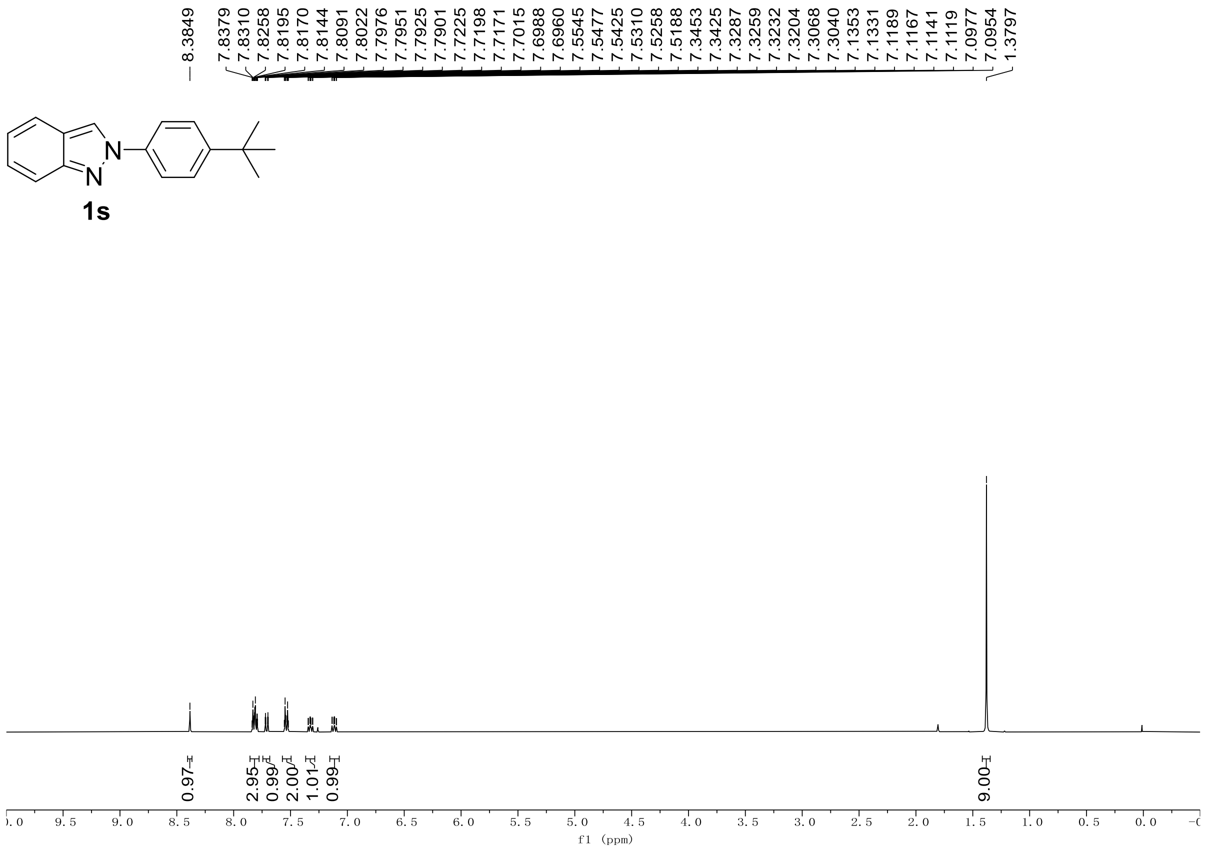

Figure S116 :^{1}H NMR spectrum of **1s** (400 MHz, $CDCl_3$)

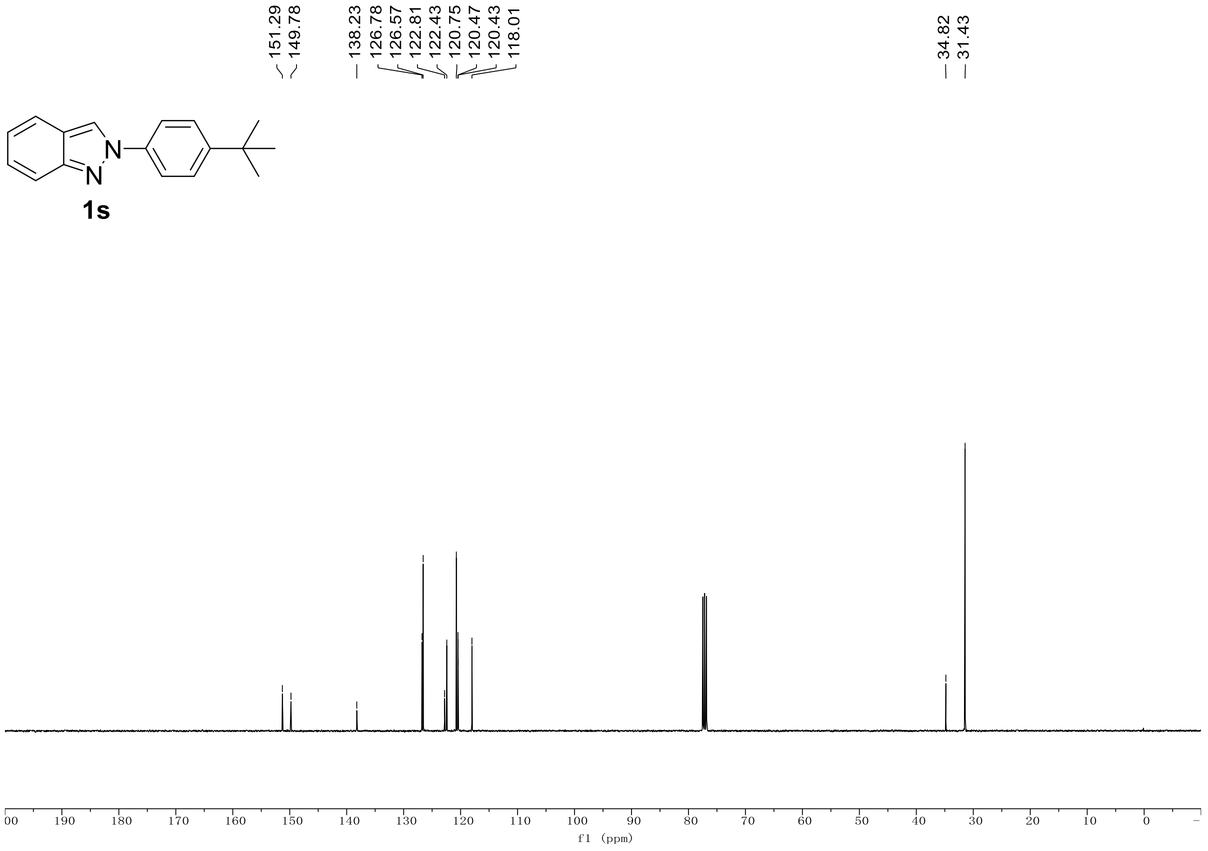

Figure S117 :^{13}C {^{1}H} NMR spectrum of **1s** (100 MHz, $CDCl_3$)

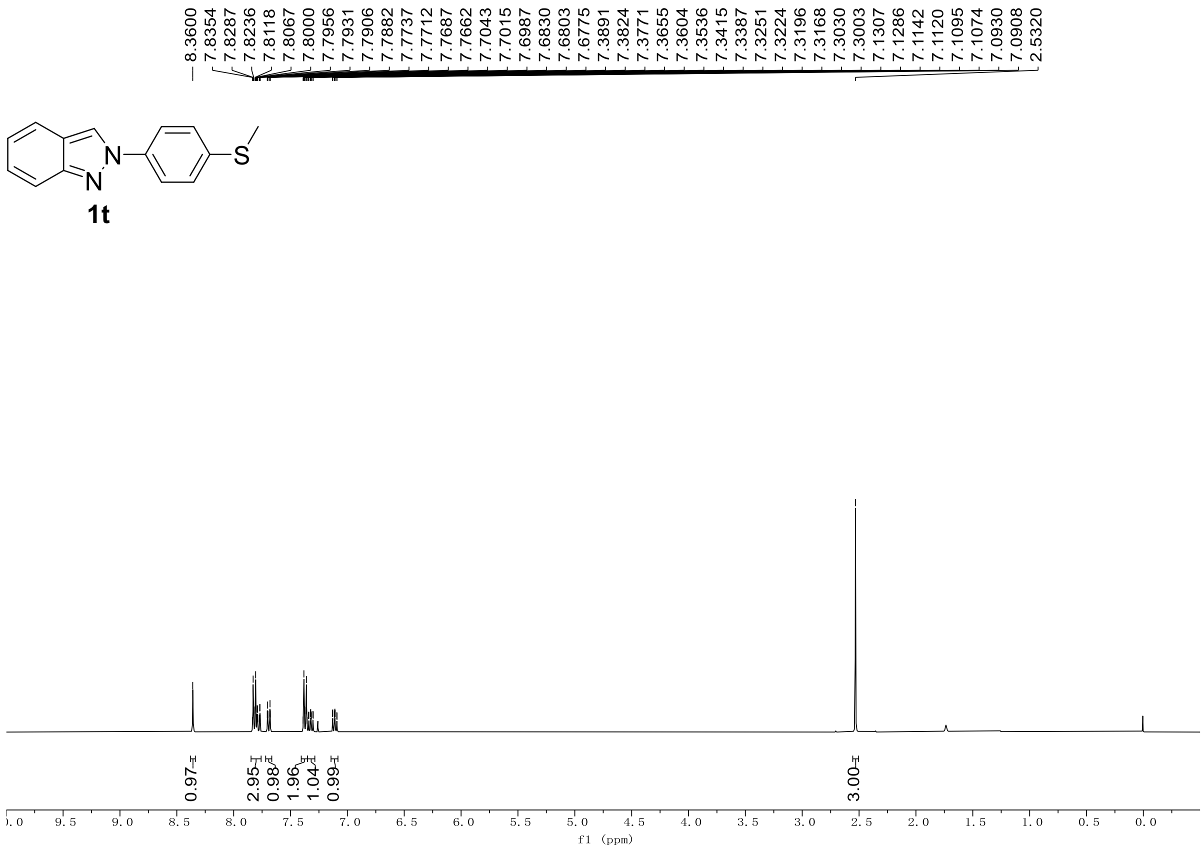

Figure S118 :^{1}H NMR spectrum of **1t** (400 MHz, $CDCl_3$)

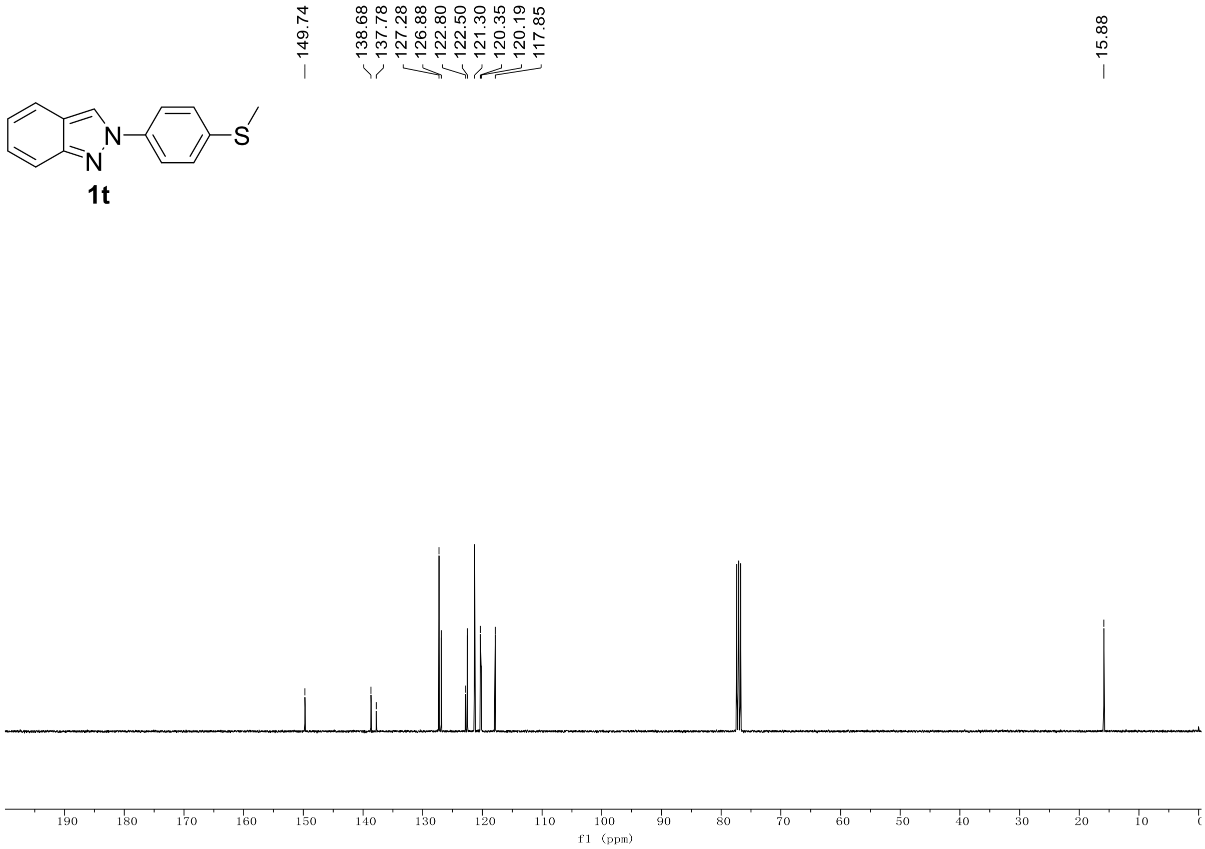

Figure S119 :^{13}C {^{1}H} NMR spectrum of **1t** (100 MHz, $CDCl_3$)

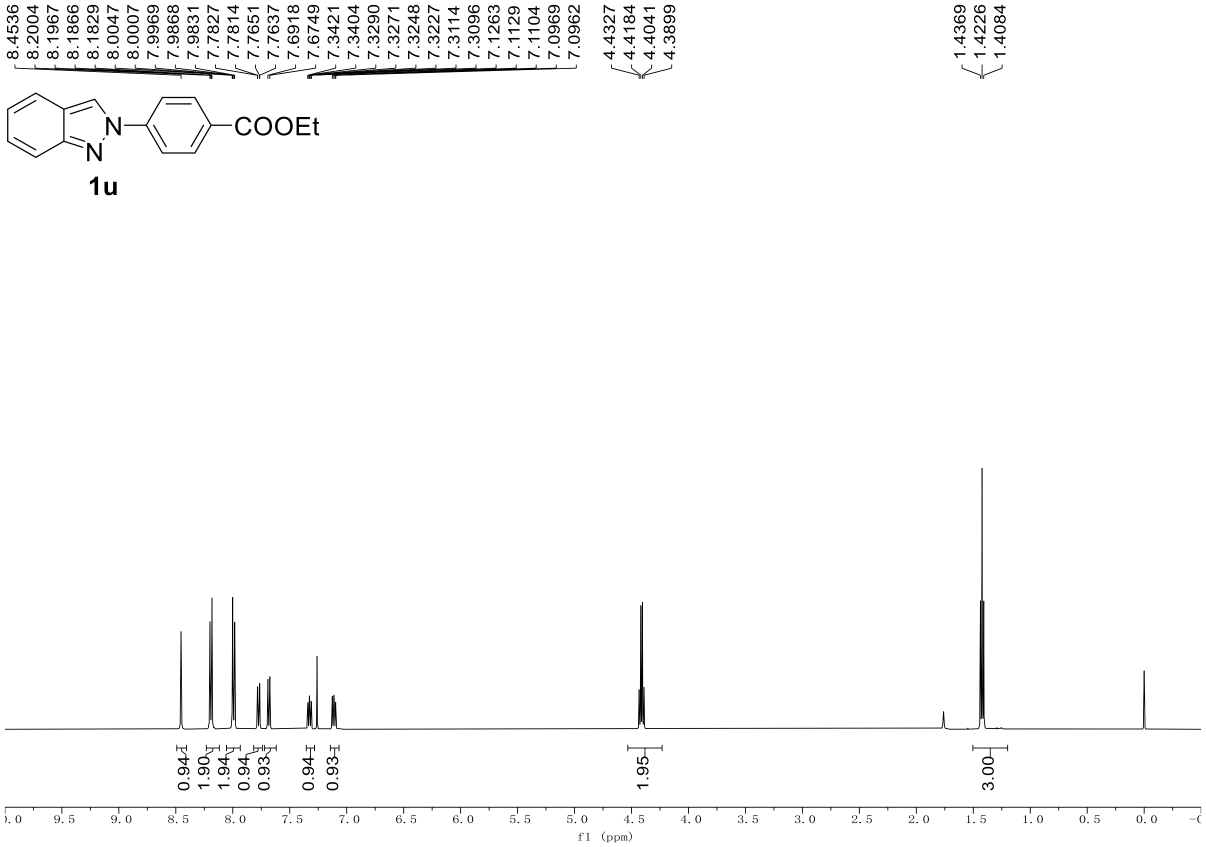

Figure S120 :^{1}H NMR spectrum of **1u** (500 MHz, $CDCl_3$)

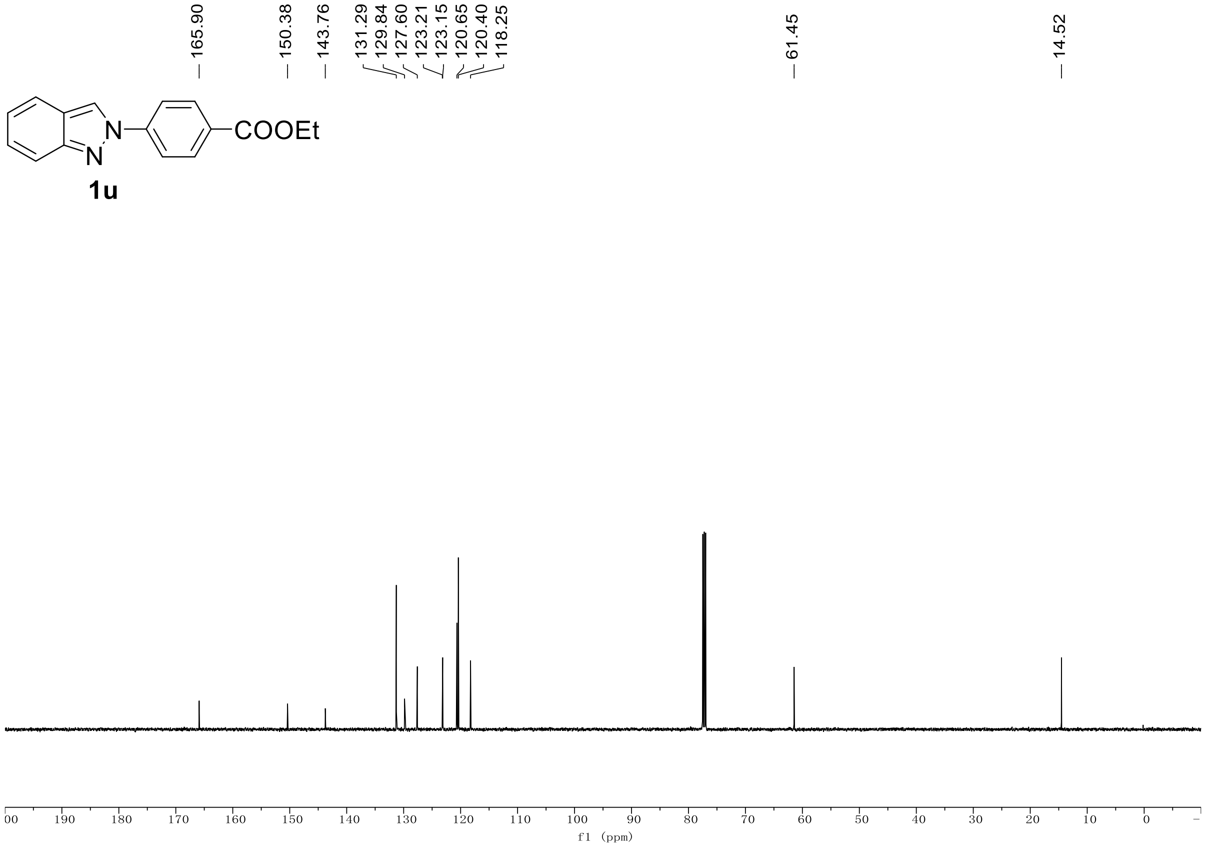

Figure S121 :^{13}C {^{1}H} NMR spectrum of **1u** (125 MHz, $CDCl_3$)

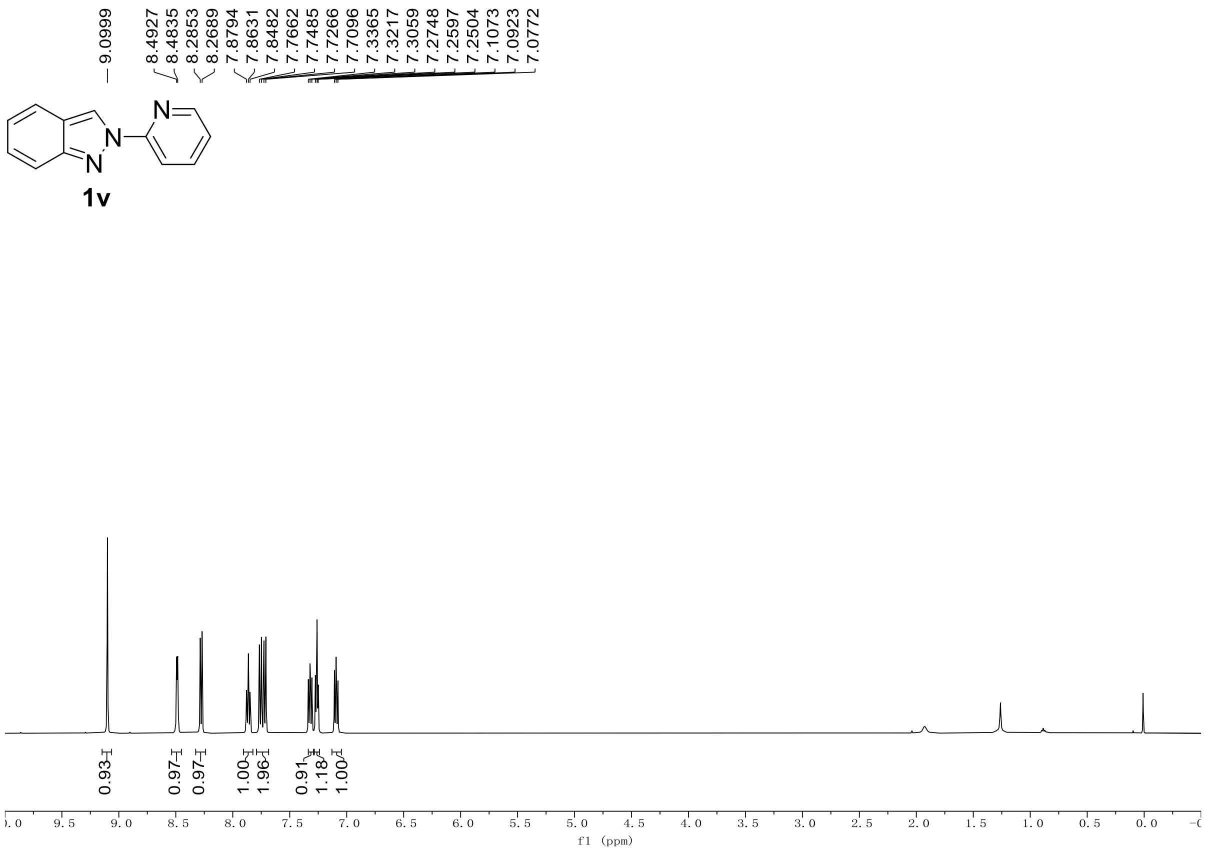

Figure S122 :^{1}H NMR spectrum of **1v** (500 MHz, $CDCl_3$)

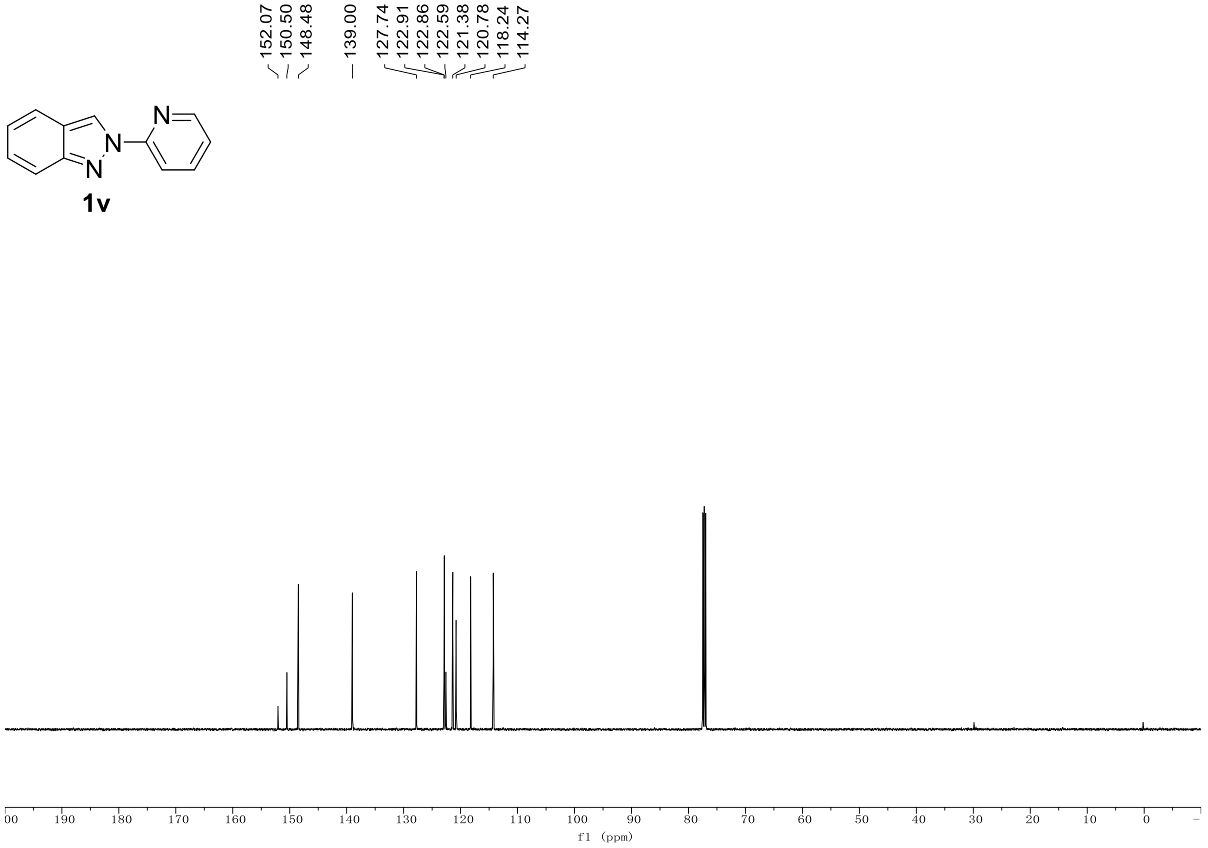

Figure S123 :^{13}C {^{1}H} NMR spectrum of **1v** (125 MHz, $CDCl_3$)

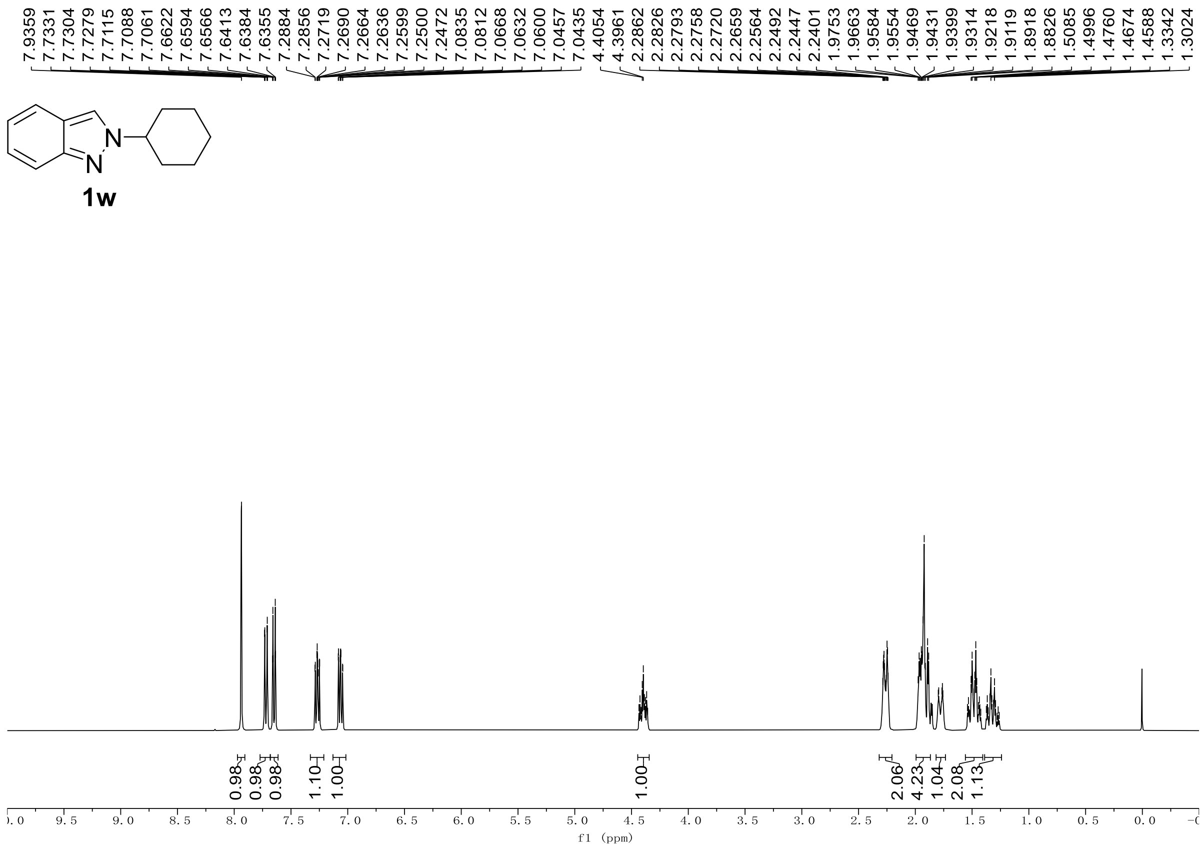

Figure S124 :^{1}H NMR spectrum of **1w** (400 MHz, $CDCl_3$)

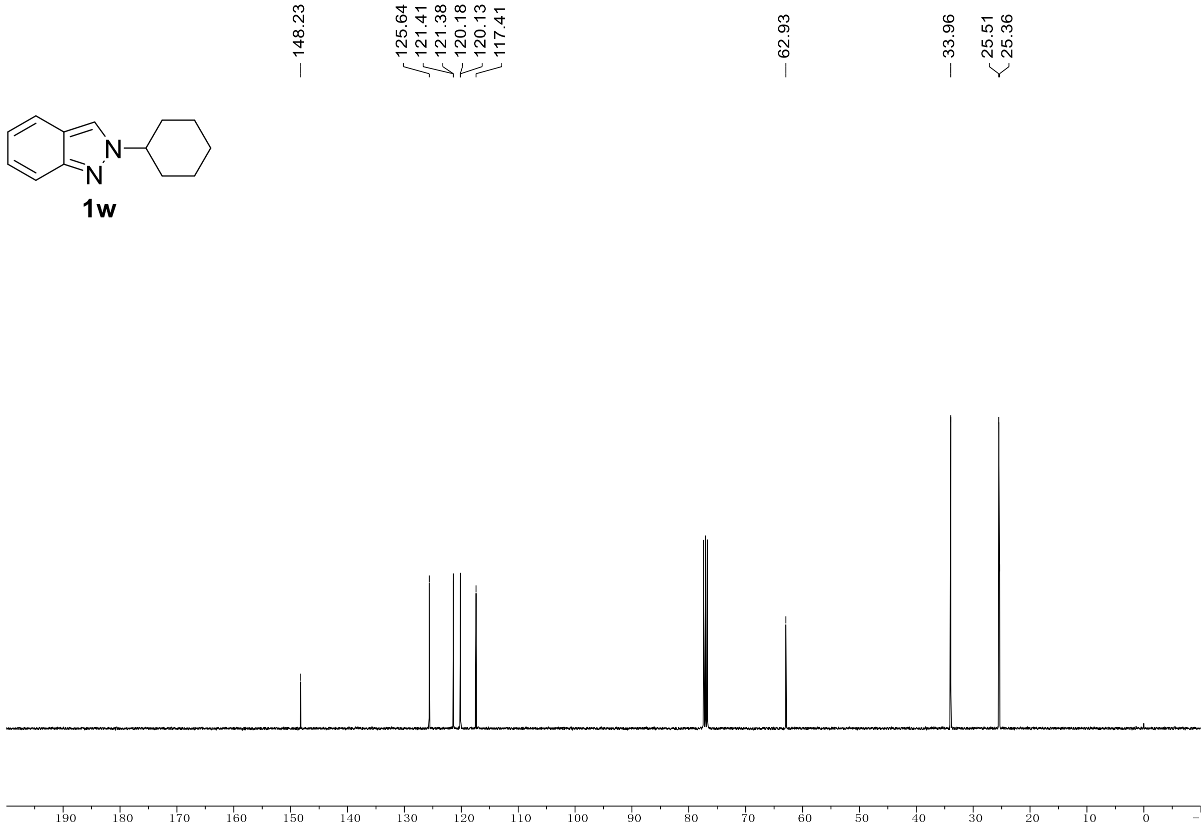

Figure S125 :^{13}C {^{1}H} NMR spectrum of **1w** (100 MHz, $CDCl_3$)

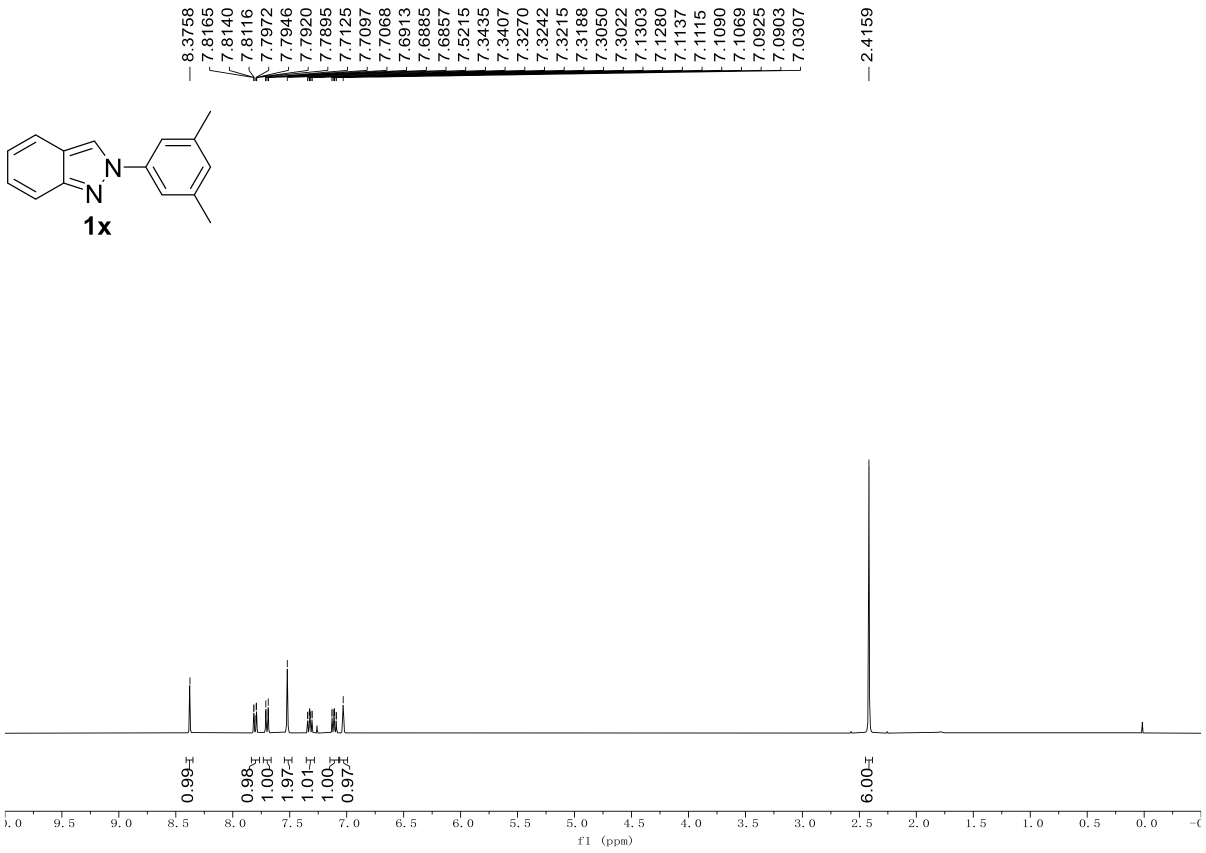

Figure S126 :^{1}H NMR spectrum of **1x** (400 MHz, $CDCl_3$)

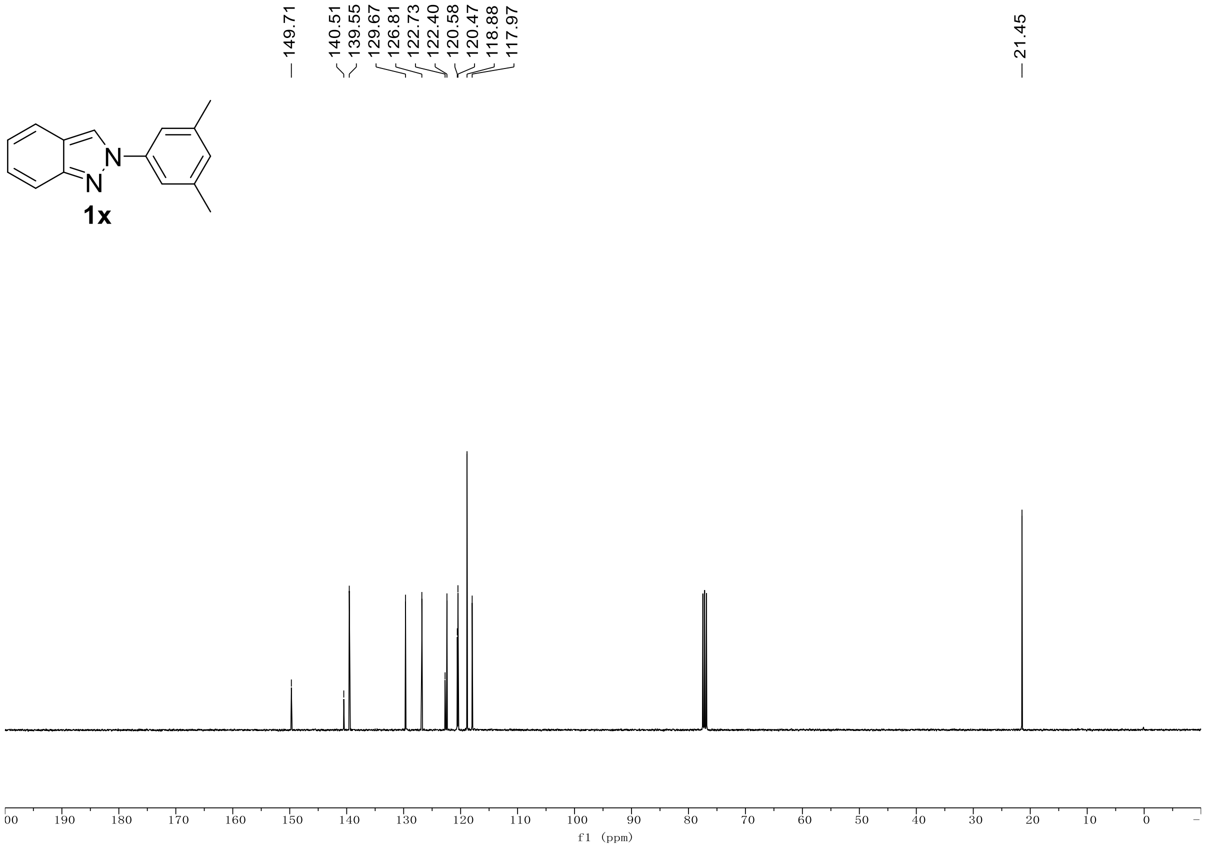

Figure S127 :^{13}C {^{1}H} NMR spectrum of **1x** (100 MHz, $CDCl_3$)

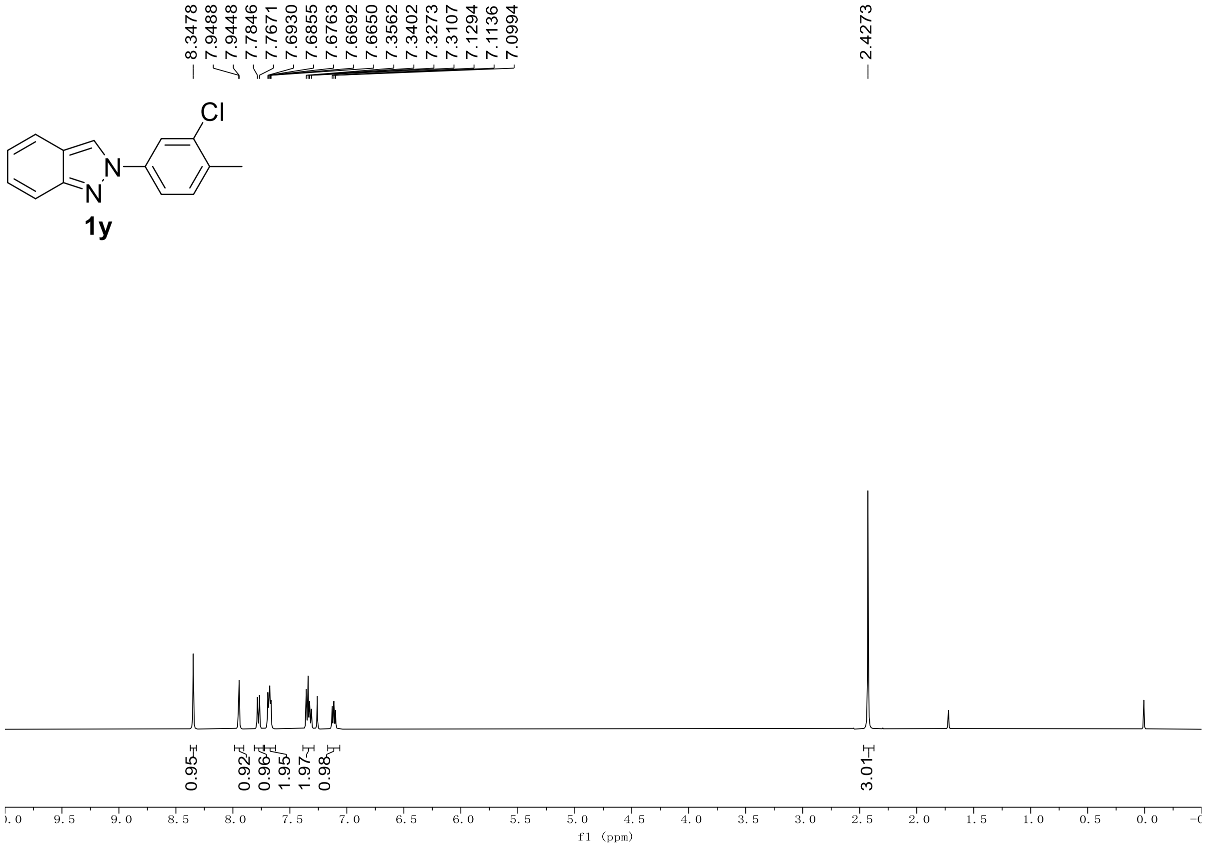

Figure S128 :^{1}H NMR spectrum of **1y** (500 MHz, $CDCl_3$)

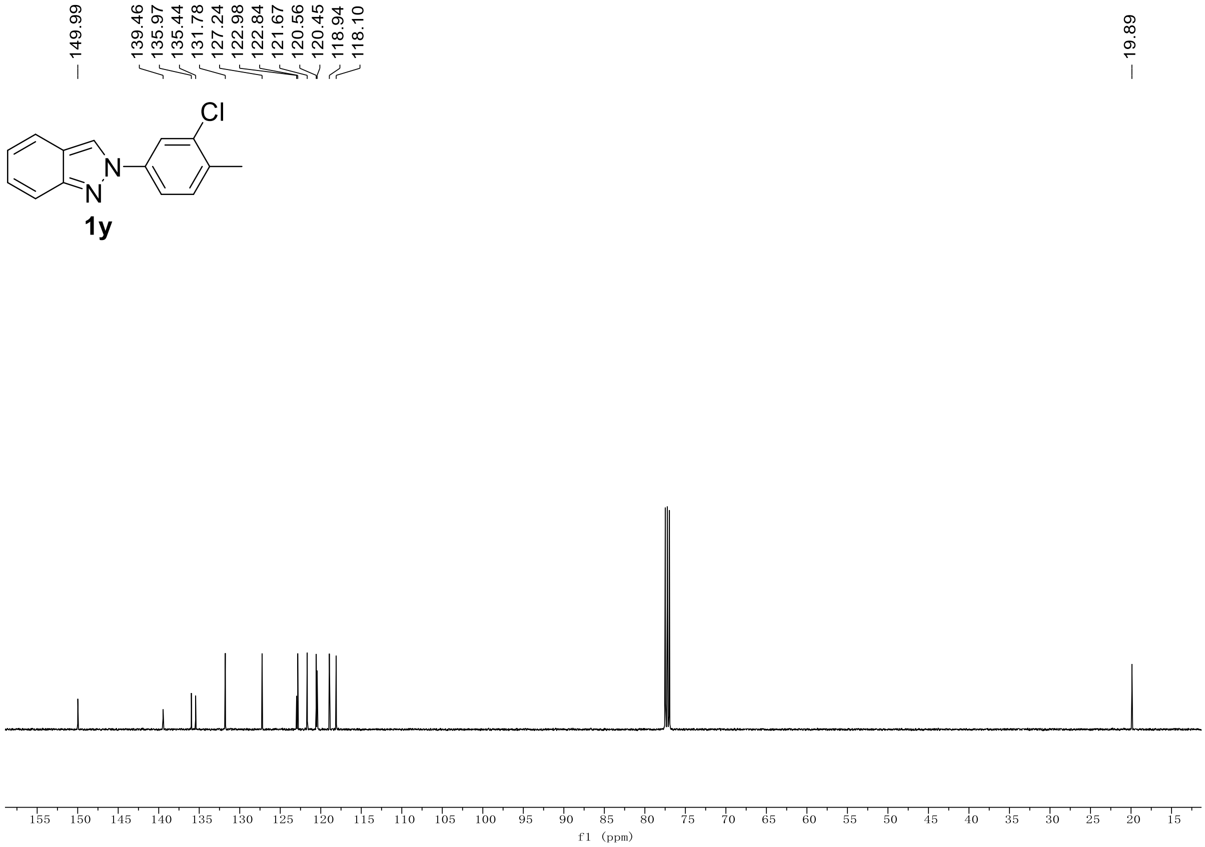

Figure S129 :^{13}C {^{1}H} NMR spectrum of **1y** (125 MHz, $CDCl_3$)

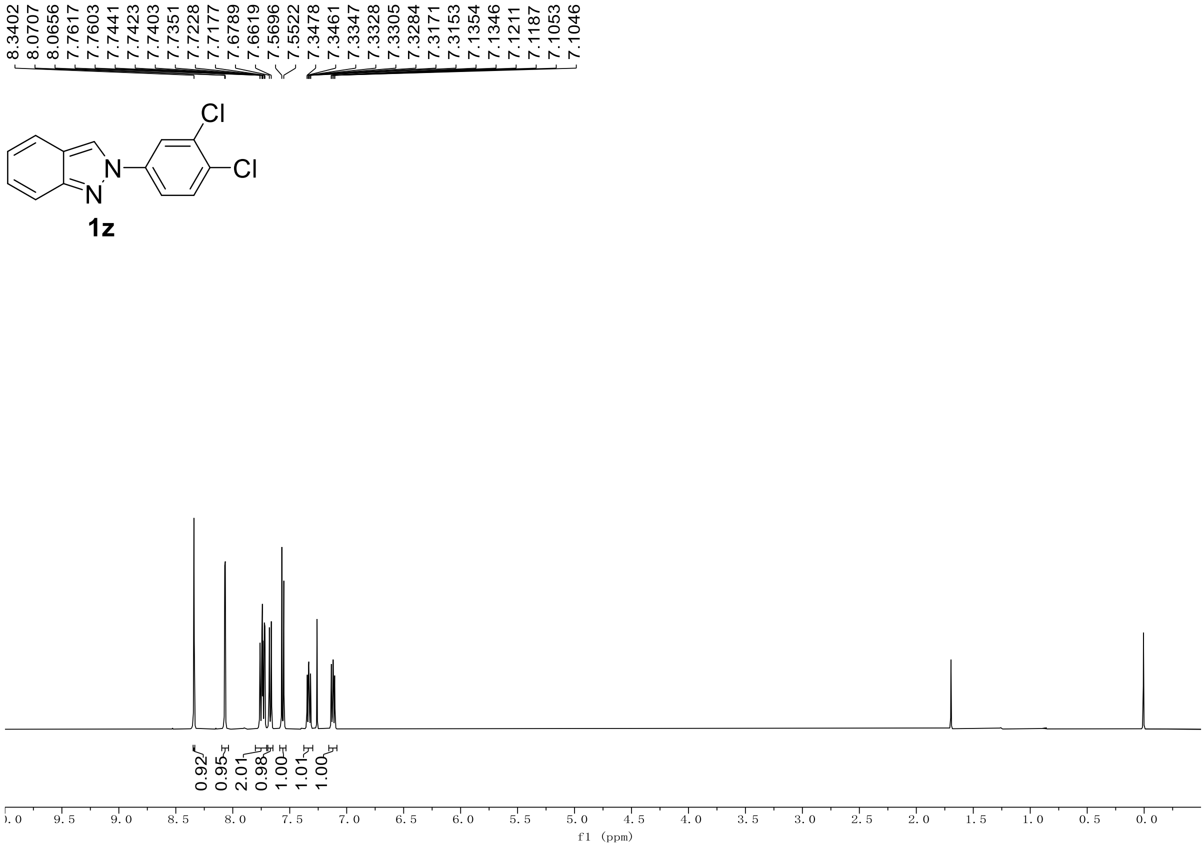

Figure S130 :^{1}H NMR spectrum of **1z** (500 MHz, $CDCl_3$)

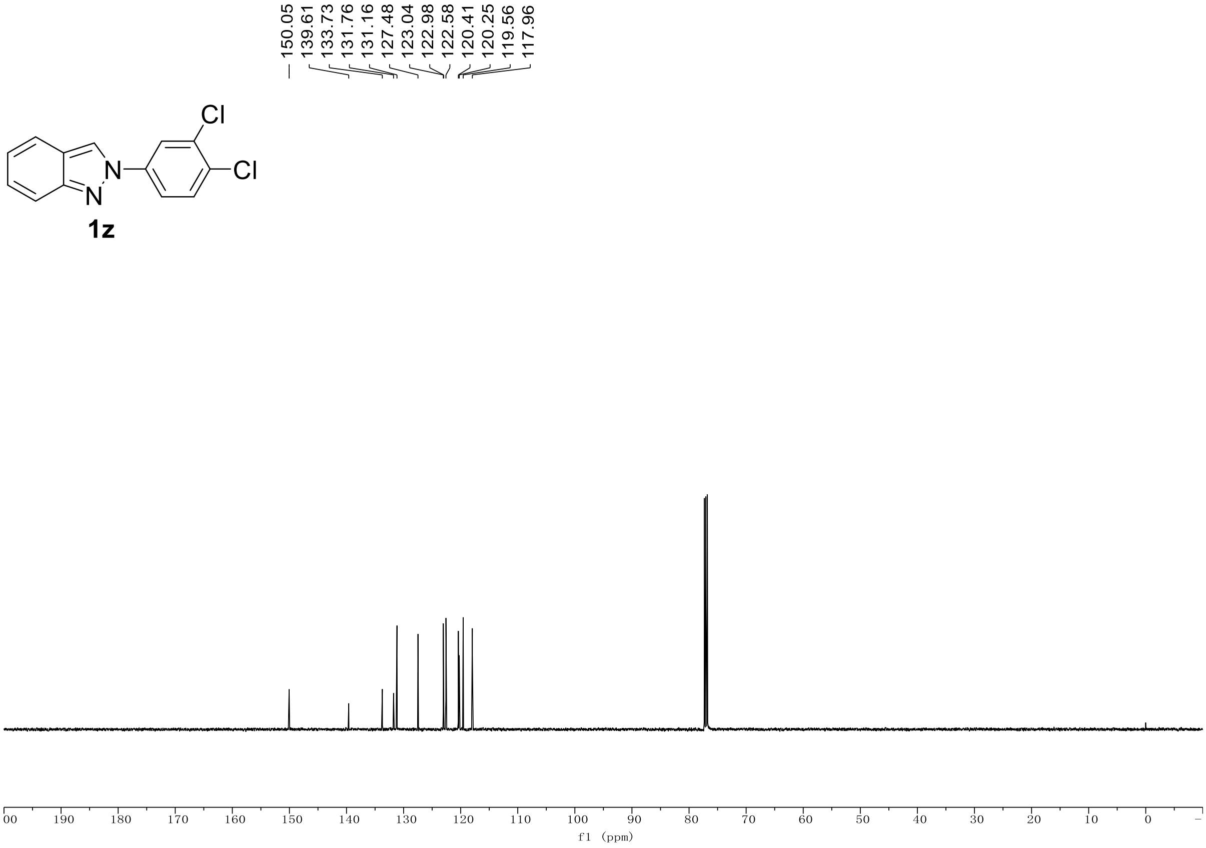

Figure S131 :^{13}C {^{1}H} NMR spectrum of **1z** (125 MHz, $CDCl_3$)

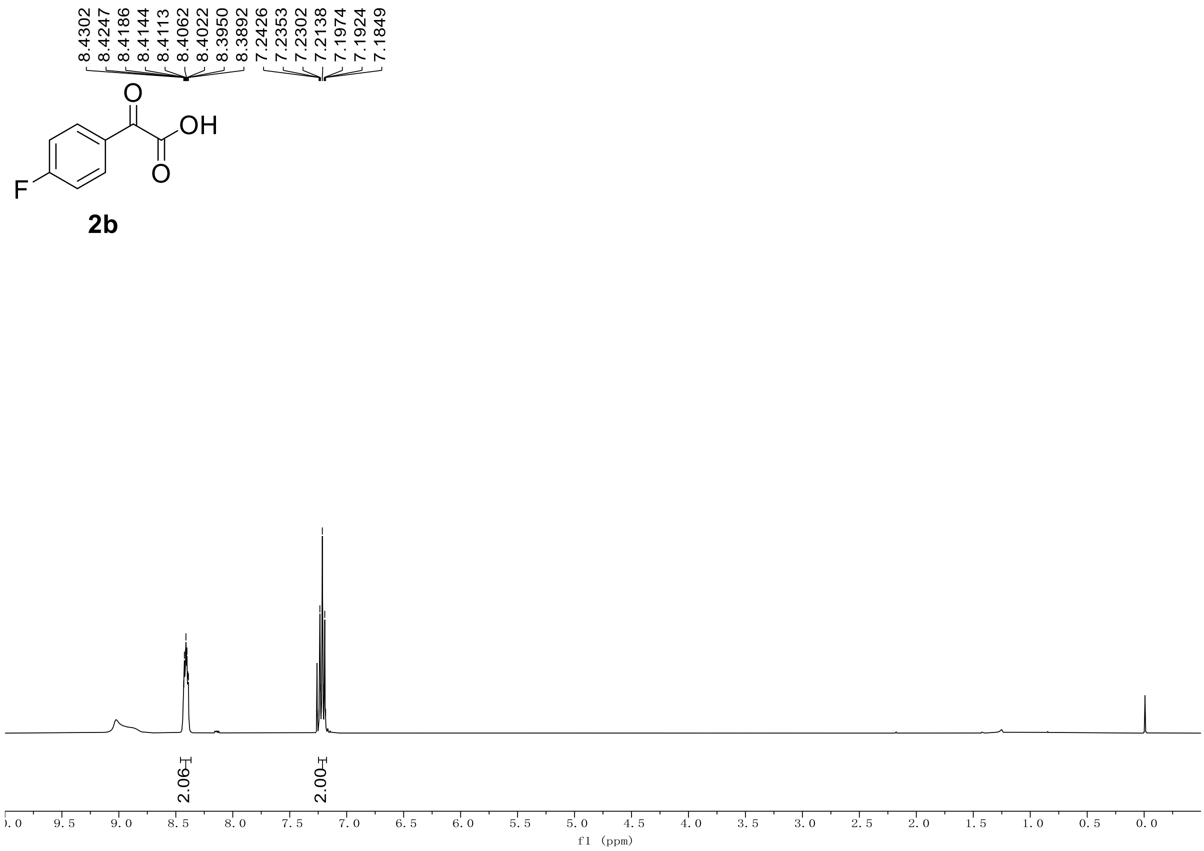

Figure S132 :^{1}H NMR spectrum of **2b** (400 MHz, $CDCl_3$)

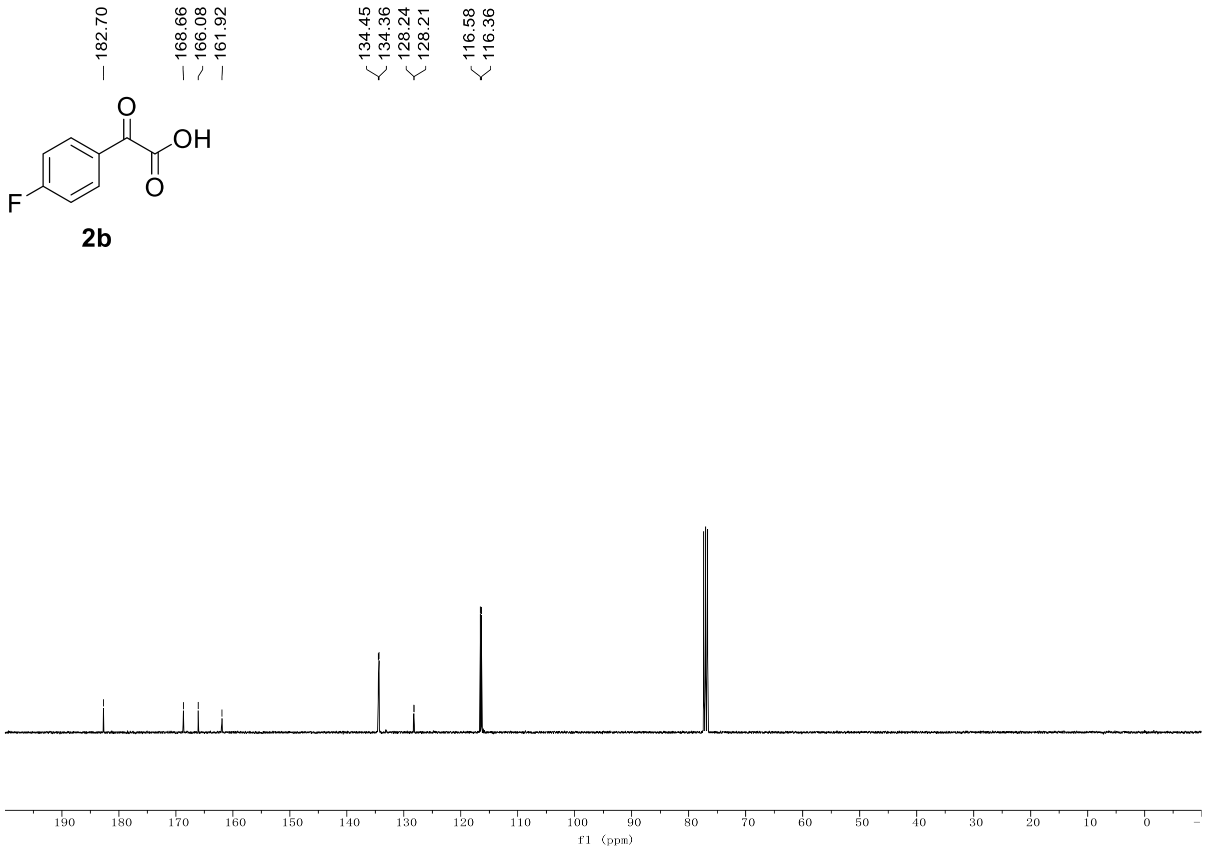

Figure S133 :^{13}C {^{1}H} NMR spectrum of **2b** (100 MHz, $CDCl_3$)

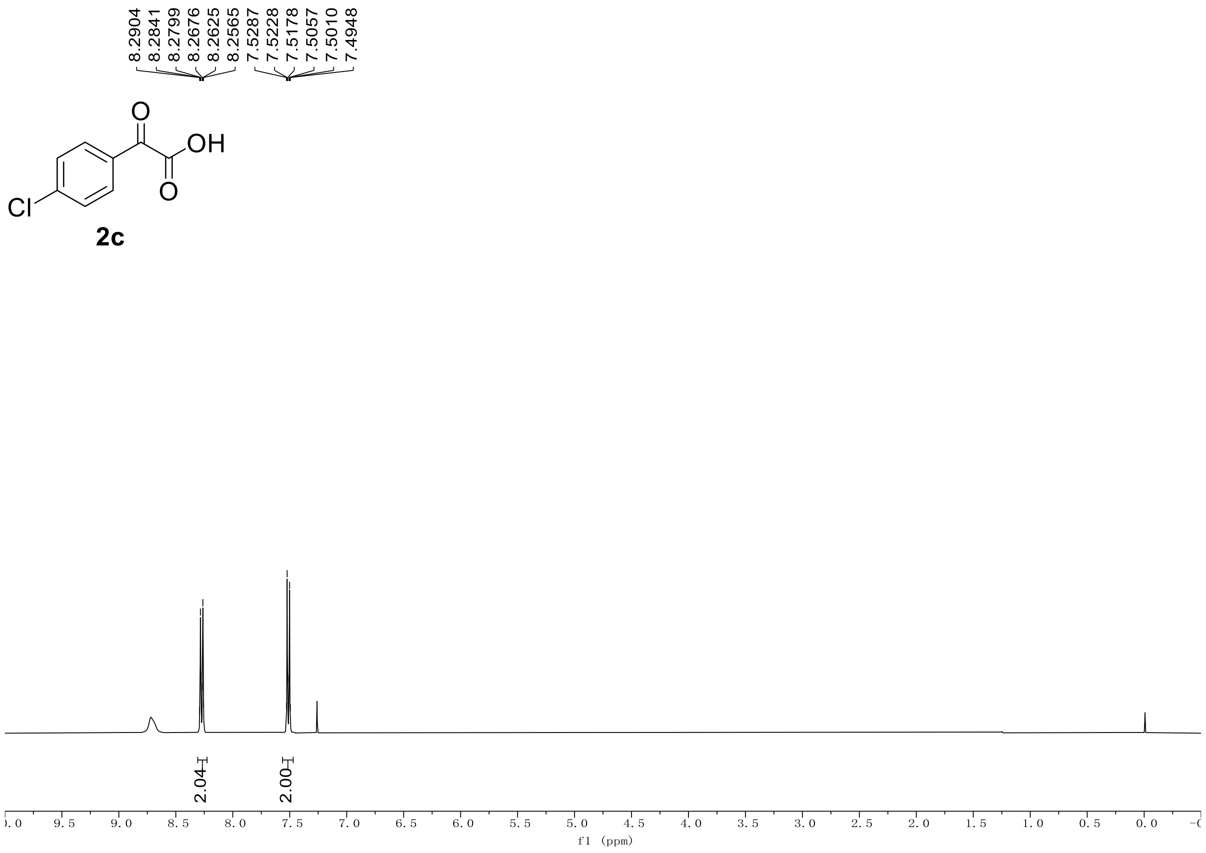

Figure S134 :^{1}H NMR spectrum of **2c** (400 MHz, $CDCl_3$)

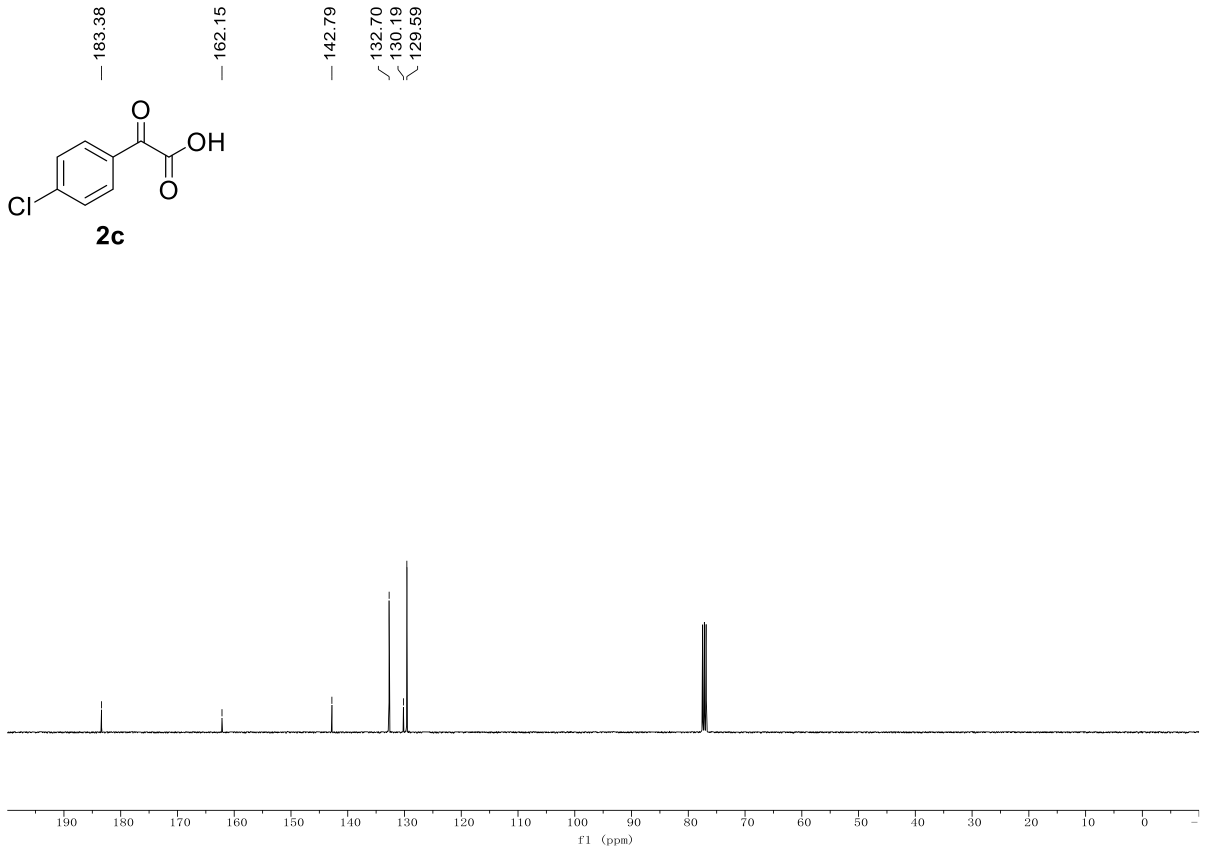

Figure S135 :^{13}C {^{1}H} NMR spectrum of **2c** (100 MHz, $CDCl_3$)

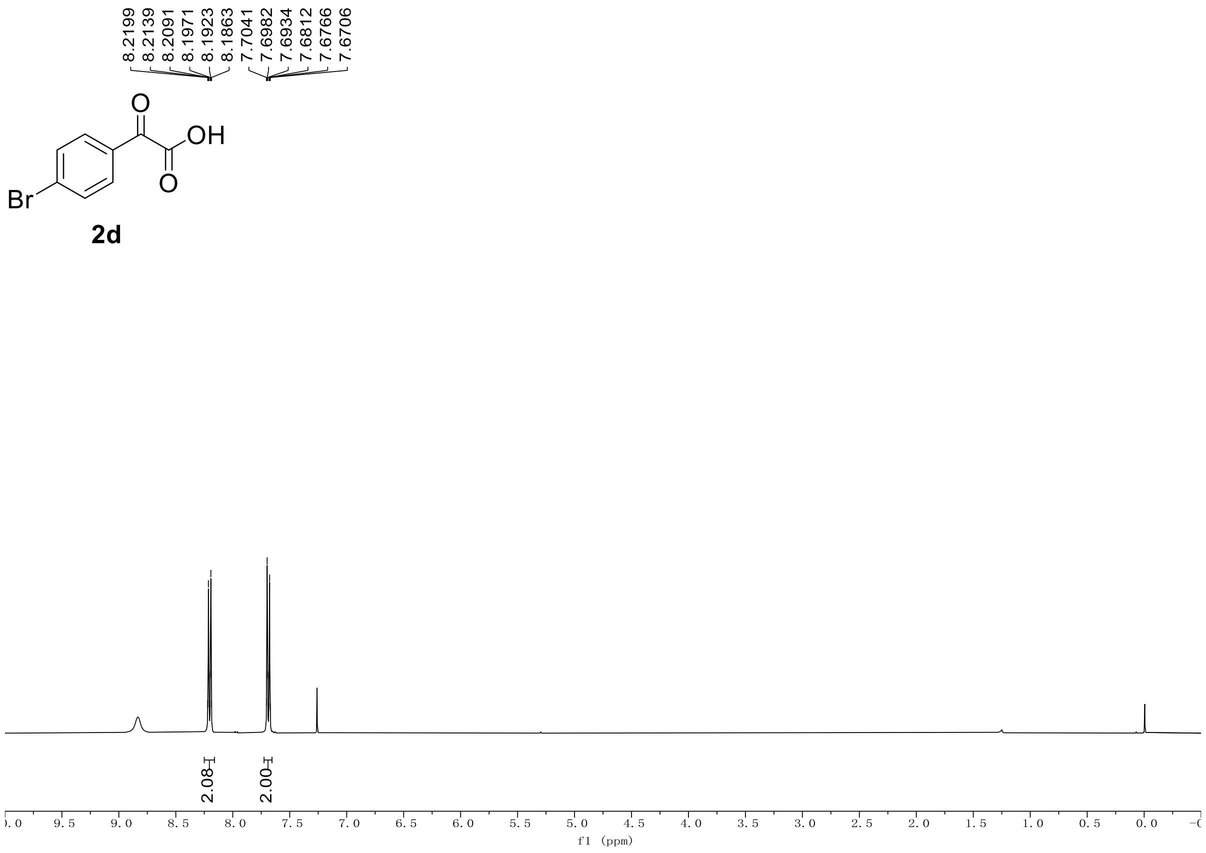

Figure S136 :^{1}H NMR spectrum of **2d** (400 MHz, $CDCl_3$)

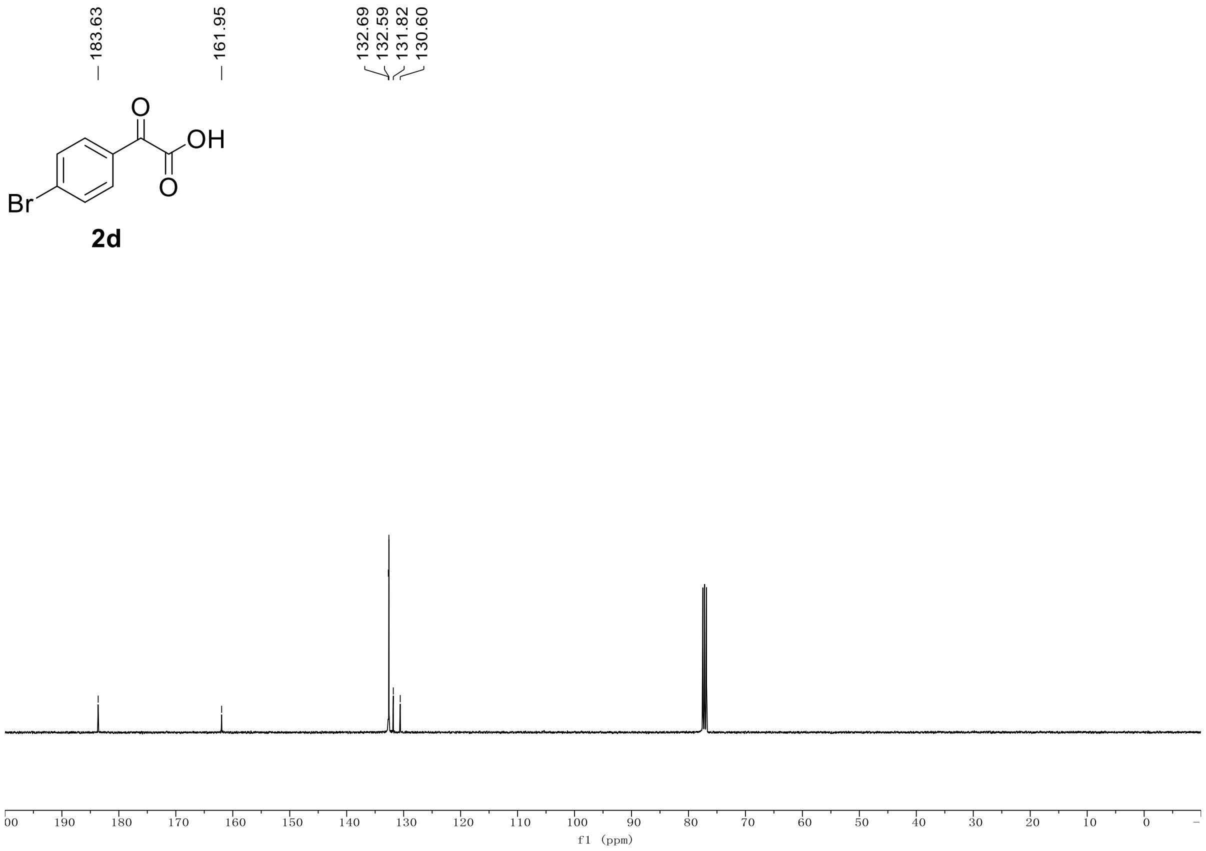

Figure S137 :^{13}C {^{1}H} NMR spectrum of **2d** (100 MHz, $CDCl_3$)

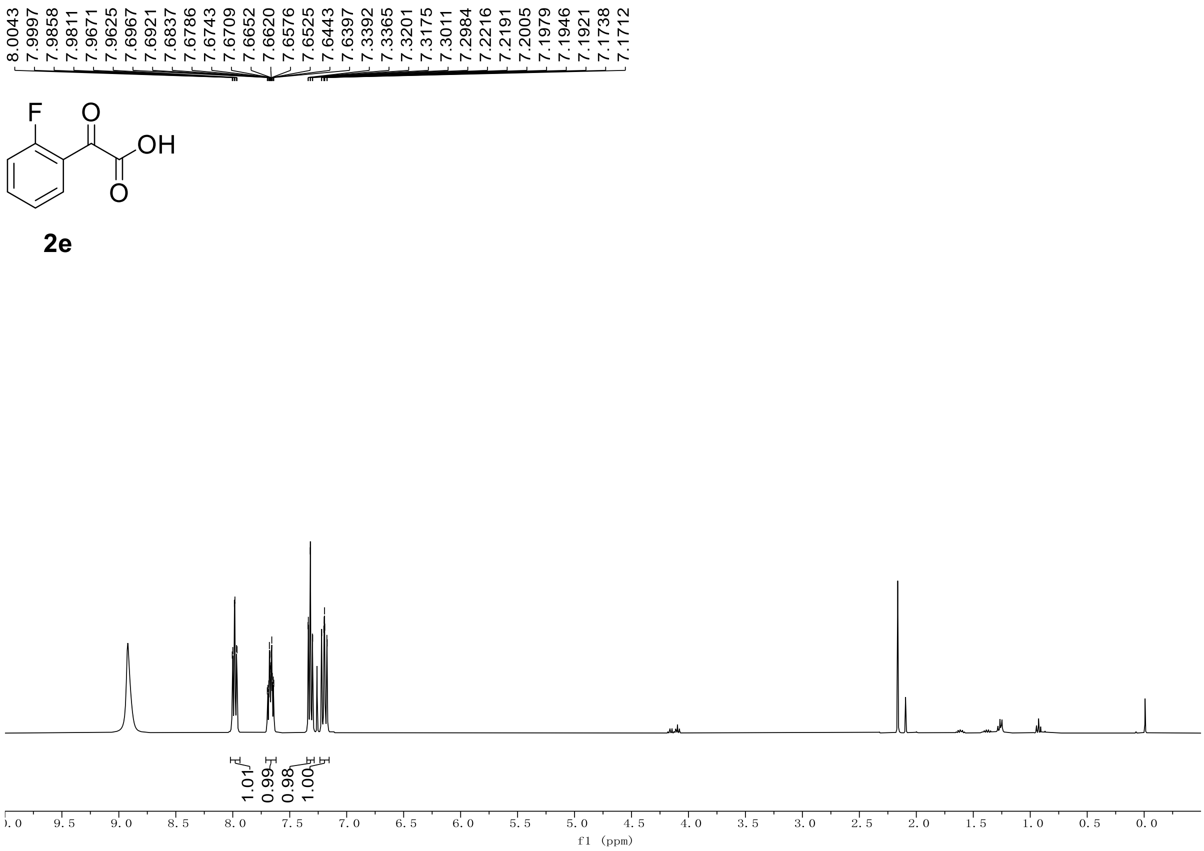

Figure S138 :^{1}H NMR spectrum of **2e** (400 MHz, $CDCl_3$)

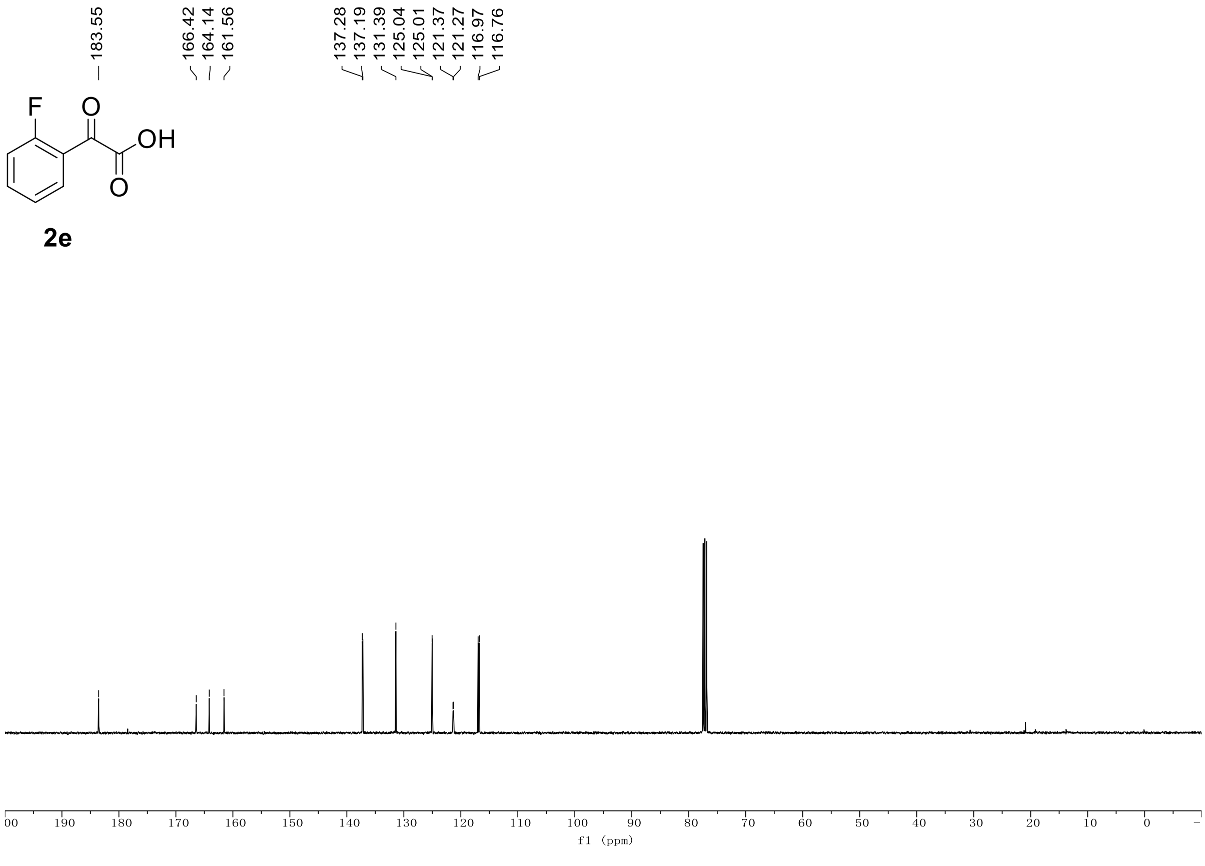

Figure S139 :^{13}C {^{1}H} NMR spectrum of **2e** (100 MHz, $CDCl_3$)

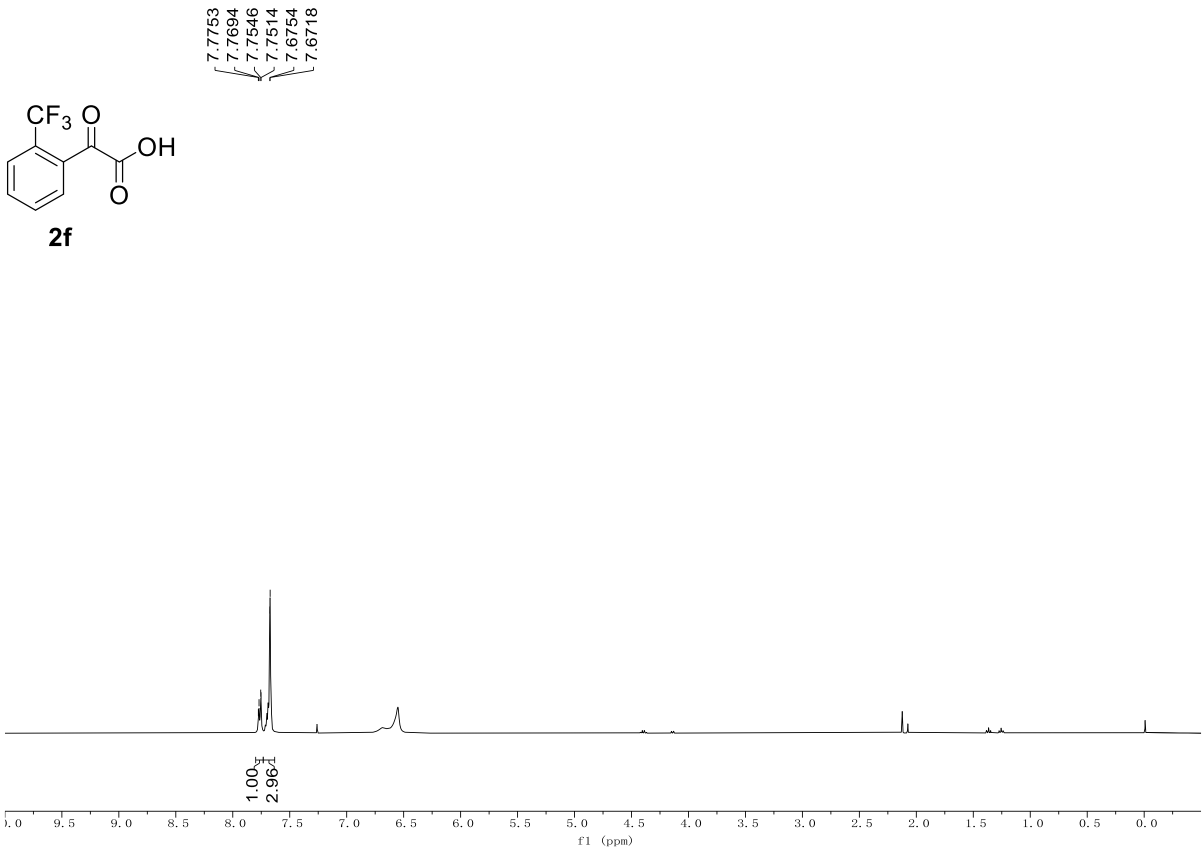

Figure S140 :^{1}H NMR spectrum of **2f** (400 MHz, $CDCl_3$)

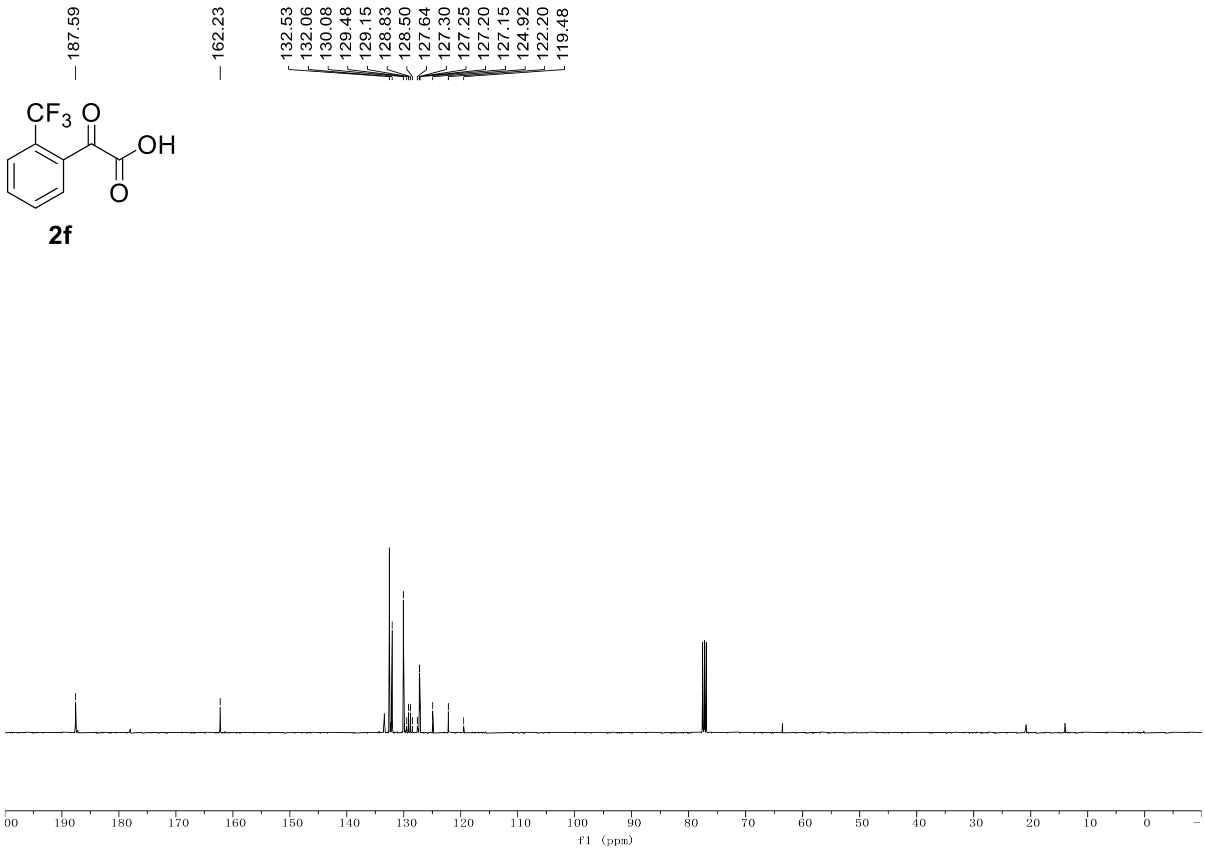

Figure S141 :^{13}C {^{1}H} NMR spectrum of **2f** (100 MHz, $CDCl_3$)

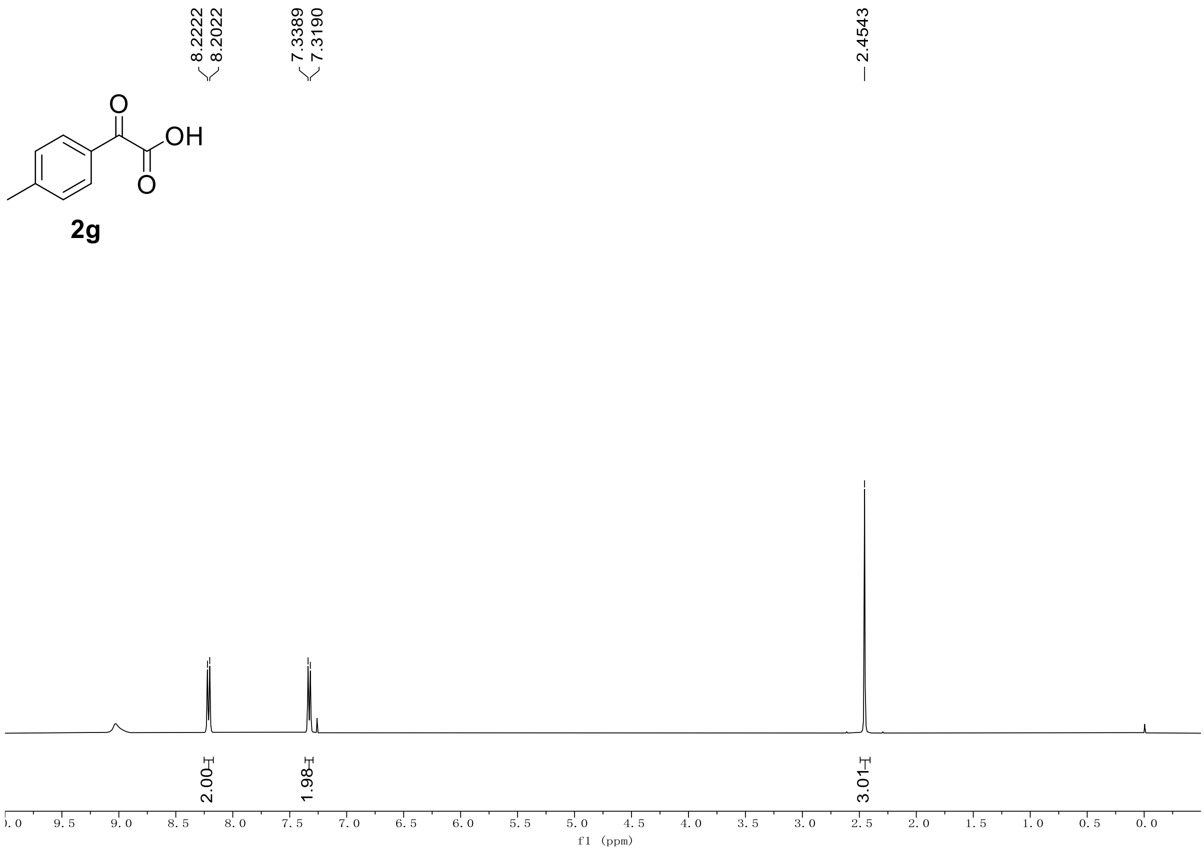

Figure S142 :^{1}H NMR spectrum of **2g** (400 MHz, $CDCl_3$)

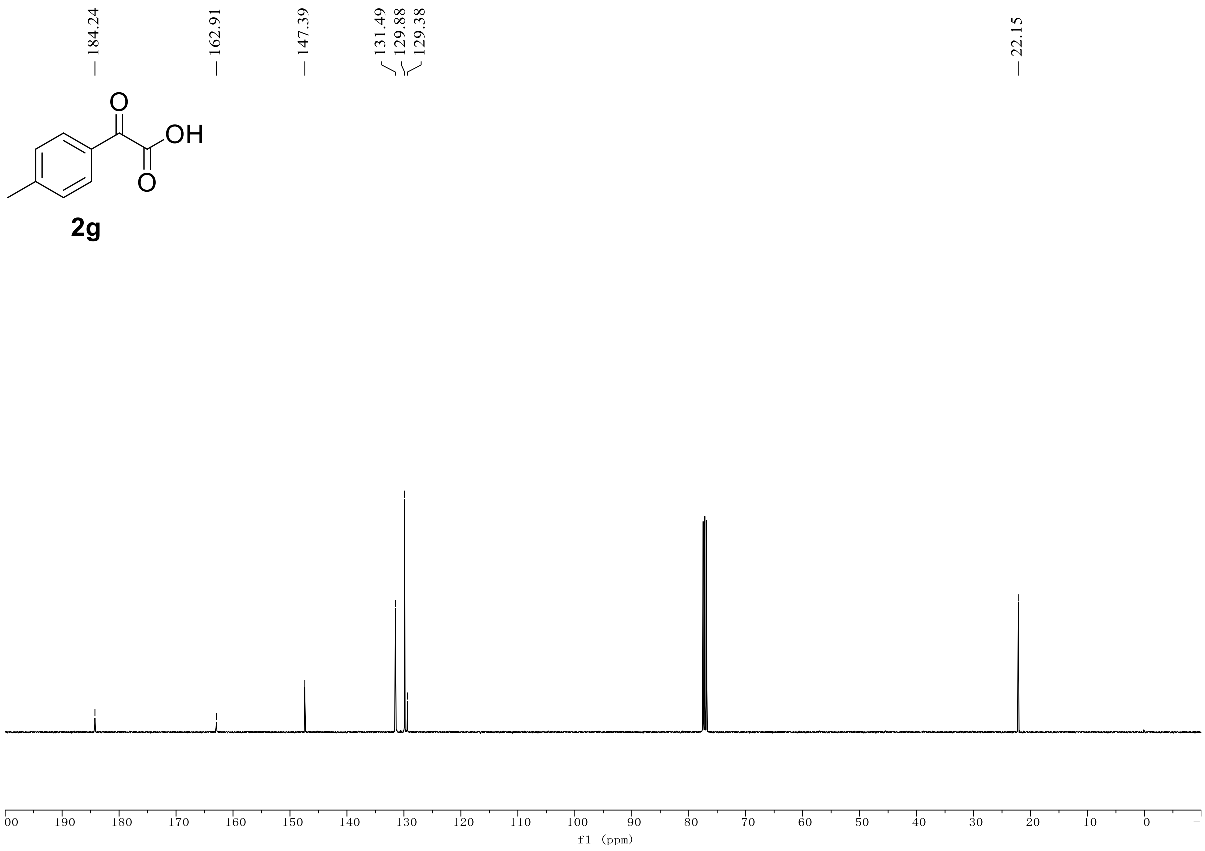

Figure S143 :^{13}C {^{1}H} NMR spectrum of **2g** (100 MHz, $CDCl_3$)

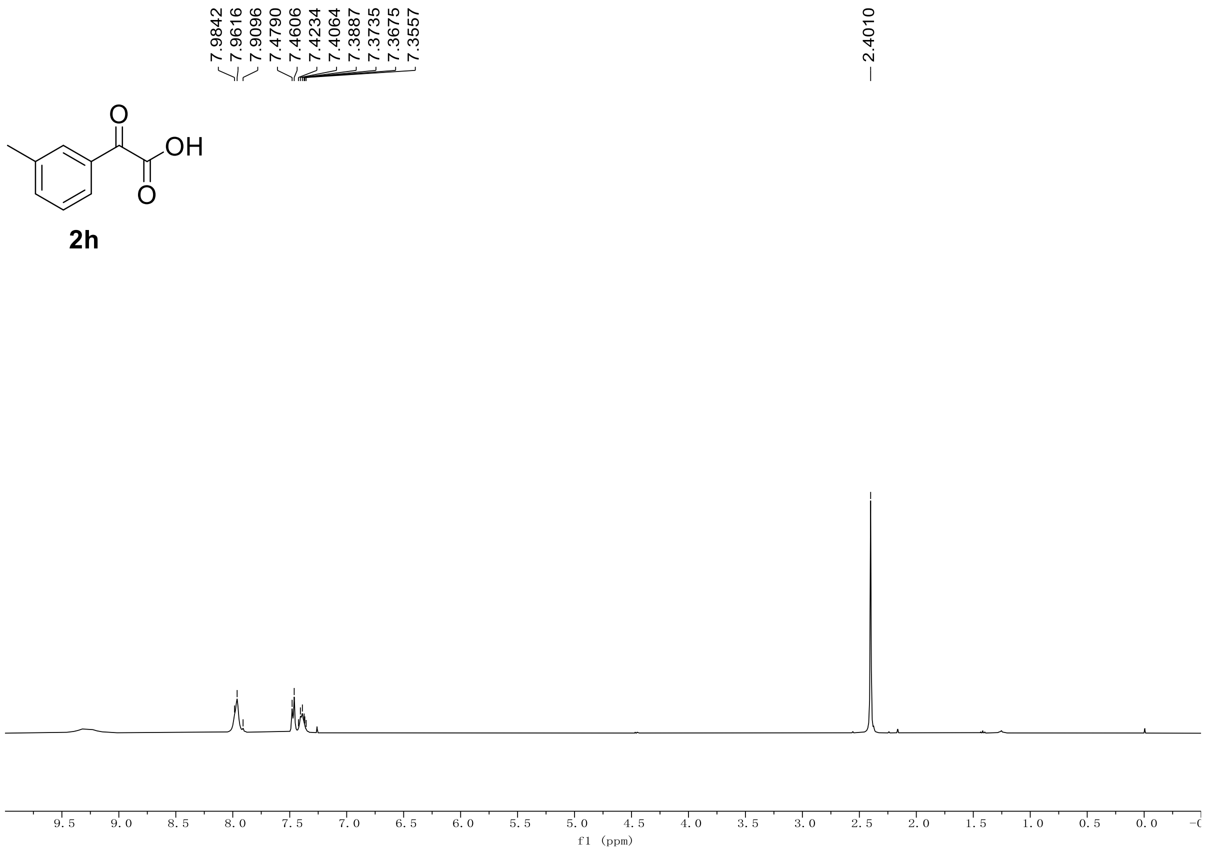

Figure S144 :^{1}H NMR spectrum of **2h** (400 MHz, $CDCl_3$)

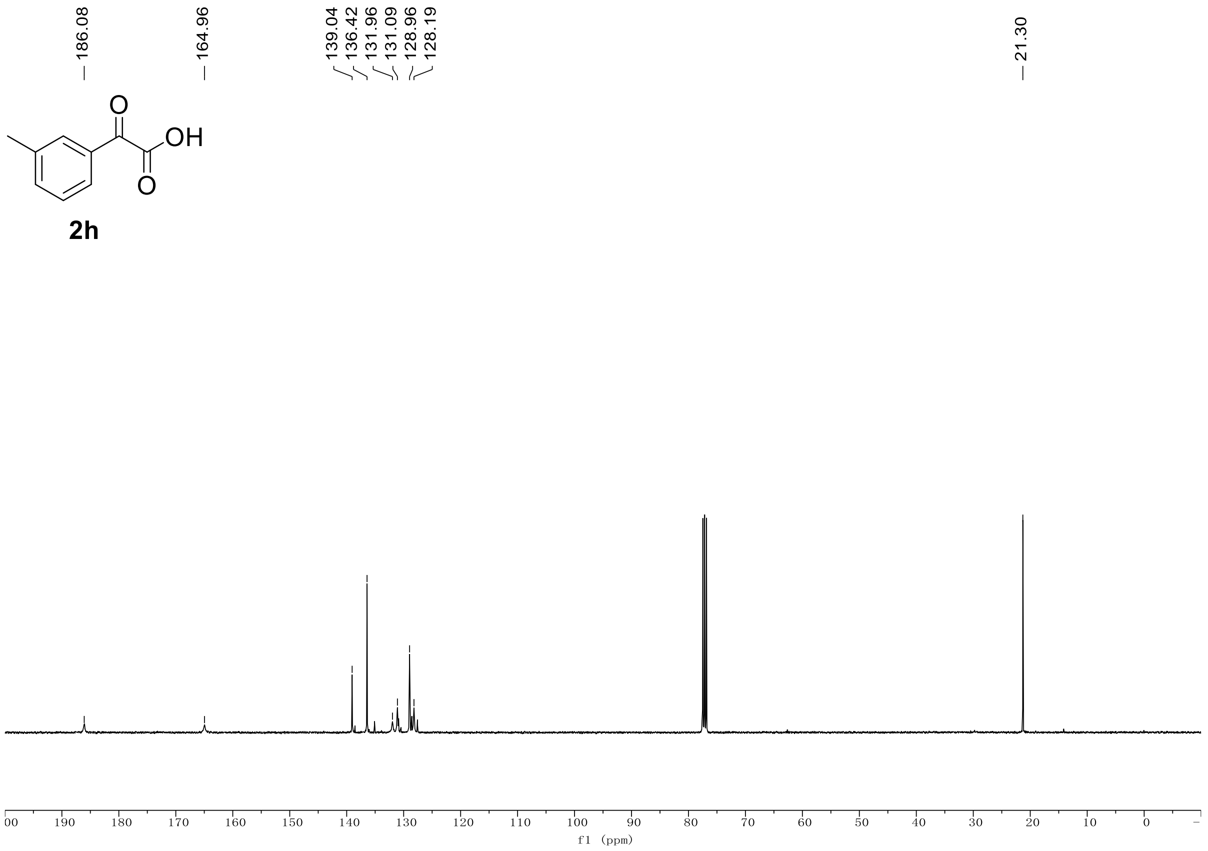

Figure S145 :^{13}C {^{1}H} NMR spectrum of **2h** (100 MHz, $CDCl_3$)

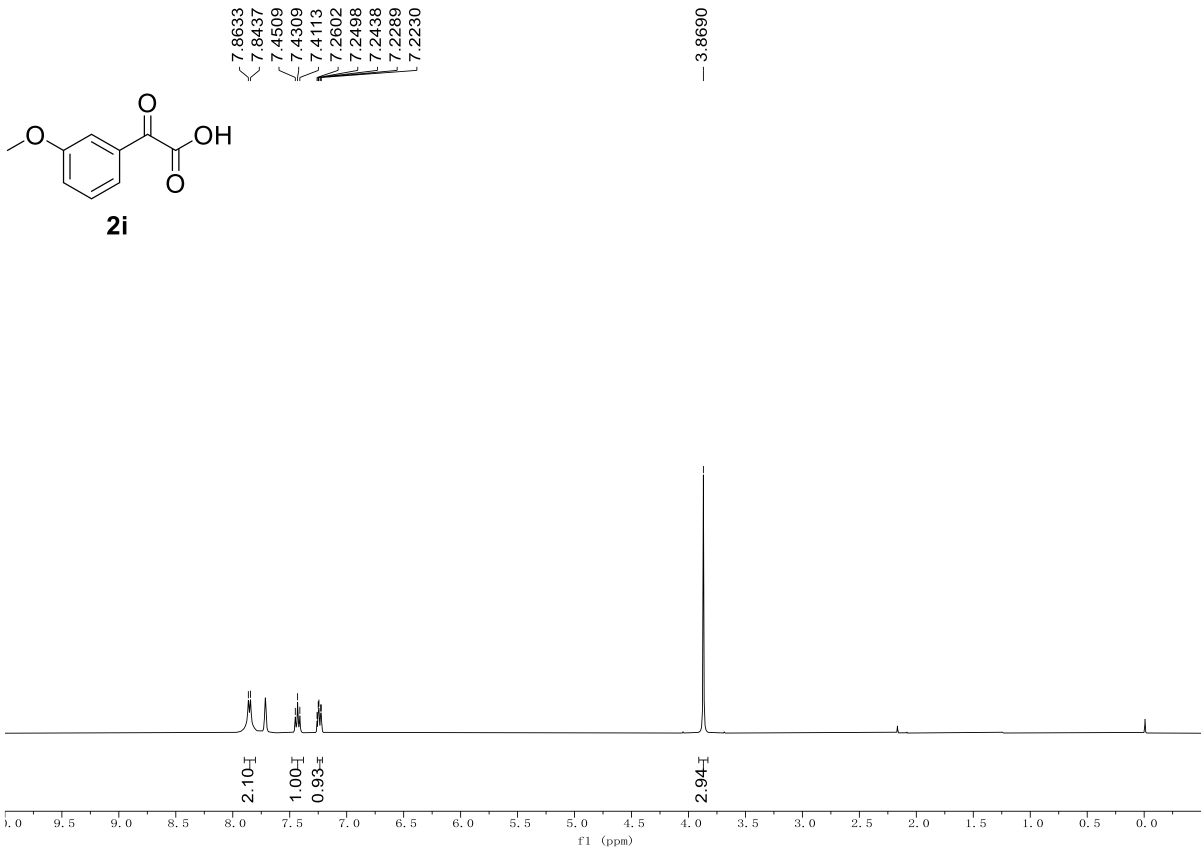

Figure S146 :^{1}H NMR spectrum of **2i** (400 MHz, $CDCl_3$)

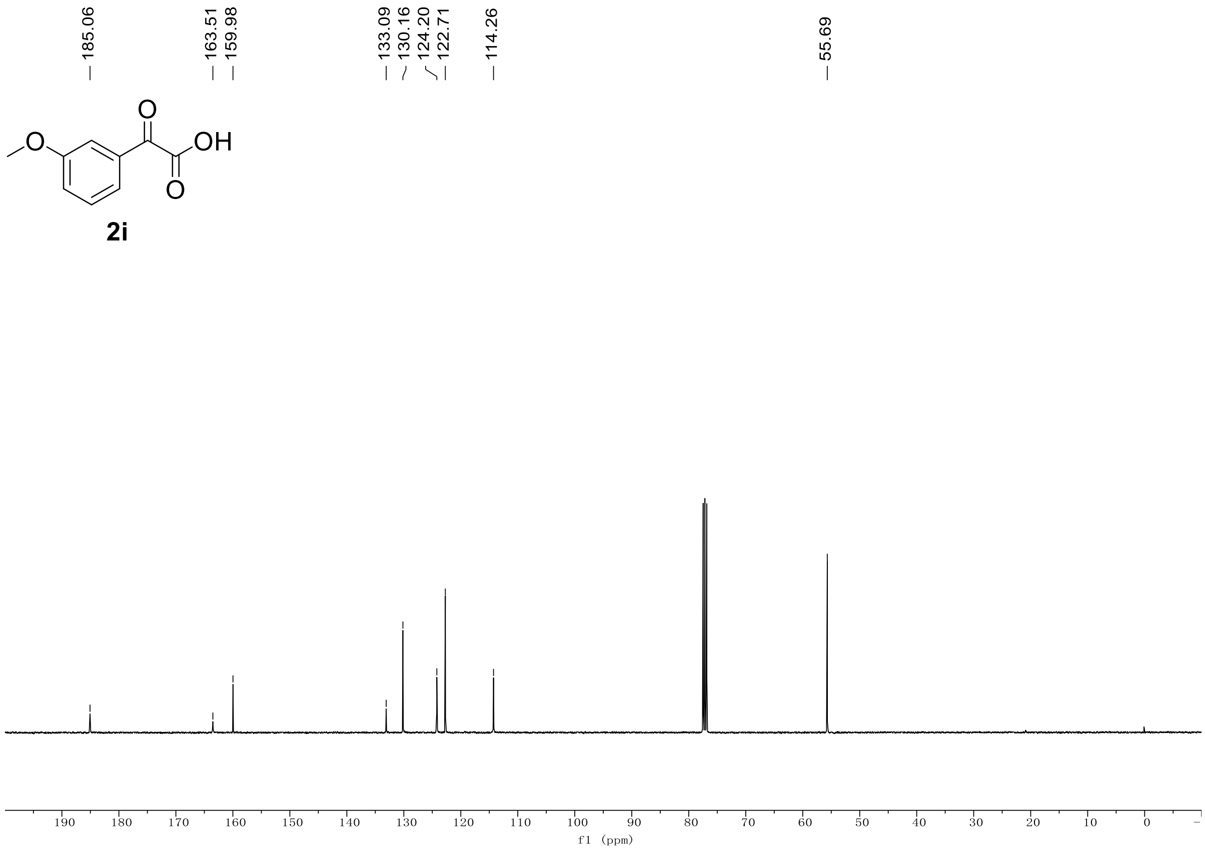

Figure S147 :^{13}C {^{1}H} NMR spectrum of **2i** (100 MHz, $CDCl_3$)

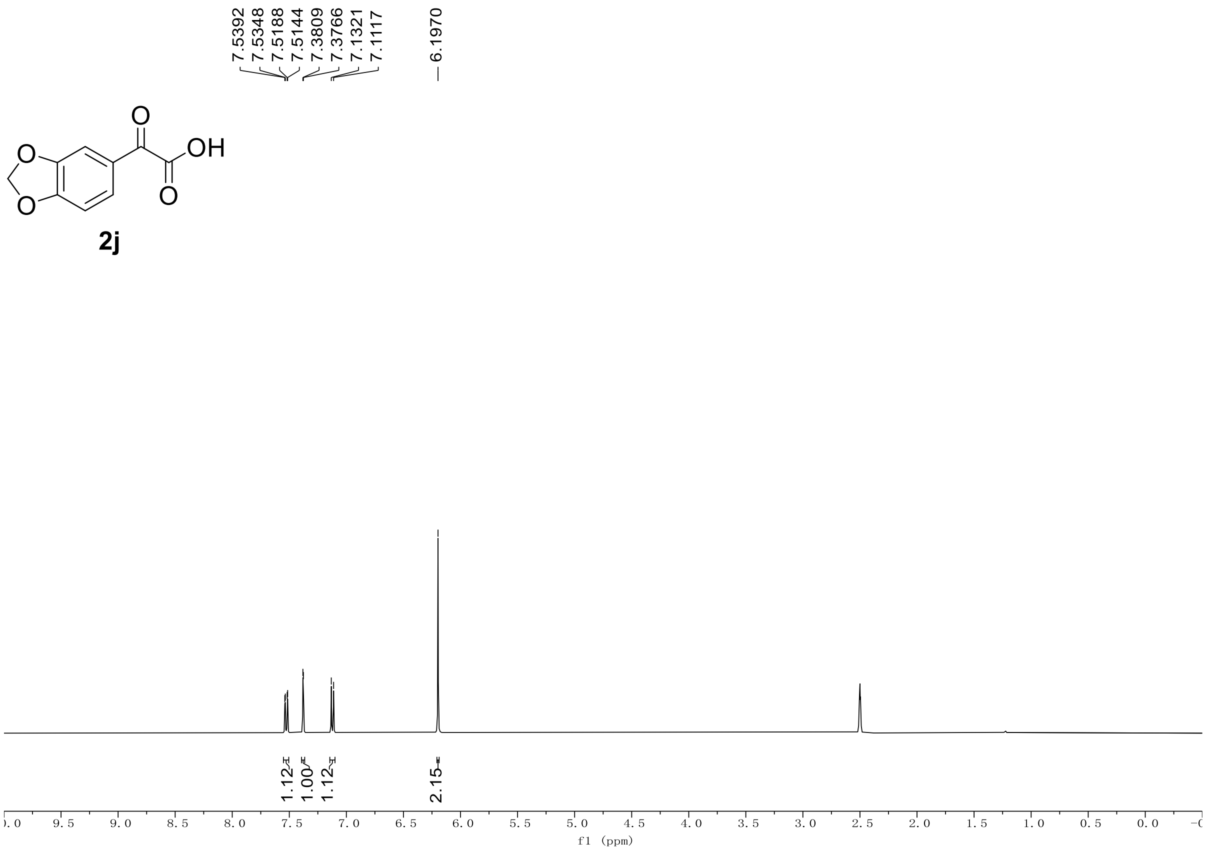

Figure S148 :^{1}H NMR spectrum of **2j** (400 MHz, $(CD_3)_2SO$)

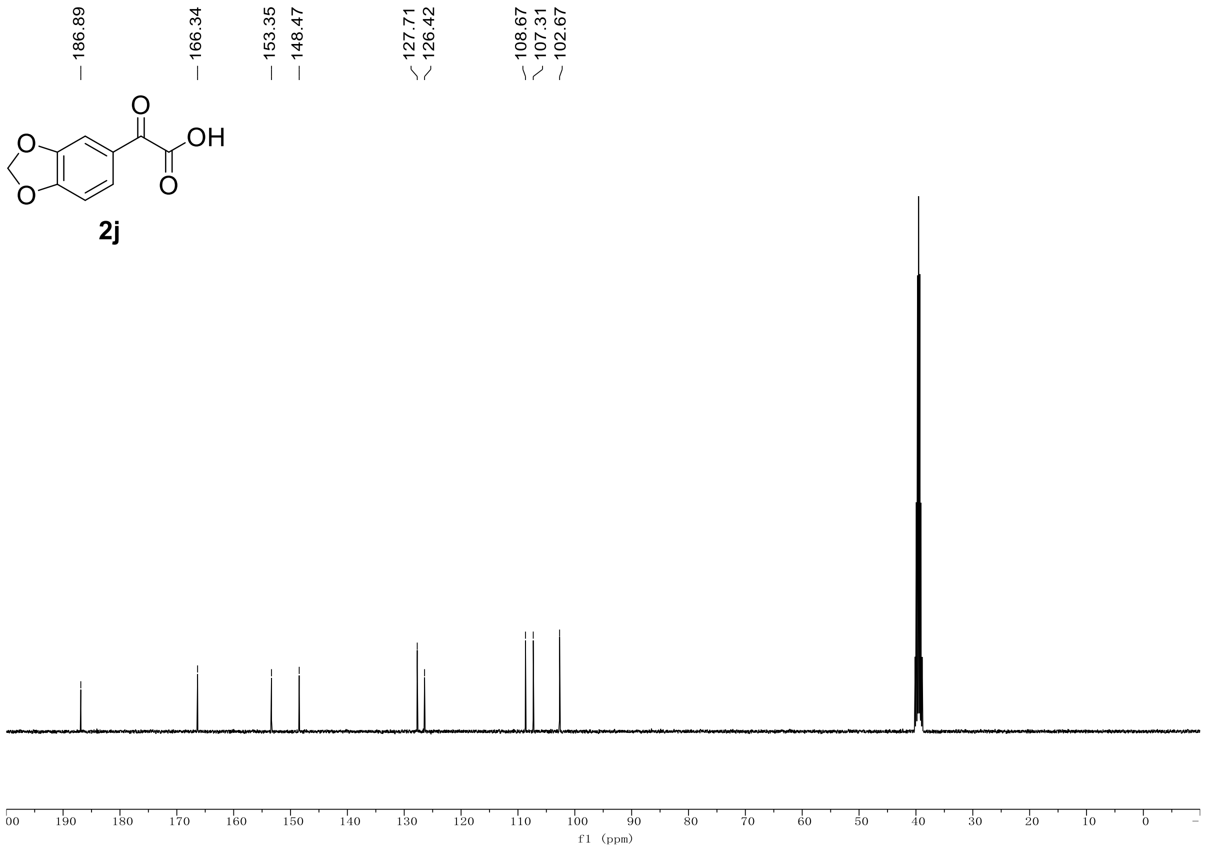

Figure S149 :^{13}C {^{1}H} NMR spectrum of **2j** (100 MHz, $(CD_3)_2SO$)

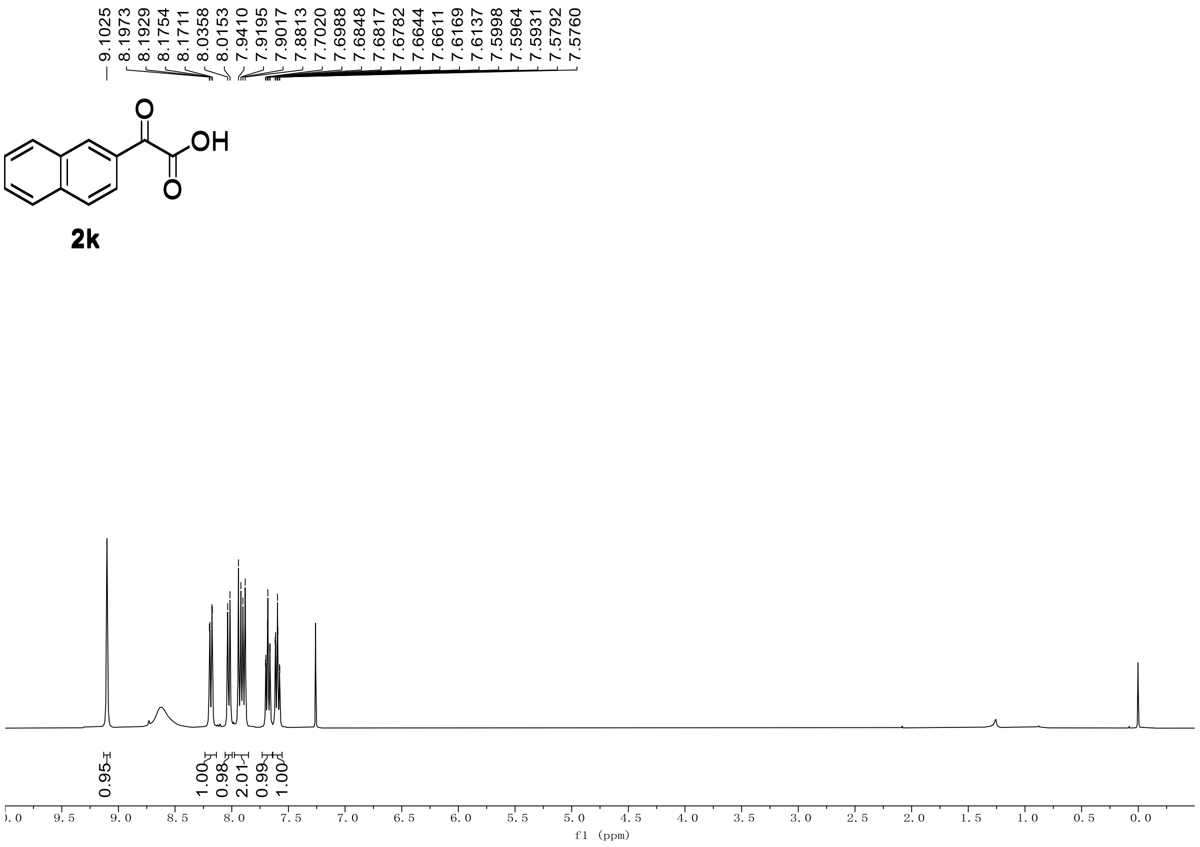

Figure S150 :^{1}H NMR spectrum of **2k** (400 MHz, $CDCl_3$)

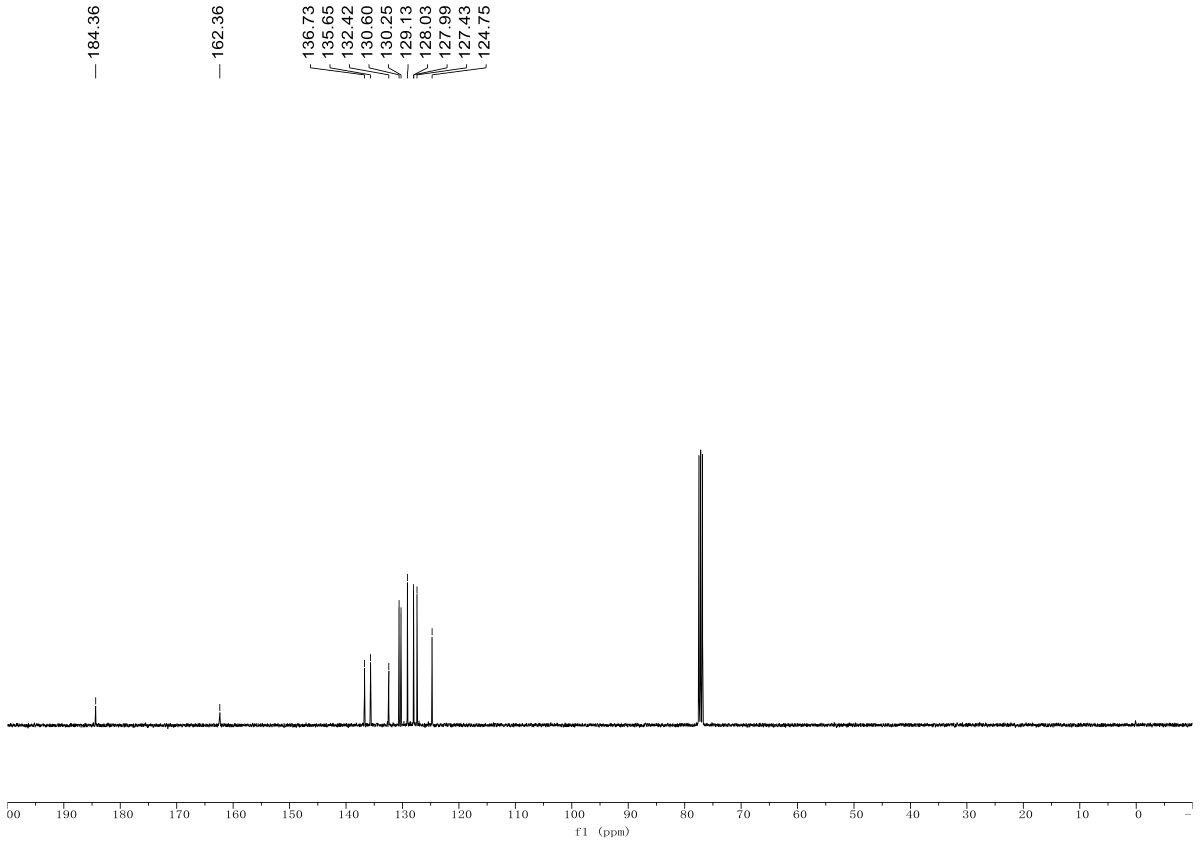

Figure S151 :^{13}C {^{1}H} NMR spectrum of **2k** (100 MHz, $CDCl_3$)
